# Chinese Social Media Reaction to Information about 42 Notifiable Infectious Diseases

**DOI:** 10.1371/journal.pone.0126092

**Published:** 2015-05-06

**Authors:** Isaac Chun-Hai Fung, Yi Hao, Jingxian Cai, Yuchen Ying, Braydon James Schaible, Cynthia Mengxi Yu, Zion Tsz Ho Tse, King-Wa Fu

**Affiliations:** 1 Department of Epidemiology, Jiann-Ping Hsu College of Public Health, Georgia Southern University, Statesboro, Georgia, 30460–8015, United States of America; 2 Department of Biostatistics, Jiann-Ping Hsu College of Public Health, Georgia Southern University, Statesboro, Georgia, 30460–8015, United States of America; 3 Department of Computer Science, The University of Georgia, Athens, Georgia, 30605, United States of America; 4 Journalism and Media Studies Centre, The University of Hong Kong, Hong Kong; 5 School of Journalism and Communication, Peking University, Beijing 100871, China; 6 College of Engineering, The University of Georgia, Athens, Georgia, 30602, United States of America; Georgia State University, UNITED STATES

## Abstract

This study aimed to identify what information triggered social media users’ responses regarding infectious diseases. Chinese microblogs in 2012 regarding 42 infectious diseases were obtained through a keyword search in the Weiboscope database. Qualitative content analysis was performed for the posts pertinent to each keyword of the day of the year with the highest daily count. Similar posts were grouped and coded. We identified five categories of information that increased microblog traffic pertaining to infectious diseases: news of an outbreak or a case; health education / information; alternative health information / Traditional Chinese Medicine; commercial advertisement / entertainment; and social issues. News unrelated to the specified infectious diseases also led to elevated microblog traffic. Our study showcases the diverse contexts from which increased social media traffic occur. Our results will facilitate better health communication as causes underlying increased social media traffic are revealed.

## Introduction

Social media have become increasingly useful in digital disease detection and surveillance [1,2,3,4], such as Twitter data analysis in experimental digital surveillance of influenza [5,6] and cholera [7]. Social media are also used as communication tools in public health [8,9,10,11,12], such as health communications via Twitter on breast cancer and diabetes [13,14].

Equivalent to Twitter, which is blocked by the Chinese authorities, Weibo is a popular microblogging service in China [15]. By the end of 2012, Sina Weibo, the leading service provider, claimed to have more than 500 million registered users [16]. Weibo users discuss a variety of social issues, including post-disaster management and politics [17,18,19]. The Chinese health authorities also use Weibo as a communication tool [20]. Recently, scientists began analyzing Weibo data in light of its implications in public health. For example, Weibo users’ reactions to a self-presented suicide attempt [21] and to the outbreaks of Middle East Respiratory Syndrome coronavirus (MERS-CoV) and avian influenza A(H7N9) merited our attention [22]. A recent study also revealed the great variety of health-related keywords used in Weibo posts [23].

While outbreak detection and incidence prediction are one of the goals of digital epidemiology [4,24], a good theoretical understanding of why people tweet about diseases or health conditions is largely lacking. Our understanding of online reaction to media exposure of disease information should go beyond seeing it as social media “chatter” [5] or as spikes of search queries that reduce the predictive power of digital epidemiological tools [25]. A better understanding will allow public health agencies to improve their health communication strategies and digital epidemiologists to better understand and model the relationship between time series of social media trends and disease incidence trends.

In this article, we extend our previous study [22] to cover keywords associated with all the infectious diseases that were notifiable in mainland China in 2012. Qualitative content analysis was performed to identify the news and information that triggered an elevated volume of microblog traffic (peaks in daily Weibo count on certain keywords). Our results will allow public health practitioners to better formulate their health communication messages and shed light upon the underlying assumption of digital epidemiology that an increase in disease incidence may trigger an increase in social media messages related to the disease.

## Methods

### Data acquisition and sampling

**Fig 1:**
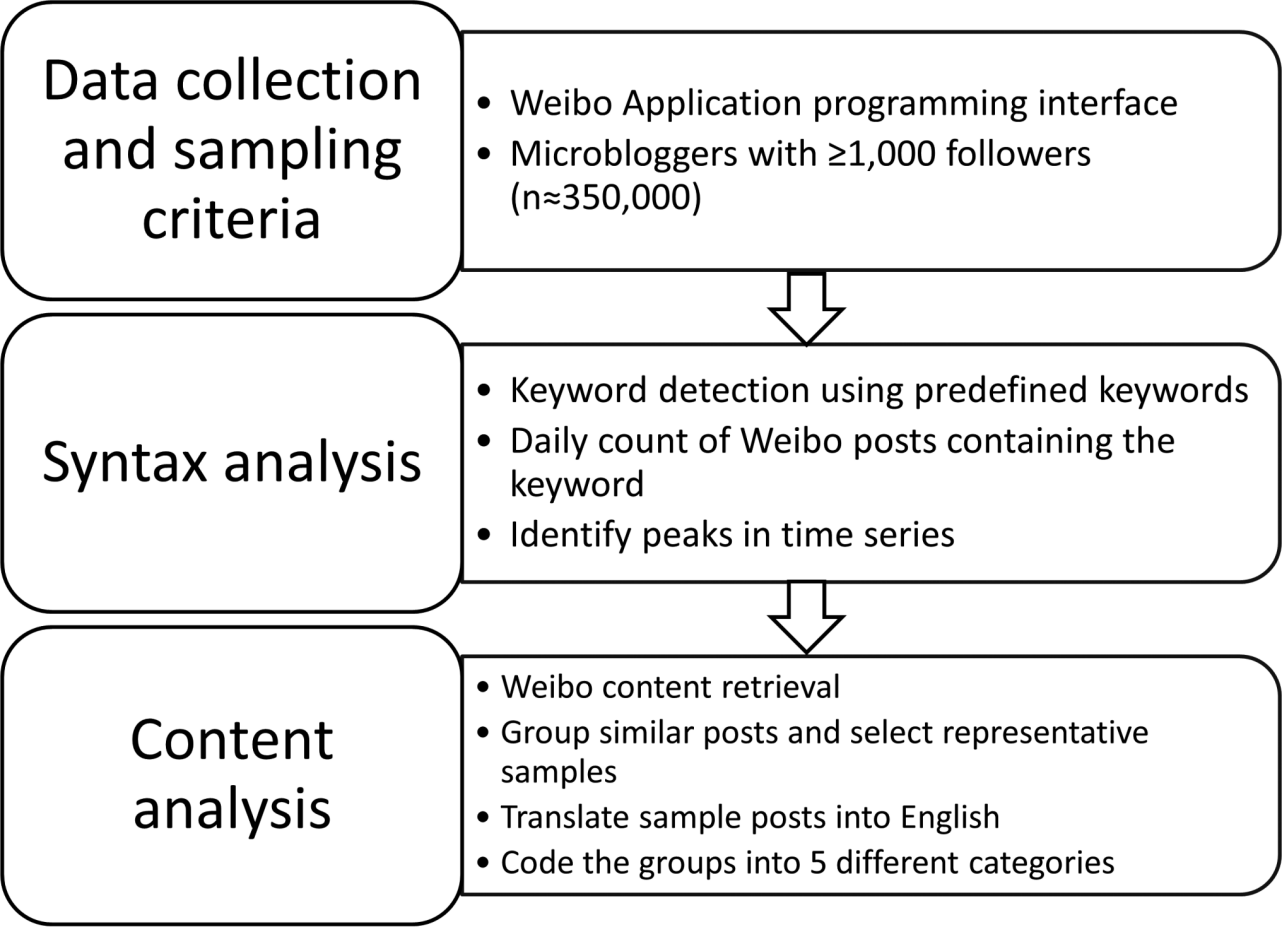
Flowchart of data collection, sampling criteria, syntax analysis and content analysis.

Our Weibo data were collected via the Weiboscope project, maintained by KWF and his team in the University of Hong Kong, as described elsewhere [18,22]. Initially, a list of about 350,000 indexed microbloggers was generated by systematically searching the Sina Weibo user population using the Sina Weibo Application Programming Interface (API) (Fig 1). The users were selected based on an inclusion criterion of having 1,000 followers or more when the project began data collection in 2011. The rationale for our high-follower-count samples was two-fold. First, social media users with high follower count are more influential than those with fewer followers and usually attract disproportionately large public attention [15]. Second, this criterion can exclude the spam accounts that are very common among Chinese social media [26].

As previously described [18], the raw Chinese microblog data were acquired in Comma-Separated Values (CSV) format and sorted by week. In the CSV files, metadata, such as the post content, the created date and user ID, are available for secondary analysis. We de-identified the user IDs by converting them into a different string of characters (known as “hashing”). The user identity codes were obfuscated using an algorithm similar to base64 algorithm to generate the unique but anonymized identifier. The screen names referenced in the posts were replaced with the corresponding obfuscated user identity codes. Each file begins with its properties in the first line, followed by the record of the post [22]. For the purpose of this study, we limited our time frame from January 1, 2012 to December 31, 2012.

### Keyword detection and data analysis

Similar to our previous study [22], we prescribed a list of keywords for keyword search in the dataset. They were based on the list of notifiable infectious diseases in mainland China, as specified by the *Law of the People’s Republic of China on the Prevention and Treatment of Infectious Diseases*, as of 2012 [27] (Table 1; viral hepatitis is counted as one disease in the law, but we counted Hepatitis A, B, C and E separately following the disease reporting scheme of the Chinese Center for Disease Control and Prevention). We then obtained a time series of aggregated daily counts of Weibo posts containing a specified keyword for each disease. We selected the day of the year with the largest number of posts pertinent to each disease and, using an online platform that we developed, obtained the content of the Chinese posts that were generated on that day. If there were two peaks of equal magnitude, we obtained the content of both. We obtained the microblog content of the whole year of 2012 for typhus (n = 14) and leishmaniasis (n = 24), given their small numbers. Three co-authors manually read the retrieved microblog content, grouped them by topics (e.g. sharing the same piece of news), performed preliminary coding, and counted them. A representative post was selected for each group. They manually searched online and identified the relevant news, events or information that triggered the elevated microblog traffic. Afterwards, the first author manually performed quality check by selecting the microblog posts of about one-third of the list of diseases and performed the grouping himself. He revised the groupings if necessary. He reviewed all the groupings and preliminary coding of the microblog contents and their representative posts, for emerging themes to develop the following coding scheme. For the major news, events or information that triggered the highest peak of daily microblog post count for each notifiable disease in mainland China in 2012, the first author coded them into the following categories (Table 1):

1. News of (a) outbreaks or (b) cases;
2. Health education or information, as released by health authorities, news agencies, education institutions, etc., that conform to modern scientific medicine;
3. Alternative health information that fell out of the realm of modern scientific medicine (such as information of Traditional Chinese Medicine);
4. Commercial advertisements or entertainment; and
5. Social issues, including the conflicts between the medical profession and the patients (Table 2).

**Table 1:** List of infectious diseases notifiable in mainland China according to the Law of the People’s Republic of China on the Prevention and Treatment of Infectious Diseases (as of 2012), and the corresponding keywords used to search Weibo posts.

Simplified Chinese	English translation*	Chinese keyword used to search Weibo posts	Notes / References
甲类传染病	**Class A**		
1. 鼠疫	Plague	鼠疫	
2. 霍乱	Cholera	霍乱	
乙类传染病	**Class B**		
3. 传染性非典型肺炎	Severe Acute Respiratory Syndrome	非典	Please refer to Fung et al. [22] for more details.
4. 艾滋病	HIV/AIDS	艾滋	
病毒性肝炎	Viral hepatitis		Viral hepatitis was listed as a single disease in the law. The Chinese Center for Disease Control and Prevention reports the total number of viral hepatitis per month, and its sub-categories: A, B, C, E and un-typed. We decided to study Weibo content about hepatitis A, B, C and E only.
5. 甲型肝炎	Hepatitis A	甲肝	
6. 乙型肝炎	Hepatitis B	乙肝	
7. 丙型肝炎	Hepatitis C	丙肝	
8. 戊型肝炎	Hepatitis E	戊肝	
9. 脊髓灰质炎	Poliomyelitis	脊髓灰质炎	
10. 人感染高致病性禽流感	Human infections of highly pathogenic avian influenza	禽流感	
11. 甲型H1N1流感†	Influenza A(H1N1)	H1N1	Listed as a separate Category B entity from 2009 to 2013.
12. 麻疹	Measles	麻疹	
13. 流行性出血热	Epidemic hemorrhagic fever	出血热	
14. 狂犬病	Rabies	狂犬病	
15. 流行性乙型脑炎	Epidemic Encephalitis B	流行性乙型脑炎, 乙脑, 日本脑炎	We searched three terms that refer to the same disease. They are流行性乙型脑炎 (Epidemic Encephalitis B), 乙脑 (short form for Epidemic Encephalitis B) and 日本脑炎 (Japanese encephalitis).
16. 登革热	Dengue	登革热	
17. 炭疽	Anthrax	炭疽	
18. 细菌性和阿米巴性痢疾	Bacterial and amoebic dysentery	痢疾	
19. 肺结核	Tuberculosis	肺结核	
20. 伤寒和副伤寒	Typhoid and paratyphoid	伤寒	Our keyword search did not distinguish typhoid and paratyphoid.
21. 流行性脑脊髓膜炎	Epidemic (meningococcal) meningitis	脑膜炎, 脑脊髓膜炎,流脑	We searched three terms that refer to the same disease. They are 脑膜炎(meningitis), 脑脊髓膜炎(cerebrospinal meningitis) and流脑(short form for epidemic meningitis).
22. 百日咳	Pertussis	百日咳	
23. 白喉	Diphtheria	白喉	
24. 新生儿破伤风	Neonatal tetanus	破伤风	
25. 猩红热	Scarlet fever	猩红热	
26. 布鲁氏菌病	Brucellosis	布鲁氏菌病	
27. 淋病	Gonorrhea	淋病	
28. 梅毒	Syphilis	梅毒	
29. 钩端螺旋体病	Leptospirosis	钩端螺旋体病	
30. 血吸虫病	Schistosomiasis	血吸虫病	
31. 疟疾	Malaria	疟疾	
丙类传染病	**Class C**		
32. 流行性感冒	Influenza	流行性感冒，流感	We use both the full term (流行性感冒) and its short form (流感) for keyword search.
33. 流行性腮腺炎	Mumps	腮腺炎	
34. 风疹	Rubella	风疹	
35. 急性出血性结膜炎	Acute hemorrhagic conjunctivitis	结膜炎	
36. 麻风病	Leprosy	麻风	
37. 斑疹伤寒	Typhus	斑疹伤寒	
38. 黑热病	Leishmaniasis	黑热病	
39. 包虫病	Echinococciosis	包虫病	
40. 丝虫病	Filariasis	丝虫病	
41. 其它感染性腹泻病	Other infectious diarrheal diseases	腹泻	
42. 手足口病	Hand, foot and mouth disease	手足口病	

*Class A and B diseases require immediate notification to health authorities of a higher administrative level; Class C diseases require periodical notification. Class A refers to diseases highlighted in the International Health Regulations [28] that may constitute a public health emergency of international concern, of which cholera and plague (listed in the law) are (or were) endemic in parts of China. The law specified that outbreaks of class A diseases require prompt infection control measures including immediate isolation of patients and quarantine (medical observation) of their contacts. Collection, transportation and testing specimens of Class A pathogens require approval from the provincial authorities or the central government [27].

† Influenza A(H1N1) was classified as a Class B infectious disease and was added to the list as a separate entity during the 2009 pandemic [29]. On October 28, 2013, it was announced by the Chinese government that starting from January 1, 2014, influenza A(H1N1) would cease to be listed separately and would be grouped with other influenza strains as an aggregated number listed in Class C [30].

**Table 2:** News and information that led to a peak in Weibo traffic as found in the WeiboScope database, January 1 to December 31, 2012.*

Category	Disease	Peak date	Total count on peak date	News / Information	Count (%)
1a News (outbreak)					
	2. Cholera	Oct 10	84	Outbreak in Huangshi, Hubei Province	73 (87%)
	7. Hepatitis C	Feb 23	100	Outbreak in Zijin County, Guangdong Province	92 (92%)
	11. Influenza A(H1N1)	Mar 12	42	Outbreak in Hotan, Xinjiang Uyghur Autonomous Region	29 (70%)
	17. Anthrax	Aug 14	103	Outbreak in Liaoling Province	61 (59%)
1b News (cases)					
	10. Avian influenza	Jan 2	264	A fatal case of human infection of avian influenza A(H5N1) in Shenzhen, Guangdong Province	134 (51%)
	13. Epidemic hemorrhagic fever	Oct 16	22	An imported case of Crimean-Congo Hemorrhagic Fever in the United Kingdom (a comment on the “Armageddon” virus made by a British professor)	22 (100%)
	15. Epidemic encephalitis B	Aug 5	30	A fatal case in Ningbo City, Zhejiang Province	
	25. Scarlet Fever	Jan 20 (Peak 1)	6	Two cases of scarlet fever in Hong Kong	5 (83%)
	31. Malaria	Aug 22	38	An imported case of severe malaria in Beijing (a patient’s wife uses Weibo to recruit blood donors)	33 (87%)
	39. Echinococciosis	Mar 21	113	A case of echinococciosis leading to paralysis	113 (100%)
	41. Diarrhea	Jun 14	454	Cases of diarrhea	242 (53%)
				*Two cases of diarrhea after consumption of allegedly contaminated ham*	*196 (43%)*
				*Cases of diarrhea after consumption of allegedly contaminated milk*	*24 (5%)*
				*A case of diarrhea in a swimming pool*	*14 (3%)*
				*A case of diarrhea after consumption of allegedly contaminated mixed ingredient porridge*	*7 (2%)*
				*A case of infant diarrhea (“A shop assistant mistook pregnant women formula for infant formula and sold it to consumers*. *This led to continuous diarrhea of a 4-month-old baby*.*”)*	*1 (0*.*2%)*
2 Health information					
	4. HIV/AIDS	Dec 1	2046	Health education related to World AIDS Day†	1204 (59%)
	5. Hepatitis A	Jan 13 (Peak 1)	18	“Unclean scallions can lead to hepatitis A”	16 (89%)
		Jan 15 (Peak 2)	18	Same as above	18 (100%)
	8. Hepatitis E	Dec 10	17	A conference on hepatitis E epidemiology and risk	14 (82%)
	9. Poliomyelitis	Dec 6 (Peak 1)	11	National Immunization Day	11 (100%)
		Dec 15 (Peak 2)	11	Same as above	11 (100%)
	12. Measles	Jan 4	77	Health information about four diseases with symptoms similar to common cold	68 (88%)‡
	20. Typhoid	Sep 21	29	Old news about a vending machine in Manhattan that sold “dirty water” (UNICEF’s Tap Project, World Water Week 2010)	16 (55%)
	22. Pertussis	Oct 27	38	Conversations related to US CDC Advisory Committee on Immunization Practice recommendation on Tdap for pregnant women (Note: US CDC press release was posted on Oct 24, 2012) and pertussis incidence in US and China	24 (63%)
	23. Diphtheria	Oct 27	8	Conversations related to US CDC Advisory Committee on Immunization Practice recommendation on Tdap for pregnant women (Note: US CDC press release was posted on Oct 24, 2012)	7 (88%)
	24. Tetanus	Jul 27	35	“Should not use Band-Aid for three types of wounds”	31 (86%)
	25. Scarlet Fever	May 16 (Peak 2)	6	“How to take care of ill children infected with scarlet fever”	6 (100%)
	26. Brucellosis	Sep 5	9	Information about *Brucella canis*	9 (100%)
	29. Leptospirosis	Oct 27	8	“Zoonotic infectious diseases common in both human and dogs”	8 (100%)
	33. Mumps	Jan 4	74	Health information about four diseases with symptoms similar to common cold	67 (91%)‡
	34. Rubella	Aug 18	26	Early prevention of childhood diseases in spring	10 (38%)
	35. Conjunctivitis	Dec 16	77	“90% of eye drops available in the market contain preservatives. Doctors advice use with caution.”	70 (91%)
	40. Filariasis	Aug 9–10	5+5	“After bitten by mosquitoes what diseases are we likely to get”	4+5 (90%)
3 Alternative health information / TCM	18. Dysentery	Feb 22	59	“Food that cannot be consumed together with chicken meat”	58 (98%)
4 Commercial advertisement / Entertainment					
	1. Plague	Jan 27	32	Conversation about a Korean pop star	31 (97%)
	16. Dengue	Sep 9	36	Commercial advertisement of holidays in Thailand with mosquito repellent as a gift to prevent dengue	36 (100%)
	32. Influenza	Dec 8	441	The story in the movie *2012* described an earthquake in Japan on Dec 7, 2012 and the occurrence of a new strain of influenza in Europe on Dec 18, 2012. As Japan did experience a strong earthquake on Dec 7, 2012, many Weibo users wondered whether the other “predictions” of the movie might come true.	384 (87%)
	36. Leprosy	Feb 4	25	“Inspire China” (Gandong Zhongguo) 2011 Award Ceremony. One of the awardees, Zhang Pingyi, was a Taiwanese reporter-philanthropist who raised money to build a school for kids whose parents were leprosy patients in a rural village in Sichuan Province.	24 (96%)
5 Social issue					
	3. SARS	Jul 25	214	Rainstorm and flooding in Beijing led to many deaths	169 (79%)
	6. Hepatitis B	Feb 26	103	Petition to end discrimination against HBV carriers	101 (98%)
	21. Epidemic meningitis	Aug 18	1485	Comments on a suspected case of meningitis / Distrust of the medical profession in China	1475 (99%)
	27. Gonorrhea	Mar 20	113	Body check for female civil servant recruits: the issue of gender equality	62 (55%)
	28. Syphilis	Aug 4	207	A case of denial of employment due to a positive test result of syphilis: loss of public trust of the hospitals andthe government	168 (81%)
	30. Schistosomiasis	Sep 25	54	News of a case of unilateral renal agenesis and suspected medical malpractice: tension between a medical doctor and a patient	54 (100%)
	42. Hand-foot-and-mouth disease	May 23	121	News of a hospital cashier who asked patients who could not wait to go to another hospital (It was during an epidemic of hand-foot-and-mouth disease in China)	63 (52%)
6 Others					
	14. Rabies	May 30	692	Comments on the Miami cannibal attack in the United States	675 (98%)
	19. Tuberculosis	Dec 31	95	News related to a case of alleged child torture (The suspect pleaded for mercy as her father suffered from tuberculosis and her mother had mental disability.)	90 (95%)

AIDS: Acquired Immunodeficiency Syndrome; CDC: Centers for Disease Control and Prevention; HBV: Hepatitis B virus; HIV: Human Immunodeficiency Virus; TCM: Traditional Chinese Medicine.

*Two diseases were excluded from this table as their total numbers of posts were too small: typhus (n = 14) and leishmaniasis (n = 24). We obtained and analyzed the Weibo content of the whole year of 2012 for these two diseases (see S1 File).

†Including Weibo posts of health statistics and of news about political figures’ involvement in World AIDS Day.

‡One repost of that post was truncated by the Weibo user who posted it, and “mumps” went missing in that post.

The representative post for each group was then translated into English to be presented in S1 File. Please refer to S1 File for detailed content analysis for the peak of daily microblog post count for each disease in 2012.

### Ethics Statement

The protocol of data processing and anonymization was approved by the Human Research Ethics Committee for Non-Clinical Faculties, The University of Hong Kong, and by the Institutional Review Board, Georgia Southern University (H14167). This paper is the report of a secondary data analysis of the WeiboScope dataset of 2012. All the Weibo posts in the original WeiboScope dataset were publicly available posts. No attempt was made to inform Weibo users of the current study. The data had been de-identified before the current analysis began.

## Results

### 1(a) News (outbreaks)

**Fig 2:**
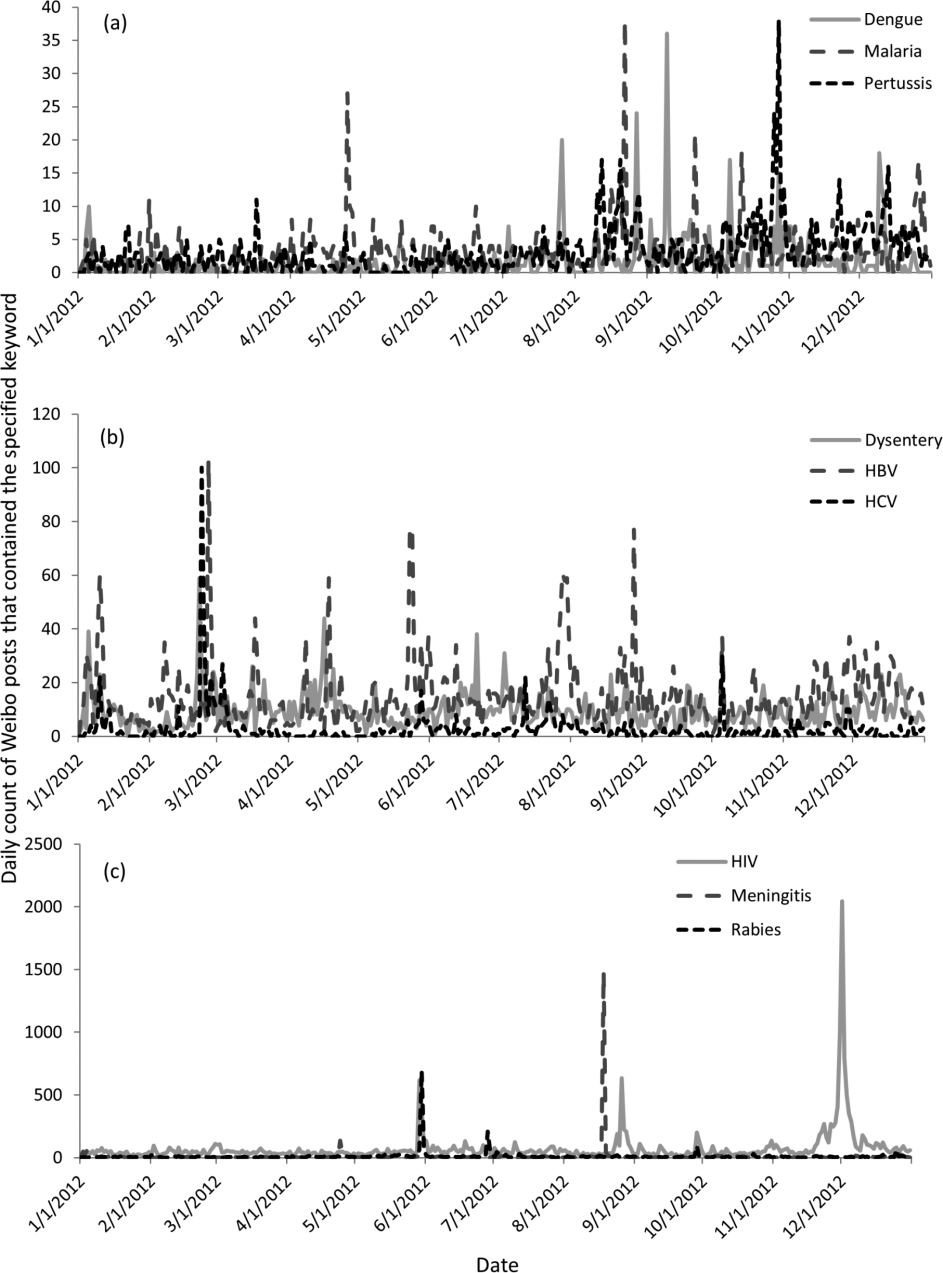
Sample time series of daily count of Weibo posts containing keywords pertaining to selected diseases in 2012 in WeiboScope database (posts generated by Weibo users with 1000 followers or more). Nine diseases are chosen as illustrations for the magnitude of the peaks and are grouped accordingly: (a) dengue, malaria and pertussis; (b) dysentery, hepatitis B and hepatitis C; (c) HIV, meningitis and rabies.

Chinese microbloggers reacted to news about outbreaks of cholera, hepatitis C, influenza A(H1N1) and anthrax in different parts of China (Table 2). An example was the news of an outbreak of hepatitis C infection in Zijin County, Guangdong Province (Fig 2; Table 3). Allegedly, syringes in the health clinic in the township were used repeatedly. Over 90% of the posts in the peak were news posts (or re-posts). Other posts include personal comments, including the personal experience of a journalist who investigated the case and who overcame many hurdles before the report was published (see S1 File for details).

**Table 3:** Categories and examples of Weibo posts that led to a peak in Weibo traffic as found in the WeiboScope database (January 1 to December 31, 2012).

	Category	Example	Sample Weibo post (English translation)
1a	News of an outbreak	Outbreak of hepatitis C infection in Zijin County, Guangdong Province	“Nearly 200 people in Guangdong have been infected with hepatitis C, potentially due to repeated use of syringes.” According to the *People’s Daily*, residents in Zijin County, Guangdong, reported that nearly 200 people, who lived along Xiangshui Road in that county, have been infected with hepatitis C, through unknown routes of transmission. According to the report, all the patients who were infected have been vaccinated in the health center in the township. That health center did not use single-use syringes. Guangdong Department of Health has instructed the Zijin County Bureau of Health to investigate. Medical experts of Guangdong Province have also rushed to Zijin County to participate in the investigation. http://t.cn/zOUVCqH
1b	News of a case	A case of a severe malaria infection in a Chinese worker who had returned from Africa	Comrade Zhang Liang suddenly returned to China from his business trip in Africa because his mother was dying from illness. Being back at home taking care of his mother for 3–4 days made him very tired. When he fell ill, because he did not carry any anti-malarial medication with him, he could only return to Beijing for treatment. It is really sad. Pray for Zhang Liang.
2	Health information	The recommendations made by the Advisory Committee of Immunization Practice of the U.S. Centers for Disease Control and Prevention that all pregnant women should receive Tdap 3-in-1 vaccine	United States CDC Advisory Committee on Immunization Practice (ACIP) recommends that for every pregnancy, women should receive Tdap (tetanus, diphtheria and pertussis) 3-in-1 vaccine. The advisory committee said that it is safe for pregnant women to receive the vaccination against pertussis and all pregnant women should receive that vaccine. The best timing is the last trimester of pregnancy. @Vaccine and Science
3	Alternative health information / Traditional Chinese medicine	A post (and its reposts) about “food that cannot be mixed with chicken meat”, including certain traditional Chinese medicine	“Food that cannot be consumed together with chicken meat” (1) sesame (can lead to death); (2) sweet potato (leading to abdominal pain); (3) anti-inflammatory tablet (poisoning); (4) *Penis et Testis Canis* (leading to dysentery); (5) Chinese plum (if eaten, dysentery); (6) soya milk (lower protein absorption); (7) Glutinous rice (can lead to bodily discomfort); (8) Chrysanthemum (can lead to death); (9) mustard (damages vitality); (10) garlic (the nature and taste of the foods do not match); (11) carp (the nature and taste of the foods do not match); (12) *Carassius auratus* (the nature and taste of the foods do not match).
4	Commercial advertisement / Entertainment	An advertisement about holidays in Thailand. The travel agency gave customers mosquito repellent cream as a gift to prevent dengue.	#Visit Thailand Mosquito repellent cream as a gift Prevent dengue# Circulate it. I believe that I can generate good luck by circulating it!!! Address: http://t.cn/zWgiE7d
5	Social issue	A petition to end discrimination against hepatitis B virus carriers, addressed to the Chinese People’s Congress and the Chinese People’s Political Consultative Conference (collectively known as *Lianghui* in Chinese)	“Plead *Lianghui* delegates to pay attention to the problem of hepatitis B” Dear @[user]: Delegate, hello. I am LEI Chuang, Shanghai Jiaotong University graduate student, a hepatitis B virus carrier, who is a long-term observer of the problem of hepatitis B discrimination in China. Hope that you can submit to *Lianghui* the proposals #Further eliminate discrimination based on hepatitis B status# and #Full ban against hepatitis B advertisement#. For details, see http://t.cn/zObxzrO. If I can represent China’s 100 million hepatitis B virus carriers, I would like to say thanks to you.

### 1(b) News (cases)

News of cases of human infection of avian influenza, epidemic hemorrhagic fever (in the United Kingdom), epidemic encephalitis B, scarlet fever, malaria, echinococciosis and diarrhea also attracted microblog users’ attention (Table 2). One example was a severe malaria infection in a Chinese worker who had returned from Africa; this case attracted a lot of attention (Fig 2; Table 3), including newspaper coverage [31] (see S1 File for details).

### 2. Health education / information

Health promotion campaigns organized by the Chinese health authorities, such as World AIDS Day (Fig 2) and National Immunization Day for poliomyelitis could attract Weibo users’ attention. Weibo users could also share health information from other sources too, as in the case of hepatitis A, hepatitis E, measles, typhoid, pertussis, diphtheria, tetanus, scarlet fever, brucellosis, leptospirosis, mumps, rubella, conjunctivitis and filariasis (Table 2). One example is that the recommendation made by the Advisory Committee of Immunization Practice of the U.S. Centers for Disease Control and Prevention that all pregnant women should receive the Tdap 3-in-1 vaccine was circulated online by certain microbloggers in China (Fig 2; Table 3). Both peaks of posts about diphtheria and pertussis were online conversations about this recommendation. However, not necessary all health information came from formal sources. For example, many were circulated as health advice or information provided by certain popular websites or Weibo users. For example, the peaks for measles and mumps were about “Four diseases that have symptoms similar to the common cold” (see S1 File for details).

### 3. Alternative health information / Traditional Chinese Medicine

Alternative health information or knowledge of Traditional Chinese Medicine may attract Weibo users’ attention, as in the case of dysentery. The peak for “dysentery” was generated by a post (and its reposts) about “food that cannot be consumed together with chicken meat”, including *penis et testis canis*, a type of traditional Chinese medicine (Fig 2; Table 3). According to that post, consuming it together with chicken meat will lead to dysentery (see S1 File for details).

### 4. Commercial advertisement / entertainment

Commercial advertisements and the entertainment industry might also lead to an increase in Weibo posts that mentioned certain diseases, as in the case of plague, dengue, influenza and leprosy (Table 2). An example was an advertisement about holidays in Thailand. The travel agency gave customers mosquito repellent cream as a gift to prevent dengue (Fig 2; Table 3; see S1 File for details).

### 5. Social issues

Weibo users discussed various social issues, such as gender equality (gonorrhea and syphilis), discrimination against carriers of certain viruses (hepatitis B), the loss of trust between the government and the citizens (SARS), and the loss of trust between the medical profession and the patients (meningitis, schistosomiasis and hand-foot-and-mouth disease) (Fig 2; Table 2). One example was a petition to end discrimination against hepatitis B virus (HBV) carriers, addressed to the Chinese People’s Congress and the Chinese People’s Political Consultative Conference (Fig 2; Table 3). A civil rights advocate who was also an HBV carrier used Weibo to contact delegates and asked if they would submit the proposal on behalf of the China’s 100 million HBV carriers (see S1 File for details).

### 6. Others

News and online discussion that were not pertaining to infectious diseases might mention the infectious diseases (rabies and tuberculosis) for other reasons. An example was the so-called “Miami cannibal attack” in the United States leading to a peak on the keyword for rabies (Fig 2; Table 3; see S1 File for details).

## Discussion

We performed qualitative content analysis to investigate what news, events or information would trigger a reaction among Chinese social media users, leading to a peak in the daily count of Weibo posts containing keywords for a list of infectious diseases, notifiable in mainland China as of 2012. We identified 5 categories, namely, news of an outbreak or a case, health education / information, alternative health information (or Traditional Chinese Medicine), commercial advertisement / entertainment, and social issues.

Identification of these categories of triggers for Chinese microbloggers’ responses is important for digital health communications in two ways. First, digital epidemiologists use time series of search query data or social media data to predict trends of infectious diseases, such as influenza [5,32]. These prediction analyses rely on keywords or their combinations to identify the relevant search query data or Twitter tweets. The underlying assumption is that an increase in disease incidence would motivate people to search online for health information or to share their experiences online via social media. Content analysis of social media posts enables researchers to go beyond keyword to fully understand the contents of the posts and the contexts from which they were generated. Our analysis highlights the fact that social media trends of disease-specific keywords could reflect people’s online reaction to a variety of events and information, of which many are not related to any actual outbreak. Our study paves the way for future studies that can investigate the predictive value of similar news, events or information.

Second, public health agencies use social media to communicate messages of health promotion or disease prevention to the public [10,11,12,14]. Our analysis identified examples of health promotion events or messages that triggered people’s reactions (e.g. World AIDS Day, Fig 2), as well as examples of alternative health information. Our findings will enable researchers to further explore the most effective methods to communicate health information.

In addition, social media content analysis can provide public health researchers an additional means to reveal health-related social issues. For example, concurring with previous reports [33], our content analysis revealed that tension between the medical and public health professions and the general public is often a topic of heated discussion on social media in China. Examples such as the news of an alleged cover-up of a hepatitis C outbreak or the conflict associated with testing for sexually transmitted diseases, leading to the peaks of microblog posts that we observed, revealed a mistrust of government institutions and the medical profession at large. The routine censorship of social media content did not help to build trust, either [18]. Transparency in disease control efforts and outbreak data management should be part and parcel of an effective public health communications strategy.

### Strength and limitations

Our study is the first to categorize Chinese microblog contents on 42 infectious diseases and also identify the related news and/or information. While another study compared Chinese and US social media use in health communication at the onset of the H1N1 pandemic, no Weibo data were analyzed therein [34].

In our study, we manually coded Chinese microblog posts to obtain a better understanding of the actual contents. While there are other studies that used computerized machine learning methods [35,36], or keyword search to analyze health-related Twitter contents [37], there are others that manually coded Twitter data on: childhood obesity [38], influenza A(H1N1) [39], and antibiotics [40].

The Chinese microblogger community is a fraction of the 1.3 billion people living in mainland China. According to a random sampling study, Weibo users are more likely to be male and living in provinces/regions that are more economically developed (with some exceptions) [15]. Compared to the younger generation, the older Chinese are in general less likely to use the internet to seek health information [41]. Furthermore, our dataset is comprised of the 350,000 users who had 1,000 followers or more. Of these 350,000 users, 5000 were Chinese dissident writers, journalists and scholars, and another 38,000 were users with an authenticated (also known as VIP) status with more than 10,000 followers, as discussed in the original study [18]. While our sample constituted less than 1% of all registered users of Sina Weibo, this sampling strategy allowed us to avoid many spam accounts [15]. The Weibo universe is highly heterogeneous. A random sampling study found that over 50% of Sina Weibo users have never posted anything, whereas 80% of the original posts were created by 5% of the users [15]. Hence, our sample represents the most influential Chinese microbloggers who contributed a majority of Weibo contents and drew the most attention as far as reposts and comments were concerned [15]. Nonetheless, our findings may not be generalizable to samples obtained through other sampling strategies. Our operational sampling parameters were not determined to optimize data collection specific to any diseases. Future research design that is customized for specific epidemiologic research may help reconfirm our research findings.

In the absence of content analysis of Weibo posts for each disease keyword at the baseline, and given the diversity of information that triggered the elevated Weibo activities, we did not attempt to compare across the categories of information that triggered the elevated Weibo activities. Future research along that direction is warranted.

Our analysis is only a small step towards a better understanding of the Chinese online community’s understanding and communications about health issues. Many questions remained unresolved. For example, how can we predict users’ reactions to different types of news, events or information? What, if any, behavioral changes will health information on social media bring about? What is the relationship between healthcare-seeking behavior and tweeting behavior? Our study provides the ground work for future research that will shed light upon these issues.

## Conclusions

Infectious disease-related information triggered Chinese social media users’ reactions to create and repost microblog posts. Our content analysis categorized them into 5 categories, namely, news of an outbreak or a case, health education / information, alternative health information (or Traditional Chinese Medicine), commercial advertisement / entertainment, and social issues. Our study opens the door towards a better understanding of online health information diffusion and health information seeking behavior that will better equip digital epidemiologists to fine-tune their prediction and detection tools, and will enable health communicators to fine-tune their social media messages.

The identification of news and information pertaining to infectious diseases that trigger an elevated microblog activity and the contents thereof has utility and significance in health communications and disease surveillance. Our study covers 42 infectious diseases. We demonstrated how information and issues about these diseases drew social media users’ attention and led to increased social media traffic. Such finding, in turn, will pave the way for future studies about online health communications. This study contributes to the literature by analyzing data from Sina Weibo, the leading microblogging platforms in China, where Twitter is blocked. We analyze the reactions of a sample of influential users in a national online community to health-related news, information and social issues. This helps facilitate better social media health communication and may shed light on the “false positives” in digital disease detection. The lessons learned in our study can be transferrable to other middle income countries.

## Supporting Information

S1 File Supplemental Online Appendix. (PDF)
