# Correction: Chinese Social Media Reaction to Information about 42 Notifiable Infectious Diseases

**DOI:** 10.1371/journal.pone.0129525

**Published:** 2015-05-20

**Authors:** The PLOS ONE Staff

There are a number of errors in Table 1. Please see the corrected Table 1 here. The publisher apologizes for the errors.

**Table 1:** List of infectious diseases notifiable in mainland China according to the Law of the People’s Republic of China on the Prevention and Treatment of Infectious Diseases (as of 2012), and the corresponding keywords used to search Weibo posts.

Simplified Chinese	English translation*	Chinese keyword used to search Weibo posts	Notes / References
**甲类传染病**	**Class A**		
1. 鼠疫	Plague	鼠疫	
2. 霍乱	Cholera	霍乱	
**乙类传染病**	**Class B**		
3. 传染性非典型肺炎	Severe Acute Respiratory Syndrome	非典	Please refer to Fung et al. [22] for more details.
4. 艾滋病	HIV/AIDS	艾滋	
病毒性肝炎	Viral hepatitis		Viral hepatitis was listed as a single disease in the law. The Chinese Center for Disease Control and Prevention reports the total number of viral hepatitis per month, and its sub-categories: A, B, C, E and un-typed. We decided to study Weibo content about hepatitis A, B, C and E only.
5. 甲型肝炎	Hepatitis A	甲肝	
6. 乙型肝炎	Hepatitis B	乙肝	
7. 丙型肝炎	Hepatitis C	丙肝	
8. 戊型肝炎	Hepatitis E	戊肝	
9. 脊髓灰质炎	Poliomyelitis	脊髓灰质炎	
10. 人感染高致病性禽流感	Human infections of highly pathogenic avian influenza	禽流感	
11. 甲型H1N1流感†	Influenza A(H1N1)	H1N1	Listed as a separate Category B entity from 2009 to 2013.
12. 麻疹	Measles	麻疹	
13. 流行性出血热	Epidemic hemorrhagic fever	出血热	
14. 狂犬病	Rabies	狂犬病	
15. 流行性乙型脑炎	Epidemic Encephalitis B	流行性乙型脑炎, 乙脑, 日本脑炎	We searched three terms that refer to the same disease. They are流行性乙型脑炎 (Epidemic Encephalitis B), 乙脑 (short form for Epidemic Encephalitis B) and 日本脑炎 (Japanese encephalitis).
16. 登革热	Dengue	登革热	
17. 炭疽	Anthrax	炭疽	
18. 细菌性和阿米巴性痢疾	Bacterial and amoebic dysentery	痢疾	
19. 肺结核	Tuberculosis	肺结核	
20. 伤寒和副伤寒	Typhoid and paratyphoid	伤寒	Our keyword search did not distinguish typhoid and paratyphoid.
21. 流行性脑脊髓膜炎	Epidemic (meningococcal) meningitis	脑膜炎, 脑脊髓膜炎,流脑	We searched three terms that refer to the same disease. They are 脑膜炎(meningitis), 脑脊髓膜炎(cerebrospinal meningitis) and流脑(short form for epidemic meningitis).
22. 百日咳	Pertussis	百日咳	
23. 白喉	Diphtheria	白喉	
24. 新生儿破伤风	Neonatal tetanus	破伤风	
25. 猩红热	Scarlet fever	猩红热	
26. 布鲁氏菌病	Brucellosis	布鲁氏菌病	
27. 淋病	Gonorrhea	淋病	
28. 梅毒	Syphilis	梅毒	
29. 钩端螺旋体病	Leptospirosis	钩端螺旋体病	
30. 血吸虫病	Schistosomiasis	血吸虫病	
31. 疟疾	Malaria	疟疾	
**丙类传染病**	**Class C**		
32. 流行性感冒	Influenza	流行性感冒, 流感	We use both the full term (流行性感冒) and its short form (流感) for keyword search.
33. 流行性腮腺炎	Mumps	腮腺炎	
34. 风疹	Rubella	风疹	
35. 急性出血性结膜炎	Acute hemorrhagic conjunctivitis	结膜炎	
36. 麻风病	Leprosy	麻风	
37. 斑疹伤寒	Typhus	斑疹伤寒	
38. 黑热病	Leishmaniasis	黑热病	
39. 包虫病	Echinococciosis	包虫病	
40. 丝虫病	Filariasis	丝虫病	
41. 其它感染性腹泻病	Other infectious diarrheal diseases	腹泻	
42.手足口病	Hand, foot and mouth disease	手足口病	

*Class A and B diseases require immediate notification to health authorities of a higher administrative level; Class C diseases require periodical notification. Class A refers to diseases highlighted in the International Health Regulations [28] that may constitute a public health emergency of international concern, of which cholera and plague (listed in the law) are (or were) endemic in parts of China. The law specified that outbreaks of class A diseases require prompt infection control measures including immediate isolation of patients and quarantine (medical observation) of their contacts. Collection, transportation and testing specimens of Class A pathogens require approval from the provincial authorities or the central government [27].

^†^ Influenza A(H1N1) was classified as a Class B infectious disease and was added to the list as a separate entity during the 2009 pandemic [29]. On October 28, 2013, it was announced by the Chinese government that starting from January 1, 2014, influenza A(H1N1) would cease to be listed separately and would be grouped with other influenza strains as an aggregated number listed in Class C [30].
