# Supplementary material for: Chinese Social Media Reaction to Information about 42 Notifiable Infectious Diseases
Source: PLoS One. 2015 May 6;10(5):e0126092. doi: 10.1371/journal.pone.0126092 (PMC4422708; doi:10.1371/journal.pone.0126092)
Supplement: S1 File — (PDF) [file pone.0126092.s001.pdf]

Chinese social media reaction to information about 42 notifiable infectious diseases

Supplemental Online Appendix

Isaac Chun-Hai Fung[1*], Yi Hao[2¶], Jingxian Cai[2¶], Yuchen Ying[3], Braydon James Schaible[2], Cynthia Mengxi Yu[4,5], Zion Tsz-Ho Tse[6&], King-Wa Fu[4&]

[1] Department of Epidemiology, Jiann-Ping Hsu College of Public Health, Georgia Southern University, Statesboro, Georgia 30460-8015, USA.

[2] Department of Biostatistics, Jiann-Ping Hsu College of Public Health, Georgia Southern University, Statesboro, Georgia 30460-8015, USA.

[3] Department of Computer Science, The University of Georgia, Athens, Georgia 30605, USA.

[4] Journalism and Media Studies Centre, The University of Hong Kong, Hong Kong;

[5] School of Journalism and Communication, Peking University, Beijing 100871, China.

[6] College of Engineering, The University of Georgia, Athens, Georgia 30602, USA.

* Corresponding author

Email: cfung@georgiasouthern.edu;

[¶] These authors contribute equally to this work

[&] These authors also contribute equally to this work

Table of contents

Introduction to the Supplemental Online Appendix 4
1. Plague 5
2. Cholera 7
3. SARS 11
4. HIV/AIDS 14
5. Hepatitis A 33
6. Hepatitis B 36
7. Hepatitis C 39
8. Hepatitis E 44
9. Poliomyelitis 47
10. Avian influenza 51
11. Influenza A(H1N1) 56
12. Measles 59
13. Epidemic hemorrhagic fever 62
14. Rabies 65
15. Epidemic encephalitis B 69
16. Dengue 72
17. Anthrax 74
18. Bacterial and amoebic dysentery 77
19. Tuberculosis 79
20. Typhoid or paratyphoid 81
21. Epidemic meningitis 85
22. Pertussis 92
23. Diphtheria 95
24. Tetanus 98
25. Scarlet Fever 101
26. Brucellosis 104
27. Gonorrhea 106
28. Syphilis 109
29. Leptospirosis 112
30. Schistosomiasis 114
31. Malaria 116
32. Influenza 119
33. Mumps 130

34. Rubella..132
35. Conjunctivitis 136
36. Leprosy..139
37. Typhus...142
38. Leishmaniasis 145
39. Echinococciosis 148
40. Filariasis 150
41. Diarrhea 153
42. Hand-foot-and-mouth disease...168
References 170

Introduction to the Supplemental Online Appendix

The Supplemental Online Appendix provides the results of the qualitative content analysis of Weibo posts containing the keyword for each infectious disease included in our paper. Each section follows the same format: first, a figure of the time series of the daily count of Weibo posts that contained the specified keyword; second, the date of the peak for which we performed the content analysis; third, sample Weibo posts that were representative of the posts of the peak, as well as the count and categories of Weibo posts that they represented. The order in which the diseases are presented follows the list of the *Law of the People's Republic of China on Prevention and Treatment of Infectious Diseases* (中华人民共和国传染病防治法), as of 2012 [1]. For an official English translation of the Law, see reference [2]. Viral hepatitis is counted as one disease in the law, but we counted Hepatitis A, B, C and E separately following the disease reporting scheme of the Chinese Center for Disease Control and Prevention.

Notes:

The user IDs of Weibo users in our database had been anonymized and replaced by random numbers. If they appeared in the Weibo posts, they are either deleted from the presentation below, or they are printed as @[user]. Sometimes, @[user1], @[user2] etc., are used to distinguish one user from another in the same Weibo post (for example, in a repost of a repost of a Weibo post).

1. Plague

Figure S1. Daily count of Weibo posts with the keyword for “Plague” (鼠疫) in 2012 among Sina Weibo users with 1000 followers or more

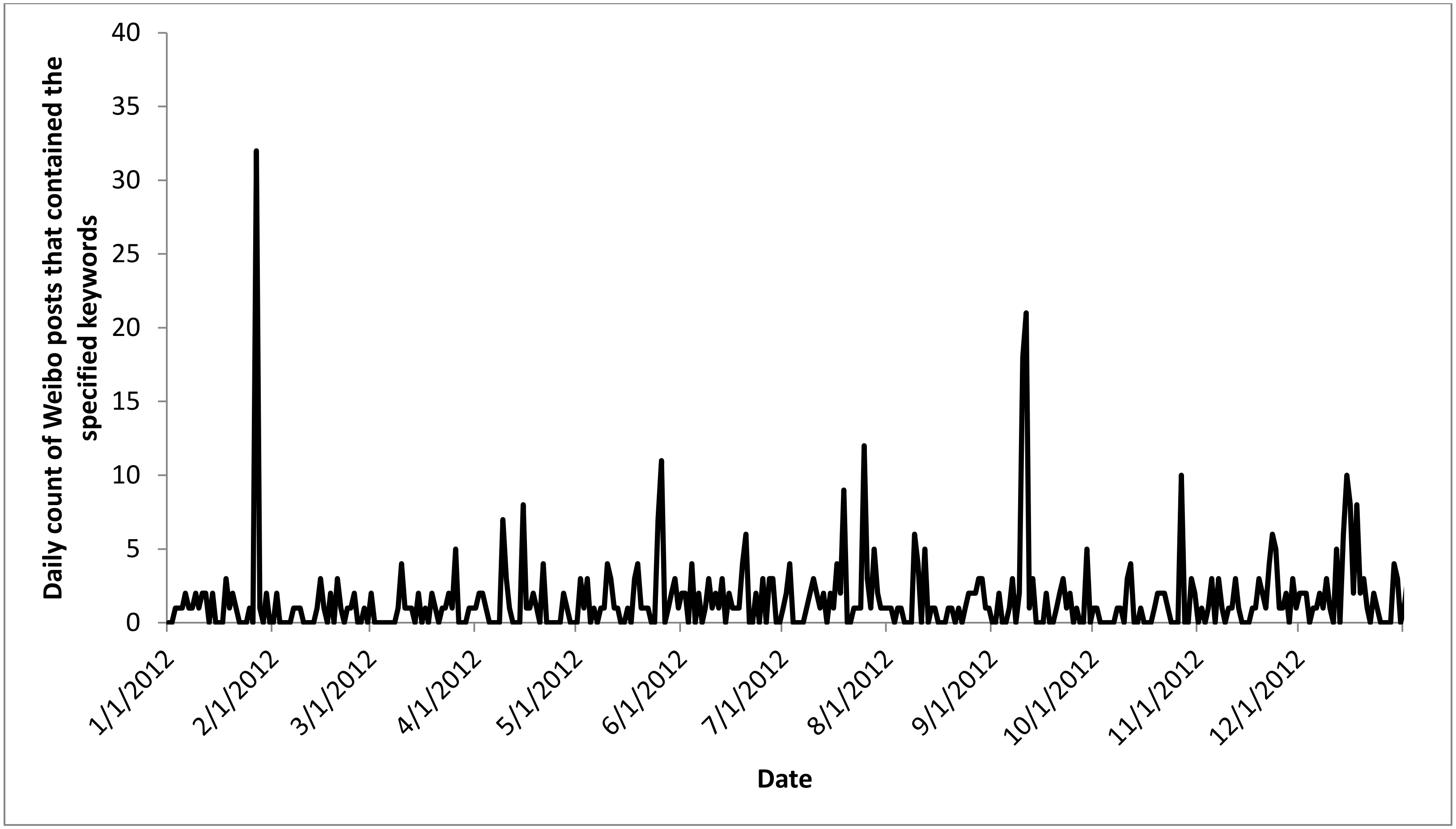

Content analysis was performed for Weibo posts generated on the peak date of January 27, 2012.

Peak Date: January 27, 2012

Total count: 32

Summary: There were 32 Weibo posts that mentioned “plague” on January 27, 2012. Of them, 31 posts had nothing to do with the real disease. “Plague” was used as an analogy to negative comments about Kim Hee-Chul (金希澈, 김희철), a member of the Korean pop group Super Junior, spread by those Weibo users who disliked him. The Chinese fans club of that Korean pop group was telling Kim’s fans not to argue with them, not to re-post their posts, and to delete their posts, so that their opponents would not gain any benefit (i.e. high re-post rate) from them.

The other post involved someone’s family history: his father got rid of plague in Beijing in 1949.

Table S1. Sample Weibo entries that contained the keyword for “Plague” (鼠疫) on January 27, 2012.

Sample Weibo posts on that day	English translation / description	Count (%)	Sources / References / Notes	Category
不要给那个阴沟里的耗子传播鼠疫的机会~我们需要干净的空气和环境~有希美人养眼就好了~大过年的别没事找气生~// @[user] 大扫除一下~~首页还是干净点的好是不咯	Do not give the rat in the sewer a chance to spread the plague. ~ We need clean air and environment. ~ To have the handsome Xi [i.e. Kim Hee-Chul, a member of the Korean pop group Super Junior] to look at is a feast for the eyes. ~ At the Chinese New Year, create trouble for yourself by finding things to get angry. //@[user] To clean the house~~ The Homepage looks better if it is cleaner, doesn’t it?	31 (97%)	Original Weibo post Reference: http://www.weibo.com/1567054654/y2LIYwOOq<br><br>Fans club notice (long Weibo post, January 27, 2012): http://www.weibo.com/1779682345/y2IVK2GQe	Personal comments (Pop culture)
1949 年 1 月北平解放，2 月 4 日，父亲被任命为第 7 区区长，辖区正在市中心，包括天安门广场、东西长安街、东西交民巷等，许多重要机关也在这里。区委会发动群众，建立政权，收缴枪支，打击黑市，清除垃圾、整顿摊贩，组织生产，消灭鼠疫，使原来脏乱不堪，百业凋敝的辖区迅速改观，保证了开国大典顺利举行。	Beijing was liberated in January 1949. On February 4, my father was appointed as the Head of the 7th district. The jurisdiction was city center, including the Tiananmen Square, East & West Chang’an Street, East & West Jiaominxiang [Legation quarter] etc., many important institutions were also there. The district committee motivated the people; established the political authority; confiscated firearms; combated the black market; removed rubbish; tidied up the street vendors; organized production; got rid of plague. They rapidly improved the dirty and chaotic district (with languished businesses) and ensured that the Founding ceremony [of the People’s Republic of China] would be successfully held.	1 (3%)	Original blog post: http://blog.renren.com/share/246107183/11357677201	Quotation from someone’s blog post that mentioned family history

2. Cholera

Figure S2. Daily count of Weibo posts with the keyword for "Cholera" (霍乱) in 2012 among Sina Weibo users with 1000 followers or more.

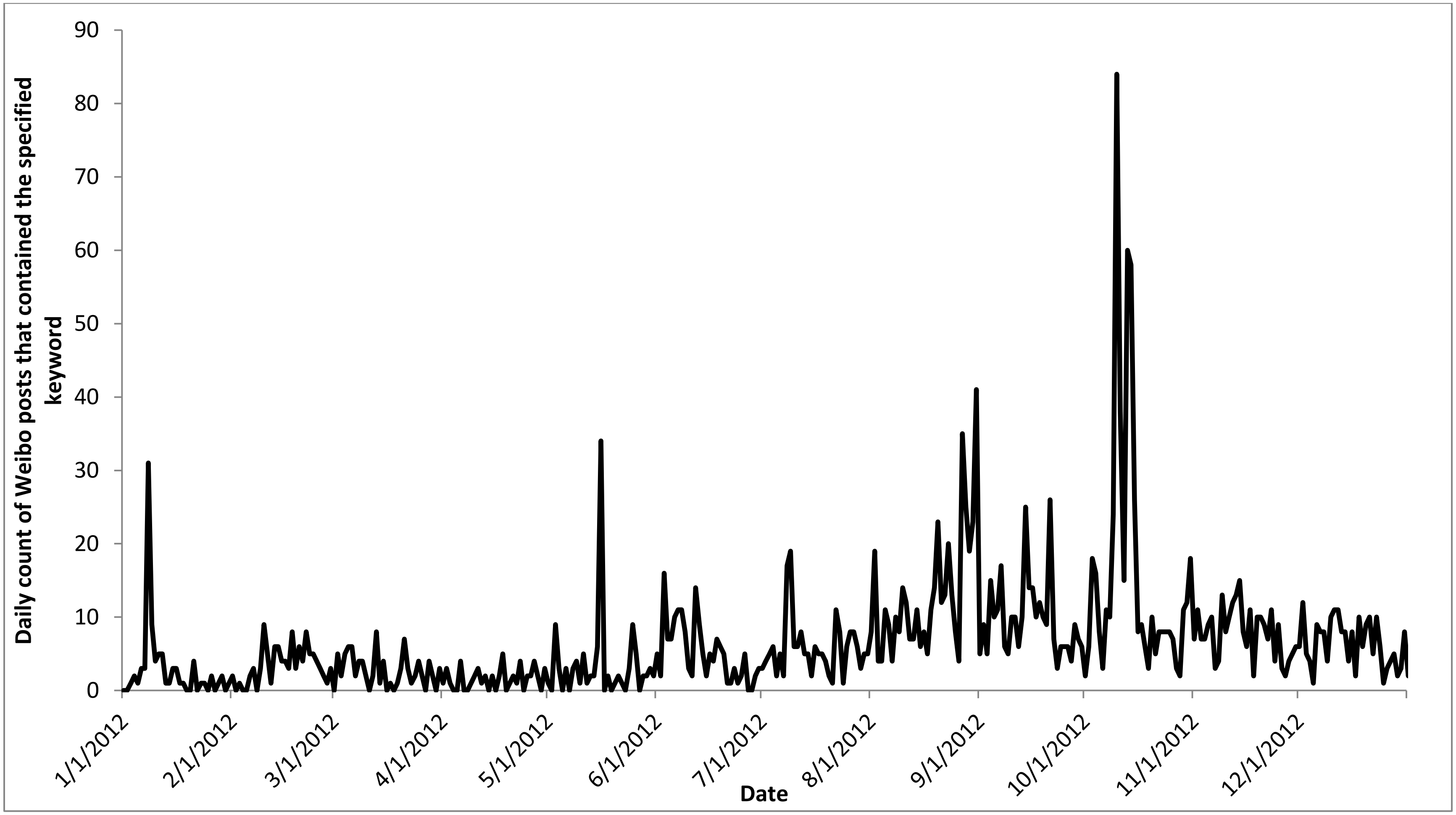

Content analysis was performed for Weibo posts generated on the peak date of October 10, 2012.

Peak Date: October 10, 2012

Total count: 84

Summary: Of the 84 Weibo posts that mentioned "cholera" on October 10, 2012, 73 were related to a cholera outbreak in Huangshi, Hubei province. Of these 73 posts, the majority (n=64) were news reports and re-posts thereof. Nine posts were health education, advising people to avoid food vendors of low hygienic standard and to practice good personal hygiene, including "eating cooked food, drinking boiled water, washing hands".

Other Weibo posts that mentioned "cholera" include 3 posts that described the challenges of development and cholera prevention in Haiti; 2 posts about García Márquez's *Love in the Time of Cholera*; and 6 about Mr. Zhu Rongji, the former premier of China.

Table S2. Sample Weibo entries that contain the keyword for "cholera" (霍乱) on October 10, 2012.

Sample Weibo posts on that day	English translation / description	Count (%)	Sources / References / Notes	Category
**Cholera outbreak in Huangshi, Hubei**		73 (87%)		
#霍乱#【湖北黄石发生一起霍乱疫情 确诊病例 3 例】湖北省近 3 个月内发生的第 3 起霍乱疫情。经查，疫情所涉患者在某酒店聚餐，酒店餐饮加工人员有一带菌者，且负责加工凉菜。	#Cholera# "An outbreak of cholera takes place in Huangshi, Hubei; 3 confirmed cases" This is the 3rd cholera outbreak in Hubei province in the recent 3 months. The outbreak investigation reveals that patients had meals in a hotel. Among the catering staff at the hotel, one was a carrier who was responsible for processing of the cold dish.	64 (76%)	http://www.chinanews.com/fz/2012/09-25/4208934.shtml	News related to Cholera outbreak
//@[user1]贪嘴有风险，凉菜请小心！ //@[user2] #霍乱#【提醒】对卫生条件不达标的餐饮单位和小食摊点上的凉菜、卤菜和生冷食品最好别买。回家自己做更安全！ //@[user3]又是凉菜！！！ 大家小心了！（2010 年安徽蒙城报告霍乱病例 33 例，全县禁凉菜冷食供应。）	//@[user1] Being gluttonous is risky; beware of cold dish //@[user2] #Cholera# Beware. It is better not to buy cold dish, dish made with master stock (Chinese: 卤水), and cold food from restaurants and food vendors whose hygienic conditions are not up to standard. It is safer to go home and make them yourself! //@[user3] Again, cold dish!!! Beware, everyone! (In 2010, after receiving reports of 33 cholera cases in Meng City, Anhui, the whole county bans the provision of cold dishes.)	5 (6%)	Original Weibo post	Health education; personal comments on the cholera outbreak
霍乱是常见传染病，可防可治。据专家分析，本次发病的大部分病例与进食不洁食物有关。专家提醒市民，注意保持个人卫生，做到"吃熟	Cholera is a common infectious disease that can be prevented and treated. According to expert analysis, the majority of the cases in this outbreak are linked to eating unclean food. Experts remind citizens: if you	4 (5%)	http://news.qq.com/a/20050916/001476.htm	Health education

食、喝开水、洗净手”，就不会感染霍乱病。	pay attention to maintain personal hygiene and practice “eating cooked food, drinking boiled water, washing hands”, you will not be infected with cholera.			
**Haiti, its reconstruction after the earthquake and its cholera prevention efforts.**		3 (4%)		
#维和影像#【海地，别哭】海地强震发生两年多后，灾区仍有近 50 万人在临时搭建的庇护帐篷营地居住。作为世界上最不发达国家之一，该国失业率仍超过 60%。持续减少的人道主义救援资金对海地重建进程造成了严重威胁，尤其体现在霍乱病的防治方面。刚刚又遭受热带风暴袭击，海地经济社会发展更是雪上加霜。	#Peace-keeping image# “Haiti, don’t cry” Since the Haitian strong earthquake happened more than two years ago, there are still about 500,000 people living in tents in temporary settlements. Among one of the least developed countries, Haiti’s unemployment rate is over 60%. The continual decrease of humanitarian aid is a severe threat to the reconstruction process in Haiti, especially in the prevention and treatment of cholera. The recent hurricane has made the economic and societal development of Haiti even worse.	3 (4%)	Original Weibo post	About Haiti
**Gabriel García Márquez’s *Love in the Time of Cholera***		2 (2%)		
#图书排行榜#大众书局 9 月份销售排行榜，《霍乱时期的爱情》依然榜上有名，在五十年的时间跨度中，马尔克斯展示了所有爱情的可能性，所有的爱情方式：幸福的爱情，贫穷的爱情，高尚的爱情，庸俗的爱情，粗暴的爱情……甚至，“连霍乱本身也是一种爱情病”。	#Book ranking# *Love in the Time of Cholera* is still listed in the book sales charts of People’s Bookshop in September. Over a time span of 50 years, Márquez demonstrated all possibilities of love, all ways of love: blissful, poor, noble, vulgar, rude… even “cholera itself is a disease of love”.	2 (2%)	http://weibo.10086.cn/feed/single.php?type=reply&feedid=110318071054613	About the novel *Love in the Time of Cholera*
**Zhu Rongji, former premier of China**		6 (7%)		
在中国，有这样一个人：一个孤儿，未出生，父早逝，九岁，母病死。命运多舛，湘西求学，染上霍乱，几乎死去。清华有名的高材生。他带领中国顺利度过亚洲金融危机，面对九江大堤豆腐渣工程勃然大怒，看到抗洪士兵怆然泪下。他还准备 100 口棺材，99 口留给贪	In China, there was such a man: an orphan, whose father died before he was born, and his mother died when he was nine. Ill-fated, when he attended school in western Hunan, he nearly died of cholera. He was a famous high achiever in Tsinghua University. He led China to overcome the Asian Financial Crisis smoothly. He was infuriated because of the corrupt and poor engineering work at Jiujiang Dyke. He weeped when he saw the soliders who fought the	6 (7%)	http://m.3g.qq.com/wbread/copage/singlemsg?msgid=273758006953866	About former Chinese premier Zhu Rongji

官，一口留给自己。他就是一代名相——朱镕基。	flood. He also prepared 100 coffins, of which 99 were reserved for the corrupt government officials and one for himself. He is the famous premier – Zhu Rongji.			

3. SARS

Figure S3. Daily count of Weibo posts with the keyword "Fei-dian" (非典), i.e. SARS, in 2012 among Sina Weibo users with 1000 followers or more.

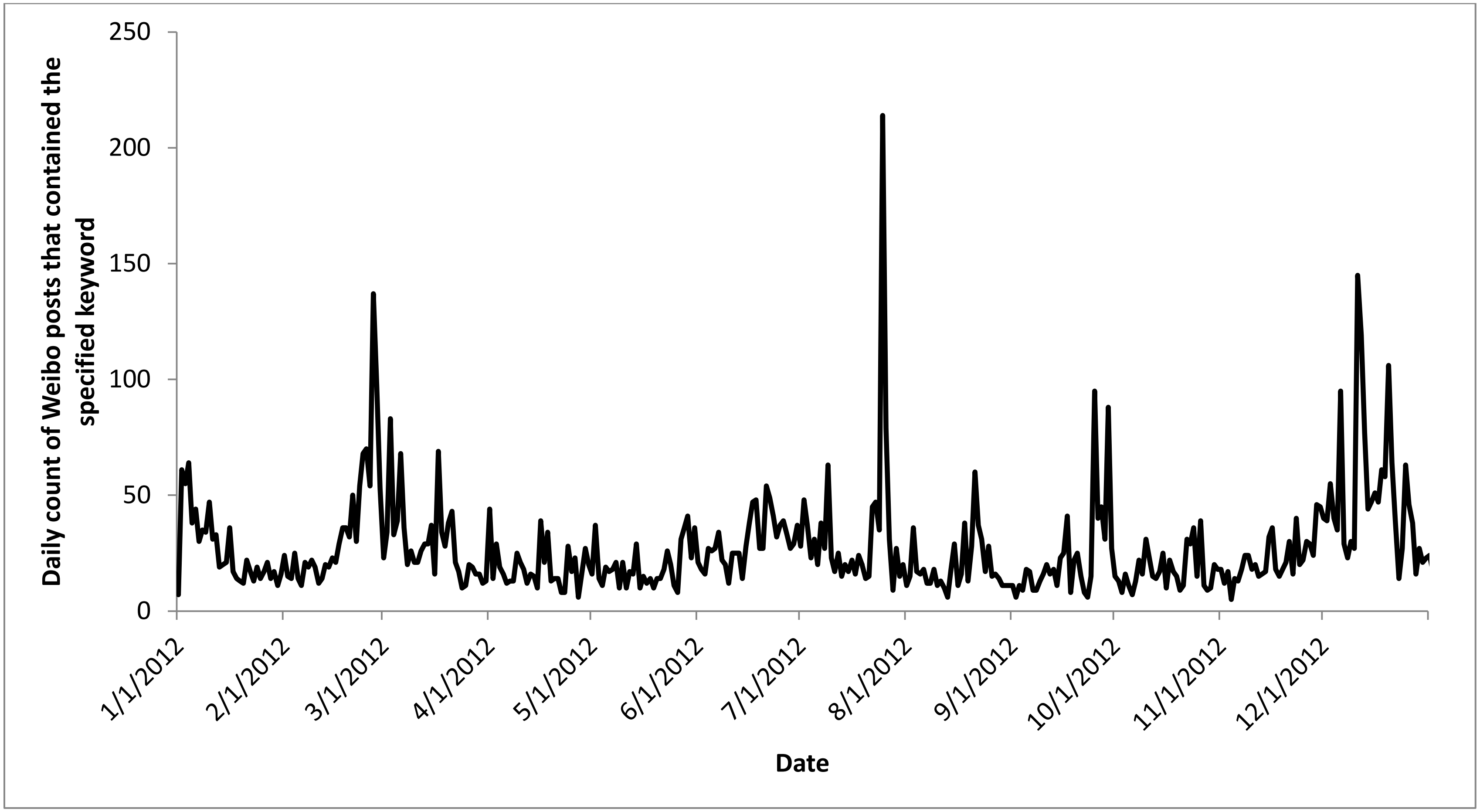

Content analysis was performed for Weibo posts generated on the peak date of July 25, 2012.

Peak Date: July 25, 2012

Total count: 214

Summary: Of the 214 Weibo posts about "Fei-dian" (非典), i.e. SARS, on July 25, 2012, 169 posts were about the rainstorm and flooding in Beijing that led to many injuries and deaths on July 21, 2012. As many people suspected that the government was covering up the actual number of victims of the floods, the Beijing Municipal Government held a press conference on July 24 and declared that they would release the official data as quickly as possible. The director of the press office of the Beijing Municipal Government also said that having experienced the challenge of SARS ("Fei-dian"), they would never cover up the case count of injury and death again.

Another 41 posts could be classified as personal comments or recollection of personal experiences. Weibo users recollected their own experiences of disasters including SARS in 2003.

Table S3. Sample Weibo entries that contained the keyword "Fei-dian" (非典), i.e. SARS, on July 25, 2012.

Sample Weibo posts on that day	English translation / description	Count (%)	Sources / References / Notes	Category
**Rainstorm and flooding in Beijing led to many deaths**		**169 (79%)**		**Social issue**
欲语还羞！//@[user]【北京新闻办：经历过非典不会再隐瞒伤亡数字】7 月 24 日，针对民众对暴雨伤亡数字的质疑，北京新闻办主任王惠称，正在辨识新发现的遗体，会尽快公布，并表示北京市政府经过 SARS 的考验，不会再隐瞒伤亡数字。(新民网)	Too embarrassed to say anything even if he wants to speak up! //@[user] "Beijing Municipal Press Office: Having experienced SARS, we would not cover up the case count of injury and death" On July 24, in response to people's suspicion of the case count of injury and death caused by the rain storm, the Director of the Press Office of the Beijing Municipal Press Office, Wang hui, said that they were identifying the corpses and would release information as soon as possible. Wang also mentioned that having experienced the challenge of SARS, Beijing Municipal Government would never cover up the case count of injury and death again.	137 (64%)	http://news.qq.com/a/20120725/000097.htm	News related to flooding in Beijing (the press conference of Beijing Municipal Government press office)
2003 年非典期间，因为瞒报疫情和人数，北京市长孟学农和卫生部长张文康被免职。瞒报是有传统的。	During the SARS epidemic in 2003, the major of Beijing, Meng Xuenong, and the minister of health, Zhang Wenkang, were dismissed because they covered up the epidemic and the case count. There is a tradition of cover-up.	32 (15%)	An example of a similar post: http://weibo.com/2256351864/yu6YLsfXp	Personal comments
**Recollection of memory / reminiscence (SARS as personal experience)**		**41 (19%)**		

活了二十年，碰上 50 年一遇的旱灾，60 年一遇的雪灾，100 年一遇的洪涝，300 年一遇的泥石流，500 年一遇的地震，600 年一遇的海啸，还有什么日食月食流星…迎着非典上小学，踏着禽流感上初中，冒着甲流上高中，现在上大学了，又说 2012 世界未日！我们这辈子也算活得轰轰烈烈了... 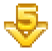	I have lived 20 years, having experienced a drought that was 1 in 50 years, a snow storm that was 1 in 60 years, a flood that was 1 in 100 years, a mudslide that was 1 in 300 years, an earthquake that was 1 in 500 years, a tsunami that was 1 in 600 years, and also solar eclipses, lunar eclipses and meteors…. Facing SARS, I attended primary school, stepping on avian flu, I attended lower middle school; taking the risk of influenza A, I attended upper middle school; now I attend university, someone said that 2012 is the end of the world! What a spectacular lives we have… 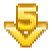	41 (19%)	http://tieba.baidu.com/p/1823489513	Personal comments
**Posts that were not related to SARS**		**4 (2%)**	Original Weibo post *	

*Note: Our keyword for SARS was “Fei-dian”, which is the short form of Fei-dian-xing Fei-yan, atypical pneumonia. As we did not perform tokenization process of the Chinese Weibo text, our search algorithm returned any posts that contained the term 非典型 “Fei-dian-xing” (atypical).

4. HIV/AIDS

Figure S4. Daily count of Weibo posts that contained the keyword for HIV/AIDS (艾滋) in 2012 among Sina Weibo users with 1000 followers or more.

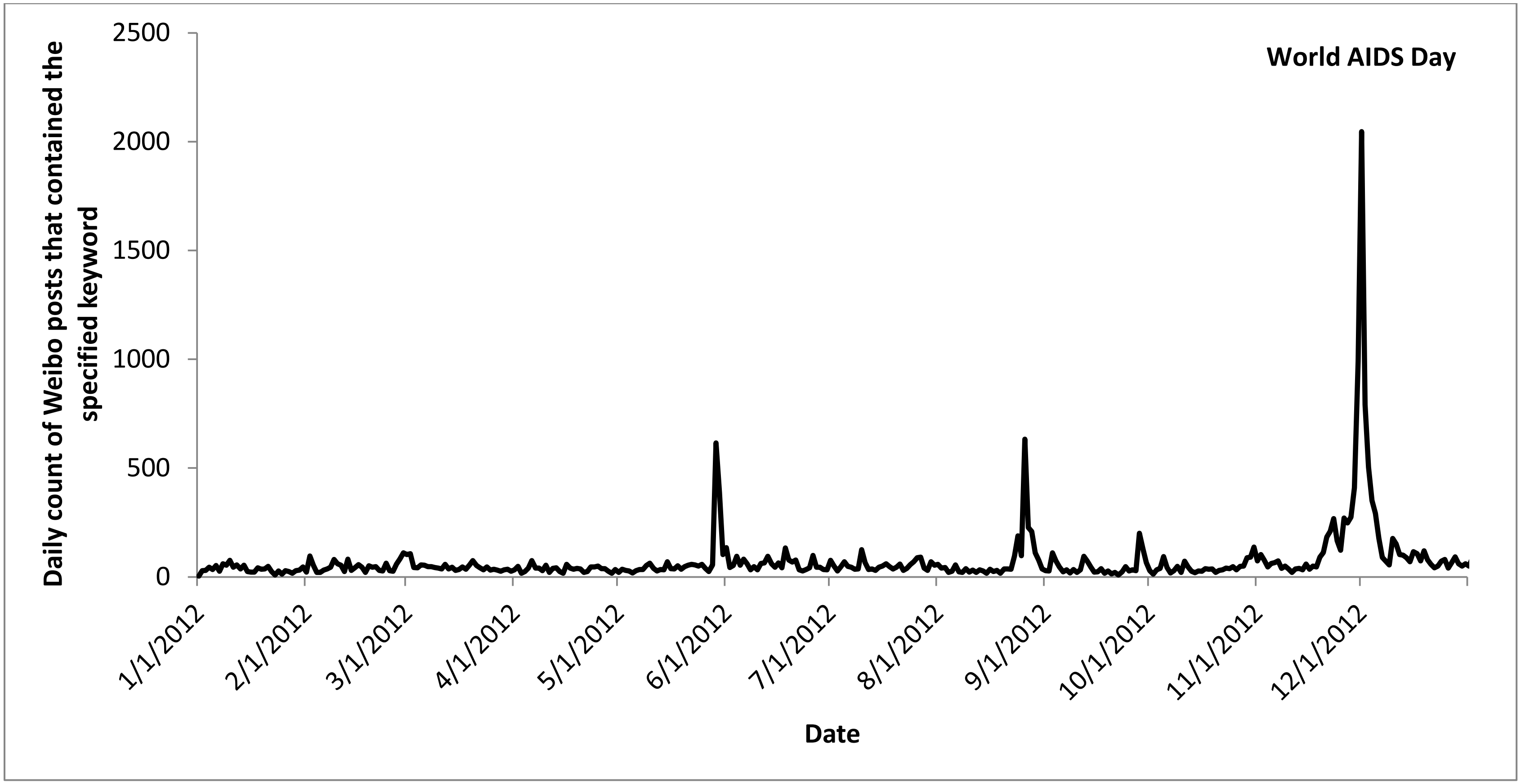

Content analysis was performed for Weibo posts that were generated on the peak date of December 1, 2012.

Peak Date: December 1, 2012
Total count: 2046

Summary: December 1, 2012 was the 25th World AIDS Day. Both the Chinese government and its media outlets were active advocates of HIV/AIDS prevention and the elimination of discrimination against HIV/AIDS patients. Many famous individuals took part in HIV education activities.

Of the 2046 Weibo posts generated on December 1, 2012, that mentioned the keyword for "HIV/AIDS" (艾滋), there were posts that mentioned individual HIV patients that were part of media news reports on World AIDS Day, including an HIV-positive woman and her husband (n=31), an HIV-positive man inviting people to drink tea with him (n=28), and an HIV-positive homosexual man afraid to meet his parents (n=19). There were also some news highlight posts that mentioned World AIDS Day.

The majority of the posts were related to health education/information, for example, introducing World AIDS Day (n=474); carrying the slogan for "Striving towards zero AIDS" (n=85); educating the public on the origin of World AIDS Day (n=55); advocating the removal of discrimination against HIV/AIDS patients (n=53); providing information related to HIV transmission among men who have sex with men (n=50); describing the routes of HIV transmission (n=48); mentioning World AIDS Day as "Today in history" (n=33); explaining what AIDS is (n=23); and explaining that the proper use of condoms is an effective means to prevent HIV transmission (n=22). Weibo posts are also means to promote other health promotion events, for example, an interactive health education activity (n=17); and a health promotion activity that gives away condoms (n=17).

We also identified posts that describe HIV/AIDS statistics, including UN global estimates (n=22), China's national estimates on male homosexual transmission (n=50), and national HIV estimates (n=15). There were also posts that provided statistics from various provinces and municipalities, including Zhejiang Province (n=23), Henan Provine (n=22), Hunan Province (n=4), Shanghai Municipality (n=3) and Chongqing Municipality (n=2), and posts that provided statistics from certain cities, including Shenzhen (n=5), Dongguan (n=4), Zhuhai (n=3) and Foshan (n=3) of Guangdong Province, and Suzhou city (n=4) of Jiangsu Province.

The involvement of top politicians in HIV/AIDS prevention activities attracted a lot of attention. These included the visit of HIV/AIDS patients on the eve of World AIDS Day 2012 by Xi Jinping (then General Secretary of the Central Committee of the Communist Party of China, and later in March 2013 also the President of the People's Republic of China) (n=94), the public service advertising / short movies on World AIDS Day that featured Peng Liyuan, the wife of Xi Jinping as well as WHO Goodwill Ambassador for Tuberculosis and HIV/AIDS (n=21) (reference: http://www.un.org/apps/news/story.asp?NewsID=38609#.U8Q4iPldVQF; June 3, 2011; accessed July 14, 2014) and a comment made by Premier Wen Jiabao (n=9).

Some Weibo posts discussed HIV-related social issues. For example, some remembered the renowned gynaecologist and AIDS activist Dr. Gao Yaojie who was forced to leave China for the United States in 2009 (n=32); some discussed the discrimination faced by HIV patients when they look for employment opportunities (n=25).

Based on our data, it was apparent that World AIDS Day 2012 successfully drew the attention of Chinese netizens, as represented by our dataset of users who had 1000 or more followers. Furthermore, World AIDS Day 2012 was a health promotion campaign that encompassed many events and activities across China.

Our data demonstrated that there was no single event or news article or Weibo post (and its reposts) that dominated the entire Weibo conversation about World AIDS Day 2012. There was a diversity of Weibo contents about HIV/AIDS on World AIDS Day 2012.

Table S4. Sample Weibo entries that contained the keyword for "HIV/AIDS" (艾滋) on Dec 1, 2012

Sample Weibo posts on that day	English translation / description	Count (%)	Sources / References / Notes	Category
**News of individual HIV patients**		**80 (3.9%)**		
【女子与多人一夜情后染艾滋 丈夫称将守其一生】她为情所伤，流连于夜店，终染 HIV。健康男子与其结婚，婚后万幸产下一健康孩子，后又多次怀孕，严重伤害了身体。12 月 1 日，世界艾滋病日，关注艾滋病。（现代金报）http://t.cn/zjc0OfF	"A woman was infected with HIV after having one-night-stands with many people Husband says will protect her all her life" After having problems in her relationship, she spent her time active in the night life, and was infected with HIV eventually. A healthy man gets married to her. After marriage, fortunately she gave birth to a healthy child. Later she became pregnant multiple times, seriously damaging her health. December 1, World AIDS Day, pay attention to AIDS. (Xiandai Jin Bao) http://t.cn/zjc0OfF	31 (1.52%)	http://www.china.com.cn/info/dress/2012-12/01/content_27280838.htm Xiandai Jin Bao (Modern Gold Post) is the name of a newspaper.	News of an HIV patient
#西北·狮情报#【河南艾滋病患者西安街头请喝茶】"我是艾滋病患者秦跃杰，你愿意停下来喝一杯热茶吗？"11 月 29 日，在西安鼓楼回民街的入口处，河南艾滋病感染者秦跃杰想用这种特殊的方式让更多的人了解艾滋病。12 月 1 日是第 25 个"世界艾滋病日"，他希望通过这种方式，可以被社会所接纳。	#Northwest. Lion Affairs Post# "Henan HIV patient provided tea on the street of Xian" "I am Qin Yuejie, a HIV patient. Would you be willing to stop and have a cup of hot tea?" On November 29, at the entrance of Huimin Street, Gulou, Xian, Qin Yuejie, an HIV patient from Henan, would like to use this special method to let more people understand HIV. December 1 is the 25th World AIDS Day, he hoped that through this method, he would be accepted by society.	28 (1.37%)	http://life.gmw.cn/2012-11/30/content_5852747.htm	News of an HIV patient / Health education *
#今日商报#【同性恋的他感染艾滋怕见爹娘】虽然人们谈起艾滋病不再像过去那样"色变"，但这还未完全被医疗界攻克的疾病仍让人心生畏惧。据市卫生局的数据统计，今年 1～9 月，我市共报告 HIV / AIDS 287 例，其中感染者 172 例，病人 71 例，报告死亡 12 例。http://t.cn/zjciS3n	#Jinri Shang Bao# "He, a homosexual, is infected with HIV, and is afraid to see dad and mum." Even though people would not be so scared as in the past when talk about AIDS, this disease that has not been fully conquered by the medical profession, is still frightening to many. According to the statistics of the city's Health Bureau, this year from January to September, our city reports 287 cases of HIV/AIDS in total, of which there were 172 cases of infection and 72 patients and 12 reported deaths. http://t.cn/zjciS3n	19 (0.93%)	http://dnsb.cnnb.com.cn/portal.php?mod=view&aid=5375	News / health information (News about a homosexual HIV patient and statistics from Ningbo city)

【今天是第 25 个世界艾滋病日】曾"征人微笑"的艾滋病毒感染者马治发，一年后，他后悔自己站了出来，因为他想见女儿，可找不到她了；31 岁的艾滋病毒感染者长征，做了 6 年的志愿者，他表示：虽然被"艾"了 但要活出未被命运安排的内容来……关爱艾滋病感染者，从现在做起。http://t.cn/zjc9KrU	"Today is the 25th World AIDS Day" Ma Zhifa, the HIV infectee who had "recruited smiling people", a year later, regrets that he had stood forward, because he likes to see his daughter, but he cannot find her now. 31-year-old HIV infectee Chang Zheng, has spent 6 years as a volunteer. He said, "Even though he is infected with HIV, he wants to live out the content of a life that is not mandated for his fate……Care HIV infectees, starting from now. http://t.cn/zjc9KrU	2 (0.10%)	http://hsb.hsw.cn/2012-12/01/content_8466080.htm	News of two HIV patients
**News highlights**		**4 (0.2%)**		
星报 12 月 1 日精彩导读: 1、合肥昨天首次公布 PM2.5，数据"一波三折"；<br>2、一个艾滋病犯人的"罪与罚"；3、生物、地理成绩或计入我省中考总分；<br>4、副厅长给情妇写离婚承诺书；5、奥巴马英拉眉来眼去，米歇尔打翻醋坛子；6、洛阳囚禁 6 名性奴案主犯一审被判死刑。	Star Post December 1st highlights: … 2. The "Crime and Punishment" of a criminal infected with HIV. …<br>[the rest of the Weibo post with other news is not translated here]	2 (0.10%)	http://weibo.10086.cn/_M100703339	News highlight (including a piece of news about an HIV patient)
【新闻频道●今日看点】●哈大高铁今天正式通车，记者从地面、车上、空中全方位多角度带您体验高铁之旅●世界艾滋病日：邀您关注我国艾滋病防治现状，携手向"零"艾滋迈进●最后一期"保护野生动物"我们在行动：下午 4 点直播节目邀请国家林业局保护司司长探讨我国野生动物保护现状与困境。	"News Channel. Today highlights" … World AIDS Day: Invite you to pay attention to the current status of HIV prevention and treatment in our country, to go hand-in-hand and make progress towards "zero" AIDS. …<br>[the rest of the Weibo post with other news is not translated here]	2 (0.10%)	http://native.cnr.cn/pic/201212/t20121202_511461880.html	News mentioning World AIDS Day
**Health education (World AIDS Day)**		**920 (45.0%)**		
今天是第 25 个世界艾滋病日，今年的主题是"行动起来，向'零'艾滋迈进——全民参与，全力投入，全面预防"。我国艾滋病疫情如何，预防与治疗又是怎样的情况，艾滋病人的生存现状如何？艾滋病人瞒病就医事件也让人反思，我们的社会何时才能完全消除对艾	Today is the 25th World AIDS Day. The theme of this year is "Take actions. Progress towards "Zero" AIDS – Everyone participates; fully involves; totally prevent". How is our country's HIV epidemic? How is the prevention and treatment? How is the contemporary living condition of HIV patients? The incidents of HIV patients trying to cover up their illness to avoid seeking healthcare also provoke one	474 (23.17%)	http://blog.sina.com.cn/lm/huati/health/20121129/1206689.html	Health education (Today is World AIDS Day)*

滋病人的歧视误解？详见 http://t.cn/zj5DEZW	to reflect upon until when our society will fully remove the discrimination against and misunderstanding about HIV patients? http://t.cn/zj5DEZW			
【向零艾滋迈进 特别报道】此时是 2012 年 12 月 1 日 8 时，各位声控早上好！今天是一年一度的世界艾滋病日，现在我们正式启动“@[user] #向零艾滋迈进#特别报道”，我们将以 30 分钟为频率，全方位滚动为您播报 12·1 资讯。阿声线下志愿者将在街头进行线下报道，今日之内转发本微博资讯将有机会获奖。	“Striving towards zero AIDS Special report” It is 8am, December 1, 2012. Good morning, everyone! Today is the annual World AIDS Day. Now we are formally beginning “@[user] #Towards Zero AIDS #Special report”. At a frequency of 30 minutes, we will cover information of December 1 for you at every angle. Volunteers of this program will report on the street. Repost information of this post within today will have an opportunity to win a prize.	85 (4.15%)	Original Weibo post	Health education (Striving towards zero AIDS)
世界卫生组织于 1988 年 1 月将每年的 12 月 1 日定为世界艾滋病日，号召世界各国和国际组织在这一天举办相关活动，宣传和普及预防艾滋病的知识。让我们用红丝带，传递爱！	The World Health Organization, in January 1988, designated December 1 as the annual World AIDS Day, calling upon the nations of the world and international organizations to host related activities, to promote and popularize knowledge about HIV prevention. Let us use red ribbon to pass on love!	55 (2.69%)	Original Weibo post	Health education (Origin of World AIDS Day)
在世界艾滋病日，了解艾滋病常识。反对歧视，关爱艾滋病人。	On World AIDS Day, under the basic knowledge about HIV. To fight against discrimination. Take care of HIV patients.	53 (2.59%)	Original Weibo post	Health education (to remove discrimination against HIV patients)*
#世界艾滋病日宣传专题#（二）艾滋病的传播途径？ 主要通过性接触、血液及血液制品、母婴三种途径传播。	#World AIDS Day promotion theme# (2) How is HIV transmitted? The three main routes of transmission are sexual, bloodborne & via blood products, and mother-to-child.	48 (2.35%)	http://news.cnr.cn/gnxw/200911/t20091130_505691543.shtml	Health education (routes of HIV transmission)*
#历史上的今天#今天是第 25 个世界艾滋病日，主题为“行动起来，向‘零’艾滋迈进”。艾滋病自 1981 年 12 月 1 日于美国首发后，在全球范围内迅速蔓延。1988 年 1 月世界卫生组织决定将	#Today in history# Today is the 25th World AIDS Day. The theme is “Take actions. Progress towards “zero” AIDS”. Since the first case in the USA on December 1, 1981, HIV has rapidly spread globally. In January 1988, the World Health Organization decided to designate December 1 as the annual	40 (1.96%)	Original Weibo post	Health education (Today in history)

每年 12 月 1 日定为世界艾滋病日，号召世界各国和国际组织宣传和普及预防知识。截至 2011 年底，全球共计艾滋病病毒感染者 3400 万。	World AIDS Day, to call upon the nations of the world and international organizations to promote and popularize knowledge of prevention and control. Up to the end of 2011, there were 34 million HIV positive people globally.			
#世界艾滋病宣传日宣传专题#（一）什么叫艾滋病？ 全称是获得性免疫缺陷综合征，是一种由人类免疫缺陷病毒（HIV）感染引起的传染病。这种病毒主要侵害人体免疫系统，当免疫系统被病毒破坏以后，人体由于失去了免疫能力而感染其他的疾病，最后导致死亡。	#World AIDS Day Promotion Theme# (1) What is AIDS? The full name is Acquired Immunity Deficiency Syndrome. It is an infectious disease caused by the Human Immunodeficiency Virus. This virus primarily damages the human immune system. After the immune system is damaged by the virus, the human body will be infected with other diseases due to the loss of immunity and this will eventually lead to death.	23 (1.12%)	Original Weibo post	Health education (What is AIDS?)*
避孕套又称安全套、小雨伞、小雨衣、小雨帽。相传古罗马人发明，有 3000 多年历史，以非药物的形式阻止受孕，用于在性交中阻止人类的精子和卵子结合。此外，避孕套也能防止淋病、艾滋病(HIV)等性病传播，原料通常是用天然橡胶或聚亚安酯。#世界艾滋病日# ❤️	Condom is also known as “Little umbrella”, “Little raincoat”, “Little rain cap”. It is said to be first invented by ancient Romans and has a history of over 3000 years. It uses a non-pharmaceutical means to block fertilization, to prevent human eggs from fertilization by sperms. Furthermore, condoms can also prevent the transmission of sexually transmitted diseases, such as gonorrhea and HIV. The raw materials are usually natural rubber or polyurethane. #World AIDS Day# ❤️	22 (1.08%)	Original Weibo post	Health education (condoms)*
12 月 1 日#910 互动话题#第 25 个“世界艾滋病日”。您对“艾滋病”了解吗？在艾滋病救助方面您有什么建议？欢迎登陆新闻 910 新浪或腾讯微博参与讨论。您宝贵的评论还将会在新闻 910《新闻时刻》中播出，期待您的参与。	December 1 #910 Interactive Topic# The 25th “World AIDS Day”. Do you understand “AIDS”? Do you have any suggestions regarding saving and helping people with HIV? You are welcome to visit News 910 Sina or Tencent Weibo to participate in the discussion. Your valuable comment will also be broadcasted in News 910 “News Hour”. Looking forward to your participation.	17 (0.83%)	Original Weibo post	Health education (Interactive activities)
#美女作家安奕送避孕套#给力分享了，哈哈。全免费 [猫扑大杂烩]80 后美女作家安奕艾滋日微薄送安全套，碉堡了。 http://t.cn/zjc92xl	#Pretty lady author An Yi gives away condoms# Motivated to share, haha. Completely free [Mop Hodgepodge] Pretty lady author of the 80s generation, An Yi, gives away condoms on Weibo. Too powerful!	17 (0.83%)	http://dzh.mop.com/whbm/20121130/0/zzgS58I2b2265aF5.shtml Mop (猫扑) is a very popular website in China (www.mop.com). Mop Hodgepodge (猫扑大杂烩) is	Health education (health promotion activity to give away free

			a community of different discussion threads on a variety of topics.	condoms)
【杜蕾斯携手中国预防性病艾滋病基金会防艾】杜蕾斯向中国预防性病艾滋病基金会捐赠百万安全套，用于中国艾滋病的防治。杜蕾斯希望在这次捐赠之后，成为中国预防性病艾滋病基金会“艾滋病防治事业合作伙伴”中的重要 点击详情：http://t.cn/zjcqkAO	“Durex goes hand in hand with the Foundation for the prevention of sexually transmitted diseases and AIDS to prevent HIV” Durex donates one million condoms to the Foundation for the prevention of sexually transmitted diseases and AIDS. Durex hopes that after this donation, it will become an important “partner in the prevention of AIDS” with the Foundation for the prevention of sexually transmitted diseases and AIDS. For details, click on http://t.cn/zjcqkAO	9 (0.44%)	http://www.91kangkang.com/c/44WWlXbjuY1l.html	Health information (News of Durex’s donation)
【探访中国唯一一所艾滋病患儿学校】临汾“绿色港湾”红丝带学校，生活着 23 名艾滋病患儿。学校于 2011 年 12 月 1 日挂牌成立，成为我国目前唯一一所艾滋病患儿学校。这些孩子大多是由母婴传播而感染艾滋病。目前学校为他们开设了语文、数学、英语、体育以及心理等多项课程。via 新华网 http://t.cn/zjtnwwl	“Visit China’s only school for kids infected with HIV” 23 kids infected with HIV live in the red ribbon school “Green Bay” in Linfen. The school was founded on December 1, 2011, and has become the only school for kids infected with HIV in our country as of today. These kids were mainly infected via mother-to-child transmission. Currently the school provides a number of courses on Chinese, mathematics, English, sports, psychology and etc.. via Xinhua Net http://t.cn/zjtnwwl	8 (0.39%)	http://news.xinhuanet.com/photo/2012-11/30/c_124027721.htm	Health education (A school for HIV-positive children)
［世界艾滋病日］日常生活接触会感染艾滋病病毒吗？艾滋病病毒不会通过与性无关的日常接触传染。与其他病毒不同，与艾滋病病毒感染者握手、拥抱、共用卫生间、共用一个杯子喝水或者接触到艾滋病病毒感染者的喷嚏和咳嗽，都不会感染艾滋病病毒。所以，不必害怕与艾滋病病毒感染者进行日常交流。	[World AIDS Day] Will HIV be transmitted via daily contacts? HIV cannot be transmitted via daily contacts that are unrelated to sex. Unlike other virus, shaking hands with HIV virus infectees, hugging them, sharing toilets with them, using the same cups to drink water or in contact with their sneezes or coughs, will not infect others with HIV. Therefore, do not be afraid of daily contacts with HIV infectees.	7 (0.34%)	Original Weibo post	Health education
北京地坛医院有个专门收治艾滋病人的病房，在那里大家都有个习惯，如果患者皮肤没有溃破，如果不是进行侵入性操作的检查。医护人员在查房时是不带手套的，用手拍拍患者肩膀，用手触诊	In Beijing Ditan Hospital, there is a ward that is specialized in treating HIV patients. There, everyone has a customary practice, that if there is no wounds on the patient’s skin and if they are not performing invasive check-up, the medical personnel would not wear gloves during their grand rounds. They use	6 (0.29%)	Original Weibo post	Health education (Comment on a hospital)

脖部淋巴结，用手揭开衣服看看身上的皮疹……别小看这个，许多患者在看到医护人员不带手套接触自己，都感动的哭了。	their hands to pat the patients' shoulders; use their hands to touch the lymph nodes on their necks; use their hands to unbutton the patients' clothes to look at their rash…… Don't belittle this. Many patients are so moved and cry, when they see that the medical personnel do not wear glove to touch themselves.			
今天是世界艾滋病日，這套微電影是有香港電台及衛生署紅絲帶中心一齊製作的微電影，希望大家可以透過這套微電影認識和預防艾滋病，了解和關懷艾滋病病人！	Today is World AIDS Day. This micro-movie is jointly produced by Radio & Television Hong Kong and the Department of Health Red Ribbon Center. Hope that through this micro-movie, we all can know and prevent HIV, and understand and care for the HIV patients!	6 (0.29%)	Original Weibo post	Health education (Comment on a public service advertising)
【天津调整艾滋病定点医院布局】#天津新闻#天津统筹规划了艾滋病定点医院布局。市第二人民医院(原传染病医院)主要负责艾滋病感染者和病人抗病毒治疗和收治危重、重症机会感染、有伴发疾病或者合并症的艾滋病病人；市海河医院负责收治艾滋病和肺结核双重感染的病人。今后将适时增设一所定点治疗医院。	"Tianjin adjusted the plan of specified HIV hospitals" #Tianjin News# Tianjin has planned the specified HIV hospitals. The Municipal 2nd People's Hospital (formerly Infectious Disease Hospital) is primarily responsible for the antiviral treatment for HIV infectees and patients, and for the inpatient treatment (including that in intensive care units) of serious HIV cases with opportunities infections, accompanying diseases or complications; Municipal Haihe Hospital is responsible for inpatient treatment for patients coinfected with HIV and TB. In the future, there will duly be one more hospital specified [for HIV treatment].	6 (0.29%)	Original Weibo post	Health information (News about hospitals in Tianjin Municipality)*
［世界艾滋病日］什么是女用安全套？女用安全套是目前市面上唯一一种由女性主动使用的避孕措施。女用安全套由坚固耐磨、质地柔软、透明的聚氨酯材料制成的保护套。在性交前，将其放入阴道中，覆盖住阴道粘膜表面。每次性交时正确使用，可以防止怀孕和包括艾滋病在内的各种性传播疾病。	[World AIDS Day] What is a female condom? Female condom is currently the only female-initiated birth control method. Female condom is made of polyurethane materials that are robust and resistant to wearing, soft in texture, and transparent. Before sexual intercourse, insert it into the vagina and cover the muscosal surface of the vagina. If it is used properly in every sexual intercourse, it can prevent pregnancy and prevent the transmission of various sexually transmitted diseases, including HIV.	6 (0.29%)	Original Weibo post<br>Note: The content of this post is not entirely correct, as the birth control pill is also a female-initiated birth control method.	Health education (Female condoms)
【卫生部考虑将艾滋病职业暴露列入职业病范畴】日前，在卫生部防艾工作媒体通气会上，卫生部疾控局副巡视员孙	"Ministry of Health is considering listing occupational exposure of HIV as an occupational disease" A couple of days ago, in a Ministry of	5 (0.24%)	http://www.cn-healthcare.com/news/gwjk/2012-11-30/content_414330.html	Health information (News about

新华介绍，卫生部准备在原来的基础上进一步完善职业暴露相关办理办法，正考虑把艾滋病职业暴露列入职业病。官方透露，“到目前为止，医务人员没有发生一例感染。” http://t.cn/zjcHvti	Health HIV prevention media briefing, Sun Xinhua, associate inspector of the Disease Prevention Bureau of the Ministry of Health, introduced that, the Ministry of Health plans to build upon the original foundation to further perfect the related methods to handle occupational exposure. They are considering to list HIV occupational exposure as an occupational disease. According to the official authority, “up to now, there had never been a case of infection among medical personnel.” http://t.cn/zjcHvti			potential legal change regarding occupation-al exposure to HIV)*
#艾滋病日#艾滋病病毒不会借助空气、水或食物进行传播。在日常工作和生活中与艾滋病人和感染者的一般接触，如握手、拥抱、共同进餐，办公用具等不会感染艾滋病。 一个微笑，一次握手，一个拥抱……关注艾滋病日，关爱艾滋病人，少一点歧视，多一点关爱...	#AIDS Day# HIV would not be transmitted via air, water or food. Common contacts with HIV patients and infectees in daily work and life, such as handshakes, hugging, having meals together, work instruments etc., would not transmit HIV. A smile, a handshake, a hug…… Care about AIDS Day, take care of AIDS patient, less discrimination, more care and love.	5 (0.24%)	Original Weibo post	Health education
［世界艾滋病日］什么是安全性行为?性行为不可能是 100%完全安全的。安全性行为包括采取各种预防措施，降低通过性行为传染或感染性传播疾病，包括艾滋病的可能性。每次性行为时都正确使用安全套就是一种安全的性行为。不正确地使用安全套可能导致安全套滑落或者破裂，因而降低保护作用。	[World AIDS Day] What is safe sex? Sexual intercourse is impossible to be 100% safe. Safe sex involves the application of various prevention methods, to lower the possibility of transmitting or being infected with sexually transmitted diseases, including HIV. To use condoms in every sexual intercourse is a type of safe sex. Using condoms inappropriately may cause the condoms to slip or break and therefore reduce the protective function.	5 (0.24%)	Original Weibo post	Health education (Safe sex)
【中国艾滋病疫苗自主创新之路又积跬步】中国科学家攻克了以腺病毒为载体的艾滋病疫苗受抗体阻碍难题。此类疫苗因为腺病毒载体易被抗体中和而难以发挥效用。项目负责人陈凌博士和孙彩军博士介绍，此项目是继今年 8 月科技重大专项艾滋病疫苗 II 期临床试验启动后，又一项有益探索。 http://t.cn/zjcasqw	“A small step forward in independent innovation of HIV vaccine in China” Chinese scientists overcame the challenge of obstruction by antibodies against the HIV vaccine using adenovirus as a vector. This type of vaccine is difficult to be effective as adenovirus vectors are easily neutralized by antibodies. Project investigators Dr. Chen Ling and Dr. Sun Caijun introduced that this project is another beneficial exploration since the initiation of the Phase II clinical trial of HIV vaccine that is a [nationally] important science and technology project. http://t.cn/zjcasqw	4 (0.20%)	http://www.yxj.org.cn/department/kangganran/kgrxsjz/2012113013193313432.htm	Health information (News of HIV vaccine development)

【今日重要新闻】1、国家统计局和中国物流与采购联合会 1 日将公布 11 月制造业采购经理人指数 PMI；2、哈尔滨-大连高铁 1 日起正式通车运营；3、1 日是第二十五个世界艾滋病日；4、五个药品流通标准 1 日起正式实施；5、青海省 1 日起调整最低工资标准；6、人民法院司法警察条例 1 日起正式实施 。	"Today Important News" … 3. [December] 1st is the 25th World AIDS Day; …<br>[the rest of the Weibo post with other news is not translated here]	4 (0.20%)	http://t.qq.com/p/t/127443066211244	News mentioning World AIDS Day
世界艾滋病日 苹果店换红装：12 月 1 日是"世界艾滋病日"，与去年一样苹果的零售店和官网都换上了红色的主题。多家位于纽约的苹果店在内目前都已经将店中的白色苹果 logo 都被"上色"称红色。http://t.cn/zjcRPpS	World AIDS Day Apple stores clothed in red: December 1st is "World AIDS Day". Like last year, Apple stores and their official website have put on the red theme. Many Apple stores in New York have colored their white Apple logo to red. http://t.cn/zjcRPpS	4 (0.20%)	http://www.feng.com/apple/news/2012-12-01/World_AIDS_Day_apple_shop_for_red_loading_539959.shtml	News about World AIDS Day
今天是#世界艾滋病日#，据《2011 年中国艾滋病疫情报告》显示，截至 2011 年底，估计中国存活艾滋病病毒感染者和艾滋病病人 78 万人，女性占 28.6%，估计 2011 年新发 HIV 感染者 4.8 万人。经母婴传播占全国估计数的 1.1%。	Today is #World AIDS Day# According to to Epidemiologic Report of HIV in China 2011, up to the end of 2011, the estimated number of living HIV infectees and AIDS patient was 780,000, of whom 28.6% were female. The estimated number of incidence case of HIV infection was 48,000, of which 1.1% was estimated to be transmitted mother-to-child.	3 (0.15%)	http://news.xinhuanet.com/society/2011-11/29/c_111203751.htm	Health information (National statistics of HIV)
【视频：艾滋病知识宣传片】http://t.cn/zjVAnzy	"Video: AIDS knowledge promotional video" http://t.cn/zjVAnzy	3 (0.15%)	http://v.youku.com/v_show/id_XMjY0NTkzNTA4.html	Public service advertising
【12 月 1 日 世界艾滋病日】我们虽然取得了成绩，但是"艾滋病依然是全世界对健康形成最严重威胁的疾病之一，给家庭带来悲剧性影响，也削弱了社会和地方社区。" 伊琳娜·博科娃总干事致辞 http://t.cn/zjc5vww	"December 1 World AIDS Day" Even though we have attained some achievements, "AIDS is still one of the diseases that cause the most severe threat to health globally. It brings tragic influence to families and weakens society and communities." General Secretary Irina Bokova said. http://t.cn/zjc5vww	3 (0.15%)	http://unesdoc.unesco.org/images/0021/002185/218510c.pdf	Health information (Address from Irina Bokova, UNESCO General Secretary)
［世界艾滋病日］如何使用男用安全套？首先不要使用过期的安全套。	[World AIDS Day] How do you use male condoms? First, do not use expired condoms. When you remove the condom from its package, beware not to	2 (0.10%)	Original Weibo post	Health education

在取出安全套时就要小心防止弄破。将正确的一面朝外，轻轻挤出安全套前端小囊内空气并将安全套套到勃起的阴茎上。从插入开始到性交结束，应一直戴着安全套。性交结束后，握住安全套的底部将其取下。记住，安全套不可重复使用。	break it. Get the correct side to face outside. Softly, squeeze away the air from the small extrusion at the tip of the condom and cover the erected penis with the condom. One should wear the condom from the beginning to the end of the sexual intercourse. After the end of the sexual intercourse, hold the bottom of the condom and remove it. Remember, never reuse a condom.			
**Health information (Health statistics)**		**160 (7.8%)**		
【我国艾滋病传播新趋势】今年前 10 月数据显示：男男性传播占比升至两成；15-24 岁，以及 50 岁以上人群感染数逐年上升。	"Our country HIV transmission New trend" Data from the first 10 months of this year show that: The proportion of HIV transmission via sexual contacts between men [among different routes of HIV transmission] has increased to 20%; the number of infected people in the age group of 15-24 years old and that of >50 years old also increase annually.	50 (2.44%)	http://epaper.jinghua.cn/html/2012-11/29/content_1904511.htm	Health information (National statistics of HIV on transmission via homosexual contacts)*
【浙江卫生厅公布艾滋病疫情】卫生厅发布艾滋病疫情情况，截至今年 11 月 25 日，浙江全省新发现感染者和病人 2513 例，较去年同期增长 20.6%，近两年浙江省每年新报告人数都以 20% 左右的幅度上升，快速上升的趋势有所缓解。60 岁以上男性和学生感染者明显增多。http://t.cn/zjcKXnN	[Zhejiang Health Department announces HIV incidence data] The Health Department announces HIV incidence data. Up to November 25, this year, there were 2513 newly identified infected cases/patients in the whole of Zhejiang, an increase of 20.6% compared with the same period last year. In the recent two years, the number of newly identified cases has been increasing at a rate of around 20%. The trend of rapid increase has been slowed down. There was a marked increase among men who are over 60 years of age and among students. http://t.cn/zjcKXnN	23 (1.12%)	http://bbs.qjwb.com.cn/index.php?m=cms&q=view&id=8481	Health information (Statistics from Zhejiang Province)*
今天是"世界艾滋病日"。据联合国的数据显示，全球每天有 7100 多人感染艾滋病。尤其在环境恶劣的少数民族地区，许多人没有这方面的防范意识，艾滋病传播得非常快速。所以，从今天开始，我发愿要为这些地区的人防治艾滋病，略尽自己的绵薄之力。假如你身边有这样的患者，也请尽己所能地给他们	Today is "World AIDS Day". According to the data from the United Nations, every day there are 7100 people being infected with HIV globally. Especially in the poor environment of the ethnic minority areas, many people do not have any awareness about prevention and control of HIV, and therefore HIV spreads very rapidly. So, starting today, I pledge that I will help people in these areas prevent and control HIV, doing my very best. If you have patients around	22 (1.08%)	Original Weibo post	Health information (Globl statistics from the United Nations)*

关爱！	you, please do your best to take care of them!			
【河南艾滋疫情报告 学生中同性感染者迅速上升】河南省疾控中心发布本年度艾滋病疫情分析报告，发现经性传播感染者明显上升，学生中同性传播感染者所占比例上升迅速，从 2009 年的 1.39%，上升到 2012 年的 34.2%。报告发现，60 岁以上年龄组报告异性性传播感染数量增加明显。全文地址：http://t.cn/zjcl6cm	[Henan HIV incidence report Rapid increase among homosexuals among students] Henan Provincial CDC announces the report of the analysis of HIV incidence of this year. It is found that incidence infected via sexual transmission has increased markedly. The proportion of homosexual transmission among students has increased rapidly, from 1.39% in 2009, to 34.2% in 2012. It is found that, heterosexual transmission in the age group of over 60 years, has increased markedly. Full text: http://t.cn/zjcl6cm	22 (1.08%)	http://www.danlan.org/disparticle_42144.htm	Health information (Statistics from Henan Province)*
【卫生部统计显示我国属艾滋病低流行国家】今天是世界艾滋病日。卫生部最新统计显示，截至今年 10 月底，我国累计报告艾滋病感染者和病人 492,191 例，在全球范围内，我国仍属于低流行国家，艾滋病防治取得明显成效。目前，全国几乎所有县都已经具备艾滋病检测能力。http://t.cn/zjcDTZa	"Statistics from the Ministry of Health show that our country is one with low HIV incidence" Today is World AIDS Day. The most recent statistics from the Ministry of Health shows that up to the end of October this year, the cumulative incidence of HIV infectees and patients in our country were 492,191 cases. Globally, our country is a low incidence country. HIV prevention achieves obvious effect. Currently, nearly every county in the country has acquired the capacity to perform HIV tests. http://t.cn/zjcDTZa	15 (0.73%)	http://www.law-lib.com/fzdt/newshtml/22/20050822195820.htm	Health information (National Statistics of HIV)*
【深圳艾滋病感染九成由"性传播"】深圳市健康教育与促进中心透露，今年 1~9 月，全市进行艾滋病毒筛查 89 万人次，感染者 987 例，比去年同期（856 例）上升 15.3%。20 至 39 岁青壮年占 80.2%，大部分为流动人口。据筛查情况，深圳艾滋病疫情以性传播途径为主，约占 90.4%，其中经同性/双性传播比例占 39.7%。	"90% of HIV transmission in Shenzhen was "sexually transmitted"" Shenzhen Municipal Center for Health Education and Promotion revealed that from January to September this year, there had been 890,000 HIV tests conducted in the city. There had been 987 cases of HIV infection. Comparing with the same period last year (856 cases), there is an increase of 15.3%. Of these cases, 80.2% were young adults between the age of 20 and 39 years. Most of them were mobile population. According to the testing situation, the HIV epidemic in Shenzhen is primarily due to sexual transmission, that accounts for 90.4%, of which homosexual/bisexual transmission was 39.7%.	5 (0.24%)	http://wb.sznews.com/html/2012-12/01/content_2298480.htm	Health information (Statistics from Shenzhen City, Guangdong Province)*
【艾滋病迎发病高峰：东莞"男同"感染率最高】12 月 1 日是第 25 个"世界艾滋	"HIV incidence peak: Dongguan "male homosexauls" have the highest incident rate" December 1 is the 25th "World AIDS Day". This year	4 (0.20%)	http://news.cntv.cn/china/20121129/102618.shtml	Health information (Statistics

病日"。今年广东省艾滋病疫情感染数和发病数分别上升 12.6%和 18.1%，性传播成为主要传播途径，珠三角男同感染率攀至 10%，东莞最高为 22.9%http://t.cn/zj5EpJj 艾滋病离我们很远吗？防艾必读，猛戳配图！PS，配图亮了	the HIV incident rate and the rate of developing AIDS have increased by 12.6% and 18.1% respectively. Sexual transmission was the major route of transmission. The incident rate among male homosexuals has increased to 10%. That of Dongguan is the highest: 22.9%. http://t.cn/zj5EpJj Is AIDS very far away from us? Must read for AIDS prevention, click the picture! PS, The pictures are great!			from Dongguan City, Guangdong Province)
【苏州今年新增 368 例艾滋病病毒感染者和病人，其中学生 11 例】今年 1 月至 10 月，我市新报告艾滋病病毒感染者和病人 368 人，较去年同期增长 39.4%。1992 年至今，我市累计报告艾滋病病毒感染者和病人已增加至 1536 人。今年新报告感染者中，学生有 11 例，年龄最小的刚满 18 周岁，往年通常只有 1 至 2 例。（A02 版）	[This year there were 368 incident cases of HIV infectees and patients in Suzhou, of whom 11 cases were students] From January to October this year, our city reports 368 incident cases of HIV infectees and patients, an increase of 39.4% compared to the same period last year. Since 1992, the cumulative incidence of HIV infectees and patients in our city has increased to 1536. Among the newly reported incident cases, 11 cases were students, of whom the youngest one has just reached the age of 18 years. In the past, there was only 1 or 2 such cases. (Page A02)	4 (0.20%)	http://sz.sohu.com/20121201/n359186025.shtml	Health information (Statistics from Suzhou City, Jiangsu Province)
【湖南头 10 个月感染艾滋病患者 15971 例 衡阳排第一】截至 2012 年 10 月 31 日，湖南累计报告艾滋病病毒感染者和艾滋病人 15971 例(死亡 4819 例)，其中报告艾滋病病人 6989 例(死亡 2546 例)。累计报告病例排前五位的地区依次是衡阳市、永州市、怀化市、长沙市和邵阳市。 http://t.cn/zjcQSfC	[In the first ten months there were 15971 incident cases of HIV infection in Hunan. Hengyang ranked number one] Up to October 31, 2012, the cumulating incidence of HIV infectees and patients were 15971 cases (4819 deaths), of whom 6989 cases of AIDS patients (2546 deaths). The top five regions in the cumulative incident reports are Hengyang City, Yongzhou City, Huaihua City, Changsha City, and Zhaoyang City. http://t.cn/zjcQSfC	4 (0.20%)	http://hunan.sina.com.cn/news/s/2012-12-01/081428013.html	Health information (Statistics from Hunan Province)*
【上海今年防艾喜忧参半：死亡 134 例】今年 1 月 1 日至 11 月 20 日的统计，本市新发报告艾滋病毒感染者 1479 例，同期增幅 14.3%，艾滋病病人 727 例，同期增幅 42.8%，死亡病例 134 例，增幅 109.4%。有 18 例阳性孕产妇接受预防艾滋病母婴传播干预措施，实施率 100%，无一例新生儿感染	"Shanghai has a mixed picture of HIV prevention this year: 134 deaths" According to the statistics from January 1 to November 20 this year, our municipality reports 1479 new incident cases of HIV infection, an increase of 14.3% comparing with the same period last year; 727 AIDS cases, an increase of 42.8% comparing with the same period last year; and 134 deaths, an increase of 109.4%. There were 18 cases of HIV positive pregnant women receiving	3 (0.14%)	Original Weibo post	Health information (Statistics from Shanghai Municipality)

者。记者陈青	intervention preventing mother-to-child transmission. The implementation rate is 100%. No infants were born infected. Journalist Chen Qing.			
【世界艾滋病日：前十月珠海报告 113 例】今天是世界艾滋病日，今年前 10 月珠海共报告艾滋病病毒感染者 80 例和艾滋病病人 33 例共 113 例，其中死亡 23 例，比 2011 年同期增 7.6%。10 月 31 日珠海累计报告艾滋病病毒感染者和艾滋病病人 818 例，其中艾滋病病毒感染者 465 例，艾滋病病人 353 例，死亡 252 例，现存活 566 例。	"World AIDS Day: In the first 10 months, Zhuhai reports 113 cases" Today is World AIDS Day. In the first 10 months of this year, Zhuhai reports 80 incident cases of HIV infection and 33 cases of AIDS, a total of 113 cases, of which 23 died. Comparing with the same period in 2011, there is an increase of 7.6%. By October 31, the cumulative incidence of HIV infectees and AIDS patients were 818 cases, of which there were 465 HIV infectees and 353 AIDS patients; 252 had died and 566 cases were alive.	3 (0.15%)	http://www.zhnews.net/html/20121201/080117,363998.html	Health information (Statistics from Zhuhai City, Guangdong Province)
「佛山累计报告 HIV 抗体阳性者 4820 例」今日是第 25 个世界艾滋病日。市卫生局统计数据显示，目前全市累计报告 HIV 抗体阳性者 4820 例，疫情总体呈低流行态势，不过近年，经同性性接触传播的比例增长迅速。 http://t.cn/zjVhN5e	"Foshan reports cumulative incidence of 4820 HIV positive cases" Today is the 25th World AIDS Day. According to the statistics of the municipal health bureau, the whole city reports a cumulative incidence of 4820 HIV positive cases. Overall, the epidemic level is low. However, the proportion of transmission via homosexual sexual contacts has rapidly increased. http://t.cn/zjVhN5e	3 (0.15%)	http://my.tv.sohu.com/us/87275464/50109358.shtml	Health information (Statistics from Foshan City, Guangdong Province)
【重庆今年新增 HIV 感染者 2478 人】今天是第 25 个世界艾滋病日，宣传主题是"行动起来，向'零'艾滋迈进"。市卫生局通报：截至 10 月 31 日，重庆累计报告 HIV 感染者 15298 人，其中艾滋病病人 4686 人，累计死亡 2601 人。全市新增艾滋病病毒感染者 2478 人（性接触传播所占的比例达 91.4%）较去年同期增加了 29.5%。	"There are 2478 incident cases of HIV infection in Chongqing this year" Today is the 25th World AIDS Day. The promotional theme is "Take actions; Making progress towards 'zero' AIDS". Municipal health bureau reports that up to October 31, the cumulative incidence of HIV infection was 15,298, of which 4686 were AIDS patients. The cumulative number of deaths were 2601. The number of new incident cases in the whole city were 2478 (of which 91.4% was infected via sexual contacts), an increase of 29.5%, comparing with the same period last year.	2 (0.10%)	http://native.cnr.cn/city/201212/t20121201_511460364.shtml	Health information (Statistics from Chongqing Municipality)
**Health education (Political figures)**		**124 (6.1%)**		
【习近平看望艾滋病患者：防治艾滋有治无类】在第 25 个世界艾滋病日即将到来之际，习近平 30 日到北京丰台社区看望艾滋病患者，参加防艾志愿者	"Xi Jinping visits HIV patients: Prevent and Control HIV Treatment without discrimination" As the 25th World AIDS Day is approaching, on the 30th [November], Xi Jinping went to Fengtai community, Beijing, to visit HIV patients and to participate in	94 (4.59%)	http://news.xinhuanet.com/politics/2012-11/30/c_113867865.htm	News of General Secretary Xi visiting HIV/AIDS

培训交流活动。防艾需要全民参与，有治无类。http://t.cn/zjc4nEh @[user] 今天（12 月 1 日）是世界艾滋病日，社会的平等对待是对他们最大的关爱！	exchange activities with volunteer training for HIV prevention and control. The prevention and control of HIV requires the full participation of the whole population and treatment without discrimination. http://t.cn/zjc4nEh @[user] Today (December 1) is World AIDS Day. The biggest care and love that the society can offer to them is equal treatment.			patients
今天是世界艾滋病日。昨天习看望北京的艾滋病患者，亲自与他们握手。同时央视反复播出彭做的关爱艾滋病患者的公益广告，国毋以这样的方式首秀真的让人耳目一新。夫妻俩一起为珍惜生命关爱健康呐喊值得称赞。但愿这将成为实干兴邦的良好开端！	Today is World AIDS Day. Yesterday Xi visited AIDS patients in Beijing, shaking their hands in person. Meanwhile, China Central Television repeatedly broadcasted the public service advertisement on taking care of AIDS patients made by Peng Liyuan. It is refreshing to see the first lady run a show like this. The husband and wife's call for the value of life and caring for health is really worth our appreciation. I hope that this will be a good beginning for real work that builds a country!	11 (0.54%)	Original Weibo post<br><br>Note: Peng Liyuan is the wife of Xi Jinping.	Health education (Comment on Peng Liyuan's public service advertisement)*
顾长卫导演艾滋病反歧视公益短片，《永远在一起》。世界卫生组织艾滋病防治亲善大使、中国卫生部预防艾滋病义务宣传员彭姐姐倾情出演（多图慎入，美疯了呀）。参考视频：http://t.cn/zjcYvYf	Gu Changwei directs a short movie of public service on anti-HIV- discrimination, "Forever together". World Health Organization AIDS prevention ambassador, China's Ministry of Health AIDS prevention voluntary promoter sister Peng [Liyuan] took the lead in the cast. (Many photos. Take caution to enter. Really beautiful!) Reference video: http://t.cn/zjcYvYf	10 (0.49%)	http://ent.sina.com.cn/m/p/2009-11-22/22282780079.shtml<br><br>Note: Peng Liyuan is the wife of Xi Jinping.	Health education (News about Peng Liyuan's public service short movie)*
#微读京华#【温家宝:想起他们，总觉得还应做得更好】#世界艾滋病日#前夕，温家宝主持召开艾滋病防治工作座谈会称，当年见过的感染者和患者不在人世了，想起他们，总觉得我们的工作还应做得更细些、更好些。性病艾滋病预防控制中心主任吴尊友称，对病人来说，歧视比病毒更可怕。http://t.cn/zjcXlvX	#Micro-read Jinghua# "Wen Jiabao: Thinking of them, always think that it should be done better" On the eve of #World AIDS Day#, chairing the forum of HIV prevention work, Wen Jiabao said that the infectees and patients that he had seen in those days had all passed away. Thinking of them, he always thought that we could do better and more detailed. Wu zunyou, the director of the Center for the prevention and control of sexually transmitted disease and HIV said that to the patients, discrimination is more terrible than the virus. http://t.cn/zjcXlvX	9 (0.44%)	http://epaper.jinghua.cn/html/2012-12/01/content_1911846.htm	News of Premier Wen Jiabao's comment on HIV prevention work in China*
**Alternative health information / Traditional Chinese Medicine**		**6 (0.3%)**		
【2013 泌尿男科入学考试】新婚燕	"2013 Male urology entrance exam" A friend who	6 (0.29%)	Original Weibo post	Alternative

尔，友携妻来诊。诉：尿频尿急尿痛。诊：蜜月病。嘱：多饮水，忌房事。数日后，再见，问：愈否？友无奈：妻嗓子疼，声嘶。医笑：转移了。再嘱：备肛泰。1：名词解释：蜜月病 2：本文提及几种性交方式？按感染艾滋病风险，由高到低。	has just got married brought his wife to the clinic. He complained of frequent urination, urinary urgency and pain during urination. The diagnosis was “honeymoon disease”. The advice given was to drink more water and to avoid sexual intercourses. A few days later, we met again. I asked, “Recovered?” My friend was frustrated and said, “My wife has sore throat and hoarseness. The doctor smiled and said, “Relocated,” and advised, “To prepare with Gangtai”. 1: Explanation of terminology: “honeymoon disease”. 2: Which types of sexual intercourses were mentioned in the text? According to the risk of HIV infection, arrange them from high to low.		Note: Gangtai is a pharmaceutical for the treatment of hemorrhoids and it is derived from Traditional Chinese Medicine.	health information / Traditional Chinese Medicine
**Entertainment**		**43 (2.1%)**		
【世界艾滋病日：中国首届自慰大赛深圳开赛】这可能是最令人咋舌的集体“打飞机”。2012 年 11 月 28 日下午，在“世界艾滋病日”即将到来之际，中国首届自慰大赛在深圳梧桐山艺术小镇拉开序幕。本次大赛的主题是防艾滋、重健康、性冲动、莫违法、促和谐、可自慰。http://t.cn/zjt1UGC 现在国人也这么重口?	“World AIDS Day: China’s first masturbation competition began in Shenzhen” This can possibly be the most absurd event of collective masturbation. In the afternoon of November 28, 2012, as the World AIDS Day is approaching, China’s first masturbation competition began in the little town of Yishu, in Wutongshan, Shenzhen. The theme of this competition is: Prevent AIDS; Emphasize health; With sexual impulse, do not break the law; To promote harmony, one can masturbate. http://t.cn/zjt1UGC Is the taste of our countrymen really that bad?	36 (1.76%)	http://www.yxdown.com/news/201212/77518.html	Entertain-ment
【向零艾滋迈进 特别报道】中广网 30 日消息：11 月 30 日晚，在第 25 个世界艾滋病日前夕，一场主题为“红动中华·善行天下”的 2012 年世界艾滋病日主题慈善夜在北京富力万丽酒店举行。活动由中央电视台主持人沙玛阿果和郎永淳联袂主持。图为中华红丝带基金形象大使章子怡同小学生进行互动交流。	“Making progress towards zero HIV special report” China Radio Net 30th report. In the evening of November 30, on the eve of the 25th World AIDS Day, the 2012 World AIDS Day thematic charity night show, titled “Red Motivated China; Charity works under the heaven”	7 (0.34%)	http://news.ifeng.com/gundong/detail_2012_11/30/19723628_0.shtml	Entertain-ment / HIV education (News of a charity night)*
**Social issues**		**78 (3.8%)**		

又到 12 月 1 日世界艾滋病日了，又想起高耀洁老人。是她以薄弱的身躯，冲破重重阻力，揭露农村地区因采供血导致的艾滋病蔓延，揭开了中原血祸的真相，救助了无数艾滋病人和孤儿。而她却被当地官员打击，不得不以八十高龄孤身一人远走他乡。真是想你想得我肝肠断啊！浮槎万里，乡关何处	It is December 1 World AIDS Day again. This reminds me of the old lady Gao Yaojie. With her weak body, overcoming heavy resistance, she exposed the spread of HIV through selling and collecting blood in rural areas, revealing the truth about the bloodborne disaster in central China, and saving numerous HIV patients and orphans. Nonetheless, she was persecuted by the local officials and had to leave for a foreign country at the age of 80. It breaks my heart when I think of you! A raft floats for ten-thousand miles. Where is its home?	32 (1.56%)	Original Weibo post	Social issue (Comment on the famous Dr. Gao Yaojie, who advocated for HIV patients)*
#零就业歧视# 首选要建立在消除医疗歧视和消除制度性歧视的基础上，请@[user] 抓住目前中国国家领导人对艾滋病人就医歧视的关注和指示，落实承诺，一鼓做气，集中资源，彻底解决这个！ @联合国人权高专办	#Zero employment discrimination# The best option is to build upon the foundation of removing medical discrimination and removing institutional discrimination. Hope that @[user]would take advantage of China’s national leaders’ concern and instructions regarding the discrimination experienced by HIV patients with regard to access to healthcare, to put promise into practice, to get motivated, to focus resources, completely solve this problem! @United Nations Human Rights High Commissioners Office	25 (1.22%)	Original Weibo post	Social issue (Comment on the employment of HIV patients)*
【第 68 期中顾视点：保障艾滋病人就业权 公益律师在行动】12 月 1 日是一年一度的世界艾滋病日,而就业问题一直是困扰艾滋患者生活的沉重话题。近日，南昌一名报考教师岗位的年轻人因查出携带艾滋病毒而遭决绝，一方面是对艾滋患者就业权利的保护，另一面却遭遇公务员体检标准的拒绝。 http://t.cn/zjcXaZ4	[No. 68 Zhong Gu Perspective: Protect the right to employment of HIV patients Lawyers take action to defend public interest] December 1 is the annual World AIDS Day. Employment has long been a heavy topic that disturbs the lives of HIV patients. Recently, a young person was denied a teaching position because he/she was found to carry HIV. On the one hand, there was protection of the right to employment of HIV patients. On the other hand, it was rejected by the body check standard for civil servants. http://t.cn/zjcXaZ4	17 (0.83%)	http://shidian.zhuanti.9ask.cn/zt2012/20121130_71.shtml	Social issue (Comment on the employment of HIV patients)*
【新京报：消除艾滋歧视,公务员招考要带头】反艾滋歧视，政府部门理应做好表率。如果一方面要求企业等用人单位在招聘中不得歧视艾滋病感染者，不得辞退感染艾滋病的员工，一方面却在政府部门自身的招考工作中，将艾滋病	“Beijing News: To remove AIDS discrimination, Civil servant entrance examination should take the lead” Government departments should set the example for anti-AIDS discrimination. If the government departments, on the one hand, requests companies or other employers cannot discriminate HIV infectees in the recruitment process and cannot	4 (0.20%)	http://news.sina.com.cn/pl/2012-12-01/023925703911.shtml	Social issue (discrimination against HIV patients)

感染者拒之门外，这叫其他用人单位如何信服？http://t.cn/zjcKJkE	lay off an employee who has been infected with HIV, and on the other hand, they reject HIV infectees in their own recruitment process, how would they convince other employers? http://t.cn/zjcKJkE			
**Personal comment**		**14 (0.7%)**		
【一分钟世界发生什么】250 个宝宝出生，113 个贫困，15 个有疾病。一个普通美国家庭挣了 9.6 美分，奥普拉挣了 523 美元；世界人均挣了 1.3 美分。美国 IT 从业者挣了 13 美分，印度的则挣了 2.5 美分。耐克挣了 3 万 6505 美元，而其在越南工厂的工人则有的只挣了 0.14 美分。9 人感染艾滋，107 人死去，18 人死于饥饿。	"What is happening in the world in a minute" 250 babies are born, of whom 113 are poor and 15 have diseases. An average US family earns 9.6 US cents, Oprah earns 523 US dollars; an average person in the world earns 1.3 US cents. An IT worker in the US earns 13 US cents, while one in India earns 2.5 US cents. Nike earns 36,505 US dollars, while a worker in its Vietnamese factory earns 0.14 US cents. 9 people are infected with HIV. 107 people died, of whom 18 died of hunger.	8 (0.39%)	Original Weibo post	Personal comment
五个家徒四壁的贵州孩子在垃圾桶中安静死去，广西 6 岁艾滋孤儿独自生活，本该撒娇无忧的年龄却背负重担；让我们帮帮他们，献一份爱心吧！ //@[user] 一支小铅笔也是一份爱心 转给那些有爱心不知道如何捐献的热心人士。行善，积福！	Five extremely poor kids died quietly in a rubbish bin in Guizhou. A 6-year-old orphan in Guangxi lives alone. At an age that should be spoiled and carefree, they live carrying heavy burden. Let us help them, donate your love! //@[user] A small pencil is love too Repost to those who have love in their heart but do not know how to donate. Practice charity, accumulate blessings!	4 (0.20%)	Original Weibo post	Personal comment
makiyo 在康熙说过只要杨千霈要的木有勾搭不上的，话说内谁还认识杨千霈！ //@[user1] 又是杨千霈又是闺蜜 //又是杨千霈 喝喝喝 //@[user2] 祝福劈腿的男男女女早日得艾滋 早死早超生 真应该一刀捅死劈腿狗男女再自杀啊	… Wish those guys and girls who cheated their partners, will soon get HIV… [This was actually a curse. Since the post was too vulgar, we did not translate the whole post.]	2 (0.10%)	Original Weibo post	Personal comment
**Others**		**614 (30.01%)**	These posts were predominately single Weibo posts that we did not group together as the aforementioned posts.	

* The category with an asterisk (*) means that those Weibo posts share the same meaning with some Weibo posts with some textual differences. The sample post presented is a representative one.

5. Hepatitis A

Figure S5. Daily count of Weibo posts with the keyword for "Hepatitis A" (甲肝) in 2012 among Sina Weibo users with 1000 followers or more.

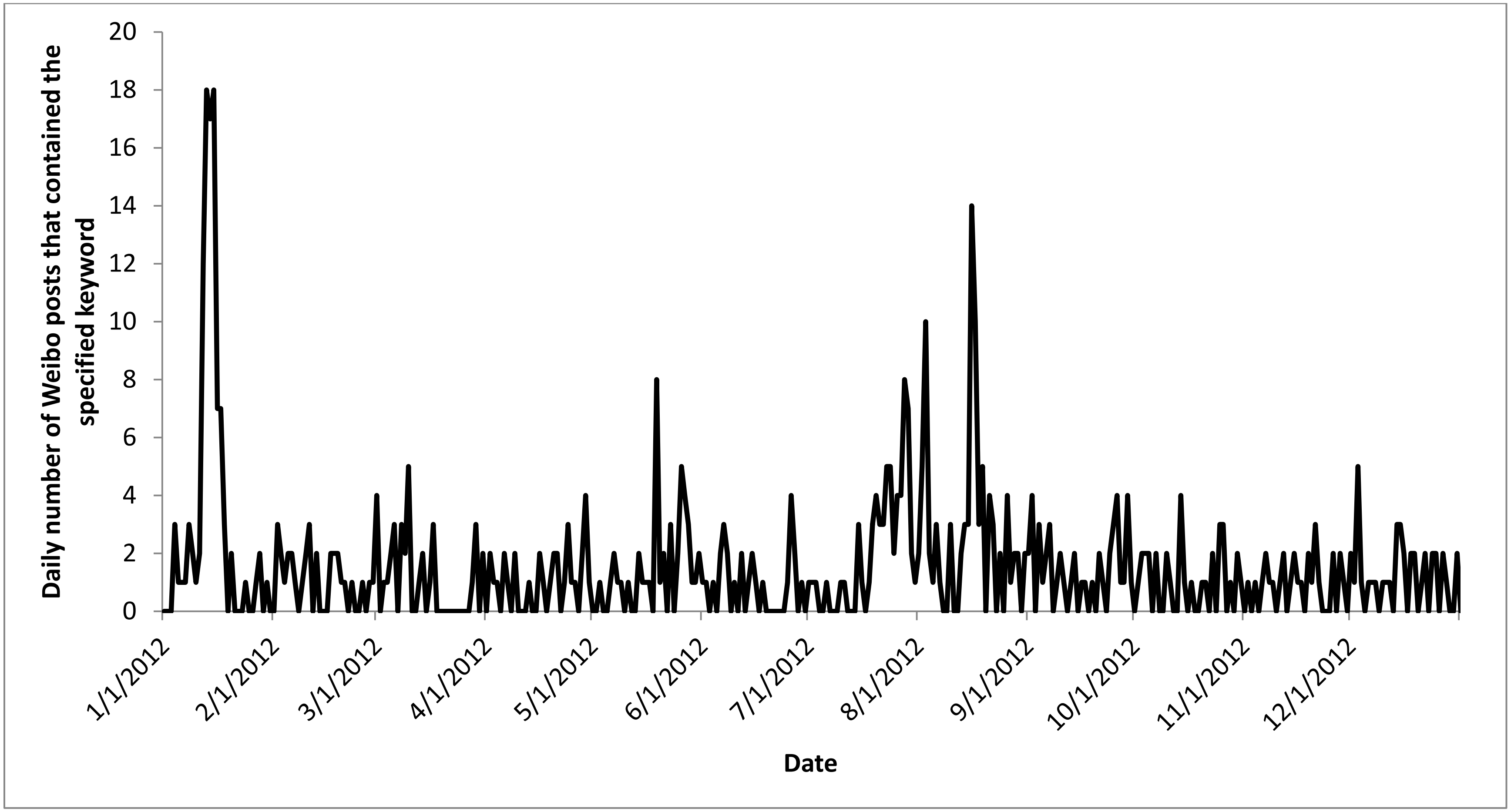

Content analysis was performed for Weibo posts generated for the two peaks date of January 13 and January 15, 2012.

Summary: We analyzed the Weibo posts for both January 13 and January 15, 2012. The vast majority of the posts in both days were reposts of a Weibo post titled “Food with a lot of germs”, that mentioned “Unclean scallions can lead to hepatitis A”. (See Wheeler et al. [3] for a hepatitis A outbreak associated with green onions in Pennsylvania, USA). We also found that the 17 posts mentioning “hepatitis A” on January 14, 2012 were all reposts of that post. Together, they formed one “peak” created by re-posting one post. And this post has been re-posted multiple times from early January to late 2012.

On January 13, 2012, there was a Weibo post that was re-posted twice (as observed in our dataset): “I said it once more, for the sake of the kids, domestic (i.e. China-made) products of calcium supplements with dairy ingredients, and cod liver oil, should not be given to the kids. Also, [even though] vaccine produced domestically is free, one should not take it, especially, the Encephalitis B vaccine and the Hepatitis A vaccine.” This post expressed the lack of consumer’s confidence in China-made health-related products. Two posts in our dataset on Jan 13, 2012 were re-posts of this post. Each of them included the microblogger’s own comments. One wrote, “What can we do? The humble poor people cannot afford imported products.” Another wrote, “For the sake of the kids, switch!” This is an example showing that the mistrust of certain healthcare products (including vaccines) by many Chinese citizens should be understood as part of a general mistrust of the quality control of Chinese products from infant formulas to vaccines.

Peak Date: January 13, 2012

Total count: 18

Table S5A. Sample Weibo entries that contain the keyword for “Hepatitis A” (甲肝) on July 13, 2012.

Sample Weibo posts on that day	English translation / description	Count (%)	Sources / References / Notes	Category
【细菌多的食物】1.哈密瓜：表皮裂缝有沙门氏菌。千万别买切开的哈密瓜。2.桃子：收获前会喷药。洗掉保护蜡或削皮。3.生鸡弯曲杆菌感染率 42%，易导胃肠炎，应先用水浸泡。4.沙门氏菌会在鸡蛋间传播，忌食未熟的蛋。5.不干净的大葱能导甲肝。6.牛肉馅中 30%含葡萄球菌.	Food with a lot of bacteria. 1. Hami melon [or cantaloupe]: The cracks on the surface trap Salmonella. Never buy Hami melons that have been cut open. 2. Peach: insecticide will sprayed before harvest. Wash away the protecting wax or peal the skin. 3. Live chicken: *Campylobacter* infection rate is 42%. It may easily lead to gastroenteritis. Should immerse it in water first. 4. *Salmonella* will spread among chicken eggs. Never eat uncooked eggs. 5. Unclean scallions can lead to hepatitis A. 6. 30% of ground beef contains Staphylococcus.	16(89%)	http://www.360doc.com/relevant/15091948_more.shtml	Health information
那咋办，屁民买不起进口的啊。//@[user]偶就再多次嘴，为了孩子。国产的含乳制品的钙类补充剂。鱼肝油，都不要给孩子用。还有疫苗，国产的免费，但真不能用，尤其是乙脑和甲肝疫苗。	What can we do? The humble poor people cannot afford imported products. //@[user] I said it once more, for the sake of the kids, domestic (i.e. China-made) products of calcium supplements with dairy ingredients, and cod liver oil, should not be given to the kids. Also, [even though] vaccine produced domestically is free, one should not take it,	2（11%）	Re-posts of an original post	Social issue / Personal comment (Concerns about the Quality of China-made

	especially, the Encephalitis B vaccine and the Hepatitis A vaccine.			Hepatitis A vaccine)

On Jan 14, 2012, all 17 posts with the keyword for "Hepatitis A" was reposts of the first sample Weibo post in the above table.

Peak Date: January 15, 2012

Total count: 18

Table S5B. Sample Weibo entries that contain the keyword for "Hepatitis A" (甲肝) on July 15, 2012.

Sample Weibo posts on that day	English translation / description	Count (%)	Sources / References / Notes	Category
【细菌多的食物】1.哈密瓜：表皮裂缝有沙门氏菌。千万别买切开的哈密瓜。2.桃子：收获前会喷药。洗掉保护蜡或削皮。3.生鸡弯曲杆菌感染率 42%，易导胃肠炎，应先用水浸泡。4.沙门氏菌会在鸡蛋间传播，忌食未熟的蛋。5.不干净的大葱能导甲肝。6.牛肉馅中 30%含葡萄球菌	Food with a lot of bacteria. 1. Hami melon [or cantaloupe]: The cracks on the surface trap Salmonella. Never buy Hami melons that have been cut open. 2. Peach: insecticide will sprayed before harvest. Wash away the protecting wax or peal the skin. 3. Live chicken: *Campylobacter* infection rate is 42%. It may easily lead to gastroenteritis. Should immerse it in water first. 4. *Salmonella* will spread among chicken eggs. Never eat uncooked eggs. 5. Unclean scallion can lead to hepatitis A. 6. 30% of ground beef contains Staphylococcus.	18(100%)	http://www.360doc.com/relevant/15091948_more.shtml	Health information

6. Hepatitis B

Figure S6. Daily count of Weibo posts with the keyword for “Hepatitis B” (乙肝) in 2012 among Sina Weibo users with 1000 followers or more.

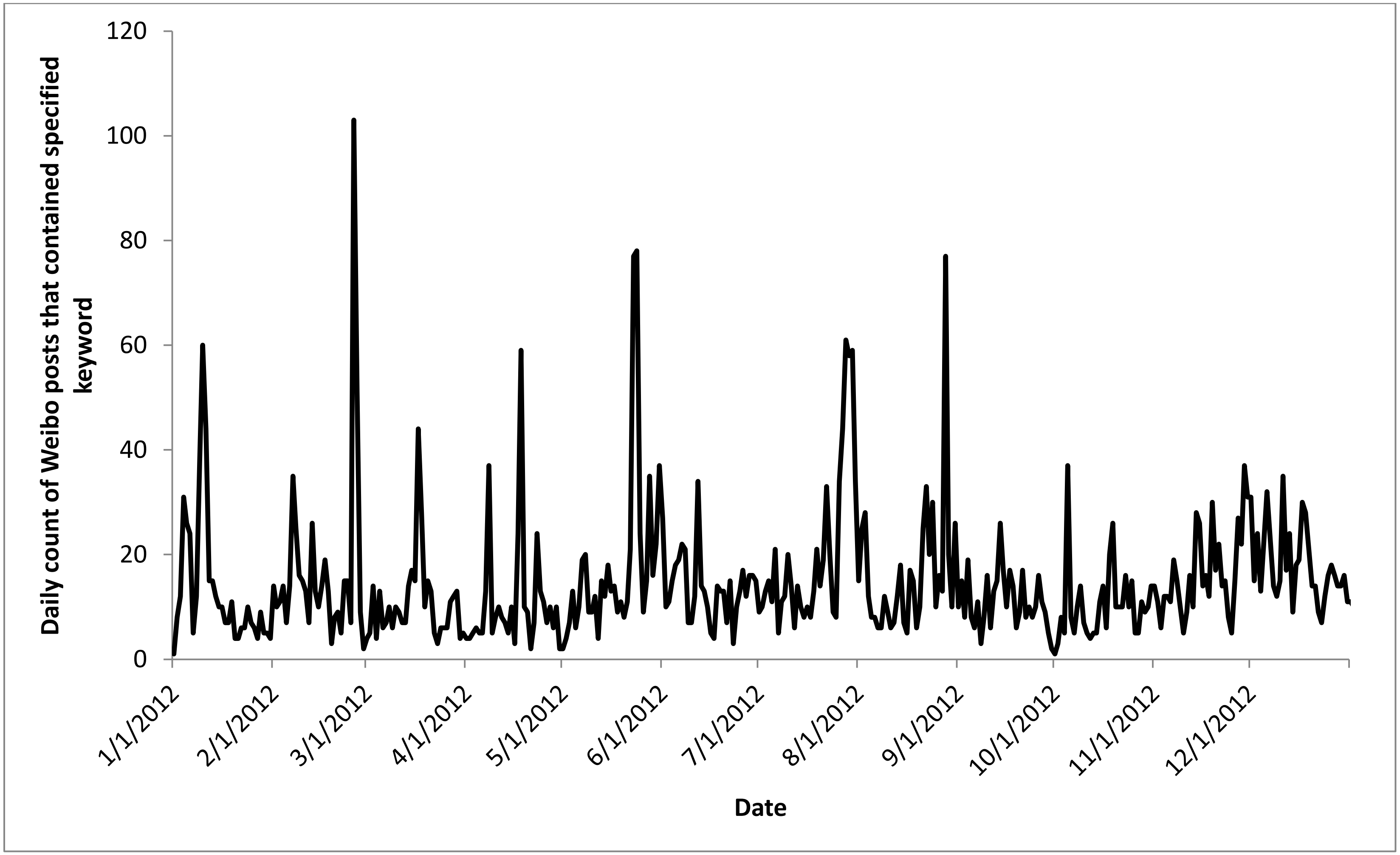

Content analysis was performed for Weibo posts of the peak date of February 26, 2012.

Peak Date: February 26, 2012

Total count: 103

Summary: Of the 103 Weibo posts mentioning "Yi-Gan" (乙肝) , which is the acronym for hepatitis B in Chinese, created on February 26, 2012, 99 (96%) were about advocating the elimination of discrimination against hepatitis B virus carriers in China. A young advocate, Mr. Chuang Lei (LEI, Chuang 雷闯), himself positive of hepatitis B virus, made petitions to the delegates of the Chinese People's Congress and the Chinese People's Political Consultative Conference (also known as *Lianghui*, the two Assemblies). Mr. Lei hoped that they can submit proposals to further eliminate discrimination against hepatitis B and fully ban advertisement about liver disease. Mr. Lei's Weibo posts received over 90 re-posts. Some simply re-posted it while some added their own comments. More importantly, Mr. Lei received a reply from a delegate (see below).

There was also a Weibo post (and one re-post) that provided a link to a Tudou (the Chinese equivalent of YouTube) video that was a news program. The first part of the news program was about a hepatitis B virus carrier trying to promote the knowledge of the proper routes of transmission of hepatitis B, by asking passersby to kiss her hand. She waited for 10 minutes before a passersby was willing to do so (Video: http://t.cn/zO2SiRy).

Table S6. Sample Weibo entries that contained the keyword for "Hepatitis B" (乙肝) on February 26, 2012.

Sample Weibo posts on that day	English translation / description	Count (%)	Sources / References / Notes	Category
**Petition to end discrimination against Hepatitis B carriers**		**101 (98%)**		
【恳请两会代表关注乙肝问题】尊敬的@[user] 委员 ，您好，我叫雷闯，上海交大研究生，一名乙肝病毒携带者，长期关注中国乙肝歧视问题。希望您能在两会递交#进一步消除乙肝歧视#和#全面禁止肝病广告#等建议，详见 http://t.cn/zObxzrO 。如果我能代表中国 1 亿乙肝病毒携带者，想对您说声谢谢。	"Plead *Lianghui* delegates to pay attention to the problem of hepatitis B" Dear @[user]: Delegate, hello. I am LEI Chuang, Shanghai Jiaotong University graduate student, a hepatitis B Virus carrier, who is a long-term observer of the problem of hepatitis B discrimination in China. Hope that you can submit to *Lianghui* the proposals #Further eliminate discrimination based on hepatitis B status# and #Full ban against hepatitis B advertisement#. For details, see http://t.cn/zObxzrO. If I can represent China's 100 million hepatitis B virus carriers, I would like to say thanks to you.	88 (85%)	Original Weibo post (and its re-posts)<br><br>The URL link in the Weibo post is equivalent to this URL link: http://blog.sina.com.cn/s/blog_5b359b300100yma3.html which is the full text of the petition.<br>Similar information written by Mr. Lei can also be found at: http://www.zhaoniupai.com/hbv/wap.asp?act=View&id=384	Petition to delegates at the Chinese People's Congress and the Chinese People's Political Consultative Conference (collectively known as *Lianghui*)
到时我会剪发的//@[user1]闯哥的头发是越发的长了，到时候跟总理吃饭的时候不扎起来的话会把头发弄到菜里去的。//@[user2] 希望您转发，帮乙肝病毒携带者	I would cut the hair then. //@[user1] Brother Chuang's hair is getting longer and longer. When you have meals with the Premier, your hair will touch your food, if you do not tie it up. //@[user2] Hope that you can repost it. Help HBV carriers to have their dreams come true.	2 (2%)	Original Weibo post<br>Note: In simplified Chinese, the word for posting and for hair is the same (发). Therefore, someone was making a joke that Mr. Lei's	Comments on Mr. Lei's petition

实现梦想			post had been reposted by many.	
回复@[user1]恩，那我其他啥事都不干了，呵呵 //@[user1]支持，如果每天寄的信是手写的就最好了！ //@[user2]希望您转发，帮乙肝病毒携带者实现梦想	Reply @[user1]Emm… Then I would not be able to do any other things. Huh. //@[user1]Support. If the letter sent every day is hand-written would be the best! //@[user2]Hope that you can repost it. Help HBV carriers to have their dreams come true.	1 (1%)	Original Weibo post	Reply to comments on Mr. Lei's petition
代表委员们，可愿代表下乙肝病毒携带者？	Delegates, are you willing to represent HBV carriers?	3 (3%)	Original Weibo post	Petition
这是我应该做的，能为进一步消除对乙肝的歧视做些事情，也是我的荣幸。 //@[user1]常接地气的人大代表，才会真有作为。 //@[user2]回复@[user3]只是尝试着联系您，没想到却得到您的支持，意外之喜，是感动，是收获。 //@[user3]支持。关注。递交。祝好。	This is what I ought to do. To be able to help further eliminate discrimination against Hepatitis B is my honor. //@[user1]Only the delegates to the Chinese People's Congress who regularly get in touch with the people can make a difference. //@[user2]Reply @[user3] I only tried to contact you. I never thought that I would be able to obtain your support. This is really a surprise. Very moving. What a gain. //@[user3] Support. Pay attention. Submit. Wish you well.	1 (1%)	Original Weibo post	Conversation with a delegate
关注乙肝问题，关爱他们。我朋友的一个同学就是上学后查出来乙肝，之后三年都没有跟任何同学朋友联系，最近才在 QQ 上见到，原来早就休学在家，而且得了自闭症。社会应该正确的对待他们，让他们可以正常生活，才会有勇气去面对自己的疾病。 //@[user] #恳请两会代表关注乙肝问题#	Concern about the problem of Hepatitis B. Care about them. My friend's classmate has been diagnosed with hepatitis B. After that for 3 years he was not in touch with any classmates. Recently we met again on QQ. It turns out that he has suspended classes and stayed home. He has also acquired autism. Society should treat them correctly, and let them live a normal life, so that they will have courage to face their own illness. //@[user]#Plead *Lianghui* delegates to concern about the problem of hepatitis B#	2 (2%)	Original Weibo post	Comments on Mr. Lei's petition
#乙肝病毒携带者可否当保姆？	Can hepatitis B virus carriers be babysitters?	1 (1%)	Original Weibo post	Original Weibo post
回复@[user] 第一：乙肝的传播途径是血液、母婴和未经安全防护的性接触传播，日常生活接触（共餐等）不会传播乙肝。第二：乙肝病毒携带者可以办健康证，可以从事食品、服务性行	Reply @[user] First, the routes of transmission of Hepatitis B are bloodborne, mother-to-child and through unprotected sexual intercourses. Daily routine contacts (such as having meals together) do not transmit hepatitis B. Second, hepatitis B virus carrier can carry "Health Card", and can work in catering and service industry. Third, the State (i.e. China) has not limited hepatitis B	1 (1%)	Original Weibo post	Original Weibo post

业。第三：国家并未限制乙肝病毒携带者从事家政行业。所以贵机构的行为，已经构成对乙肝病毒携带者的歧视。	carriers to engage in the industry of home economics. Therefore, the actions of your honorable institution have already discriminated against hepatitis B virus carriers.			
实拍乙肝女孩街头索吻 情人节情侣接吻比塞 http://t.cn/zO2SiRy	Real video of hepatitis B positive girl asking for kisses on the street; Valentine's Day couples kissing competition	2 (2%)	Link to news program: http://www.tudou.com/programs/view/6X6iZCXbkdM/dW5pb25faWQ9MTAwOTYzXzEwMDAwMV8wMV8wMQ	News about hepatitis B health education event
**Other Weibo posts**		**2 (2%)**		
#儿子来汉陷传销 父亲急得要跳桥#河南一大学生到武汉同学家里玩，却称患上乙肝让家里寄 15000 元。父亲放心不下，来汉寻找，却不见儿子踪影，发短信不回，打电话不接。 http://t.cn/zObweBN	# Son came to Wuhan and fell into a trap of pyramid scheme. Father was very anxious # A university student from Henan went to play at the home of a classmate in Wuhan. But he claimed that he had acquired hepatitis B and asked his family to send 15,000 yuan. The father was very concerned and went to Wuhan to look for his son, but he could not find him. He sent him text messages and there was no reply. He phoned him but no one picked up the phone. http://t.cn/zObweBN	1 (1%)	http://www.changjiangtimes.com/2012/02/375628.html	News
【谨记宝宝出生 6 件大事】1、第一声啼哭(肺部开始工作)；2、第一次吃奶(出生后半小时内，也许你只有几滴初乳，但对 BB 是最宝贵滴，且尽早吸吮，可促进乳汁分泌)；3、接种疫苗(出生后 24 小时内接种乙肝和卡介苗)；4 排绿黑色胎便(24 小时内)；5、生理性体重减轻(24 小时内)；6、脐带脱落(3~14 天)	[6 big events to remember since the birth of a baby] 1. The first cry (lungs start working). 2. The first breastfeeding (half an hour after birth, even though you may only have a few drops of colostrum, but these are very precious to the baby. Early feeding can also stimulate the secretion of breastmilk). 3. To receive vaccination (hepatitis B virus and BCG vaccine within 24 hours). 4. Physiological weight loss (within 24 hours). 6. The umbilical cord falls off (3 – 14 days).	1 (1%)	Original Weibo post	Health education about taking care of neonates

7. Hepatitis C

Figure S7. Daily count of Weibo posts with the keyword for "Hepatitis C" (丙肝) in 2012 among Sina Weibo users with 1000 followers or more.

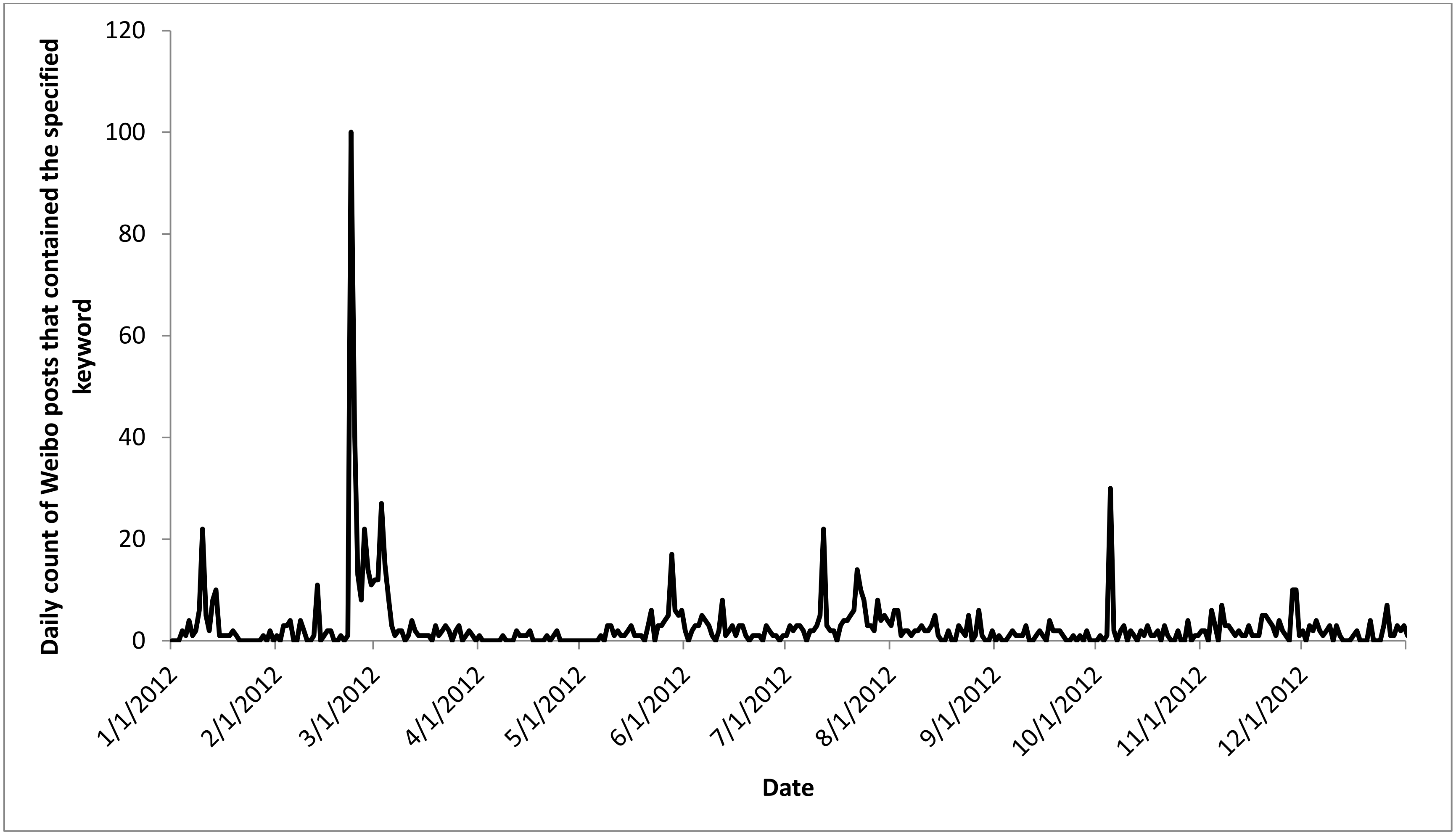

Content analysis was performed for Weibo posts on the peak date of February 23, 2012.

Peak Date: February 23, 2012
Total count: 100

Summary: An outbreak of hepatitis C has been happening for two years in Zijin County, Guangdong province. Nearly 200 people have been infected with hepatitis C virus. The suspected route of transmission is through repeated use of contaminated syringes at the health center in the township in that county. A

reporter of a newspaper in Guangzhou (the capital of Guangdong province) went there for interviews in December 2011. According to the reporter, personnel from the Communist Committee of Zijin County tried to offer bribes to cover it up. After being rejected by the reporter, they tried to block the article to appear in print via various means (see Weibo post in the Table below). Eventually, after two months, the news article finally went in print and the news was covered by major newspapers in China, including the People's Daily. Weibo posts of news articles about this outbreak were then re-posted by many (n=92).

Table S7. Sample Weibo entries that contained the keyword for "Hepatitis C" (丙肝) on February 23, 2012.

Sample Weibo posts on that day	English translation / description	Count (%)	Sources / References / Notes	Category
**News about an outbreak of hepatitis C infection in Zijin County, Guangdong Province, possibly due to repeated use of contaminated syringe**		**92 (92%)**		
This is one example out of many different Weibo posts generated by different news agencies and media:<br>【广东近 200 人疑因卫生所重复使用针筒感染丙肝】据人民日报报道，广东紫金县居民反映，家住该县响水路一带的近 200 名居民感染丙肝，原因不明。据称，感染者都曾到镇上卫生所打针，卫生所未采用一次性针筒。广东卫生厅已责成紫金县卫生局调查，广东医疗专家也正赶往紫金县参与调查。http://t.cn/zOUVCqH	This is one example out of many different Weibo posts generated by different news agencies and media:<br>【Nearly 200 people in Guangdong have been infected with hepatitis C, potentially due to repeated use of syringes】According to the People's Daily, residents in Zijin County, Guangdong, reported that nearly 200 people, who lived along Xiangshui Road in that county, have been infected with hepatitis C, through unknown routes of transmission. According to the report, all the patients who were infected have been vaccinated in the health center in the township. That health center has not used single-use syringe. Guangdong Department of Health has instructed the Zijin County Bureau of Health to investigate. Medical experts of Guangdong Province has also rushed towards Zijin County to participate in the investigation.<br>http://t.cn/zOUVCqH	92 (92%)	http://news.qq.com/a/20120223/000159.htm	News about a hepatitis C outbreak
**Personal comments on the outbreak**		**5 (5%)**		
埋了两个月，稿子终于见天。紫金丙肝疫情爆发两年，却一直被当地政府瞒报，对前往调查的专家进行阻挠。去年 12 月本人接到报料前往采访后，县委人员竟追来广州，欲重金收	Buried for two months, the article finally appears in print. The hepatitis C outbreak has taken place for two years in Zijin County. However, the local government has continuously covered it up and has prevented the experts who went there to perform their investigations. In December last year, after I	1 (1%)	Re-post of a personal Weibo post	Personal experience of reporting the outbreak

买。遭拒后，又通过其它种种渠道，阻挠报道见报。强烈要求惩治有关瞞报责任人。	received hints [from readers] and went there for interviews, personnel from the Communist Committee of the County dared to chase after me to Guangzhou. They wanted to pay me a huge sum to cover it up. After being rejected [by me], [they] tried to block the article to go in print, through various means. [I] strongly request [the authorities] to penalize those in authority who tried to cover up the outbreak.			
【日常生活中如何有效预防丙肝】近日广东近 200 人疑因卫生所重复使用针筒感染丙肝。丙型病毒性肝炎，是由丙型肝炎病毒(HCV)主要通过血液传播途径，少数通过密切接触传播途径所引起的急性肝脏炎症，临床表现与乙型肝炎相似。那么丙肝究竟是怎么传播的？我们又该如何预防呢？http://t.cn/zOUxUjA （康康）	"How to effectively prevent hepatitis C" Recently nearly 200 people in Guangdong have been infected with hepatitis C, potentially due to repeated use of syringes. Viral hepatitis C, is an acute viral hepatitis, caused by hepatitis C virus (HCV). Its major route of transmission is bloodborne, with a minority of transmission happens via close contacts. The clinical symptoms are similar to that of hepatitis B. So, how would hepatitis C be transmitted? How would we prevent it? http://t.cn/zOUxUjA (Kang Kang)	1 (1%)	http://health.sina.com.cn/d/2012-02-23/095223087.shtml	Health education
【美食中的丙肝"帮凶" !】河源市紫金县居民反映，响水路一带近 200 名居民感染丙肝，原因不明。这引起了社会的关注。丙肝患者除了定期复查，正确用药治疗外，日常生活应格外注意，有些禁忌一定不要触犯。究竟丙肝病人有哪些饮食禁忌？那些是美食中的丙肝"帮凶"？详情请戳：http://t.cn/zOUxnxd	""Accomplice" of hepatitis C among delicious food" Residents in Zijin County, Heyuan City, reported that nearly 200 residents along Xiangshui Road have been infected with hepatitis C. This attracts the attention of the society. Apart from regular check-ups and correct treatment with medication, hepatitis C patients should pay extra attention to certain dietary taboos that must not be committed. So, what are the dietary taboos for hepatitis C patients? What are the "accomplices" of hepatitis C among delicious food? For details, visit: http://t.cn/zOUxnxd	1 (1%)	Original link: http://group.gd.sina.com.cn/112517/thread-9476.html (no longer available; July 24, 2014)	Health education
广东河源离奇丙肝疫情爆发，本人于 2 月 2 日 00:13 写风水预测再次验证！（更早期的见博客）2012 年东南飞临玄空风水中最大凶星五黄。五黄主一切灾祸、病变。另外五黄遇原局飞星四绿，主传染病流行，或地震之发生！	Strange outbreak of hepatitis C at Guangdong Heyuan. My Fengshui prediction written at 00:13 on February 2 has been verified again! (For earlier predictions, see [my] blog.) In 2012, overcast the South-east Xuankong Fengshui was the largest malefic planet Wuhuang [i.e. Saturn]. Wuhuang is in charge of all disasters and epidemics. In addition, Wuhuang met Yuanju Feixing Xilü (i.e. planet	1 (1%)	Original Weibo post	Fengshui

	Jupiter), leading to the occurrence of epidemics or earthquakes.			
#我的乙肝也是因为共用针筒感染的# 广东紫金近 200 人感染丙肝 疑卫生所反复用针筒 http://t.cn/zOUxFG8	# My hepatitis B infection was due to sharing syringes# Guangdong Zijin nearly 200 people infected with Hepatitis C, potentially caused by repeated use of syringes in a health center.	1 (1%)	Original Weibo post	
**Other Weibo posts**		**3 (3%)**		
关注丙肝	Concern about Hepatitis C	1 (1%)	Original Weibo post	
肝脏沉默杀手——丙肝	Silent killer of the liver – Hepatitis C	1 (1%)	Original Weibo post	
【手腕上的紫色痒块】扁平苔藓是一种皮疹，由红紫色的顶部扁平的肿块组成，它有时候会让人痒到发疯。它经常出现在手腕或者膝盖上，但也可能在后背下方，脖子上，腿上以及生殖器上。诱因还不得而知，但如果你发现自己有，应该去检查肝脏，这可能是丙肝的征兆。	Lichen planus is a skin rash. It consists of purple-colored flat-topped papules. Sometimes it is itchy. It often appears on the waist or the knee. But it can also appear on the lower back, on the neck, on the leg and on the genitals. The cause is unknown. However, if you discover that you get it yourself, you should to have a liver check-up, as it may be a sign of hepatitis C.	1 (1%)	http://health.sohu.com/20110704/n312269697_4.shtml The original article at Sohu Health (dated July 4, 2011) was copied by many different Chinese websites and newspapers. According to Sohu Health, the article was edited and translated from WedMD, a medical website based in the USA.	Health education

8. Hepatitis E

Figure S8. Daily count of Weibo posts with the keyword for “Hepatitis E” (戊肝) in 2012 among Sina Weibo users with 1000 followers or more.

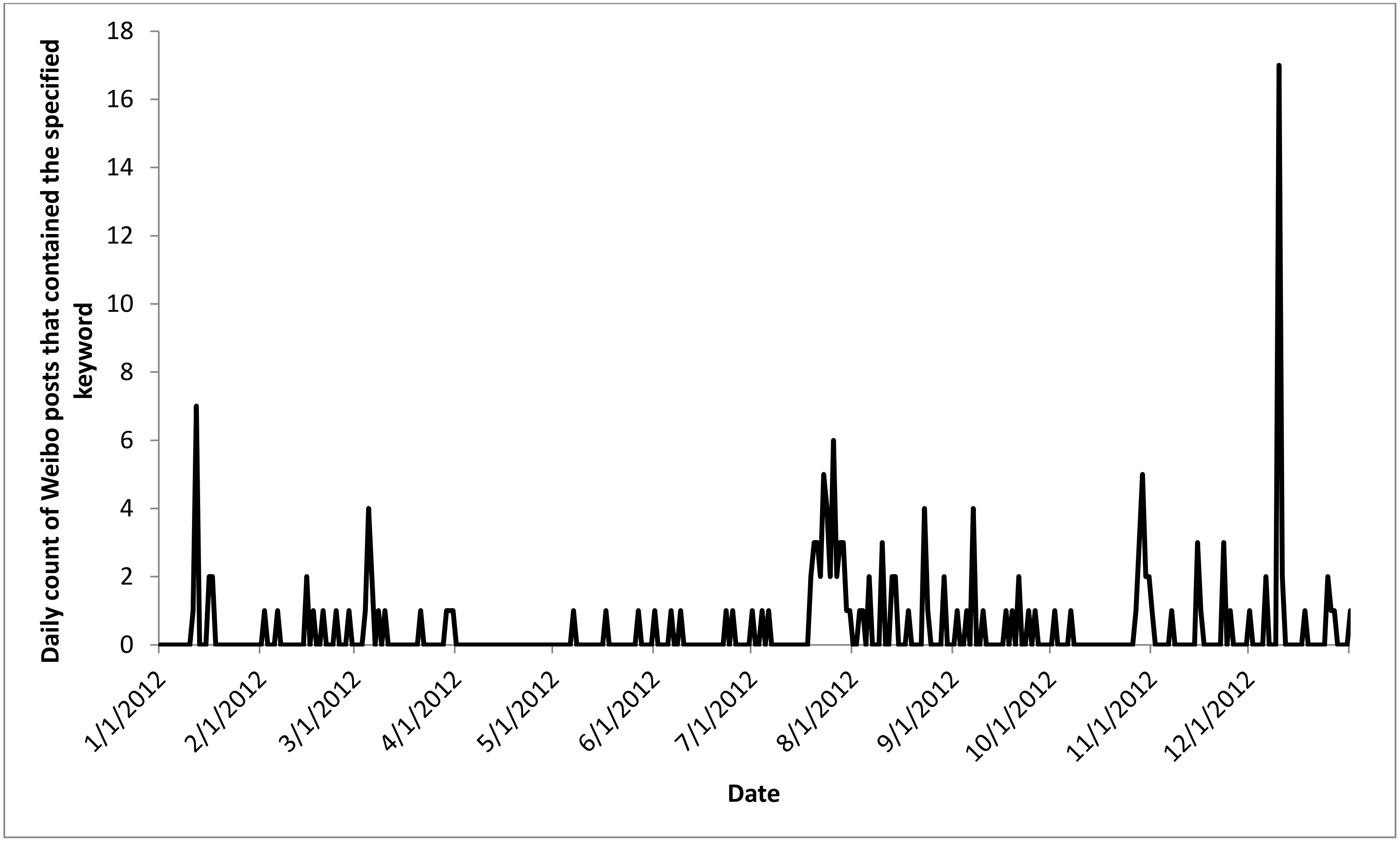

Content analysis was performed for Weibo posts generated on the peak date of December 10, 2012.

Peak Date: December 10, 2012

Total count: 17

On December 10, 2012, there were 17 Weibo posts that were related to Hepatitis E. Of these 17 posts, 14 were related to a conference held in Chongqing regarding the epidemiology and risk of Hepatitis E (including 6 posts that were news highlights). Experts from Chongqing municipal CDC highlighted the risk of hepatitis E. The other 3 posts were about the appearance in Nanchang, Jiangxi, the world's first hepatitis E vaccine, produced in China (reference for the vaccine, see: Soo Bin Park, 29 October 2012, http://www.nature.com/news/hepatitis-e-vaccine-debuts-1.11687).

Table S8. Sample Weibo entries that contained the keyword for "Hepatitis E" (戊肝) on December 10, 2012.

Sample Weibo posts on that day	English translation / description	Count (%)	Sources / References / Notes	Category
**Chongqing "Hepatitis E Epidemiology and Risk Conference"**		**14 (82%)**		**Health information**
【无证路边摊吃饭 当心感染戊肝】市疾控专家说，重庆人很喜欢吃路边美食，但那些大排档、小吃摊很多都是无证经营且卫生条件很差，很多面摊就在一盆"死"水里洗碗，根本不换，很容易传播戊肝病毒。戊肝发病率和病死率已居我国成年人急性病毒性肝炎首位。(重庆商报)	"Having a meal at unlicensed roadside food vendors Beware of being infected with Hepatitis E" Municipal CDC experts said, people at Chongqing like to eat delicious food along the roads. However, many of these big and small food vendors are unlicensed. Their sanitary conditions are very poor. Many noodle vendors wash the bowls in a single basin of "dead" water that is never changed. This will facilitate the transmission of Hepatitis E virus. The incidence and mortality rate of hepatitis E have ranked the highest among the various forms of acute viral hepatitis in our country (i.e. China). (Chongqing Shang Bao)	6 (35%)	http://e.chinacqsb.com/html/2012-12/10/content_313857.htm	Health information: News about the risk of Hepatitis E transmission
#健康警示#华龙网讯 昨日，由重庆市预防医学会主办的"戊肝流行病学与危害研讨会"在重庆市渝北举行。疾控专家表示，目前戊肝发病率和病死率已居我国成年人急性病毒性肝炎首位，对于喜欢吃路边美食的重庆人来说，预防这种肠道传染病的最好办法就是接种戊肝疫苗。	#Health warning# From Hualong Net. Yesterday, the "Hepatitis E Epidemiology and Risk Conference", organized by Chongqing Municipal Society of Preventive Medicine, took place at Yubei in Chongqing City. Disease control experts said, currently the incidence rate and mortality rate of hepatitis E rank top among acute viral hepatitis among adults in our country (i.e. China). To Chongqing people who like eating delicious food from roadside vendors, the best way to prevent this enteric infectious disease is to get vaccinated with hepatitis E vaccine.	2 (12%)	http://t.qq.com/clx1595548633?mode=0&id=178817086106709&pi=10&time=1355720381	Health information: News about the risk of Hepatitis E transmission
『重庆发生 ing』早安，这座城的早晨都由一条微博和两条大江开启。重	"Chongqing happening" Good morning. The morning of this city begins with one Weibo post and two big	6 (35%)	http://domestic.kankanews.com/c/2014-05-	Health information:

庆正在发生：1、蔬菜价同比涨 11.3%；2、养生会所做按摩 女子腰椎不幸被按裂；3、世界咖啡师比赛：冲咖啡动作也影响味道；4、重庆 13 条高速易起雾，已启动管制；5、无证路边摊吃饭 当心感染戊肝；http://t.cn/hawZc	rivers. What are happening in Chongqing …. 5. Having a meal at unlicensed roadside food vendors Beware of being infected with Hepatitis E …		06/0044481024.shtml	News highlight including about the risk of Hepatitis E transmission
**Hepatitis E vaccine**		**3 (18%)**		**Health information**
#报闻推介#【世界首支戊肝疫苗亮相南昌】由厦门大学国家传染病诊断试剂与疫苗工程技术研究中心研制的世界首支戊型肝炎疫苗益可宁，12 月 8 日，在南昌正式与我省疾控专家见面。市民可到疾控部门或有疫苗接种资质的卫生服务站接种。http://t.cn/zj6vJbk	#News in Newspaper – Recommendation# "The world's first hepatitis E vaccine appears in Nanchang" Hecolin (the world's first hepatitis E vaccine, developed by National Institute of Diagnostics and Vaccine Development in Infectious Diseases, at Xiamen University) made a formal appearance in Nanchang with our province's (i.e. Jiangxi) disease control experts, on December 8. Citizens can get vaccinated at the CDCs or health service centers that are qualified for the provision of vaccination.	3 (18%)	http://jiangxi.jxnews.com.cn/system/2012/12/10/012207201.shtml	News about the world's first Hepatitis E vaccine

9. Poliomyelitis

Figure S9. Daily count of Weibo posts with the keyword "Poliomyelitis" (脊髓灰质炎) in 2012 among Sina Weibo users with 1000 followers or more.

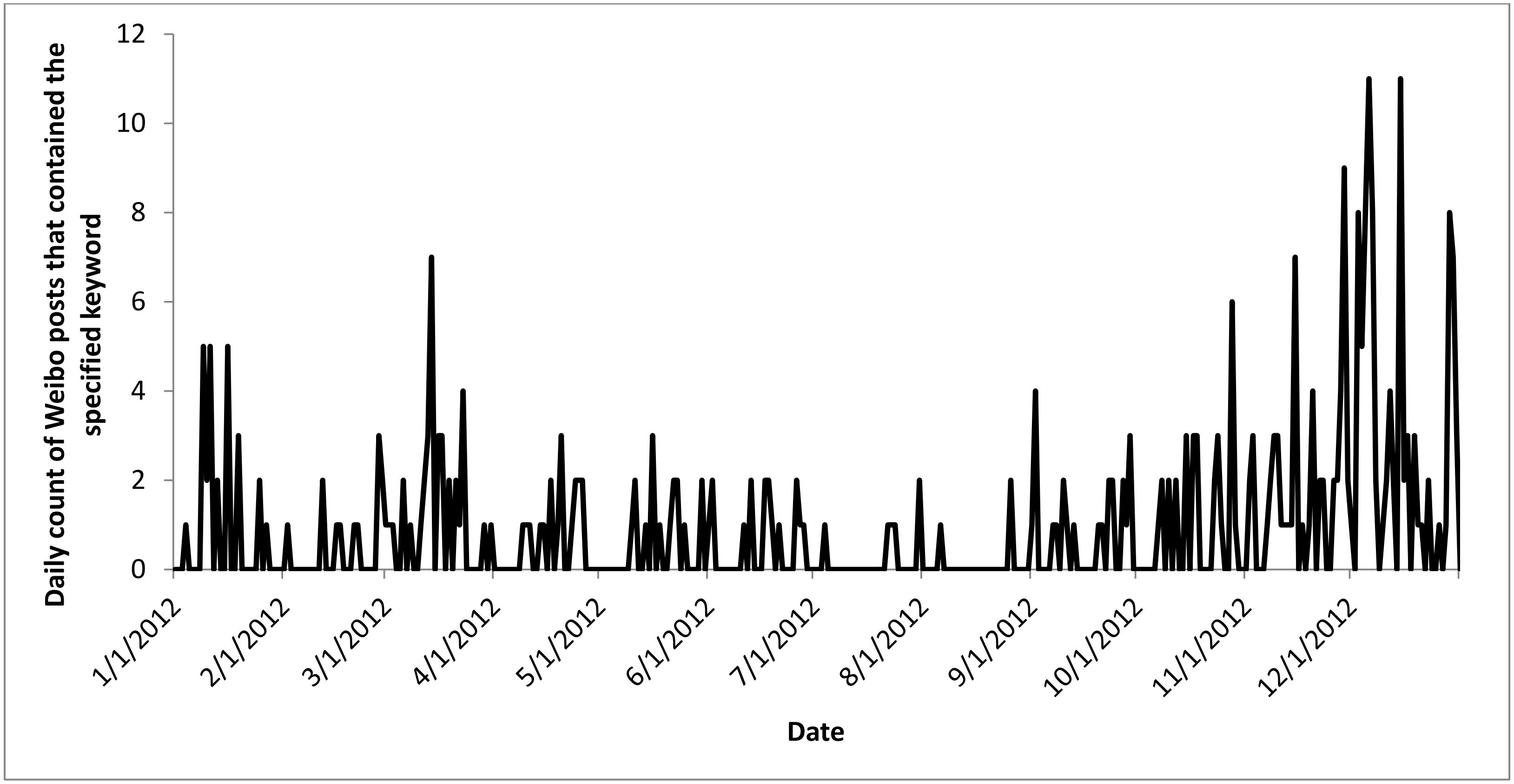

Content analysis was performed for Weibo posts generated on the peak dates of December 6 and December 15, 2012.

Peak Date: December 6, 2012
Total count: 11

Summary: Of the 11 Weibo posts mentioning "poliomyelitis" on December 6, 2012, five were related to a newspaper article about the National Immunization Day activities in the city of Shijiazhuang on December 5. Another five were personal conversation about polio vaccination for someone's baby. Another post was about the position of World Health Organization on polio vaccine.

Table S9A. Sample Weibo entries that contained the keyword for "Poliomyelitis" (脊髓灰质炎) on December 6, 2012.

Sample Weibo posts on that day	English translation / description	Count (%)	Sources / References / Notes	Category
**Health information about National Immunization Day**		**11 (100%)**		
#河青深读#【服糖丸没户籍限制 亲，不用扎堆排队】庄里的父母们，你家孩子服"糖丸"了吗？昨日，是石市适龄儿童免费口服脊髓灰质炎疫苗(糖丸)第一天，卫生服务站前排起长队。提醒您：服糖丸不受户籍地限制，家长拿着接种本可到全市任意接种点，不用在某个接种点扎堆排队。[呵呵]	#Hebei Youth Deep Reading# Take Sugar Pill not under *hukou* (family register) restriction Dear, don't need to line up in a long queue Parents in Shijiazhuang, have your children taken the "sugar pill"? Yesterday was the first day when children of the appropriate age in Shijiazhuang to take oral poliomyelitis vaccine ("sugar pill") for free. Long queue of people were lining up in front of health clinics. Reminder: To take the "sugar pills", there is no *hukou* (family register) restriction. Parents can take the vaccination records to any vaccination points in the whole city and need not line up in long queue at certain vaccination points.	5 (45%)	This was a Weibo post that linked to a news article on *Hebei Qingnian Bao*: http://t.cn/zjMEHX0	News article OPV vaccination day in a Chinese city
回复@[user1] 按照正常的接种程序，你宝宝应该在 4-6 岁时再加强一针脊髓灰质炎疫苗，按正常接种程序接种就行，没必要中间强化接种一次。 //@[user1] 4 剂次？我们只打过三次针呢。宝宝现在 10 个月。	Reply @[user1] According to the normal vaccination schedule, your baby should receive a booster dose of poliomyelitis vaccine around the age of 4-6 years. It is OK to follow the normal vaccination schedule. There is no need to receive a booster dose in between. //@[user1] 4 doses? We have only taken 3 doses. Baby is 10-month-old.	5 (45%)	Original Weibo post	Personal comments (vaccination schedule)
世界卫生组织的网站上对口服疫苗引起的脊髓灰质炎有介绍，WHO 的态度是，虽然有危险，但是这种情况极少发生，而且可	World Health Organization had introduced poliomyelitis derived from OPV in its website. WHO's attitude is this: even though there is a risk, but such situation rarely occurs. And this can be prevented with multiple rounds of high-quality	1 (10%)	WHO article: http://t.cn/zjxii2x http://www.who.int/features/qa/64/zh/	Links to WHO position on vaccine-derived polio

以通过多次高质量免疫避免，整体上仍然支持 OPV 的使用：http://t.cn/zjxii2x	immunization campaigns. Overall, WHO still supports the use of OPV: http://t.cn/zjxii2x			

Peak Date: December 15, 2012

Total count: 11

Summary: Of the 11 Weibo posts that mentioned "poliomyelitis" on December 15, 2012, seven mentioned a so-called "World Strengthened Immunity Day". What is written in Chinese as "Strengthened Immunity Day" is known as National Immunization Days (NIDs) or supplementary immunization activities (SIAs) in WHO's parlance. This is intended to supplement – not replace – routine immunization. National Immunization Days are conducted in two rounds, one month apart (see reference: http://www.polioeradication.org/Aboutus/Strategy/Supplementaryimmunization.aspx, accessed June 9, 2014). In mainland China, the National Immunization Day was conducted on December 5 and January 5.

While the National Immunization Days are part of WHO strategy to eradicate polio globally, there is no such event known as "World Strengthened Immunity Day" listed in the list of World Health Assembly mandated health days (reference: http://www.who.int/mediacentre/events/official_days/en/, accessed June 9, 2014). However, in Chinese mass media, as in the online discussion of Chinese netizens, there are many instances in which such an event "exists". For example, a 2013 newspaper article began: "Yesterday was the 'World Strengthened Immunity Day'" (Chun Cheng Wan Bao, reposted in Yunnan Net, December 16, 2013; http://society.yunnan.cn/html/2013-12/16/content_2998552.htm, accessed June 9, 2014). It cited a website for primary school education (http://www.xxkt.cn/yuwen/2006/7538.html, accessed June 9, 2014). Someone outside mainland China created a Facebook page for such an event (https://zh-cn.facebook.com/notes/%E8%90%A5%E5%85%BB%E5%85%8D%E7%96%AB%E5%AD%A6/%E4%B8%96%E7%95%8C%E5%BC%B7%E5%8C%96%E5%85%8D%E7%96%AB%E6%97%A5/664483666906915, accessed June 9, 2014). A health clinic in Beijing translated the information into English (http://www.hkclinic.com/en/edu_show.asp?id=180, accessed June 9, 2014). This so-called "World Strengthened Immunity Day" even has its own Baidu Baike (Baidu Encyclopedia) page, listing it as one of the "International Days" (http://baike.baidu.com/view/21469.htm, accessed June 9, 2014). No reliable sources were cited in any of these websites. While the 41st World Health Assembly (1988) has resolved to eradicate polio and national immunization days are one of the means to achieve that goal, the WHA did not specify December 15th as a so-called "World Strengthened Immunity Day" (See: http://www.polioeradication.org/content/publications/19880513_resolution.pdf, accessed June 10, 2014).

We can interpret this phenomenon as an interesting Chinese adaptation of NIDs and to promote it as an international day. By having it mis-understood / mis-interpreted (or even fabricated) as an international health day mandated by WHA, the Chinese authorities managed to convince the average Chinese citizen that the NIDs have international mandate (which they do have) and can encourage citizens to get vaccinated. We decided to classify these posts health information, instead of alternative health information.

Of the eleven Weibo posts, there were another four posts about the implementation of the National Immunization Day in a Chinese city.

Table S9B. Sample Weibo entries that contained the keyword for "Poliomyelitis" (脊髓灰质炎) on December 15, 2012.

**Sample Weibo posts on that day**	**English translation / description**	**Count (%)**	**Sources / References / Notes**	**Category**
**Health information about National Immunization Day**		**11 (100%)**		**Health information**
【世界强化免疫日】今天是世界强化免疫日，主要是为消灭脊髓灰质炎而设立的。强化免疫是消灭脊髓灰质炎的重要措施，中国共开展了三次六轮消灭脊髓灰质炎强化免疫日活动，即每年 12 月 5 日和 1 月 5 日对全国所有 4 岁以下儿童各服 1 剂脊髓灰质炎疫苗。让我们共同关爱儿童健康，强化人体免疫。	"World Strengthened Immunity Day" Today is World Strengthened Immunity Day [World Supplementary Vaccination Day / World Booster Dose Day]. The primary objective is to eradicate poliomyelitis. Strengthened immunity [supplementary vaccination?] is an important procedure to eradicate poliomyelitis. In total China has launched the activity of Eradicate Poliomyelitis World Strengthened Immunity Day 3 times (6 rounds). That is, every year, on December 5th and January 5th, provide every child under the age of 4 years one dose of polio vaccine. Let us take care of children's health, strengthen human immunity.	7 (64%)	The Baidu Baike (Baidu-Encyclopedia) has an entry http://baike.baidu.com/view/21469.htm but it does not cite any references. There are a number of Chinese websites that mention a so-called "World Strengthened Immunity Day" but never cited a source.	Health education (National Immuniza-tion Day)
记者从市疾控中心了解到，在强化免疫日期间，我市每年大概会发放 25 万粒脊髓灰质炎糖丸。孩子出生之后的两个月、三个月、四个月，都要服用一次这种脊髓灰质炎糖丸。	From the municipal CDC, journalists were made aware that during the "Strengthened Immunity Day" [i.e. National Immunization Day], our city [Qinhuangdao] will distribute 250,000 dose of poliomyelitis "sugar pill" [Oral Polio Vaccine]. After birth, at the age of 2nd, 3rd and 4th month, one has to take this type of poliomyelitis "sugar pill".	4 (36%)	http://www.weibo.com/1682305815/z9RRS8bMi	Health information (news)

10. Avian influenza

Figure S10. Daily count of Weibo posts with the keyword for "Avian influenza" (禽流感) in 2012 among Sina Weibo users with 1000 followers or more.

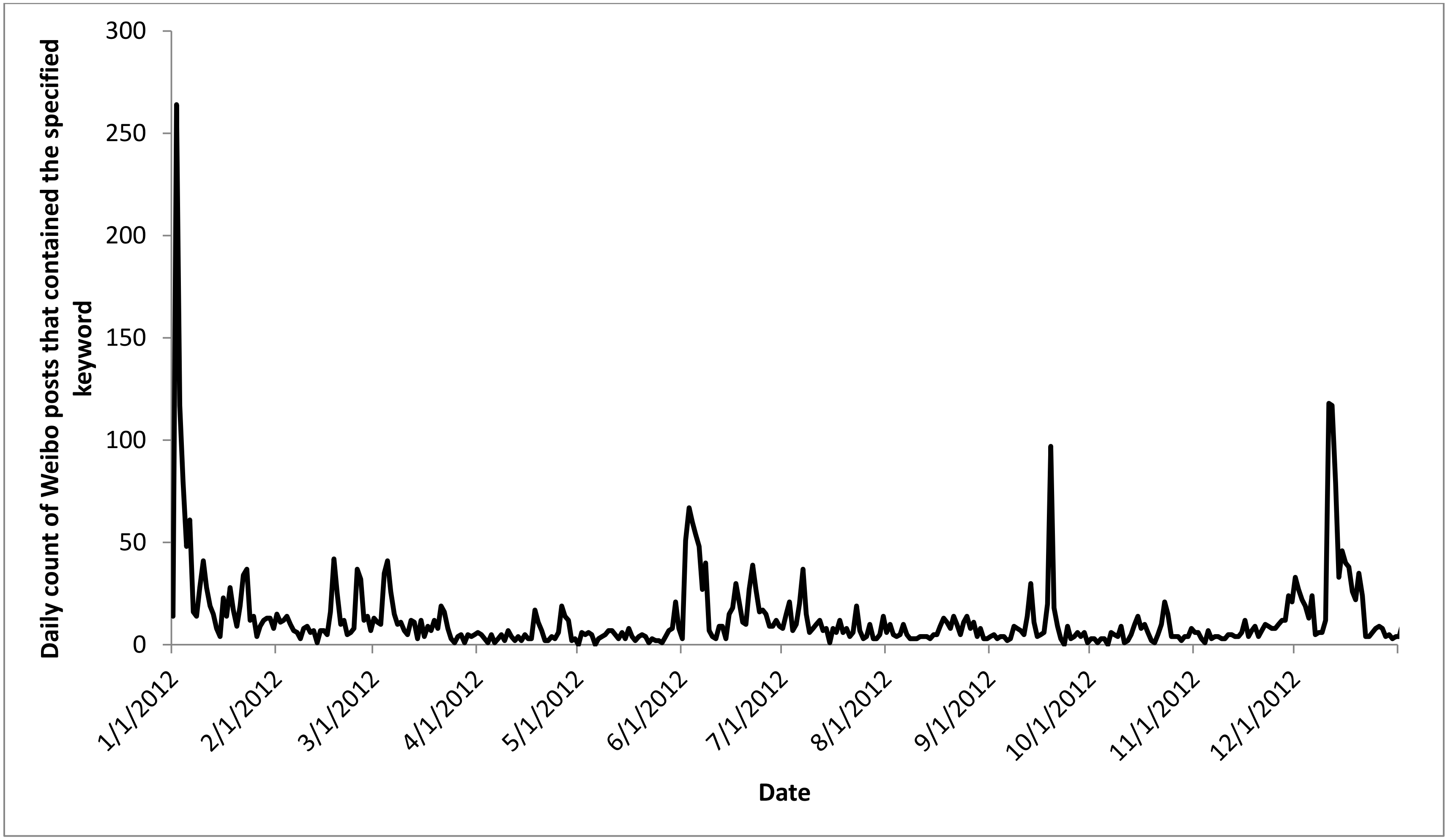

Content analysis was performed for Weibo posts generated on the peak day of January 2, 2012.

Peak Date: January 2, 2012.
Total count: 264

Summary: Of the 264 Weibo posts, carrying the keyword for “avian influenza” (禽流感), on January 2, 2012, 134 were news about a fatal case of human infection of avian influenza A(H5N1) in Shenzhen City and 6 posts were news about poultry culling and an import ban in neighboring Hong Kong.

Another 14 posts were news about poultry culling in a farm in Zengcheng, Guangzhou. The poultry there were not infected avian influenza, but “avian leukemia complicated with laryngotracheitis”.

29 posts were about side effects of Tamiflu. 3 posts were health education posts about avian influenza.

49 posts were re-posts or variations of a post that had been widely circulated in China that expressed the feeling of certain young Chinese who were born in the 1990s about the various epidemics and natural disasters that they have encountered in their lives.

26 posts were original Weibo posts expressing comments about avian influenza; one post was comment about avian leukemia. 2 other posts were about other news that somehow mentioning avian flu.

Paying closer attention to these comments, we can have a glimpse of a variety of online reaction towards the avian influenza incident. One Weibo user recommended “eating less chicken and ducks; also not having meals at Kentucky Fried Chicken.” (“少吃鸡鸭吧，肯德基也别吃了”). One user asked, “There is someone who died of complications of avian influenza in Shenzhen. [China] CDC said that the virus has not mutated to become transmissible between humans. Should we believe in what you [China CDC] say?” (“深圳有人因禽流感引發併發症死亡，疾控中心說病毒沒變種至人傳人。究竟信不信你好？”) One hoped that it would end soon (“希望到此为止”); another was worried (“刚刚新闻说深圳有禽流感，担心。”); and someone asked, “What good, advanced and wonderful ways to prevent avian flu do you have?” (“防治禽流感，您有什么好招、高招和妙招？”). But there was also a user who was not worried: “It was really cold tonight. I have just had a meal of wild game … [a list of dishes]… It was really good. Who care about SARS and avian flu? Let’s satisfy the appetite first!” (“今晚夠冷，吃了頓野味宴，有炆菓子狸，山豬，炒黃猄肉，燒大鱔，班鳩鯪魚肉飯，飯鑊頭蛇湯，黃金蝦，各類有機菜，真是正絶，什麼沙士禽流感均拋諸腦外，大快朶頤才算！”)

Table S10. Sample Weibo entries that contained the keyword for "Avian influenza" (禽流感) on January 2, 2012.

Sample Weibo posts on that day	English translation / description	Count (%)	Sources / References / Notes	Category
**A fatal case of human infection of avian influenza H5N1 in Shenzhen***				
【深圳禽流感死亡患者毒株确定不能在人与人之间传播】 深圳市疾控中心 2012 年 1 月 1 日晚通报确定患者感染毒株，属 2.3.2.1 亚系的高致病性禽流感病毒，受体依然为禽类受体，依旧不能在人与人之间进行传播，广大市民无需恐慌。	[The avian influenza strain of the dead patient in Shenzhen has been confirmed not transmissible between humans] In the evening of January 1, 2012, Shenzhen Municipal CDC reported the strain that infects the patient belongs to a highly pathogenic avian influenza subtype 2.3.2.1. The receptor remains avian receptors. The virus remains non-transmissible between humans. There is no need for the citizens to panic.	134 (51%)	http://news.163.com/12/0102/04/7MO2J01N00014AED.html	News about a case
**News about Hong Kong poultry culling and import ban***				
路透社报道，香港日前屠宰了 19000 只家禽，并禁止进口和销售，只因发现两只感染了禽流感。邻接的深圳有 39 岁陈姓公车司机近日去世，经检死于禽流感，此病死亡率 60%。该男子 21 日发烧，圣诞节送医院，当地官员称与他亲密接触的 120 人被测无恙。据称，卫生部已将此事照会世界卫生组织。	Reuters reported that recently Hong Kong had culled 19,000 poultry and banned imports and sales, even though only two poultry were infected with avian influenza. Neighboring Shenzhen, a 39-year-old bus driver, Mr. Chen, passed away. After autopsy, he was found to be dead of avian influenza. This disease has a case-fatality ratio of 60%. That man had a fever on the 21st, was taken to the hospital on Christmas Day. Officials there said that all the 120 people who were in close with him were not found ill. It was reported that the Ministry of Health had presented a note about this incident to the World Health Organization.	6 (2%)	http://t.cn/SJdc5J	News about poultry import ban
**News about culling chicken in Guangzhou (avian leukemia)**				
#广州增城近 3 万只鸡染病被扑杀#去年 12 月 28 日，广州增城一家养鸡场的鸡只集体染病，全场鸡只被集体扑杀；昨日广州市农业局表示，该养鸡场鸡只所感染的是"禽白血病并发喉气管炎"，并非高致	#Nearly 30,000 chicken were infected and were culled in Zengcheng, Guangzhou.# On December 28, last year, chicken in a chicken farm in Zengchang, Guangzhou, fell ill collectively, and all the chicken were culled. Yesterday, Guangzhou Municipal Bureau of Agriculture said that, the chicken in that chicken	14 (5%)	http://www.chinanews.com/gn/2012/01-02/3577430.shtml	News about culling chicken in a farm (confirmed not avian

病性禽流感。为防止疫病扩散，增城市农业局和动检机构于去年 12 月 31 日将全场 2.75 万只鸡扑杀。CNTV 陈柏屹编辑报道	farm was infected with “avian leukemia complicated with laryngotracheitis”, and not the highly pathogenic avian influenza. In order to prevent the epidemic from spreading, Zengcheng Municipal Bureau of Agriculture and animal health inspection authorities culled all the 27,500 chicken in the farm on December 31. Edited and reported by CNTV Chen Baiyi			influenza)
**News about Tamiflu**				
【达菲被指有强副作用严重可致死】达菲（又名奥司他韦）是由瑞士罗氏制药公司研制的抗流感药物，近年来在抗禽流感和甲型 H1N1 流感时广泛使用。日本和美国的药品安全部门已警告，达菲可导致精神异常、产生幻觉等多种副作用，甚至造成死亡。	Tamiflu (also known as Oseltamivir) is an anti-influenza drug developed by Roche, a Swiss pharmaceutical company. In recent years, it has been widely used to combat against avian influenza and influenza A(H1N1). The drug safety authorities of Japan and the US have warned that Tamiflu can lead to side effects such as mental disorder and hallucinations, and even lead to death.	29 (11%)	http://news.qq.com/a/20120222/000830.htm	
**Health education (avian flu)**				
【12580 营养百科：健康说客】禽流感，再来袭，预防措施要做足：远离活禽，勿让病毒黏上您；勤洗双手，切断传播途径；加强锻炼，抵抗病毒来侵袭！	“12580 Nutrition Encyclopedia: Health lobbyists” Avian flu attacks again, preventive measures should be well adopted: stay away from live poultry, don’t let virus stick to you; wash both hands diligently, cut off all transmission routes; Do more exercises, to withstand the onslaught of the virus!	3 (1%)	Original Weibo post	Health Education
**Personal comments**				
活了一二十年，碰上了 50 年一遇的旱灾，60 年一遇的雪灾，100 年一遇的洪涝，300 年一遇的泥石流，500 年一遇的地震，600 年一遇的海啸，还有什么日食月食流星雨…迎着非典上小学，踏着禽流感上初中，冒着甲流上高中，现在上大学了，又说 2012 世界末日！我们这辈子也算活得轰轰烈烈了 ！	I have lived 20 years, having experienced a drought that was 1 in 50 years, a snow storm that was 1 in 60 years, a flood that was 1 in 100 years, a mudslide that was 1 in 300 years, an earthquake that was 1 in 500 years, a tsunami that was 1 in 600 years, and also solar eclipses, lunar eclipses and meteors…. Facing SARS, I attended primary school, stepping on avian flu, I attended lower middle school; taking the risk of influenza A, I attended upper middle school; now I attend university, someone said that 2012 is the end of the world! What a spectacular lives we have…	49 (19%)	http://m.3g.qq.com/wbread/topic/rt?tn=500%E5%B9%B4%E4%B8%80%E9%81%87%E7%9A%84%E5%9C%B0%E9%9C%87	Personal comments
禽流感，用雞要小心！！！唔好接	Avian flu, caution when handling chicken!!! Don’t	26	26 different original Weibo posts	Personal

觸到便便！	touch the feces!	(10%)	about avian flu	comments about avian flu
第一次知道鸡会得这些似乎只有人才会得的病… //@[user] 白血病加气管炎？！ 听起来比禽流感可怕多了。	The first time to learn that chicken will get these diseases that seem to me that only people will get… //@[user]leukemia plus tracheitis?! Sounds more dreadful than avian flu.	1 (0.4%)	Original Weibo post	Personal comments about avian leukemia
**Others**		2 (1%)		Others (news unrelated to avian flu)

* Some Weibo posts mentioned both the case in Shenzhen and the chicken culling and import ban of Hong Kong. The first author (ICHF) made the decision which news was the main message of that Weibo post and categorized accordingly.

11. Influenza A(H1N1)

Figure S11. Daily count of Weibo posts with the keyword "H1N1" in 2012 among Sina Weibo users with 1000 followers or more.

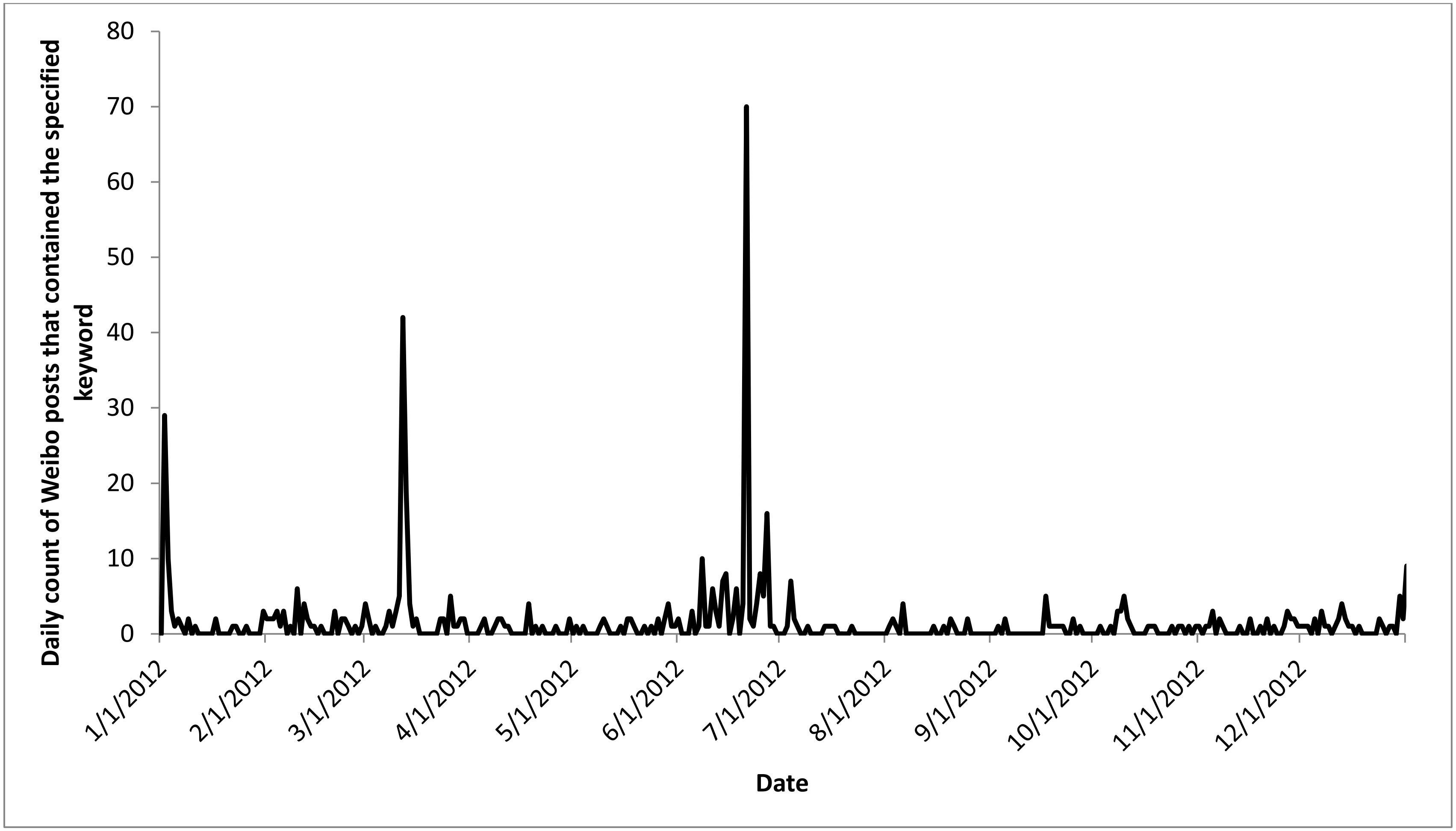

Content analysis was performed for Weibo posts that were generated on March 12, 2012 and June 21, 2012.

“False Positive” Peak Date: June 21, 2012

Total count: 70

Of the 70 posts on the peak date of June 21, 2012, 67 (96%) were “false positives” as they were counted due to the pseudonym @u3JH1N1HY (the random number generated to replace a username) contained the numeral-letter combination of “H1N1”. Therefore, we did not count it as a real peak and we moved on to analyze the data on March 12, 2012.

Peak Date: March 12, 2012

Total count: 42

Of the 42 Weibo posts mentioning “H1N1” that were generated on March 12, 2012, 37 posts were about the outbreak of influenza A(H1N1) among students in Hotan, Xinjiang. There were news reports (n=29), health education materials (n=5) and personal comments (n=3).

Table S11. Sample Weibo entries that contained the keyword “H1N1” on March 12, 2012.

Sample Weibo posts on that day	English translation / description	Count (%)	Sources / References / Notes	Category
**H1N1 outbreak in Hotan, Xinjiang**		**37 (88%)**		
【新疆和田多名中小学生确诊为甲型 H1N1 流感】新华网消息，记者 11 日从新疆有关部门获悉，因和田地区相继出现多名中小学生被确诊为甲型 H1N1 流感，为有效控制疫情在各级各类学校的蔓延，新疆从即日起在中小学和幼儿园恢复晨检制度。	“Many secondary and primary schools students were confirmed of influenza A (H1N1) infection in Hotan, Xinjiang” From Xinhua Net. On the 11th, journalists learnt from the responsible authorities in Xinjiang, because many secondary and primary school students were confirmed of influenza A(H1N1) infection in Hotan, in order to effectively control the epidemic to spread in different levels and different types of schools, the morning inspection system has to be resumed today in secondary and primary schools and kindergartens across Xinjiang.	29 (70%)	News from Xinhua Net (March 11, 2012): http://news.xinhuanet.com/local/2012-03/11/c_111637153.htm	News about an outbreak of influenza A(H1N1) in Hotan, Xinjiang
【甲型 H1N1 流感防护手册】春季为呼吸道传染病甲型 H1N1 流感高发期，须及时防护。那么什么是甲型 H1N1 流感？此型流感有哪些症状？哪些人容易被传染？怎样做好防护？它是通过哪些途径传播的？详情请见甲型 H1N1 流感防护手册。	“Influenza A(H1N1) protection handbook” Spring is the season when the incidence of respiratory infection influenza A(H1N1) is high. We should protect ourselves in time. So, what is influenza A(H1N1)? What type of symptoms will this type of influenza lead to? Who will be susceptible? How would we protect ourselves? Through which routes will it spread? For details, please read the Influenza A(H1N1) protection handbook.	5 (12%)	This is a Weibo post release by Sina Health to promote its health education website on influenza A(H1N1) http://health.sina.com.cn/z/H1N1fangzhi/index.shtml	Health education

http://t.cn/zOfjMRQ via.@新浪健康	http://t.cn/zOfjMRQ via @Sina Health			
额，H1N1 又来了？	Huh, H1N1 comes again?	2 (4%)	Original Weibo post	Personal comment
H1N1 引发的医药股行情 。。sort of ironic?	The market trends of the pharmaceutical industry triggered by H1N1 … sort of ironic?	1 (2%)	Original Weibo post	Personal comment
**A journalist's reminiscence of his reporting of the 2009 pandemic**		**5 (12%)**		
当记者是应该好好学学这一招，丁老师厉害~！//@[user]好习惯。多年前在南方周末做 H1N1 猪流感报道时，在某省卫生厅垃圾桶翻到了被丢弃的大部分内部汇报材料。没有翻垃圾桶绝技的记者不是好调查记者。	Journalists should learn this skill. Teacher Ding, bravo! //@[user]Very accustomed. Many years ago when I was reporting the H1N1 swine flu at the *Southern Weekend*, I picked up the majority of the internal reports that had been thrown away in the rubbish bin of the Department of Health of a certain province. A journalist without the wonderful skill of turning over the rubbish bin is not a good investigative journalist.	5 (12%)	http://t.caijing.com.cn/album/reply?tid=636053	Personal reminiscence of the reporting experience during 2009 H1N1 pandemic

12. Measles

Figure S12. Daily count of Weibo posts with the keyword for "Measles" ("麻疹") in 2012 among Sina Weibo users with 1000 followers or more.

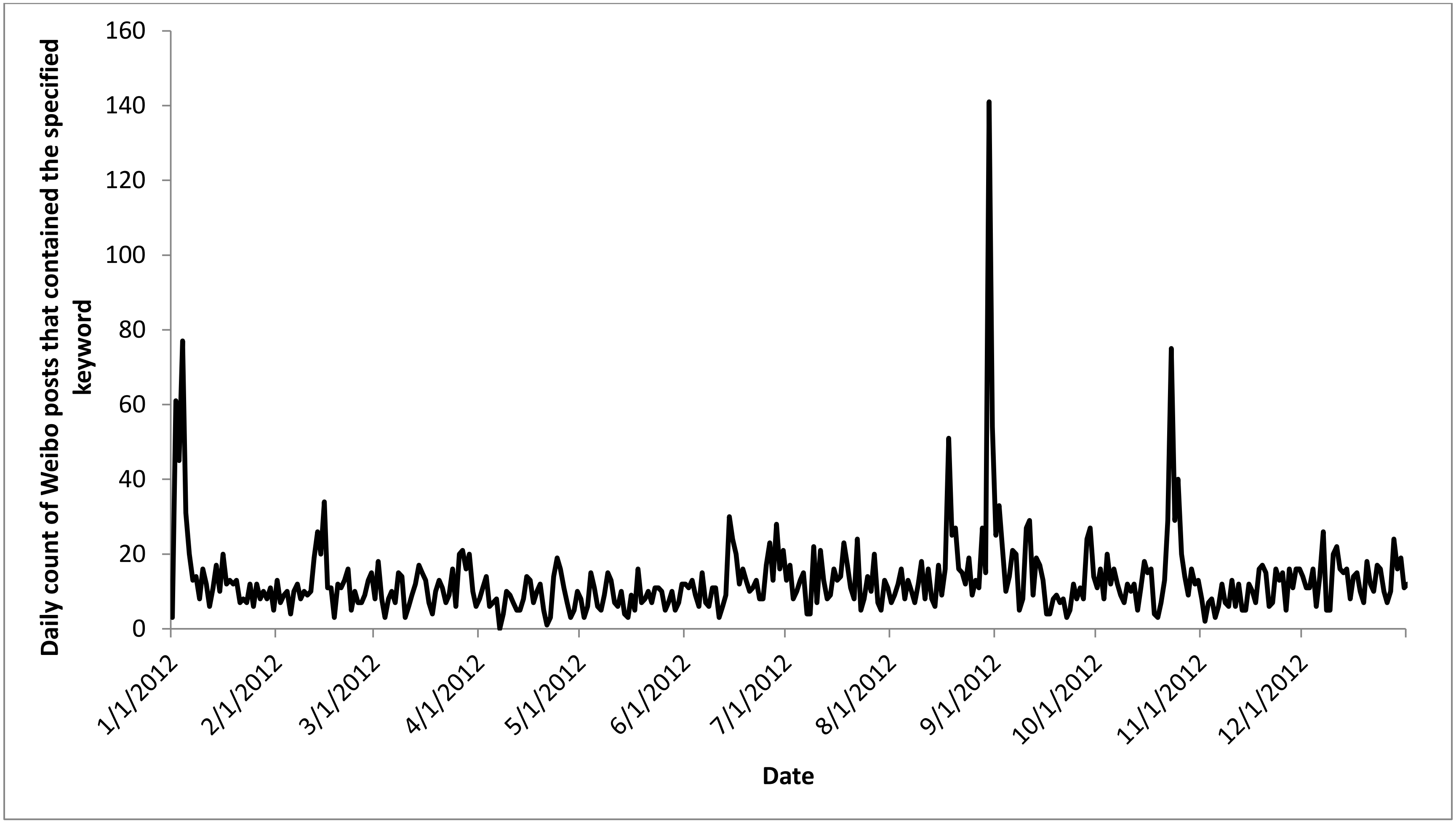

Content analysis was performed for Weibo posts generated on the peak dates of January 4 and August 30, 2012.

"False positive" Peak Date: August 30, 2012.

Total count: 141

All 141 Weibo posts on August 30, 2012, that contained the term "measles" ("麻疹") were actually about urticaria (commonly known as hives) and not about measles. In Chinese, urticaria is known as "荨麻疹". The Chinese medical term for urticaria "荨麻疹" contains "麻疹" (measles) as its second and third characters.

Therefore, we moved on to analyze the peak on January 4, 2012.

Peak Date: January 4, 2012.
Total count: 77

Summary: There were 77 Weibo posts that mentioned "measles" (麻疹) on January 4, 2012. Of them, 68 were posts and re-posts of health information provided by a commercial company that mentioned four diseases that have symptoms similar to common cold. Another 5 Weibo posts were related to alternative health information (n=3), health information (n=2) and traditional Chinese medicine (n=1).

There were 4 posts that were related to urticaria, not measles.

Table S12. Sample Weibo entries that contained the keyword for "Measles" (麻疹) on January 4, 2012.

Sample Weibo posts on that day	English translation / description	Count (%)	Sources / References / Notes	Category
【四种"假感冒"要当心"】治病最怕误诊，有四种病与感冒的症状类似，千万别大意。1、水痘：身上会出水泡；2、麻疹：面颊上会出现红点，红点的中心是白色或蓝色，之后向全身蔓延；3、脑膜炎：手足剧烈疼痛、高烧、呕吐、难以唤醒；4、腮腺炎：低烧、头痛、两腮肿起。	"Beware of four types of "false common cold"." Misdiagnosis is the worst when it comes to treating illness. There are four illnesses that have symptoms to common cold. Do not be careless. 1. Chicken pox: blisters on the body; 2. Measles: red dots will appear on cheeks. The center of the red dots is white or blue. Later, they spread across the whole body; 3. Meningitis: severe pain at the extremities, high fever, vomiting, difficult to wake up; 4. Mumps: low fever, headache, swollen cheeks.	68 (88%) *	This piece of information was reposted in a variety of platforms. One recent example is: http://www.eyoogo.com/info-2582.html	Health information
【看自己的皮肤检测自己的健康】1、手掌和脚面发黄，可能是甲状腺机能减退；2、日晒现麻疹可能吃了光敏药物；3、掌纹颜色加深可能肾上腺素分泌不足。4、腿部大片青色肿块或是静脉曲张。5、胫骨部现褐色斑点或是糖	"Look at your own skin to inspect your own health" 1. If the pale and sole appear yellowish, it may be hypothyroidism. 2. Under sunlight, the appearance of measles [urticaria?] may be a result of taking a photosensitive drug. 3. The color of palm print has darkened may be a result of inadequate secretion of adrenaline. 4. Large blue lumps in the leg may be a sign of varicose. 6. Tingling sensation and rash appear	3 (4%)	Example: A popular webpage that carries similar information: http://health.ifeng.com/disease/skin/detail_2011_01/17/4308879_0.shtml	Alternative health information

尿病；6、皮肤麻刺感并出现皮疹可能是带状疱疹	in the skin may be a sign of shingles.			
#2012 健康月历，祝您月月吉祥，岁岁平安！# 3 月份 春暖花开病菌多 ☆气候特点：气温逐渐回升，乍暖还寒，回潮明显。 ☆高发疾病：流感、麻疹、风疹、肝炎、湿疹、过敏性鼻炎、支气管哮喘、风湿性关节炎。 ☆健康提点：1、气温逐渐回升，病菌大量繁殖，注意防范甲流、麻疹、风疹等传染病。	#2012 Health Calendar. We wish that every month is auspicious to you and peace to you every year! #March Warm spring with blossoming flowers and a lot of germs ☆Climatic characteristics: The temperature gradually increases. It is getting warm but still there are episodes of cold weather. Apparently it is getting more humid. ☆Diseases of high incidence: influenza, measles, rubella, hepatitis, eczema, allergic rhinitis, bronchial asthma, rheumatoid arthritis. ☆Health reminder: 1. The temperature gradually increases. Germs greatly multiply. Pay attention to prevent infectious diseases, such as influenza A, measles and rubella.	1 (1%)	Original Weibo post	Health information
板蓝根有清热解毒等功效，对感冒、流感、腮腺炎、红眼病、麻疹等常见病，有较好的预防和治疗作用。但是，少数人以为板蓝根冲剂是良性药，不管有病没病都要来两包，其实这是不对的。 http://t.cn/SiYJHA	Indigowoad root has the effects of detoxification. It has a better preventive and treatment effects on common diseases such as cold, flu, mumps, conjunctivitis, and measles. However, a minority of people think that indigowoad root powder is a “benign drug”. Whether one is ill or not one would take two packets. This is not correct.	1 (1%)	The URL link in the Weibo post leads to this article: http://www.jysq.net/article-26730-1.html	Traditional Chinese medicine
**Posts about Urticaria**		**4 (5%)**	4 different posts about urticaria	Not applied

* One of the reposts was a modification of the original post with the last section about “mumps” deleted. That explains why the count of this Weibo post and re-posts was 68 for “measles” and was 67 in the section on “mumps”.

13. Epidemic hemorrhagic fever

Figure S13. Daily count of Weibo posts with the keyword for “hemorrhagic fever” (出血热) in 2012 among Sina Weibo users with 1000 followers or more.

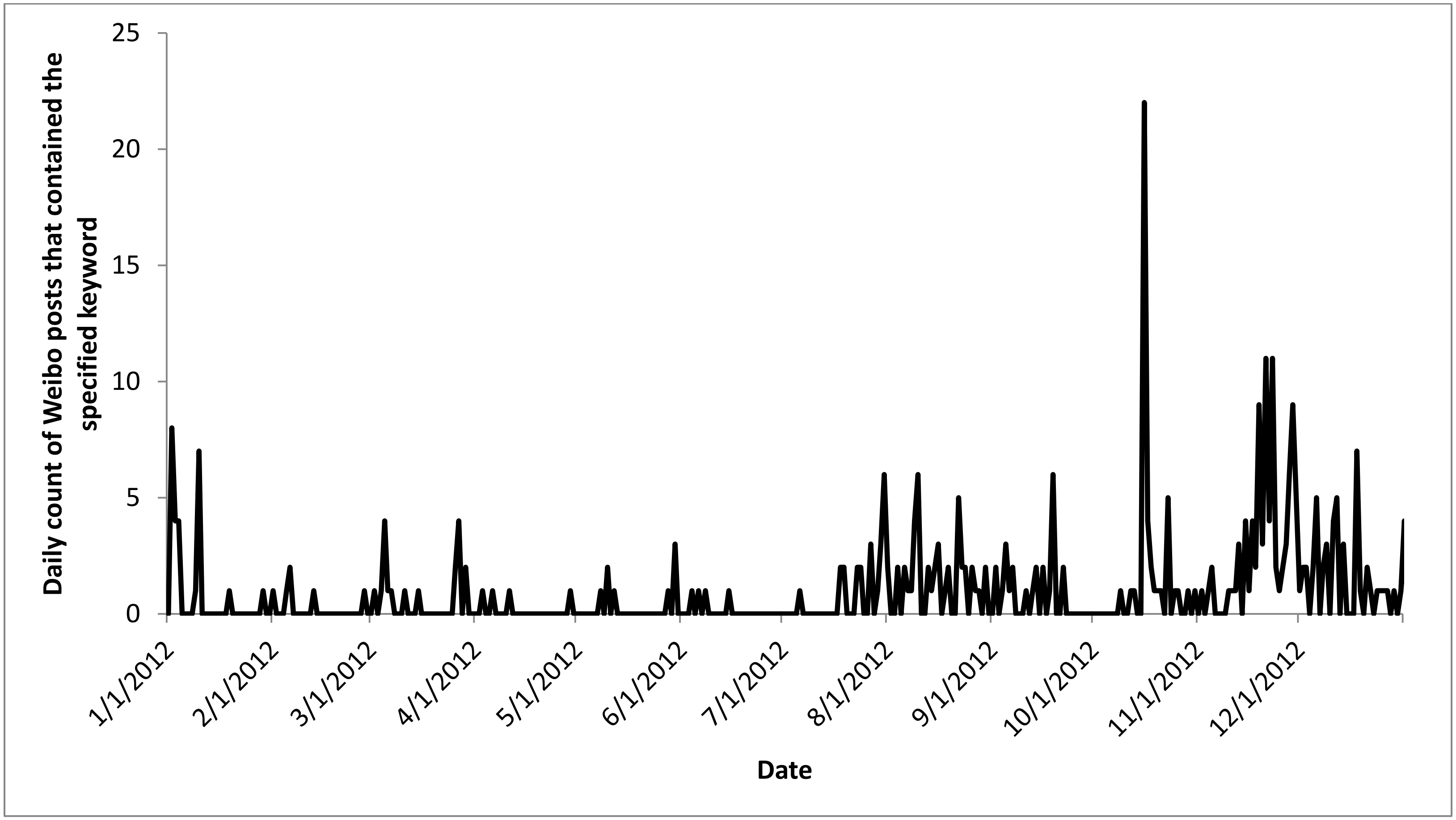

Content analysis was performed for Weibo posts generated on the peak date of October 16, 2012.

Peak Date: October 16, 2012

Total count: 22

On October 16, 2012, there were 22 Weibo posts that mentioned "hemorrhagic fever" (出血热). These Weibo posts were extracts of Chinese news articles that translated the British news article, "The Armageddon virus: Why experts fear a disease that leaps from animals to humans could devastate mankind in the next five years" (*Daily Mail*, October 16, 2012, http://www.dailymail.co.uk/sciencetech/article-2217774/The-Armageddon-virus-Why-experts-fear-disease-leaps-animals-humans-devastate-mankind-years.html). Prof. John Oxford of Queen Mary Hospital, London, commented upon a recent fatal case of Crimean-Congo Hemorrhagic Fever in the UK.

Table S13. Sample Weibo entries that contained the keyword for "hemorrhagic fever" (出血热) on October 16, 2012.

**Sample Weibo posts on that day**	**English translation / description**	**Count (%)**	**Sources / References / Notes**	**Category**
**Prof. John Oxford's comment the "Armageddon virus"**		**22 (100%)**		**News of a case**
【权威专家警告或出现"末日病毒"】英国近期接连发现从国外感染致命疾病的案例，先后有 2 名男子感染克里米亚-刚果出血热(CCHF)及新型冠状病毒。因此有权威疾病专家认为，未来 5 年全球正面临爆发新疫潮的威胁，而这些源自动物的人畜共通病，或成为"末日病毒"。	"Authoritative expert warns that "Doomsday virus"* may arise." Recently in the UK, there had been repeated imported fatal cases of infection: 2 men infected with Crimean-Congo Hemorrhagic Fever and Novel Coronavirus respectively. Therefore, an authoritative expert thought that in the next 5 years, the globe will face the threats of new epidemics directly. These zoonotic diseases may become "Doomsday virus".	19 (86%)	http://t.cn/zlHbnww 中新网<br><br>This China News Net article adapts information from *Wen Wei Po* (文匯報, Hong Kong, October 16, 2012): http://paper.wenweipo.com/2012/10/16/GJ1210160023.htm This article was extract-translation of the *Daily Mail* article aforementioned.	News related to Crimean-Congo Hemorrhagic Fever and Novel Coronavirus (i.e. MERS-CoV)
【专家称未来 50 年源自动物流行疾病或毁灭人类】10 月 16 日消息，据《每日邮报》报道，克里米亚-刚果病毒出血热会导致患者突然出现头痛、高热、关节痛、胃痛和呕吐的症状。随着病情加重，患者会出现大面积青肿和不受控制的出血。对至少 30%的病例来说是致命的。	"Expert says that in the next 50 years zoonotic diseases may destroy humanity." October 16 reports. According to the Daily Telegraph, Crimean-Congo Hemorrhagic Fever may make the patient usually show symptoms of headache, high fever, joint ache, stomach ache and vomiting. As the disease progresses, the patient will demonstrate a large area of bruising and uncontrolled bleeding. The case fatality rate is at least 30%.	3 (14%)	http://tech.sina.com.cn/d/2012-10-16/09187706375.shtml<br><br>This Sina Technology article was translated and adapted from the *Daily Mail* article aforementioned, but it changed the expert's prediction as reported in the *Daily Mail* from five years to 50 years.	News related to Crimean-Congo Hemorrhagic Fever

*The *Daily Mail* title Armageddon virus was translated as "末日病毒" (Doomsday virus), which was the Chinese name of the movie *Carriers* (2009), directed by À lex and David Pastor.

14. Rabies

Figure S14. Daily count of Weibo posts that contained the keyword for "rabies" (狂犬病) in 2012 among Sina Weibo users with 1000 followers or more.

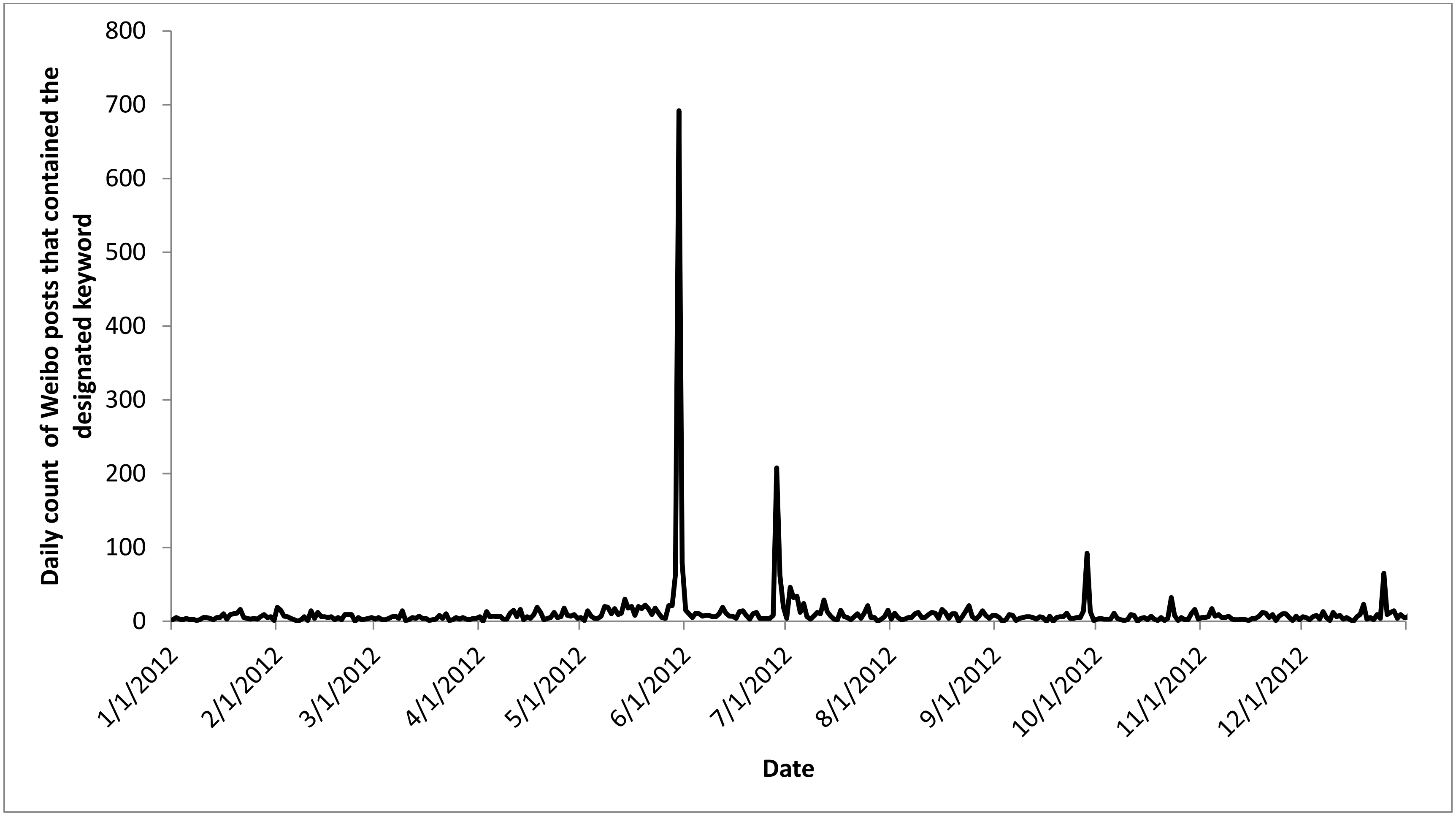

Content analysis was performed for Weibo posts generated on the peak date of May 30, 2012.

Peak Date: May 30, 2012
Total count: 692

Summary: Of the 692 Weibo posts generated on May 30, 2012, that mentioned the keyword for “rabies” (狂犬病), 675 (97.54%) were related to the so-called Miami cannibal attack that occurred on May 26, 2012. Some Weibo users commented on the news and asked whether the alleged criminal had rabies.

Only 7 of these 692 Weibo posts were genuinely related to health information or personal experience that was somehow related to rabies. Four posts were about an article on the “high cost but low effectiveness” rabies prevention program in China. One post was about keeping pets with kids and mentioned rabies. Another post was about the user’s personal experience of being bitten by a dog and afterwards receiving the vaccine against rabies. Another post was about the story of Pasteur.

The other posts were derogatory uses of the Chinese term for rabies to criticize or abuse other people. These include a comment on news of a university student carried by an old lady to class (n=6), a comment on animal right activists (n=2), and curses (n=2).

Table S14. Sample Weibo entries that contained the keyword for “rabies” (狂犬病) on May 30, 2012.

Sample Weibo posts on that day	English translation / description	Count (%)	Sources / References / Notes	Category
**Miami cannibal attack**				
//@[user1]： 地恩诶不符，也没衣服穿吗，不解。 //@[user 2]： 狂犬病吧？ //@[user 3]： 不知道真假啊！ //@[user 4]： 不会真是“生化危机”来了吧，虽然我是个生化游戏迷。 //@[user 5]： 什么情况！	[User 1] DNA not matching, no clothes to wear. Don’t understand. // [User 2] Rabies? // [User 3] Don’t know whether this is true or not! //[User 4] Hope that the “Resident Evil” has not arrived, even though I am a big fan of the game “Resident Evil”. //[User 5] What’s the situation?	675 (97.54%)	美国一名裸体男子当街啃食他人面部被击毙 http://news.sina.com.cn/w/p/2012-05-27/165824487500.shtml	Comments on the news of the Miami cannibal attack
**Health information or personal experience about rabies**				
【被误读的狂犬病 夸大宣传的背后原因】狂犬病在 2006 年开始反弹达到高峰，付出的代价世界第一，效果却是世界倒数第二。对这种死亡率达 100%的传染病的知识普及和夸大宣传，不但让民众陷入敏感恐慌，也让中国狂犬病防治陷入投入高效率低的尴尬境地。 http://t.cn/zOrAuuE	“Rabies misread The underlying reasons behind the exaggerated propaganda” Rabies has rebounded since 2006 and has reached its peak. The costs that we paid were the top in the world and yet the effect ranked number 2 from the bottom. The popularization of the knowledge and the exaggerated propaganda of an infectious disease that has a 100% case-fatality rate, has led the people into panic and has led the prevention and treatment of rabies in China into an embarrassing situation of high input and low effectiveness.	4 (0.58%)	http://discovery.163.com/special/rabies/	Health information

飞来横祸，躺着中枪。今天在散步时被狗咬，想来若是有条记载：曹谁死于狂犬病，将是天大的笑话，赶紧去接种疫苗，刚刚回来。	A tragic accident, lying down and "shot". Today when I went for a walk, I was bitten by a dog. If in the future there is a record in history that so and so died from rabies, this will be a big joke. I quickly went to receive the vaccine and have just returned.	1 (0.14%)	Original Weibo post	Personal experience about being bitten by a dog and then receiving the rabies vaccine
【家有宠物的妈咪，不得不看的两大警惕!】你以为等小宝宝能爬会走了，和猫狗呆在一起就会安全多了？你以为只有被咬伤才可能被猫狗传染上狂犬病，所以只要不让宠物咬着宝宝就行了？ 让宝宝安全卫生地与家中宠物相处，妈咪们还要补补课哦！ http://t.cn/zOgAW5k	"Mums who have pets at home. Two warnings that you must read!" Do you think that when your babies can crawl and walk, spending more time with cats and dogs, they would be safe? Do you think that rabies can only be transmitted through bites, so as long as the pets do not bite the babies, they are fine? Don't let babies to live with domestic pets in a safe and hygienic manner, there are many lessons for mums to learn! http://t.cn/zOgAW5k	1 (0.14%)	Original Weibo post	Health information
"巴斯德本人在他那举世无双的著名成就基础之上发明了一种人体免疫法，此法使人接种后对可怕的狂犬病具有免疫能力。从那时起，其他科学家也发明了防治许多严重疾病如流行性斑疹伤寒和脊髓灰质炎的疫苗。"（认为中医发明了疫苗的，证据来自清朝某个无名老中医神奇地用牛痘粉治愈了皇帝的儿子的故事）	"Pasteur himself built upon his unparalleled achievement and discovered a way to immunize humans. This method confers immunity after vaccination against the terrible rabies. Since then, other scientists have also invented vaccines against other serious illness, such as typhus and polio." (Those who believed that practitioners of Traditional Chinese Medicine invented vaccine, their evidences come from the story of an anonymous old practitioner of Traditional Chinese Medicine, who miraculously cured the Emperor's Son using vaccinia powder.)	1 (0.14%)	Original Weibo post	Health education
**Personal Comments / News unrelated to rabies**				
我只想说，人家凭什么背你!! 你还是孩子嘛？？还是得狂犬病了？？//@[user]：可敬，不过学校看这么大雨都淹成这样了还不停课，是死猪啊，没脑子。。学生主	I only want to say: for what reasons why other people have to carry you on their back!! Are you a kid?? You got rabies?? //@[user] Honorable. However, in such a	6 (0.87%)	清洁工冒雨背女大学生上课 学生被指娇气 http://video.sina.com.cn/p/news/s/v/2012-05-	Comments on the news that cleaners carried

观能动性也差，实在去不了就不去了呗，非要去就穿雨鞋啊，或者脱了鞋手拿着，怎么都能过去吧，难道阿姨要把每个人都背过去吗	heavy rain, the university has flooded as such. Why wouldn't they suspend the classes? Like dead pigs, no brain…The students are slow to take initiatives either. If they cannot really attend classes, then just don't go. If you want to go, wear a pair of rubber boots, or take off your shoes and use your hands to carry them. Anyhow, you will manage to cross the water. Is it really that the old lady has to carry everyone to cross the water?		30/193961763491.html?recomm=relation	students to school in the rain
猫狗奴伪动保只会四出打人骂人咬人诅咒人，和狂犬病狗没有任何区别，却每天自己觉得自己爱心四溢。这是一种严重的心理变态疾病，不但要有法律对其作出惩处，而且必须送到医院强制治疗。	The "slaves of cats and dogs" and the pseudo animal protection activists only know how to beat, abuse, bite and curse people. They are not different from dogs with rabies. Yet they think that their love is overflowing. This is a kind of serious psychological disorder. Not only should there be laws to punish them. They should be sent to hospitals for treatment	2 (0.29%)	Original Weibo post	Personal comment (debates about animal protection and animal rights)
**Others**		2 (0.29%)	Curses / Abusive language used against certain people	Personal comment

15. Epidemic encephalitis B

Figure S15. Daily count of Weibo posts with the three different keywords for "epidemic encephalitis B" in 2012 among Sina Weibo users with 1000 followers or more.

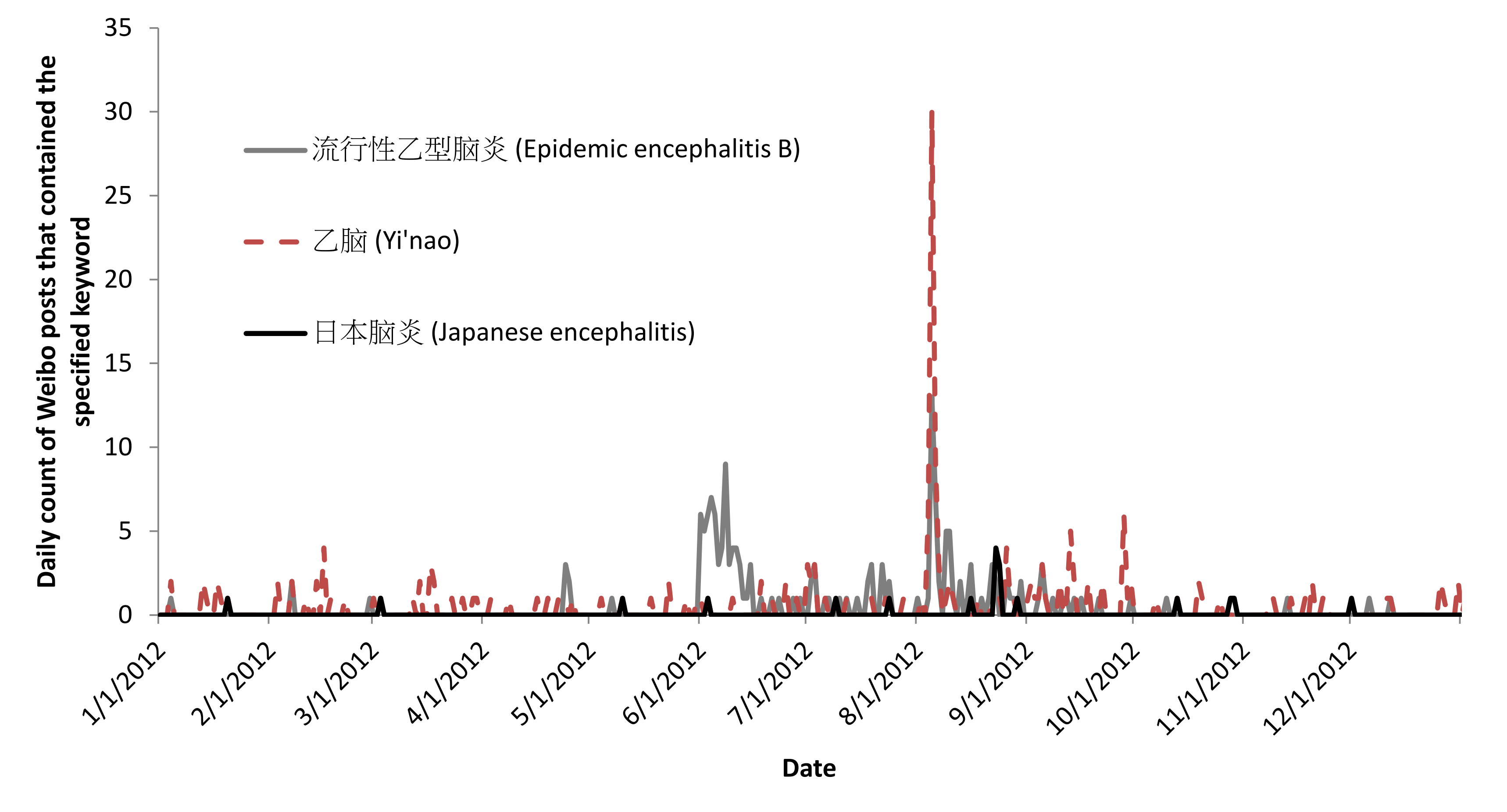

Content analysis was performed of Weibo posts of the peak date of August 5, 2012.

Peak Date: August 5, 2012

Total count:

Epidemic encephalitis B (流行性乙型脑炎) = 13;
Yi’nao (乙脑) = 30;
After removal of Weibo posts that contain both keywords = 30

Epidemic encephalitis B (流行性乙型脑炎), is shortened as Yi’nao (乙脑) in Chinese. It is also known as Japanese encephalitis (日本脑炎). Therefore, we used all three keywords in our keyword search.

For the peak of August 5, 2012, searching with the keyword “Epidemic encephalitis B” (流行性乙型脑炎) there were 13 Weibo posts, while searching with the keyword “Yi’nao” (乙脑) there were 30. Searching with “Japanese encephalitis” returns zero posts on that day. Since all the posts containing “Epidemic encephalitis B” contained “Yi’nao”, there were a total of 30 posts.

All 30 posts were related to a fatal case of epidemic encephalitis B in Ningbo City, Zhejiang Province. A 65-year-old lady died of the disease on August 2, 2012. The news was covered by news media on August 5.

Table S15. Sample Weibo entries that contained the keyword for “epidemic encephalitis B” (流行性乙型脑炎) and/or “Yi’nao” (乙脑) on August 5, 2012.

Sample Weibo posts on that day	English translation / description	Count (%)	Sources / References / Notes	Category
**A fatal case of epidemic encephalitis B in Ningbo City, Zhejiang Province**				
【宁波一女子被蚊子“咬死”】8 月 2 日，宁波一 65 岁女子因被蚊子叮咬，感染流行性乙型脑炎死亡。这是宁波今年首例乙脑死亡病例。卫生部门提醒，8 个月-12 周岁的孩子，若未接种过乙脑疫苗，要及时去当地社区卫生服务中心接种。一旦出现起病急、高热、呕吐、惊厥、意识障碍等临床症状，应立即就诊。	“Ningbo, one woman died because of a mosquito bite .” On August 2, a 65-year-old woman in Ningbo died of epidemic encephalitis B infection that was transmitted through mosquito bites. This is the first case of mortality of Yi’nao [epidemic encephalitis B] in Ningbo this year. Health authorities remind that children between 8 months and 12 years of age, who have not yet vaccinated against Yi’nao, should go to their community health centers to receive the vaccine. If there appear clinical symptoms of acute illness, fever, vomiting, convulsions and unconsciousness, they should receive medical care immediately.	28 (93%)	http://jiaxing.19lou.com/bq/%E8%9A%8A%E5%AD%90%E5%8F%AE%E5%92%AC	News about a death of epidemic encephalitis B
注意防蚊子 防乙脑	Beware to protect [yourself] from mosquitoes Prevent Yi’nao [Epidemic Encephalitis B]	1 (3%)	Original Weibo post	Health promotion

我还能打 乙脑疫苗 吗？	Can I still get vaccinated against Yi'nao [Epidemic Encephalitis B]?	1 (3%)	Original Weibo post	Personal comment

16. Dengue

Figure S16. Daily count of Weibo posts with the keyword for “dengue” (登革热) in 2012 among Sina Weibo users with 1000 followers or more.

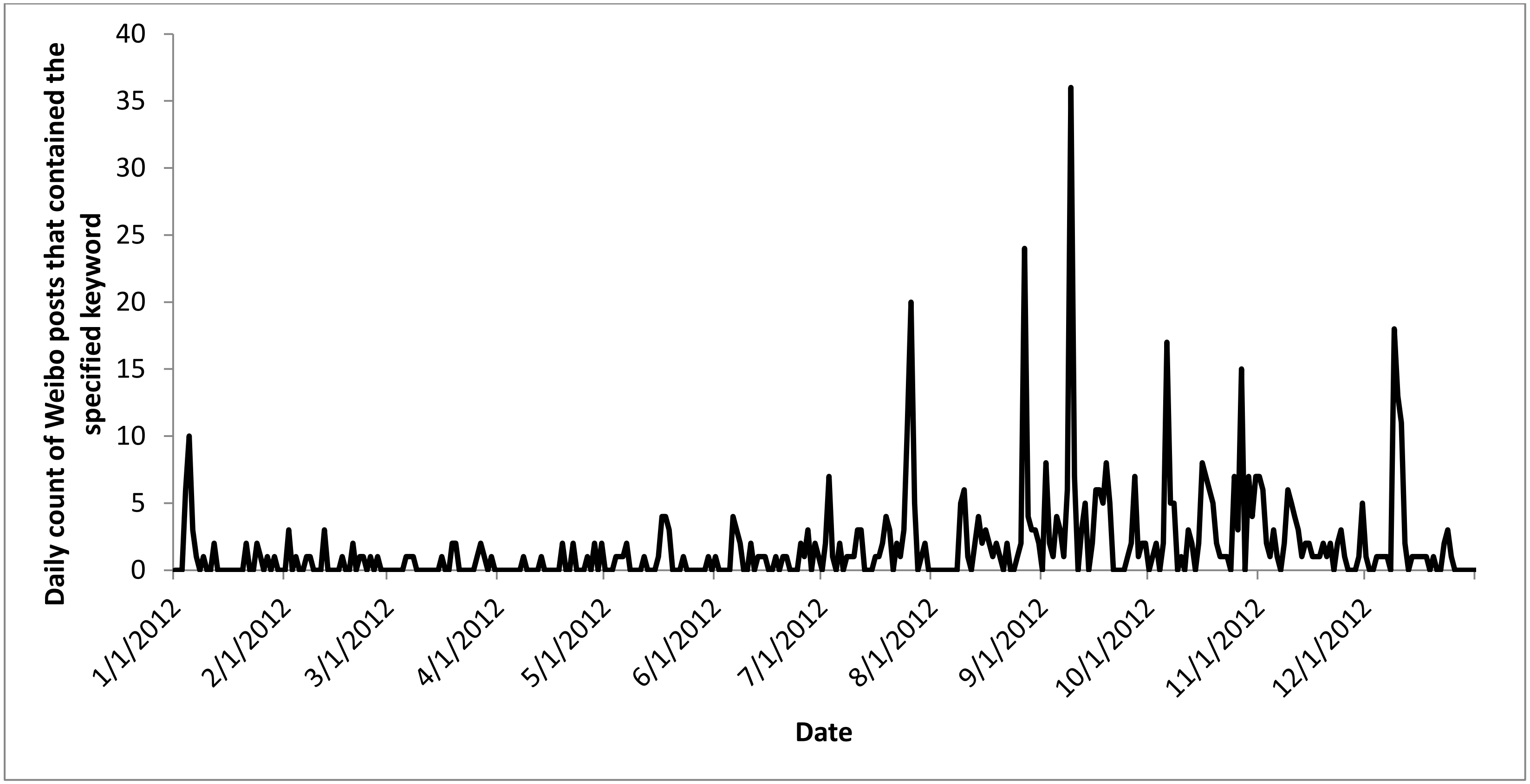

Content analysis was performed for Weibo posts generated on the peak date of September 9, 2012.

Peak Date: September 9, 2012

Total count: 36

Summary: Of the 36 Weibo posts that contained the keyword for “dengue” (登革热) on September 9, 2012, they were all commercial advertisements (and reposts thereof) for a promotional event for tourism in Thailand that provided mosquito repellent cream as a gift to prevent dengue.

Table S16. Sample Weibo entries that contained the keyword for “dengue” (登革热) on September 9, 2012.

Sample Weibo posts on that day	English translation / description	Count (%)	Sources / References / Notes	Category
#泰国旅游送长效驱蚊霜预防登革热# 转一转，相信会有好运被我转出来的！!！ 地址：http://t.cn/zWgiE7d	#Visit Thailand Mosquito repellent cream as a gift Prevent dengue# Circulate it. I believe that I can generate good luck by circulating it!!! Address: http://t.cn/zWgiE7d	36 (100%)	Original Weibo post	Commercial advertise-ment

17. Anthrax

Figure S17. Daily count of Weibo posts with the keyword for “anthrax” (炭疽) in 2012 among Sina Weibo users with 1000 followers or more.

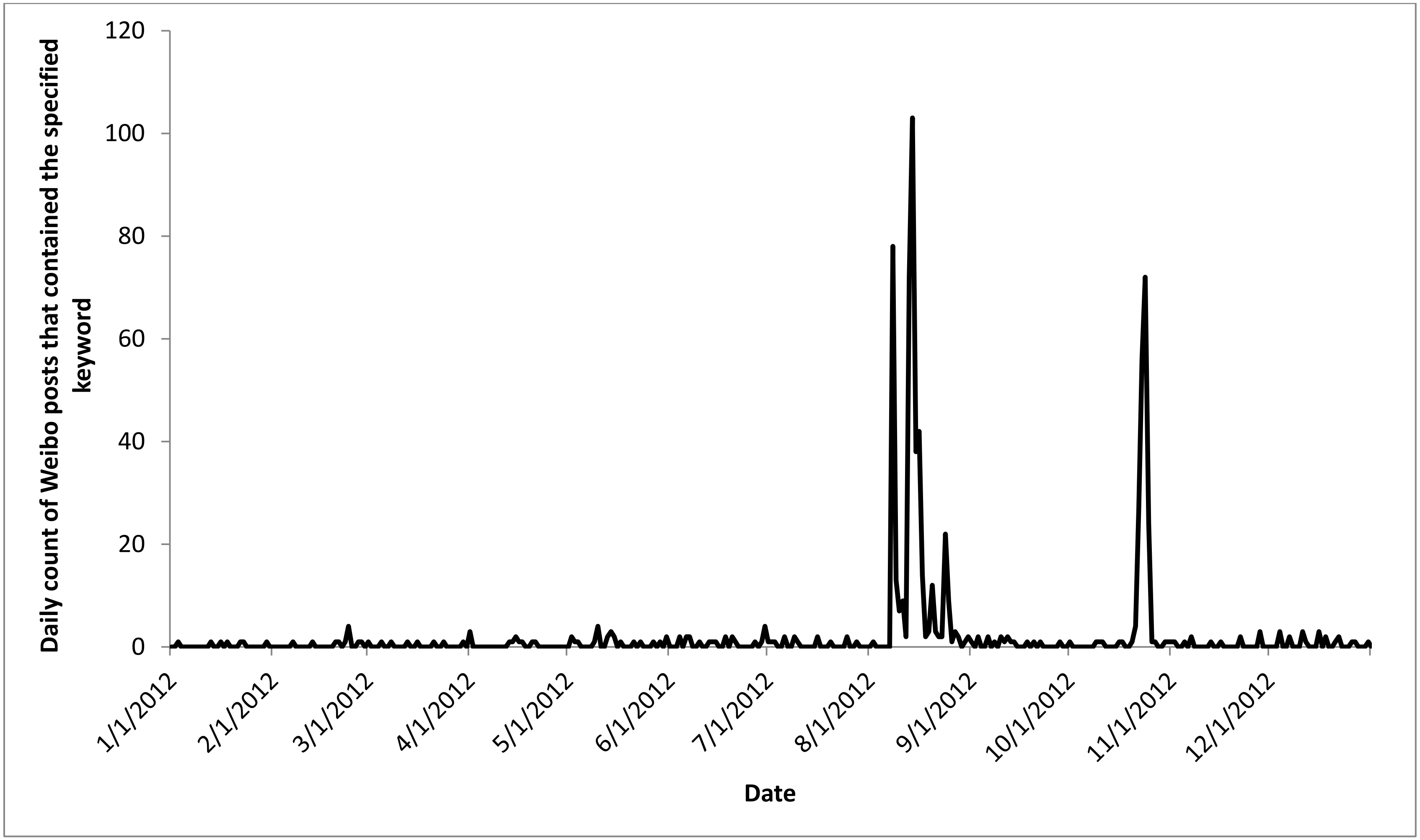

Content analysis was performed for Weibo posts generated on August 14, 2012.

Peak Date: August 14, 2012
Total count: 103

Summary: All 103 posts that mentioned “anthrax” (炭疽) on August 14, 2012 were somehow related to the news of an outbreak in Liaoling Province. Of these posts, 61 were news posts about the outbreak. Sixteen were a variety of health information messages related to anthrax prevention. Another 8 were about the blog post “There is no anthrax outbreak in our city. Citizens don’t need to panic”, released by the Director of Beijing Municipal CDC. There were 14 posts that were a variety of personal comments. Another 4 posts were categorized as “Others”.

Table S17. Sample Weibo entries that contained the keyword for “anthrax” (炭疽) on August 14, 2012.

Sample Weibo posts on that day	English translation / description	Count (%)	Sources / References / Notes	Category
【辽宁确诊 7 例人感染炭疽病 江苏月初曾现 5 人疫情】辽宁省沈阳等地近日发生人感染皮肤炭疽传染病疫情，目前已确诊 7 人发病。这是自 8 月 3 日江苏省连云港出现 2 人患病，5 人症状不典型以后，我国又一省份发现皮肤炭疽病，并出现疫情。	“In Liaoning, there are 7 confirmed anthrax cases. In Jiangsu, early this month, there had been an outbreak of 5 cases.” In Shenyang and other places in Liaoning, recently there had been an outbreak of cutaneous anthrax infection. Up to now, 7 cases were confirmed. This is the second province in our country where cutaneous anthrax was discovered and an outbreak occurred, since the outbreak of August 3rd, at Lianyungang, when 2 fell ill and 5 had atypical symptoms.	61 (59%)	Examples of news articles: http://gd.sina.com.cn/news/m/2012-08-14/07406381.html <br>http://nb.people.com.cn/GB//n/2012/0813/c201051-17353959.html	Outbreak news
【如何预防炭疽病】专家介绍，炭疽病并没有那么危险，对于非疫区居民，牛羊肉只要煮沸至 100℃40 分钟，就不会被传染。炭疽病分皮肤炭疽、肠炭疽和肺炭疽。95%的炭疽病为皮肤炭疽，通过破溃的皮肤伤口感染；肠炭疽是因食用被污染食物、水等感染；肺炭疽系呼吸道感染，致死率最高，但很罕见。	“How to prevent anthrax” According to experts’ introduction, anthrax is not that dangerous. For residents living in areas not affected by the epidemic, as long as beef and mutton are cooked at 100℃ for 40 minutes, they will not be infected. Anthrax are categorized as cutaneous, gastrointestinal and pulmonary. 95% of anthrax cases are cutaneous. They were infected through wounds on the skin. Gastrointestinal anthrax was transmitted via contaminated food and water. Pulmonary anthrax cases were infected via the respiratory tracts, with the highest case fatality rate, but they are very rare.	16 (16%)	One of many potential sources of information that carries similar information is this article: http://www.shw365.com/jkzl/jkwd/201108/90992.html	Health information
发表了博文 《我市无炭疽疫情市民不必恐慌》 - “近日，辽宁省沈阳等地发生人感染皮肤炭疽传	Publish a blog post “There is no anthrax outbreak in our city Citizens don’t need to panic” - “Recently, in Shenyang of Liaoling Province, there had been epidemics of the infectious disease cutaneous anthrax.	8 (8%)	Blog post by the Director of Beijing Municipal CDC: http://blog.sina.com.cn/s/blog_60c6170a01019isd.html	Health information (from Beijing

染病疫情，目前确认 7 人发病，其中沈阳辽中县 3 例，于洪区 1 例，其他地区 3 例，暂无死亡病例。"针对上述新闻，热心 http://t.cn/zWYVBlO	Up to now, 7 cases were confirmed, of which 3 cases are in Shenyang's Liaozhong County, 1 in Yuhong District, and 3 in other regions, and there are no deaths yet." Regarding the above news, passionate…. http://t.cn/zWYVBlO		and re-posts	Municipal CDC)
炭疽和霍乱这种以前被称作是烈性的传染病，现在治疗起来已不是什么难事儿了。	Anthrax and cholera, regarded as severe infectious diseases in the past, are not difficult to treat today.	14 (14%)	Original Weibo post	Personal comments
【金点子提醒：炭疽疫情或催生医药疫苗股行情】	"Golden Idea reminder: Anthrax epidemic may boost the price of stocks of pharmaceutical companies"	4 (4%)	Financial predictions: http://www.twioo.com/content/8043216	Others *

* This group contains different Weibo posts that cannot be grouped into other groups.

18. Bacterial and amoebic dysentery

Figure S18. Daily count of Weibo posts with the keyword for "dysentery" (痢疾) in 2012 among Sina Weibo users with 1000 followers or more.

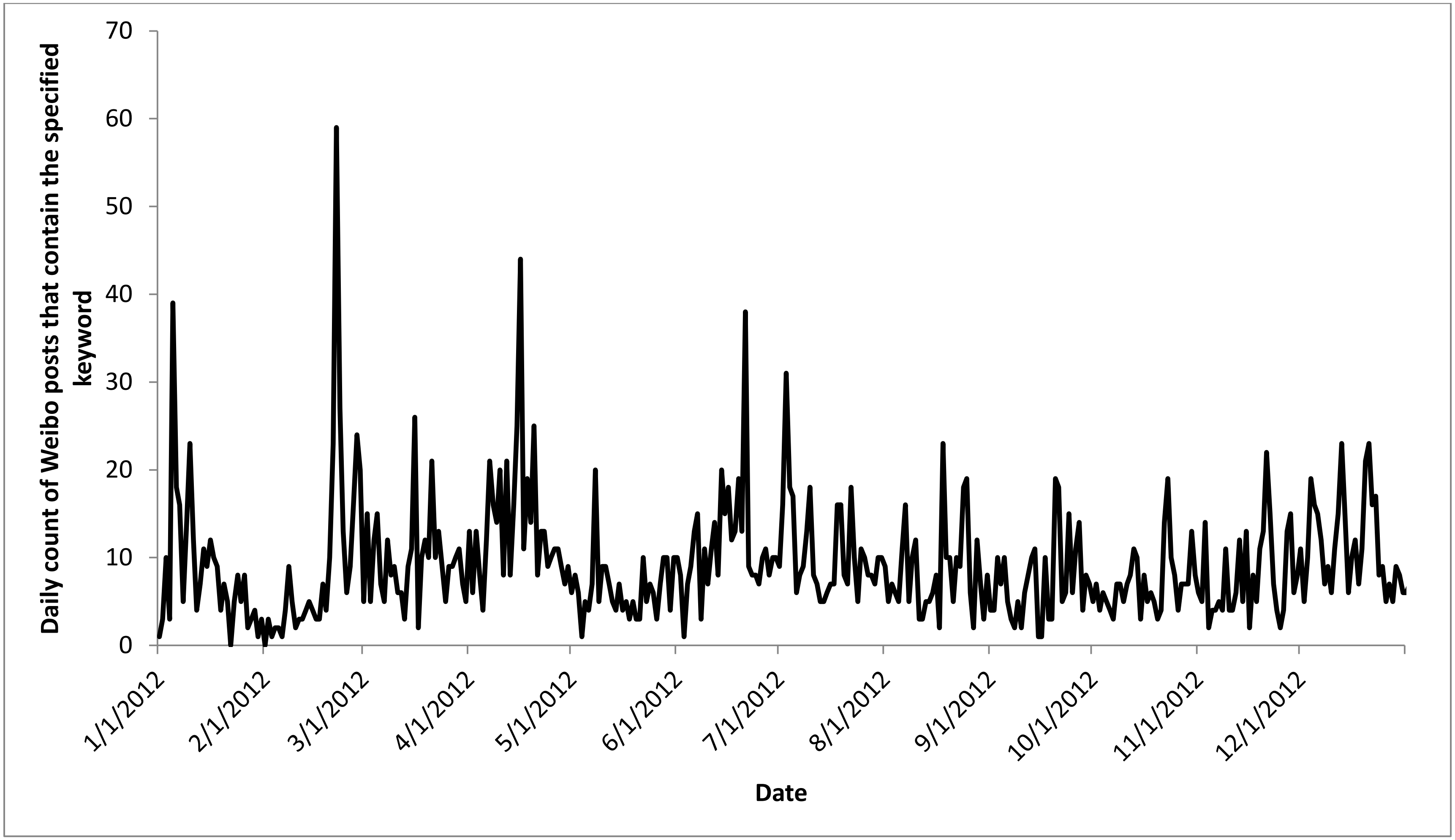

Content analysis was performed for Weibo posts that were created on the peak date of February 22, 2012.

Peak Date: February 22, 2012
Total count: 59

Summary: All 59 Weibo posts that contained the keyword "dysentery" (痢疾) on February 22, 2012 were posts that we categorized as alternative health information, of which 58 were about "food that cannot be consumed together with chicken meat". This post (see table) mentioned eating chicken and *penis et testis canis* (a type of traditional Chinese medicine) together would lead to dysentery; similarly, if people ate chicken and Chinese plum together, they would experience dysentery.

Table S18 Sample Weibo entries that contained the keyword for "dysentery" (痢疾) on February 22, 2012.

Sample Weibo posts on that day	English translation / description	Count (%)	Sources / References / Notes	Category
【不能跟鸡肉搭配地食物】⑴芝麻（可导致死亡）；⑵地瓜（导致腹痛）；⑶消炎片（中毒）；⑷狗肾（引起痢疾）；⑸李子（食则拉痢）；⑹豆浆（降低蛋白质吸收）；⑺糯米（引起身体不适）；⑻菊花（可导致死亡）；⑼芥末（伤元气）；⑽大蒜（性味相克）；⑾鲤鱼（性味相克）；⑿鲫鱼（性味相克）。	"Food that cannot be consumed together with chicken meat" (1) sesame (can lead to death); (2) sweet potato (leading to abdominal pain); (3) anti-inflammatory tablet (poisoning); (4) *Penis et Testis Canis* (leading to dysentery); (5) Chinese plum (if eaten, dysentery); (6) soya milk (lower protein absorption); (7) Glutinous rice (can lead to bodily discomfort); (8) Chrysanthemum (can lead to death); (9) mustard (damages vitality); (10) garlic (the nature and taste of the foods do not match); (11) carp (the nature and taste of the foods do not match); (12) *Carassius auratus* (the nature and taste of the foods do not match).	58 (98%)	http://mp.weixin.qq.com/mp/appmsg/show?__biz=MjM5NTY3OTU2MA==&appmsgid=10006644&itemidx=4&sign=8e7487f4dcf503f4e59cbd1c0d5481d1	Alternative health information
【生活巧用醋】1 喝较淡的醋水，可防晕车。2 食醋与冷开水搅匀，加等量蜂蜜，治失眠。3 发生昏厥，手帕浸过食醋捂在晕厥者鼻子上，刺激患者清醒。4 烹调蟹、海蜇时，用 1%的醋浸泡一小时，可防止食物中毒。5 用后的餐具，用 1%食醋煮沸，可防肝炎、痢疾等传染病。6 喝浓度 50%的食醋，可解酒养肝。	"Skillful use of vinegar in life" (1) Drink a light mix of vinegar and water can help prevent motion sickness. (2) A well-mixed mix of vinegar and cold water, with some honey added, can help cure insomnia. (3) If someone has fainted, use a handkrief that has been soaked with vinegar to cover his nose to awaken the patient. (4) When one cooks crabs and jellyfish, using 1% vinegar to sok them can help prevent food poisoning. (5) Boiling cutlery after use in 1% vinegar to the boiling point, can help prevent hepatitis, dysentery and other infectious diseases. 6. Drinking vinegar of 50% concentration can help one overcome hangover and can protect the liver.	1 (2%)	http://www.oouee.com/401.html	Alternative health information

19. Tuberculosis

Figure S19. Daily count of Weibo posts with the keyword for “Tuberculosis” (肺结核) in 2012 among Sina Weibo users with 1000 followers or more.

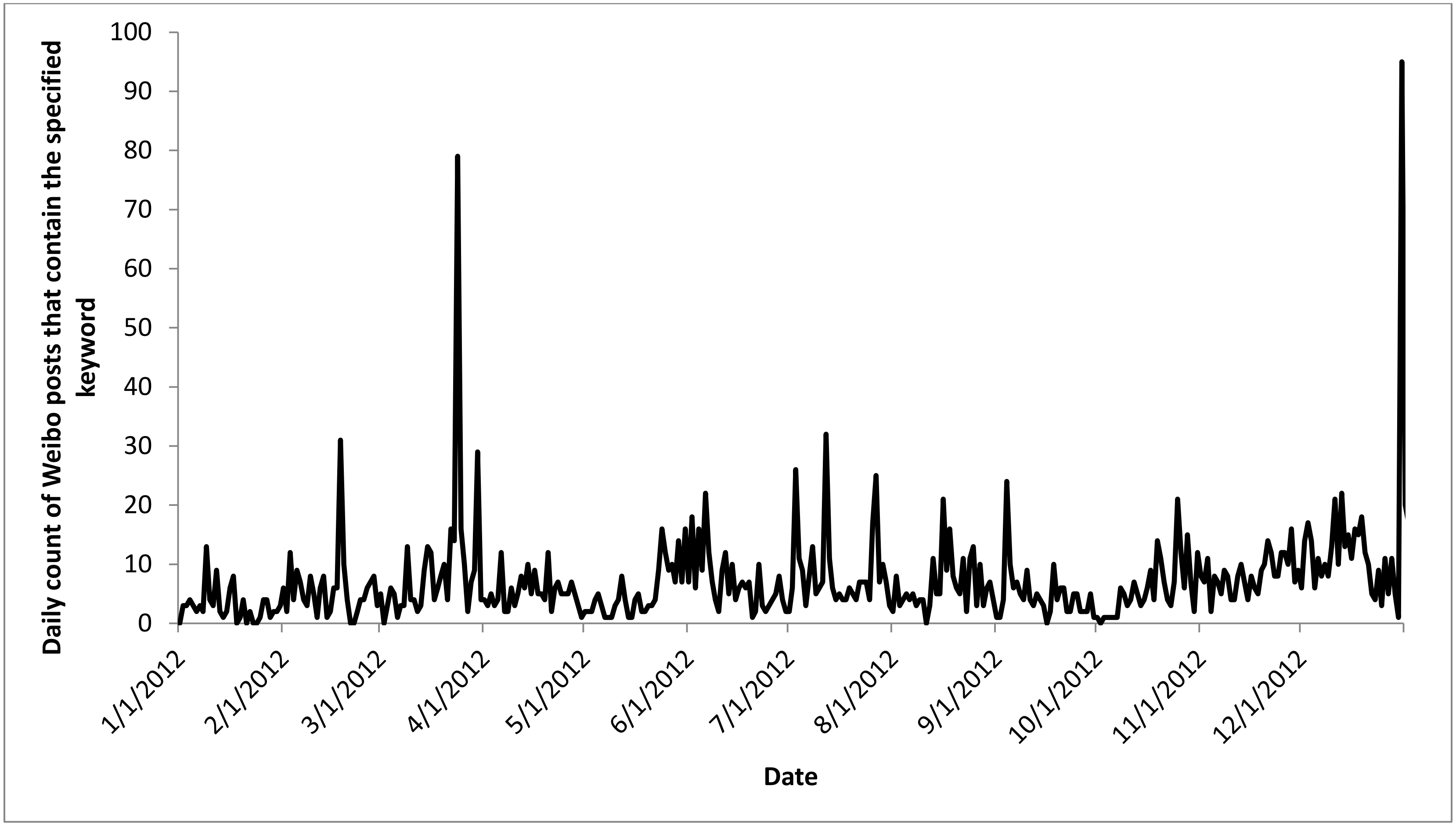

Content analysis was performed on Weibo posts generated on the peak date of December 31, 2012.

Peak Date: December 31, 2012.
Total count: 95

Summary: Of the 95 Weibo posts that mentioned "tuberculosis" (肺结核) on December 31, 2012, 90 were about a child torture case. The kindergarten teacher who allegedly tortured certain children, facing the compensation requests made by the parents, pleaded for mercy as her father suffered from tuberculosis and her mother had mental disability.

Another 5 posts mentioned that persimmon tea was good for the health of tuberculosis patients.

Table S19. Sample Weibo entries that contained the keyword for "Tuberculosis" (肺结核) on December 31, 2012.

Sample Weibo posts on that day	English translation / description	Count (%)	Sources / References / Notes	Category
【浙江虐童女幼师称爱孩子 无力赔偿哭求原谅】虐童过去了 2 个多月，对颜艳红来说远没有结束，5 位家长分别向她提出了精神抚慰金五万及道歉等要求。颜艳红说她喜欢孩子，当时未意识到伤害行为。她哭着说父亲有肺结核母亲精神残疾，无力赔偿，希望家长能原谅她。	"Female kindergarten teacher from Zhejiang claims to love kids. She cannot afford the compensation and asks for forgiveness in tears." It has been two months since the child abuse case, but it is far from over for Liu Yanhong. Five parents respectively requested her apology and 50,000 yuan in compensation for emotional distress. Liu Yanhong said that she liked kids and did not realize the harmful behavior at that time. She cried that her father suffered from tuberculosis and her mother had mental disability, so that she was unable to make monetary compensation and begged for forgiveness from the parents.	90 (95%)	http://news.sina.com.cn/c/sd/2012-12-31/034825930772.shtml	News related to a case of alleged child torture
柿茶: 茶叶 3 克，代饼一 3 个，冰糖 5 克，将柿饼加人冰糖煮烂后冲茶服，可理气化痰、益脾健胃，最适合肺结核患者饮。	Persimmon tea: 3 grams of tea leaves, 3 dried persimmon cakes, 5 grams of crystal sugar. Boil the dried persimmon cakes with crystal sugar until they are tender. Then make tea with the boiled persimmon cakes. The tea can help regulate *qi*-flowing, eliminate phlegm, and benefit the spleen and stomach. It is the most suitable drink for tuberculosis patients.	5 (5%)	http://health.lady8844.com/yinshi/2012-12-06/1354782041d1130073_4.html	Alternative health information/ Traditiaonal Chinese Medicine

20. Typhoid or paratyphoid

Figure S20. Daily count of Weibo posts with the keyword for "typhoid" (伤寒) in 2012 among Sina Weibo users with 1000 followers or more.

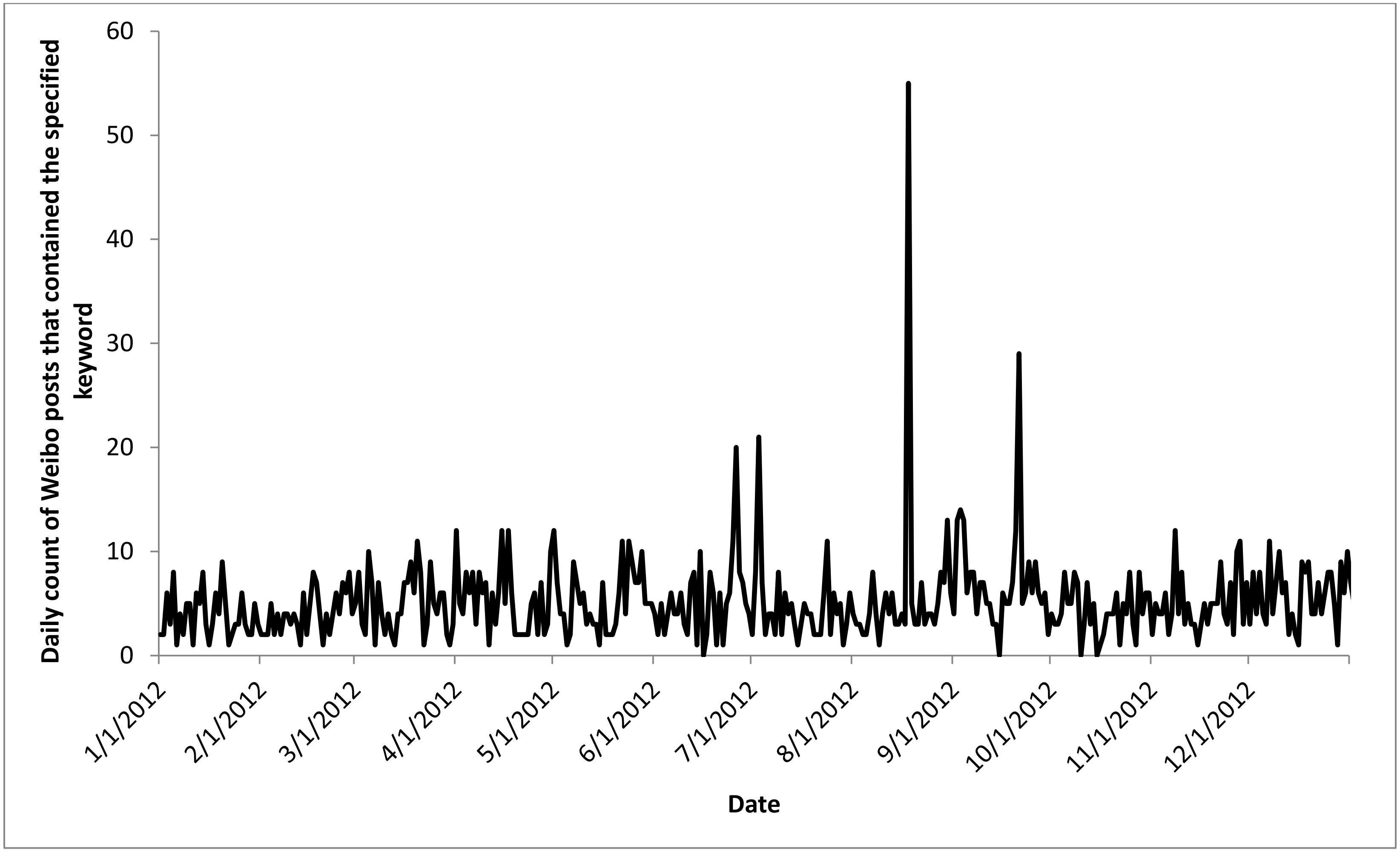

Since in Chinese, both typhoid (伤寒) and paratyphoid (副伤寒) contained the Chinese keyword for "typhoid" (伤寒), we only used 伤寒 as the keyword for our keyword search. Content analysis was performed for Weibo posts generated on the peak date of August 18, 2012.

“False positive” Peak Date: August 18, 2012

Total count: 55

Summary: Of the 55 Weibo posts generated on August 18, 2012, that mentioned the Chinese keyword for “typhoid” (伤寒), 46 posts were actually related to a story about a suspected case of meningitis – the story of the potential lumbar puncture of a small girl, Miss Yang (Chinese title: 杨娃娃腰椎穿刺惊魂). For details of the story, please refer to the Section 21 (Epidemic meningitis) in this Supplemental Online Appendix.

Of these 46 posts, 42 mentioned a classic text of Traditional Chinese Medicine, titled *Shang Han Lun* (《伤寒论》), of which the standard English translation is *On Cold Damage*. It so happens that the Chinese term “Shang Han” (伤寒) refers to a broad category of diseases and symptoms (collectively known as Cold Damage) in Traditional Chinese Medicine, while it refers specifically to typhoid in modern (Western) medicine (Table S20A).

Since the majority of the posts were not related to typhoid or paratyphoid, we considered the peak of August 18, 2012, as a “false positive”. We therefore, moved on to analyze the next peak.

Table S20A. Sample Weibo entries that contained the keyword for “Typhoid” (伤寒) on August 18, 2012.

Sample Weibo posts on that day	English translation / description	Count (%)	Sources / References / Notes	Category
**Comments on a suspected case of meningitis**		46 (83.6%)		Social issue
腰穿惊魂，只因一个简单的少阳发烧。《伤寒论》379 条：呕而发热者，小柴胡汤主之。本次《中医育儿必修课》会讲到这个证型。	“Fright night” about the lumbar puncture, merely because of a fever caused by Lesser *Yang*. Line 379 of Shang Han Lun: “When [there is] retching and heat effusion, Minor Bupleurum Decoction (*xiao chai hu tang*) governs.” “Compulsory course on parenting by adopting traditional Chinese treatment” this time will talk about the above symptom.	42 (76.4%)	http://tweets.seraph.me/search/中医育儿必修课暑期班<br><br>The translation of the Line 379 of Shang Han Lun is taken from Zhongjing Zhang (translated by Wiseman, Feng and Wiseman), *Shang Han Lun = On Cold damage: translation and commentaries*, Brookline, Massachusetts, Paradigm Publications, 1999 p.565. This is the standard English translation of this Traditional Chinese Medicine classic.	Personal comment / Traditional Chinese Medicine (mentioning the classic *Shang Han Lun*)
Posts that commented on that suspected case that mentioned 伤寒 in the sense of typhoid		3 (5.5%)		Personal comment
Posts that commented on that suspected case that mentioned 伤寒 in the sense of Cold		1 (1.8%)		Personal comment /

Damage in Traditional Chinese Medicine				Traditional Chinese Medicine
**Alternative health information / Traditional Chinese Medicine**		2 (3.6%)		
#装修学堂#【装修新居后去除异味的好方法】吊兰、芦荟、虎尾兰能大量吸收室内甲醛等污染物质，消除并防止室内空气污染；茉莉、丁香、金银花、牵牛花等花卉分泌出来的杀菌素能够杀死空气中的某些细菌，抑制结核、痢疾病原体和伤寒病菌的生长，使室内空气清洁卫生。	#School for home decoration# [Effective ways to remove undesirable odor after decorating your new home] Spider plants, aloe vera, and sansevierias can absorb lots of pollutants like indoor formaldehyde. They can also help eliminate and prevent indoor air pollution. Antibacterial molecules secreted by flowers such as the jasmine, clove, honeysuckle and morning glory is able to kill some bacteria in the air, inhibit the growth of tuberculosis, dysentery pathogens and typhoid, and keep the air clean indoors.	1 (1.8%)	http://www.owinmall.com/news_center/40.html	Alternative health information
Posts that mentioned 伤寒 in the sense of Cold Damage in Traditional Chinese Medicine		2 (3.6%)		Traditional Chinese Medicine
**Others**		5 (9.1%)		
A post that mentioned a missionary in China, who invented the written language for the Miao ethnic group. He died of typhoid back in 1915.		1 (1.8%)		History
Fiver posts that mentioned 伤寒 but in the context of breakup in love relationship		5 (9.1%)		Personal comments

Peak date: September 21, 2012.

Total count: 29

Summary: Of the 29 Weibo posts created on September 21, 2012, that mentioned the keyword for "typhoid" (伤寒), 16 posts were related to a media report in 2010 about the vending machine selling "dirty water", an advertisement in World Water Week 2010 for UNICEF's Tap Project. It is interesting to observe that even a Weibo post that mentioned an "old" news could still attract people's attention and so they reposted it. There were another 13 posts that were related to the "danger" of poor-quality napkins in restaurants (Table S20B).

Table S20B. Sample Weibo entries that contained the keyword for "Typhoid" (伤寒) on September 21, 2012.

Sample Weibo posts on that day	English translation / description	Count (%)	Sources / References / Notes	Category
这个竖立在曼哈顿街头的自动贩售机，里面出售的是让人惊奇的"脏水"，每瓶一美元。这些脏水是货真价实的含有病菌的脏水，更让人称奇的是，您还可以 选择脏水的"口味"：伤寒、疟疾、霍乱和肝炎。	This vending machine on the street of Manhattan, surprisingly sells "dirty water" for 1 US dollar a bottle. This dirty water is genuine dirty water with germs. More surprisingly, you can also choose the "taste" of the dirty water: typhoid, malaria, cholera and hepatitis.	16 (55%)	A media report in English, published on July 23, 2010: http://inhabitat.com/unicefs-dirty-water-vending-machine-offers-8-flavors-of-disease/<br><br>A media report in Chinese, published on July 27, 2010. http://www.patent-cn.com/2010/07/27/42932.shtml	Health education (UNICEF's Tap Project, World Water Week 2010)
【饭店餐巾纸危害】现在餐馆都会提供免费餐巾纸，但有些餐巾纸却有致病隐患。专家表示，劣质餐巾纸带有各种真菌、大肠杆菌、结核菌、肝炎病毒等，易引发肠炎、伤寒、痢疾、肝炎等病症。餐巾中添加漂白剂含有致癌化学物质，如荧光增白剂中的重金属镉，会损伤血液系统及记忆力。	"The danger of napkins in restaurants" Nowadays, restaurants provide free napkins. However, there is a hidden danger in some napkins. Experts say that napkins of poor quality contain various types of fungi, Escherichia coli, Mycobacterium tuberculosis, hepatitis virus, and will easily lead to disease such as enteritis, typhoid, dysentery and hepatitis. The bleaching additives in napkins contain carcinogenic chemicals, such as heavy metal cadmium in fluorescent brightener, which can damage the circulatory system and memory.	13 (45%)	This is a post that has been circulated in a variety of websites and social media platforms.	Health information

21. Epidemic meningitis

Figure S21. Daily count of Weibo posts that contained the designated keywords for epidemic meningitis. We used three keywords for detection: 1) 脑膜炎 (Nǎomó yán) (grey line), 2) 脑脊髓膜炎 (Nǎo jǐsuǐ mó yán) (black line) and 3) 流脑 (Liú nǎo) (red dotted line). We found that the daily number of Weibo posts that carried the second and third keywords was small – zero in most days – and the maximum daily count in 2012 for the second keyword is 9 and the third keyword is 15. For our content analysis, we focus on the peak for the first keyword.

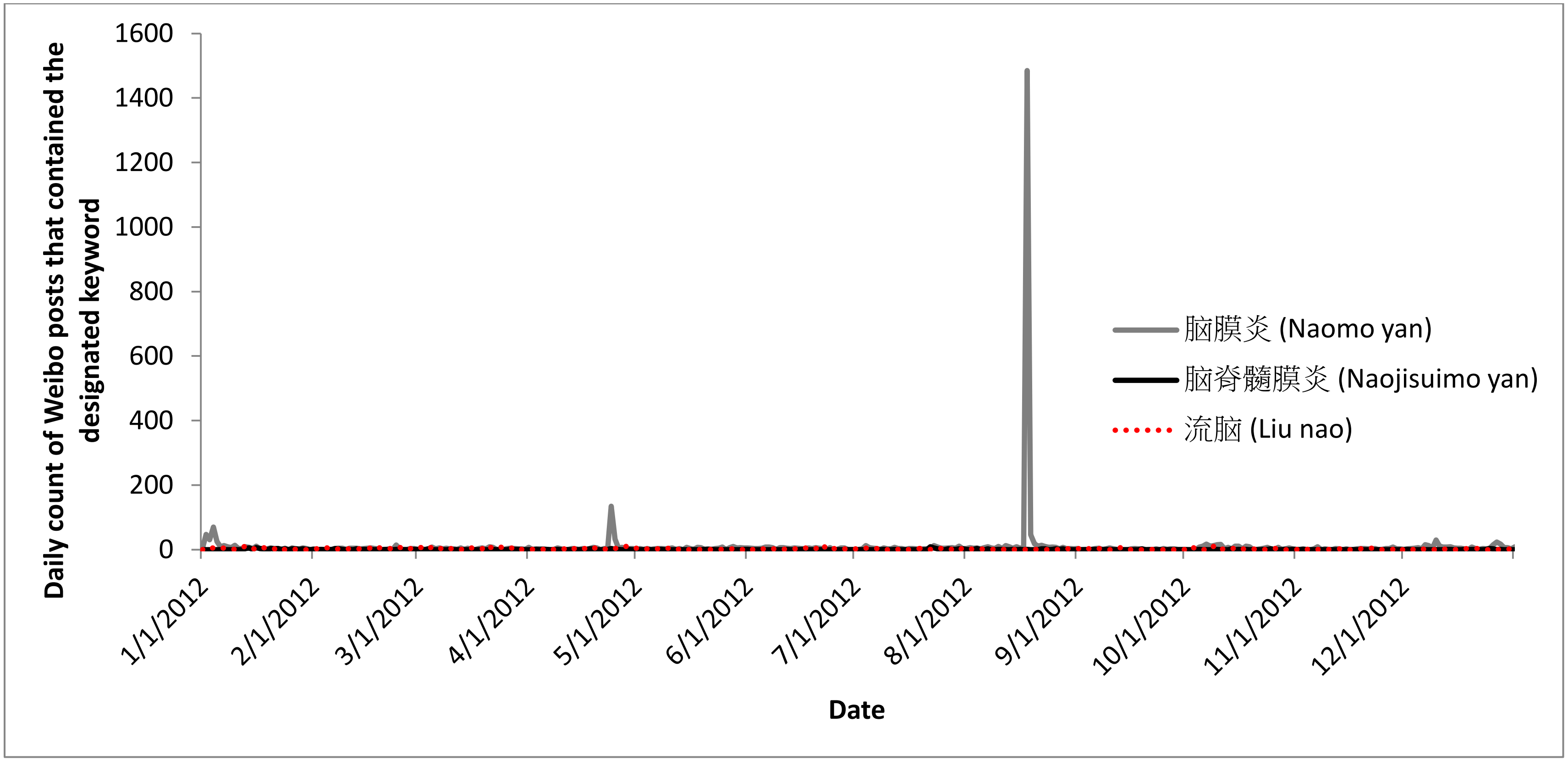

Content analysis was performed for Weibo posts generated on the peak date of August 18, 2012.

Peak Date: August 18, 2012

Total count: 1485

Summary: Of the 1485 Weibo posts generated on August 18, 2012 that mentioned the keyword for meningitis (脑膜炎), 1475 (99.33%) were about a story about a young girl who was taken to the hospital by her parents. In a nutshell, the child was first diagnosed with suspected meningitis and was prescribed lumbar puncture to confirm or exclude the diagnosis of meningitis. And then, when the parents took the child to another department of the same hospital for the procedure, there was miscommunication between the two departments. At the end, the parents lost faith in the doctors and withdrew their consent to their daughter's lumbar puncture. They then took her to another hospital for a second opinion. The doctor there diagnosed her as not having meningitis (without lumbar puncture). The father wrote a long essay and posted it to his Weibo account as a long Weibo post (Chinese title: 杨娃娃腰椎穿刺惊魂). That post triggered an avalanche of responses on Weibo.

We identified 617 posts (41.55% of 1485) that shared similar experiences of themselves or their family or other people. The comment made by one famous writer attracted many reposts (n=479). Another famous writer's critique of that post drew many reposts too (n=161). Many criticized the doctors (n=81) and some criticized the hospital by name (n=22). Some supported the doctors and their decision to prescribe a lumbar puncture (n=41). Some appeared relatively neutral and saw the merits of both sides (n=33).

This incident provided an interesting example that demonstrated the power of Weibo to amplify the experience of one patient and her parents that drew the attention of many. The underlying social problem of the tension and mistrust between patients and the medical profession in China is the root cause of this phenomenon. Many found themselves in a similar situation. They also wanted to share their experience. They did so by re-posting the original post. The fact that certain famous writers (and Weibo opinion leaders) commented on the incident further amplified the original "signal".

Table S21. Sample Weibo entries that contained the keyword for "meningitis" on August 18, 2012.

Sample Weibo posts on that day	English translation / description	Count (%)	Sources / References / Notes	Category
**Comments on the story of Miss Yang**		**1475 (99.33%)**		
我也遭遇过，去年我头痛的厉害，呕吐不止，去天津海河医院看病，结果大夫说可能是脑膜炎，需做腰穿！当时我就吓傻了！后来爸爸带我去输液，一瓶液下去，我还是头痛欲裂，呕吐不止！这时我心里害怕了，妈妈也哭了起来！后经我当中医的姑姑确认说没事，就是中暑！后来	I had a similar experience. Last year when I was having a bad headache and vomiting, I went to Tianjin Haihe Hospital. The doctor said I may have meningitis and need to do the lumbar puncture! I was badly terrified at that time and my father took me to get transfusion. However, even after one bottle of transfusion, I still kept vomiting and having a splitting headache! Then I began to feel afraid from the bottom of my heart and my mother started to cry! Later, my aunt who is a traditional Chinese medicine doctor confirmed that I was alright	617 (41.55%)	Original Weibo post<br><br>Representative of posts that share personal or family or other's experiences similar to the story under discussion. *	Social issue (relationship between the medical profession and patients) / Personal comment

输完另两瓶液就好了！医生不可信啊！	and I was just suffering from the heatstroke! I recovered soon after having two more bottles of transfusion! Doctors are not reliable!			
根据我在医院呆的情况来看，医学就是个人判断的问题，全凭个人修养与造化。而且医学是排除科学，要一样一样排查。我真碰到感冒治两周，最后脑膜炎不治去世的。如果万一呢？家长会什么感受？会是医闹的一员吗？	According to my experience in the hospital, medicine science is a matter of personal judgment and totally depends on personal cultivation and good luck. Moreover, medicine is the science of exclusion which requires excluding all possibilities before the final judgment. I have really encountered situation when the patient was treated for two weeks as having a cold but eventually died of meningitis. What if the patient really has meningitis? How will her parents feel? Will they become part of the medicine dispute?	479<br>(32.26%)	Original Weibo post<br><br>A post from famous writer Liu Liu, and its reposts.	Social issue (relationship between the medical profession and patients) / Personal comment
如果医生能说服我，但检测结果没有脑膜炎，我会感觉很幸运，幸好没得。我以前碰到过医生让病患做脑 CT 看脑瘤，没有，病患勃然大怒，说没病你让我照干嘛？ //@[user] 六六家也有小孩，如果这事发生在你家，医生要腰椎穿刺，你会接受吗？你会成为医闹吗？	If the doctor can convince me to do the lumbar puncture for testing and I am told that I do not have meningitis, I will feel very lucky. Once, I saw a doctor asked a patient to do CT scanning to see whether there was a brain tumor and it turned out to be no. The patient burst into anger and complained that," Why did you ask me to do the CT when I am alright?"//@ [user] There is child in Liu Liu's family as well. If this happened on your own child and the doctor required him or her to get lumbar puncture, would you accept? Will you become one of those who make troubles in the hospital due to the medicine dispute?	161<br>(10.84%)	Original Weibo post<br><br>A post that questioned Liu Liu's comment and its reposts.	Social issue (relationship between the medical profession and patients) / Personal comment
什么狗屁医生，判断是否脑膜炎根本不用腰椎穿刺的，都哪跟哪啊。还特需，这种人怎么当的医生！！！//@[user] 操！	What a nonsense doctor! There is no need at all to do lumbar puncture for the judgment of meningitis. How can the world be so ridiculous! Even the specialists. How come this kind of people could have become doctors! //@[user] Damn it!	81<br>(5.45%)	Original Weibo post<br><br>A representative post of posts that were critical of the doctors or the hospital. *	Social issue (relationship between the medical profession and patients) / Personal

				comment
脑膜炎可疑当然应该腰穿，可能没有解释清楚或是程序上的问题。楼主写的太主观了，会引起误导，所幸只是感冒，万一真是脑膜炎漏诊，后悔都来不及。	It is for sure that you need to do lumbar puncture if you are suspected to have meningitis. Maybe the doctor did not explain it clearly or there were some procedural problems. The post you wrote is too subjective and can be misleading. Fortunately, the child just got a cold. If it had been a missed diagnosis of meningitis, it would have been too late to feel regretful.	33 (2.22%)	Original Weibo post<br><br>A representative post of posts that were deemed fairly neutral. *	Social issue (relationship between the medical profession and patients) / Personal comment
北京儿童医院最近第二次被这样的质疑。官方却没有任何声音。很怀疑他们的宣传部门是否吃干饭。上次同样是某儿童医院及时澄清诊疗过程，赢得了尊重，同时科普广大年轻父母。高热呕吐的孩子需要排除脑膜炎是肯定的，腰穿也没有大家想象的那样后遗症。但是缺乏医院及时解释，名人推波助澜…哈哈。	It was the second time that Beijing Children's Hospital had been questioned in this way recently. However, there has not been any official statement about them. I doubted whether their Publicity Department is really functioning or not. Last time, another Children's Hospital clarified the treatment process, which not only helped the hospital earn respect, but also popularized the knowledge among young parents. It is certain that the child who has a fever and vomits needs to be checked to exclude the possibility of having meningitis, and there is no such sequela of doing lumbar puncture as the general public perceive. But since hospitals fail to explain in time and some celebrities add fuel to the flame...Haha (i.e. These are the reasons why there are still so much misunderstanding on the meningitis and lumbar puncture) .	22 (1.48%)	Original Weibo post<br><br>A representative post of posts that raised criticisms especially against Beijing Children's Hospital*	Social issue (relationship between the medical profession and patients) / Personal comment
腰穿也没那么大的风险，损伤神经的情况更是微乎其微。这是比较传统的脑膜炎诊断方法。对于病毒性脑膜炎来说，有时候腰穿是唯一的确诊方法。当事医生处理是否有问题，只能根据病历描述的情况来判断。基本上现在比	In fact, doing lumbar puncture is not that risky and has little chance of injuring the nerve. It is a relatively traditional diagnostic method for meningitis. As for viral meningitis, sometimes doing lumbar puncture is the only way of diagnosis. Whether the doctor's handing is appropriate or not can only be judged based on the description on the medical record. Nowadays, doctors	41 (2.76%)	Original Weibo post<br><br>A representative post of posts that supported the doctor's decision to prescribe lumbar puncture for the child in the story*	Social issue (relationship between the medical profession and patients) / Personal

较保守的医生都宁愿过度检查而不愿意漏诊。医患关系不好，医生也要保护自己不是？	who are relatively conservative basically prefer over-checking than incurring missed diagnosis. Owing to the poor doctor-patient relationship, doctors need to protect themselves, right?			comment
其实判断脑膜炎有个很简单的方法:让孩子放松坐好，看下巴能否尽量贴近胸口，如果可以离的很近就基本排除脑膜炎了。每位家长在孩子高烧不退的情况下都该这么做。我妈从小就教我的~	In fact, there is a very simple way to diagnose meningitis: let the child sit comfortably and see whether he or she can press the chin close to the chest. If he or she can press it very close, then basically he or she does not have meningitis. Every parent is supposed to do so when the child has a persistently high fever. My mom taught me that when I was young~	9 (0.61%)	Original Weibo post<br><br>A representative post of posts that gave advice on the diagnostic method for meningitis. *	Social issue (relationship between the medical profession and patients) / Personal comment
脑膜炎多数 2 岁以下的儿童才容易感染，这是一个基本常识，好在你没有冲动，理智的询问了第二个意见，儿童医院的医生如此草率，实在令人气愤！为了赚钱更多的钱，简直就是草菅人命！娃儿好在有你这么一位细心的父亲！	It is a common sense that only children under 2 years old are easy to get meningitis. Fortunately, you did not act recklessly and instead asked for a second opinion sensibly. It is so infuriating that doctors in Children's Hospital are so hasty! To make more money, these doctors virtually have complete disregard for human life! You child was lucky to have a careful father like you!	7 (0.47%)	Original Weibo post	Social issue (relationship between the medical profession and patients) / Personal comment
记得以前去友谊会诊过一个发烧不退老爷爷去儿研所会诊一个发烧小朋友西医都做了腰穿排除脑膜炎，然后各种退烧最后都死了，没插上手//@[user] 吓死各位！腰穿！！！！	I remember once I went to (Beijing) Friendship Hospital for a consultation with an elderly man whose fever persisted despite treatment; once I went to Capital Institute of Pediatrics for a consultation with a case of a child who had a fever. Doctors of western medicine had done lumbar puncture to exclude the possibility of meningitis and brought down the fever. However, the patients still died eventually and I could not even help.// @[user] It is so scary to all of us! Lumbar puncture!!!!	6 (0.40%)	Original Weibo post<br><br>This comment was made by a practitioner of Traditional Chinese Medicine.	Social issue (relationship between the medical profession and patients) / Personal comment

当了妈以后就老是后悔当初干嘛不学医或者营养学啥的//@[user1]曝光//@[user2]转 //@[user3] 啥专家啊 //@[user4]转，这个绝对要曝光 //@[user5]医生都得脑膜炎了吧！这种情况我都不送医院	After becoming a mother I have always been regretting for not learning something like medicine and nutrition//@[user1] Make this public//@[user2] Forward//@[user3] What kind of expert does he think he is! //@[user4] Forward. This has to be made public //@[user5] The doctor must have got meningitis! I will not even send the patient to hospital under this circumstance	5 (0.34%)	Original Weibo post<br><br>A representative post of posts that regret oneself's lack of medical knowledge. (One original & 3 reposts; plus another original post)	Social issue (relationship between the medical profession and patients) / Personal comment
韩寒团队不懂医疗常识和常规就冒然转发这样误导大众，激化医疗矛盾的文章，以后有脑膜炎的孩子拒做腰穿而失去治疗时机，你们能负责吗？不懂的东西不要乱转发！ //@[user]无言以对，医德何在？即便需要从不同角度去了解真实病情，	Not knowing much medical common sense and practice, the team of Han Han rashly forwarded an article that misled the public and irritated medical dispute. Are you to be blamed for when there are children who miss the best time for treatment because of refusing to do lumbar puncture? Don't forward things that you don't know recklessly! //@[user] Speechless. Where has the medical ethics gone? Even if you need to know the patient's condition from different perspectives,	4 (0.27%)	Original Weibo post<br><br>A post (and its reposts) that criticizes the famous writer Han Han for reposting the story	Social issue (relationship between the medical profession and patients) / Personal comment
简简单单的感冒发烧也能被诊断成脑膜炎，真够吓人的，所幸有惊无险！所以，所以 家长们，为了我们的孩子，为了我们自己，还是学点医学知识吧！！！ //@[user] 太吓人！	It is so scary that even a simple cold and fever can be diagnosed to be meningitis. Fortunately there are near misses! Therefore, all parents, for our children and for ourselves, we had better learn some medical knowledge!!//@[user] So horrible!	3 (0.20%)	Original Weibo post<br><br>A post (and its reposts) that advocates parents to learn some medical knowledge.	Social issue (relationship between the medical profession and patients) / Personal comment
此事只能说明儿童医院的各部门间协作有问题，其实根据孩子的病情做腰穿和 CT 并没有错，而且腰穿根本就没什么风险，局麻后也不痛，单就腰穿不值得大惊	From this incident, we can only tell that there are problems on the collaboration among different departments of the Children's Hospital. There is nothing wrong about asking the child to do lumbar puncture and CT according to her condition. What's more, it is of little risk to do lumbar puncture , and the patient won't	4 (0.27%)	Original Weibo post<br><br>A representative of posts that reflect on the lack of communications between doctors	Social issue (relationship between the medical profession and patients)

小怪。普通感冒和病脑在症状上并没有绝对的区别，只是孩子最后证明是感冒，这要真是脑膜炎医生给漏诊了，到时候你看吧，家长非得把医生杀了不可	feel painful after local anesthesia. No need to panic simply because of doing lumbar puncture. There is no absolute difference in symptoms between a common cold and meningitis. If the child is misdiagnosed to have a cold, by the time you will see that parents will be so angry that they will want to kill the doctor!		in the hospital. *	/ Personal comment
脑膜炎是呈喷射状呕吐，不知道这位家长知道不知道。//@[user] 吓人	A patient with meningitis vomits in a spurting way. I am not sure whether this parent knows that. //@[user] Scary	3 (0.13%)	Original Weibo post<br><br>A representative of three posts that primarily focused on the symptoms of meningitis. *	Social issue (relationship between the medical profession and patients) / Personal comment
**Health education in general**				
春季小儿疾病早预防——幼儿保健： 春季使小儿一[转发]年中最容易得病的季节，家长应针对孩子的体质状况，有(的)放矢地做好预防工作 春季疾病大致可分以下三类： 一：呼吸道传染病：常见的如麻疹、风疹、水痘、上感、腮腺炎、流行性脑膜炎	Early prevention of childhood diseases in spring--Health care for children: Spring is the season of a year when people fall ill most easily. Parents should take some precautions with purpose based on children's physiques There are mainly three types of diseases prevailing in spring. The first type is the respiratory infectious disease that includes measles, rubella, varicella, upper respiratory infection, mumps and epidemic meningitis.	10 (0.67%)	Original Weibo post	Health education

* The category with * indicates that the sample post presented was a representative of those Weibo posts that share the same meaning with some literal differences.

22. Pertussis

Figure S22. Daily count of Weibo posts with the keyword "Pertussis" (百日咳) in 2012 among Sina Weibo users with 1000 followers or more.

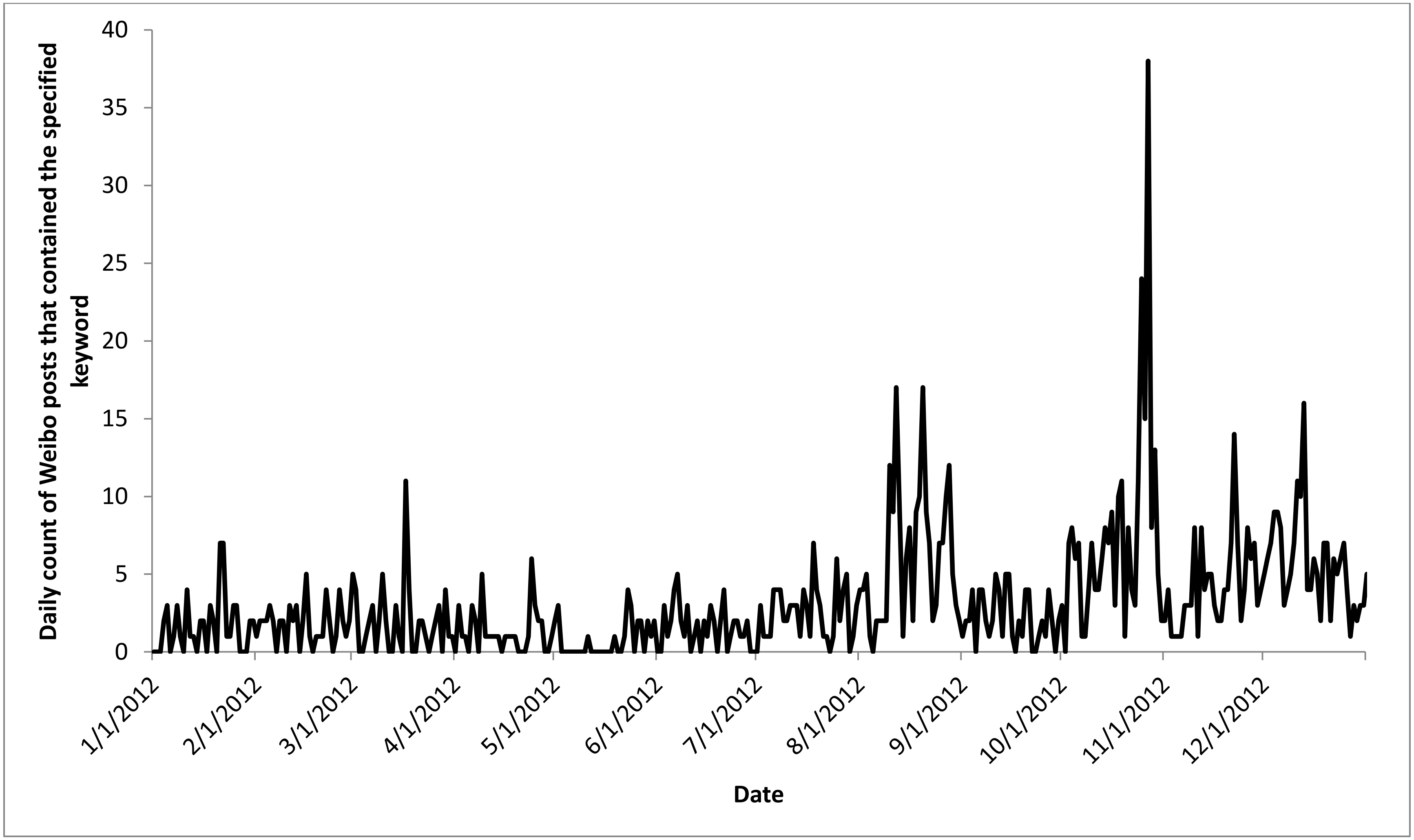

Content analysis was performed on Weibo posts of the peak date of October 27, 2012.

Peak Date: October 27, 2012

Total count: 38

Summary: There were 38 Weibo posts in our database that mentioned “Pertussis” on October 27, 2012. Of these, 15 posts were related to the recommendations of the US CDC Advisory Committee on Immunization Practice (ACIP) on Tdap (tetanus, diphtheria and pertussis) vaccine for all pregnant women (US CDC press release, dated October 24, 2012: http://www.cdc.gov/media/releases/2012/a1024_Tdap_immunization.html). Following the discussion on US CDC ACIP’s recommendations, another 8 posts were conversations related to Chinese CDC’s data on pertussis in China in 2011. There was another post that mentioned vaccination practice in Australia.

There were 14 posts on a recipe on pertussis treatment from the perspective of dietotherapy (Traditional Chinese Medicine).

Table S22. Sample Weibo entries that contained the keyword “Pertussis” (百日咳) on October 27, 2012.

Sample Weibo posts on that day	English translation / description	Count (%)	Sources / References / Notes	Category
**Conversations following US CDC ACIP recommendation on Tdap for pregnant women**		**24 (63%)**		**Health information**
孕妇们注意了。重点提示：中国百日咳没有出现类似美国的爆发 //@[user1]北京庇利积臣 Bayley Jackson 提供全面的疫苗接种服务，预约电话：(010)85629998 //@[user2]美国 CDC 咨询委员会建议：所有孕妇都应打三合一疫苗	Attention, pregnant women! Key reminder: in China, there is no outbreak of pertussis similar to that in the USA. //@[user1]Beijing’s Bayley Jackson provides full vaccination service. Booking telephone number: (010)85629998 //@[user2]US CDC Advisory Committee [on Immunization Practice]: All pregnant women should receive the 3-in-1 vaccine	15 (40%)	Original Weibo post	Health information
警惕百日咳死灰复燃！//@[user1]《2011 年度全国法定传染病报告发病、死亡统计》显示，2011 年百日咳发病数 2517 例死亡 2 例，较 2010 年发病 1764 例死亡 1 例。发病率增加 41.98% 。//@[user2]国内 CDC 的数据怎样	Beware of pertussis resurgence! //@[user1] According to the *2011 National Statistics of Incidence and Mortality of Notifiable Infectious Diseases*, in 2011 the number of incident cases of pertussis is 2517 (2 deaths), comparing with the incidence of 1764 cases in 2010 (1 death), the incident rate increases by 41.98%. //@[user2] What about data from China CDC?	8 (21%)	The statistics can be found at: http://www.yncdc.cn/inInfoNewsView.aspx?varCatyID=021407&newsid=95550; or: http://wendang.baidu.com/view/9ad547f64693daef5ef73d81.html<br><br>Also, see: http://weibo.10086.cn/feed/single.php?feedid=111821073678663&type=reply	Health information

在澳洲，出院前医院就免费给爸爸妈妈打百日咳疫苗。姥姥姥爷可以去 gp 那打，也是免费的。	In Australia, before discharge, hospitals vaccinate father and mother for free. Grandmother and grandfather can get vaccinated at GP [General Practitioners] that is also free of charge.	1(2%)	Original Weibo post	Health information / Personal comments
**Dietotherapy / Traditional Chinese Medicine**		**14 (37%)**		
【小儿百日咳的食疗】冰糖鸭蛋、冰糖 50 克，加热水适量把糖搅拌溶化，待冷，打入鸭蛋 3 个，调匀，放蒸锅内蒸熟。1 次或分次食用，每日 1 次。用于本病后期出现干咳无力、面红唇干、烦躁盗汗、手足心热等肺阴不足症。	【Young children pertussis dietotherapy】 Rock sugar duck egg, rock sugar 50g. Add adequate amount of hot water to dissolve the rock sugar. That it cool down. Then add three duck egg. Mix thoroughly. Put them in a steaming pan to steam until they are done. Serve once or in parts (once a day). To use it during the later stage of this disease when the patient presents with dry cough and weakness, red face and dry lips, irritability and night sweats, feverish palms and soles, and other "Lung *Yin*" deficiency symptoms.	14 (37%)	http://zhan.renren.com/cadcax?gid=3674946092082906649&checked=true	Alternative health information / Traditional Chinese medicine

23. Diphtheria

Figure S23. Daily count of Weibo posts with the keyword for "Diphtheria" (白喉) in 2012 among Sina Weibo users with 1000 followers or more.

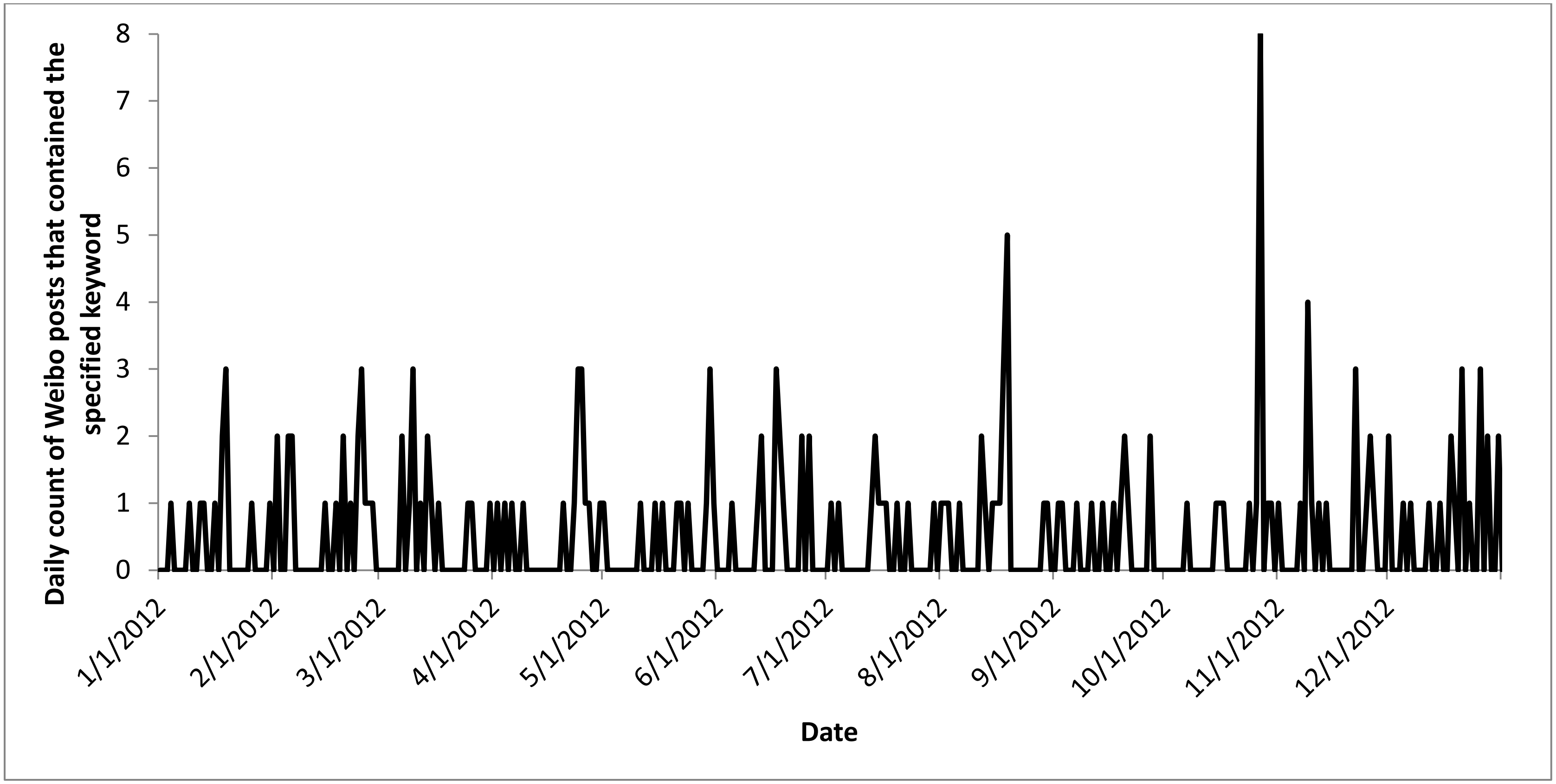

Content analysis was performed for Weibo posts on the peak date of October 27, 2012.

Peak Date: October 27, 2012
Total count: 8

Among the 8 Weibo posts containing the keyword for "diphtheria" (白喉) generated on October 27, 2012, 7 posts were related to the recommendations made by the Advisory Committee of Immunization Practice (ACIP) of the United States Centers for Disease Control and Prevention (source: http://www.cdc.gov/media/releases/2012/a1024_Tdap_immunization.html). In October 2012, ACIP recommended all pregnancy women to receive Tdap (tetanus, diphtheria and pertussis) 3-in-1 vaccine among pregnancy women. A Chinese hospital took the opportunity to emphasize that this recommendation was also practiced by them. It is interesting to note that CDC statistics about the resurgence of pertussis cases in the US was quoted directly from the CDC press release. However, the Weibo quote did not mention that the case count statistics was for the US only.

Table S23. Sample Weibo entries that contain the keyword "Diphtheria" on October 27, 2012.

Sample Weibo posts on that day	English translation / description	Count (%)	Sources / References / Notes	Category
**CDC ACIP recommendation on Tdap and pregnancy women**		7 (88%)		
美国 CDC 预防接种咨询委员会（ACIP）建议：妇女每次怀孕都应该接种 Tdap（破伤风, 白喉, 百日咳）三合一疫苗。咨询委员会称，孕妇接种百日咳疫苗是安全的，而且所有孕妇都必须接种该疫苗，最佳时间是在怀孕的最后三个月。@疫苗与科学	United States CDC Advisory Committee on Immunization Practice (ACIP) recommends that for every pregnancy, women should receive Tdap (tetanus, diphtheria and pertussis) 3-in-1 vaccine. The advisory committee said that it is safe for pregnant women to receive the *Penis et Testis Canis* vaccination against pertussis and all pregnant women should receive that vaccine. The best timing is the last trimester of pregnancy. @Vaccine and Science	5 (63%)	http://www.cogonline.com/Article/zyyd/lcjz/143048023.html	Health education (CDC ACIP recommendation)
在我院，所有孕妇都要接种 Tdap（破伤风,白喉,百日咳）三合一疫苗，时间是预产期前三个月内。提前警惕！ //@[user]根据 CDC 统计，到今年 10 月 15 日已有超过 32,000 百日咳病例。有 16 人因百日咳而死亡，大多数是小于 3 个月的婴儿，这是自 1959 年以来病例最多的一年	In our hospital, all pregnant women must receive Tdap (tetanus, diphtheria and pertussis) 3-in-1 vaccine. The timing is within 3 months of the expected date of birth. Beware in advance! //@[user]According to CDC statistics, up to October 15 this year, there had been 32,000 pertussis cases, of whom 16 died of pertussis, of whom the majority were babies under the age of 3 months. This is the year of the highest incidence since 1959.	2 (25%)	http://www.weibo.com/2349800145/z2oFbg1VX<br><br>News reference: http://m.epochtimes.com/article.php?url=/gb/12/10/26/n3714854&encode=gb&back=1&	Health information
**Other topics**				
一亩松柏林，一昼夜能分泌出 2 千克	An acre of forest of pine and cypress, can secrete 2kg	1 (12%)	http://www.360doc.com/conten	Alternative

杀菌素，可杀死肺结核、伤寒、白喉、痢疾等病菌。 一亩阔叶林，一年可蒸发 300 多吨水。因此，森林多的地区，常常是风调雨顺。	of phytoncide in a day that can kill germs that cause tuberculosis, typhoid, diphtheria, and dysentery etc. An acre of broadleaf forest, can evaporate 300 tons of water per year. Therefore, regions where there are plenty of forests, the weather is often very good.		t/11/0819/15/3802186_141705551.shtml	health information

24. Tetanus

Figure S24. Daily count of Weibo posts with the keyword for “Tetanus” in 2012 among Sina Weibo users with 1000 followers or more.

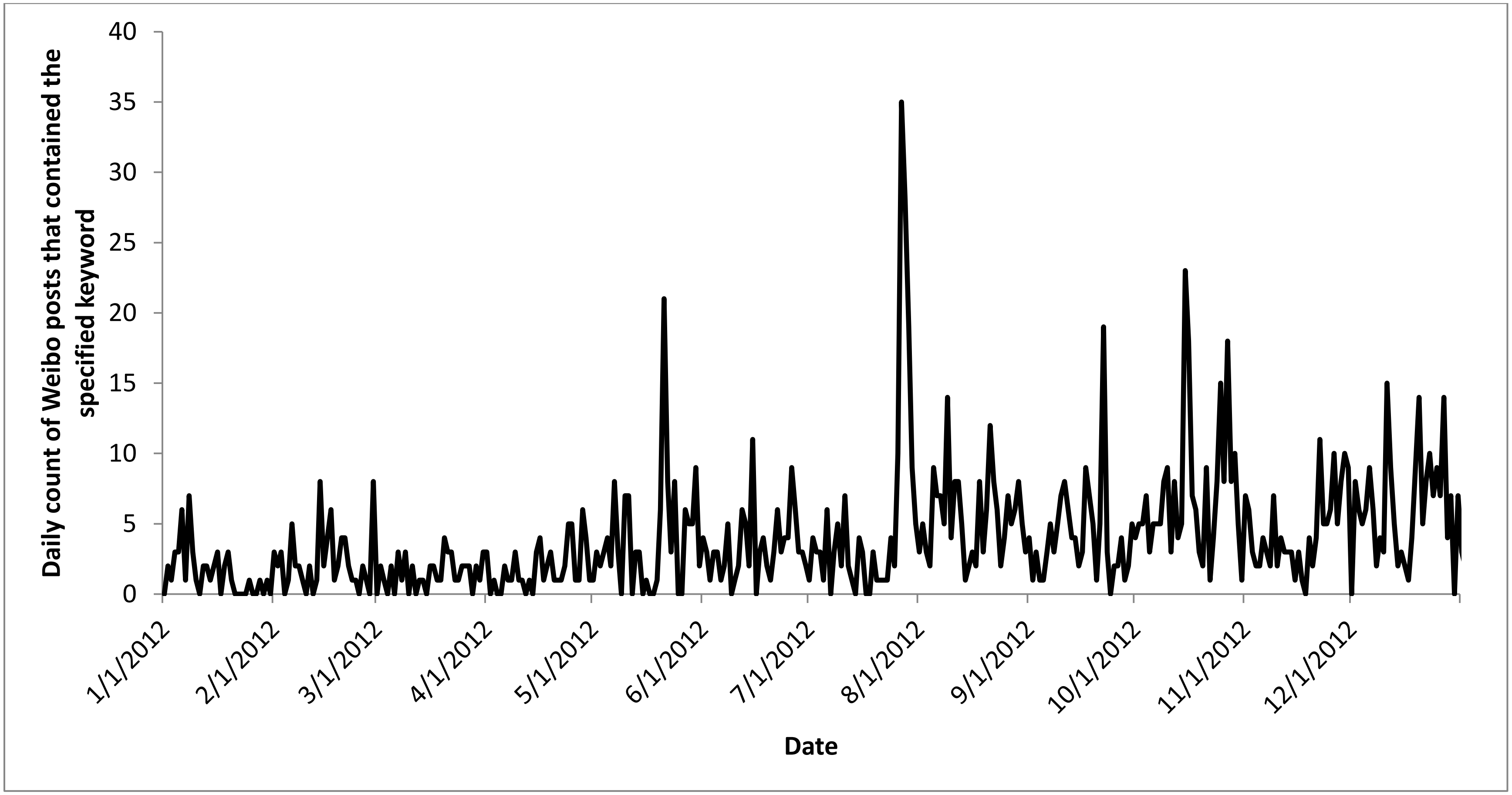

Content analysis was performed for Weibo posts generated on the peak date of July 27, 2012.

Peak Date: July 27, 2012
Total count: 35

Summary: Of the 35 Weibo posts that were generated on July 27, 2012 that mentioned the keyword for "tetanus" (破伤风), 31 posts were related to wound management. Four other posts were non-medically related.

Table S24. Sample Weibo entries that contained the keyword "tetanus" (破伤风) on July 27, 2012.

Sample Weibo posts on that day	English translation / description	Count (%)	Sources / References / Notes	Category
【三种伤口不能用创可贴】①被动物(如猫、狗)咬伤后。应先用肥皂水冲洗多次，然后用碘酒、酒精消毒伤口，让伤口暴露，并注射狂犬疫苗。②深而窄的伤口，如铁钉、刀尖扎伤等。止血后应当使伤口暴露，以防破伤风菌感染伤口。③烧伤、烫伤后，出现的溃烂、渗液，也不能用创可贴来贴，以免引起继发感染。	"Should not use Band-Aid for three types of wounds" (1) After animal bites (such as cats or dogs). Should use soap water to watch multiple times, and then use tincture of iodine and alcohol to disinfect the wounds and then expose them and receive vaccination against tetanus. (2) Deep and narrow wounds, such as those pricked by an iron nail or the tip of a knife. After stanching the bleeding, should let the wound exposed to keep them from being infected with tetanus. (3) After burns or scalds, ulceration and exudates appears. To avoid secondary infection, band-aid cannot be applied.	31 (86%)	http://blog.sina.com.cn/s/blog_a6d9a1940101njad.html	Health education / information
**Other posts**				
【哪些毛病自毁运势？】1.嘴称"食禄宫"，主食禄之气，常唉声叹气，叫苦连连绝无食禄！2.身体到处打洞穿孔，相法首重身相定格。身相穿洞受损自然破坏身体健康与运势，同时易患感染细菌、破伤风等疾病。3.经常眨眼睛为"神散"，主"人无真运，晦气连连"。	[What type of defects can destruct one's own luck?] …. 2. To make holes everywhere on the body. … Making the holes in the body damage the body's Xiang naturally damage one's health and fortune, and at the same time, make it susceptible to bacteria or diseases such as tetanus….. <br>[Only the sentence relevant to tetanus is translated.]	3 (9%)	http://www.kaixin001.com/repaste/17264743_8663301219.html	Comments about luck / fortune
有贪念不坏，它至少可以告诉你什么是留恋。会让你害怕失去与滞后，会让你在后悔之前先享受占有。我爱一个人，愿她好，最好的选择是跟我好。如果不是，那爱情的贪念就会变成一场破伤风，听来很可怕，可是你任之不管，你也会好。只是，时间的长短罢了。	To have greed is not too bad. At least it tells what is longing. It let you fear the loss and the lag. It let you occupy before you regret. I love a person and wish her well. The best option is to be with me. If not, the greed of love will turn into tetanus. It sounds terrifying. But if you leave it untouched, you will get well too. It is just a matter of time.	1 (5%)	**http://blog.sina.com.cn/s/blog_815a7b940101911l.html**	Personal comment (analogy between love and tetanus)

25. Scarlet Fever

Figure S25. Daily count of Weibo posts with the keyword for "Scarlet Fever" (猩红热) in 2012 among Sina Weibo users with 1000 followers or more.

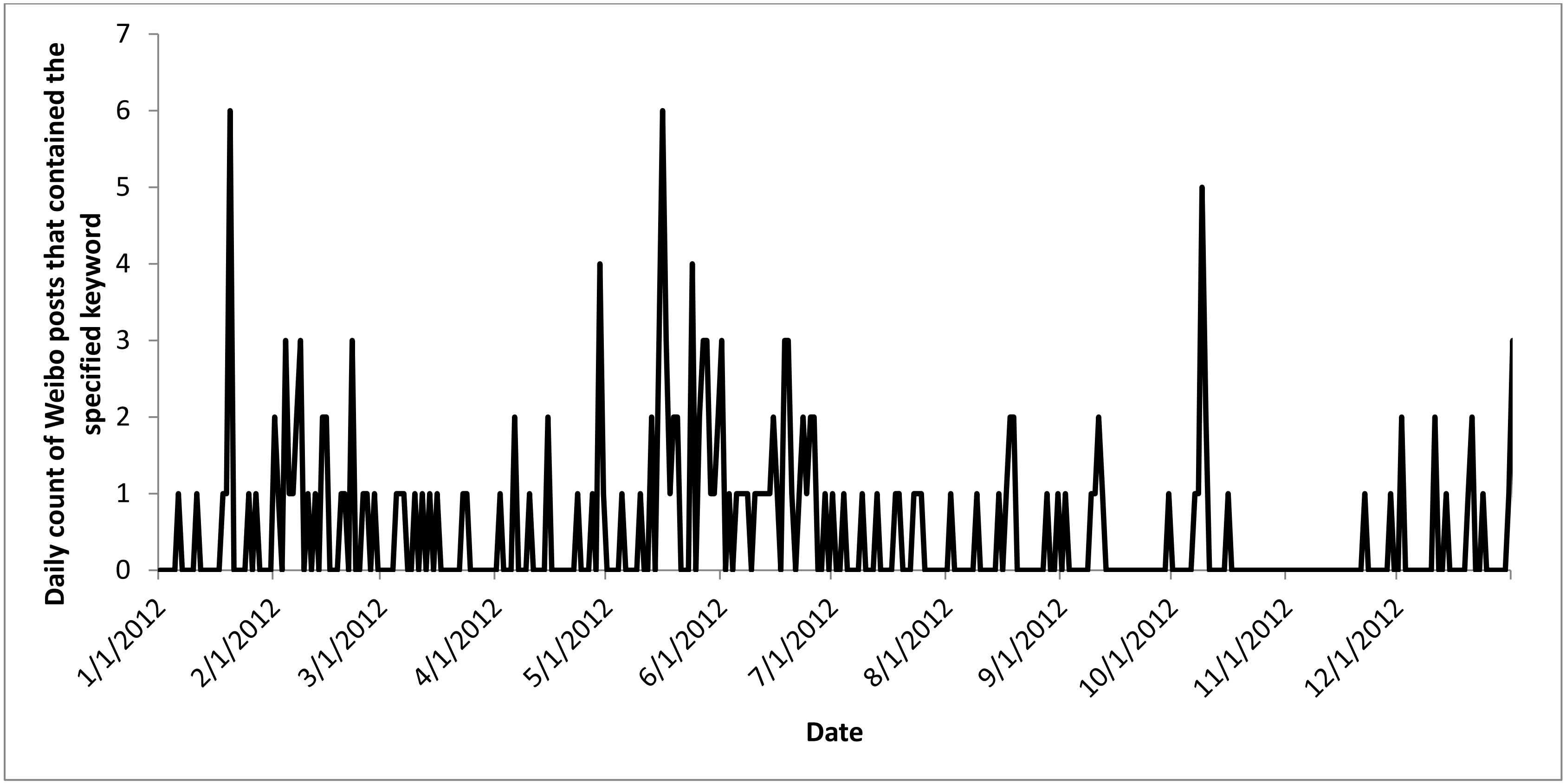

Content analysis was performed on Weibo posts generated on the two peak dates of January 20, 2012 and May 16, 2012.

Peak Date: January 20, 2012

Total count: 6

Summary: Five of the 6 Weibo posts on January 20, 2012 that mentioned “scarlet fever” were about a fatal case of scarlet fever in Hong Kong. The victim was a 14-year-old girl.

On May 16, 2012, all 6 Weibo posts mentioning “scarlet fever” were health education about how to take care of young children felling sick of scarlet fever.

Table S25A. Sample Weibo entries that contained the keyword “Scarlet Fever” (猩红热) on January 20, 2012.

Sample Weibo posts on that day	English translation / description	Count (%)	Sources / References / Notes	Category
**News about two cases of scarlet fever in Hong Kong**				
《猩红热夺女童命 不排除#病毒#或变种》 香港一名 14 岁少女证实染猩红热夺命，另一名 11 岁男童确诊感染猩红热，正于东区医院深切治疗部留医。卫生防护中心指 9 成猩红热患者为 10 岁以下儿童，认同两病例罕见，不排除病毒或变种，亦可能有抗药性，会循多方向调查。http://t.cn/z0k7Rw8	“Scarlet Fever kills a girl The possibility of mutation cannot be excluded” A 14-year-old girl in Hong Kong died of confirmed scarlet fever. Another 11-year-old boy has been confirmed of scarlet fever infection, and is in the intensive care unit in Eastern District Hospital. Centre for Health Protection says that 90% of scarlet fever cases are children under the age of 10. They agree that these two cases are rare. They cannot rule out the possibility that the virus has mutated and become drug resistant. They will investigate at all directions. http://t.cn/z0k7Rw8	5 (83%)	http://news.xinhuanet.com/gangao/2012-01/20/c_122611575.htm	News about two cases
**Other posts**				
生病宝宝快点号起来！不要被猩红热感染阿！😔 i m worry about u coz i m not near u.快点好起来快点变吃货！明天汉和韩烧不要浪费噢。回来带我吃火锅。然后尽量回香港然后回武汉！! 我知道宝宝会带好多礼物给我的回来时候，所以你要快点好起才又力气 shopping&carry the goods 4 me□	Sick baby gets well soon! Don’t get infected with scarlet fever! 😔 i m worry about u coz i m not near you. Get well soon!.... Don’t waste [the opportunity to dine at] Hon Wo Korean Restaurant tomorrow. After your return, bring me to have hot pot. And then try your best to return to Hong Kong and then return to Wuhan!! I know that when you return, baby, you will bring lots of presents. So you need to get well soon, so that you will have the energy to go shopping & carry the goods for me!	1 (17%)	Original Weibo post	Personal comment

Peak Date: May 16, 2012

Total count: 6

Table S25B. Sample Weibo entries that contained the keyword "Scarlet Fever" on May 16, 2012

Sample Weibo posts on that day	English translation / description	Count (%)	Sources / References / Notes	Category
【如何护理猩红热患儿】1.居室通风，让患儿隔离独居；2.吃营养丰富、富含维生素的流质或半流质食物，发热患儿多饮水；3.用淡盐水漱口，一日 3~4 次，清除鼻腔分泌物，用青霉素软膏涂口唇和鼻腔；4.不要用手剥脱皮屑；5.注意观察尿液颜色、尿量、面部是否红肿等。	"How to take care of ill children infected with scarlet fever" 1. Keep the room ventilated. Keep the sick child in isolation; 2. Take food in liquid or semi-liquid form that is nutritious and rich of vitamin. Feverish sick child should drink more water; 3. Use slightly salted water for mouthwash, 3-4 times a day, to remove mucous in the nasal cavity. Apply penicillin cream to the lips and nasal cavity; 4. Do not use hands to remove dead skin cells; 5. Pay attention to observe the color and volume of urine and whether the face is red and swollen etc.	6 (100%)	http://news.sina.com.cn/h/news/2011-06-25/092722703918.shtml	Health education

26. Brucellosis

Figure S26. Daily count of Weibo posts with the keyword for "Brucellosis" (布鲁氏菌病) in 2012 among Sina Weibo users with 1000 followers or more

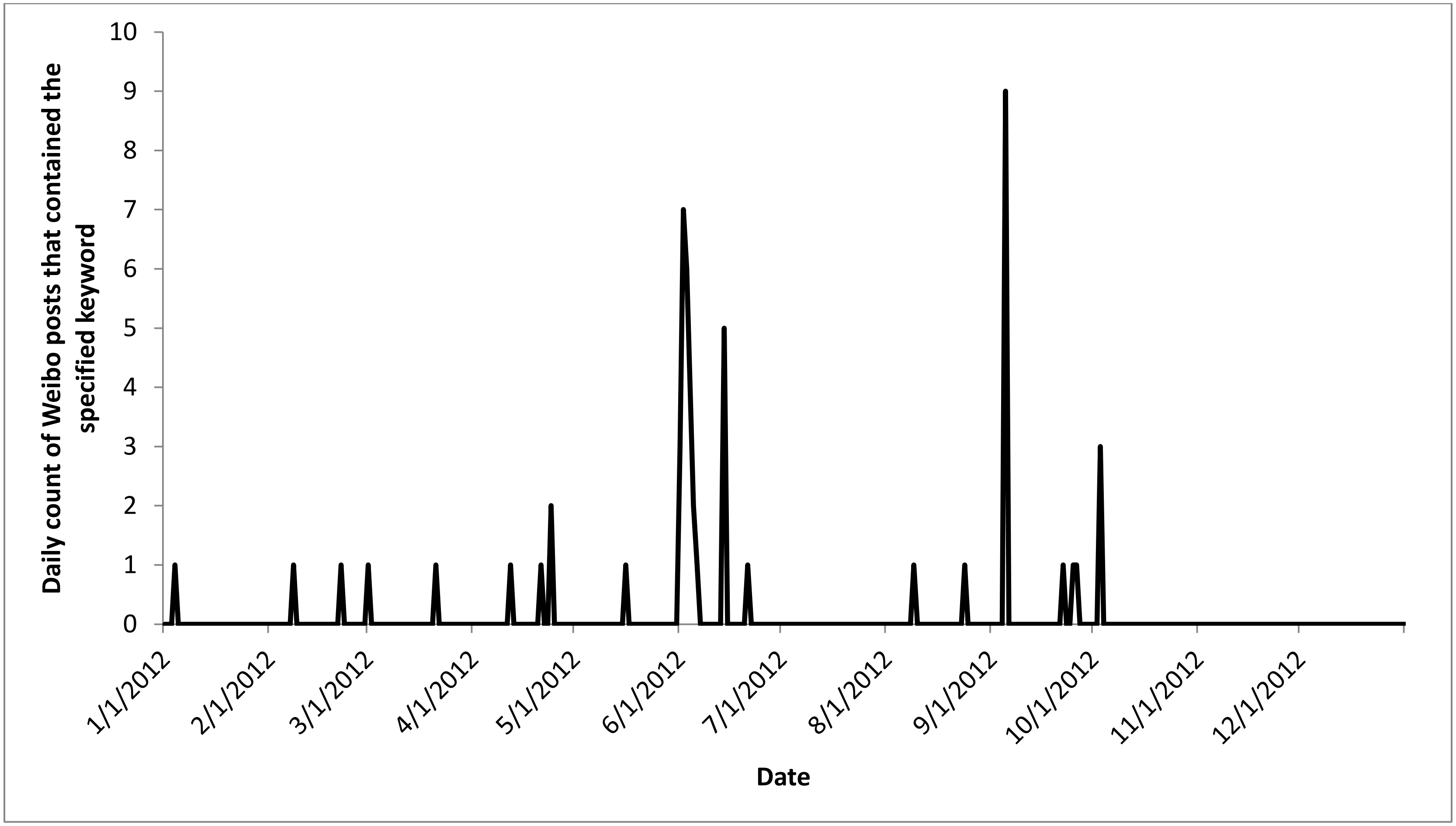

Content analysis was performed for Weibo posts generated on the peak date of September 5, 2012.

Peak Date: September 5, 2012

Total count: 9

Summary: All of the 9 Weibo posts generated on September 5, 2012 that carried the keyword for "Brucellosis" (布鲁氏菌病) were a health education post and its reposts that communicated the symptoms of brucellosis. They targeted pet dog owners and focused on accidental human infection with *Brucella canis*.

Table S26. Sample Weibo entries that contained the keyword for "Brucellosis" (布鲁氏菌病) on September 5, 2012.

Sample Weibo posts on that day	English translation / description	Count (%)	Sources / References / Notes	Category
#健康知识#【养犬提防布鲁氏菌病】如果不小心感染了犬型布鲁氏菌后发病的症状是多种多样的。急性感染的主要表现为波浪热（表现为 2~3 周的发热期，间歇 1~2 周再度发热，如此反复）、寒战、全身不适、体重减轻，还可出现头痛、恶心、淋巴结肿大、肌肉以及关节酸痛，还可能引起眼睛损伤和心内膜炎。	#Health knowledge# "Keeping dogs; Beware of brucellosis." If you accidentally infected with *Brucella canis*, there will be a variety of clinical symptoms. The major symptoms of acute infection are repeated waves of fever (presented as repeated episodes of 2-3 weeks of pyrexia; relapsing after 1-2 weeks of intermittence), chills, malaise, and weight loss. Other symptoms include headache, nausea, swollen lymph nodes, muscle and joint pain, and potential eye damage and endocarditis.	9 (100%)	http://petbird.tw/article7837.html	Health education

27. Gonorrhea

Figure S27. Daily count of Weibo posts with the keyword for "Gonorrhea" in 2012 among Sina Weibo users with 1000 followers or more.

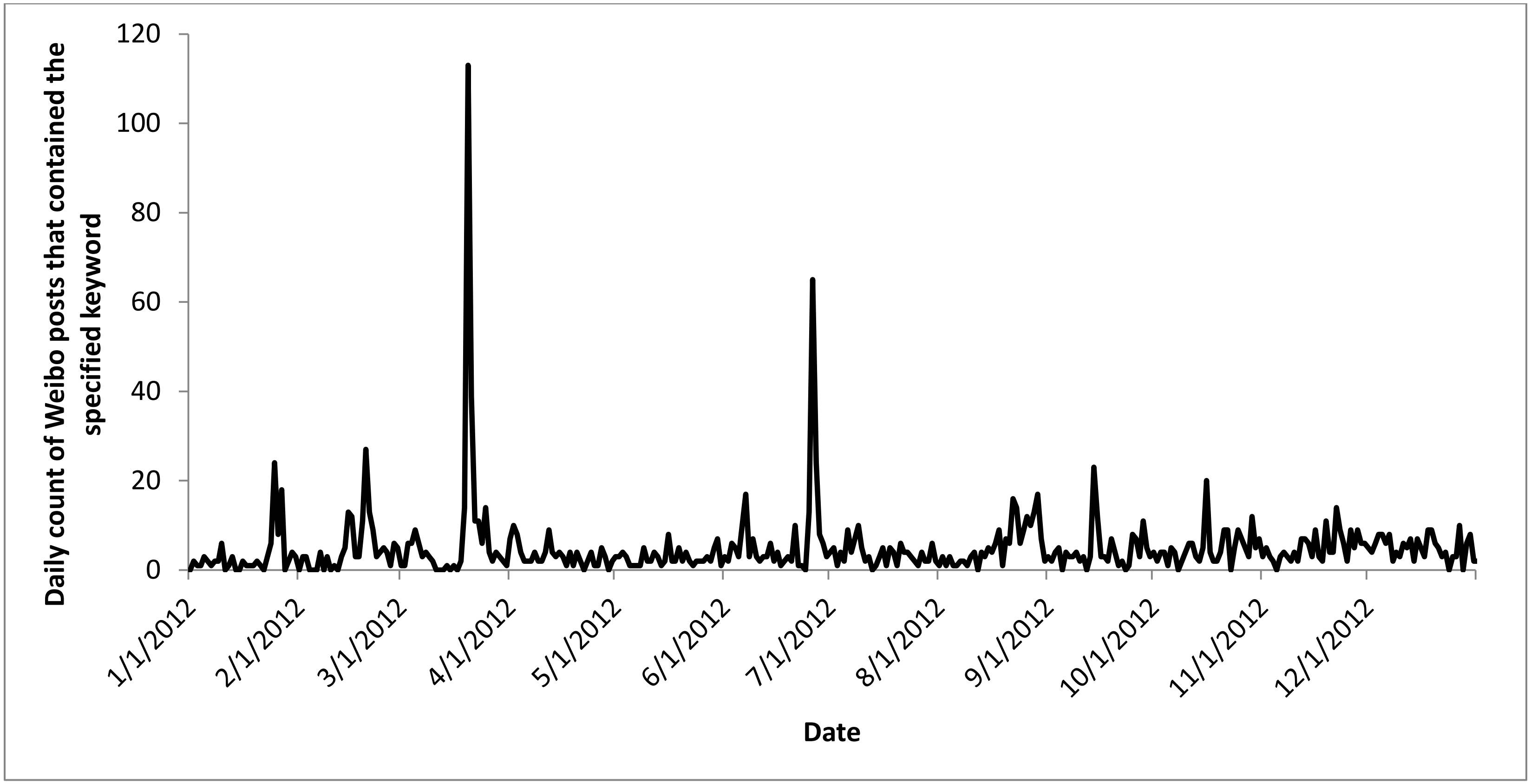

Content analysis was performed for Weibo posts generated on the peak date of March 20, 2012.

Peak Date: March 20, 2012

Total count: 113

Summary: In our database, there were 113 Weibo posts mentioning “gonorrhea” on March 20, 2012. Of these 113 posts, 62 were related to the alleged indirect gender discrimination by the Chinese government as the Chinese government required all female civil servant recruits to undergo a body check to see if they have been infected with sexually transmitted infections, such as gonorrhea. There were another 45 posts about the news of a 6-year-old girl infected with gonorrhea. Doctors suspected that the girl might have been sexually harassed.

Table S27A. Sample Weibo entries that contained the keyword “Gonorrhea” on March 20, 2012.

Sample Weibo posts on that day	English translation / description	Count (%)	Sources / References / Notes	Category
**News about body check for female civil servant recruits**				
【女公务员录用须查性病报告月经史】中国《公务员录用体检通用标准（试行)》规定，女性公务员体检须检查淋病、梅毒等性病，并搜集体检者月经史。部分体检者对此表示不满，认为隐私被侵犯。北京一家公益机构致信政府部门，建议取消该项检查，称检查性病损害女性人格尊严，造成间接歧视	“Female civil servant recruits must go through body check for sexually transmitted infection and report their menstrual history.” The *(Tentative) General Standard of Body Check for the Recruitment of Civil Servants* of China specifies that female civil servant recruits must undergo body check to inspect if they are infected with sexually transmitted diseases such as gonorrhea and syphilis and to collect their menstrual history. Some of the individuals who underwent the body check resented, arguing that their privacy had been infringed. A charity in Beijing had sent a letter to the government authorities, suggesting that this inspection should be withdrawn, as inspecting for sexually transmitted infections is damaging the dignity of females leading to indirect discrimination.	62(55%)	*Jing Hua Shi Bao*（京华时报）http://news.163.com/12/0320/04/7T0TO73O00011229.html	Social issue: Discussion about gender equality / discrimination
**News about a 6-year-old girl infected with gonorrhea**				
【6 岁女童在幼儿园被发现感染性病】近日，湖南株洲一名 6 岁女童在幼儿园感染了急性淋病，经检查，医生表示，女童遭猥亵可能是病因。女童告诉记者，曾在幼儿园和一个小男孩玩过脱衣	“6-year-old girl was found infected with sexually transmitted diseases.” Recently, a 6-year-old girl in Zhuzhou, Hunan, was found infected with acute gonorrhea in a kindergarten. After inspection, doctors said that the potential cause might have been that the girl had experienced sexual harassment. The girl told the journalist that she had played a game of taking off clothes with a small boy in the kindergarten. The	45 (40%)	China National Radio Net（中广网） http://t.cn/zOJijM5	News about a case of a little girl infected with gonorrhea

服的游戏。幼儿园方面表示，所有老师体检都没有问题。目前，事情真相还在进一步调查中。	kindergarten said that all the teachers had passed the body check without any problems. At the moment, the truth of the incident is still under investigation.			
**Sexually explicit joke**				
超搞笑啊！!! 史上最强悍的淋病解释：“细佬给淋、烫到了，不是叫鸡~~~不能和你XXOO..... ”真糸一百岁唔死都有新闻睇啊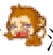淋你妹啊~~~ 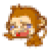	[Sexually explicit joke –we left it untranslated here.]	4 (3%)	http://t.cn/a14iqo	Joke
**Not related to gonorrhea**				
下雨了，别忘了打伞，湿身是小，淋病就麻烦啦！	[Not translated as it is not related to gonorrhea.]	2 (2%)	Original Weibo post	Not related to gonorrhea *

* Note: Even though the same keyword 淋病 appear in the posts, the words here actually mean something entirely different from gonorrhea. This is an example that nuances in language may reduce the specificity of our detection methods.

28. Syphilis

Figure S28. Daily count of Weibo posts with the keyword for "Syphilis" (梅毒) in 2012 among Sina Weibo users with 1000 followers or more.

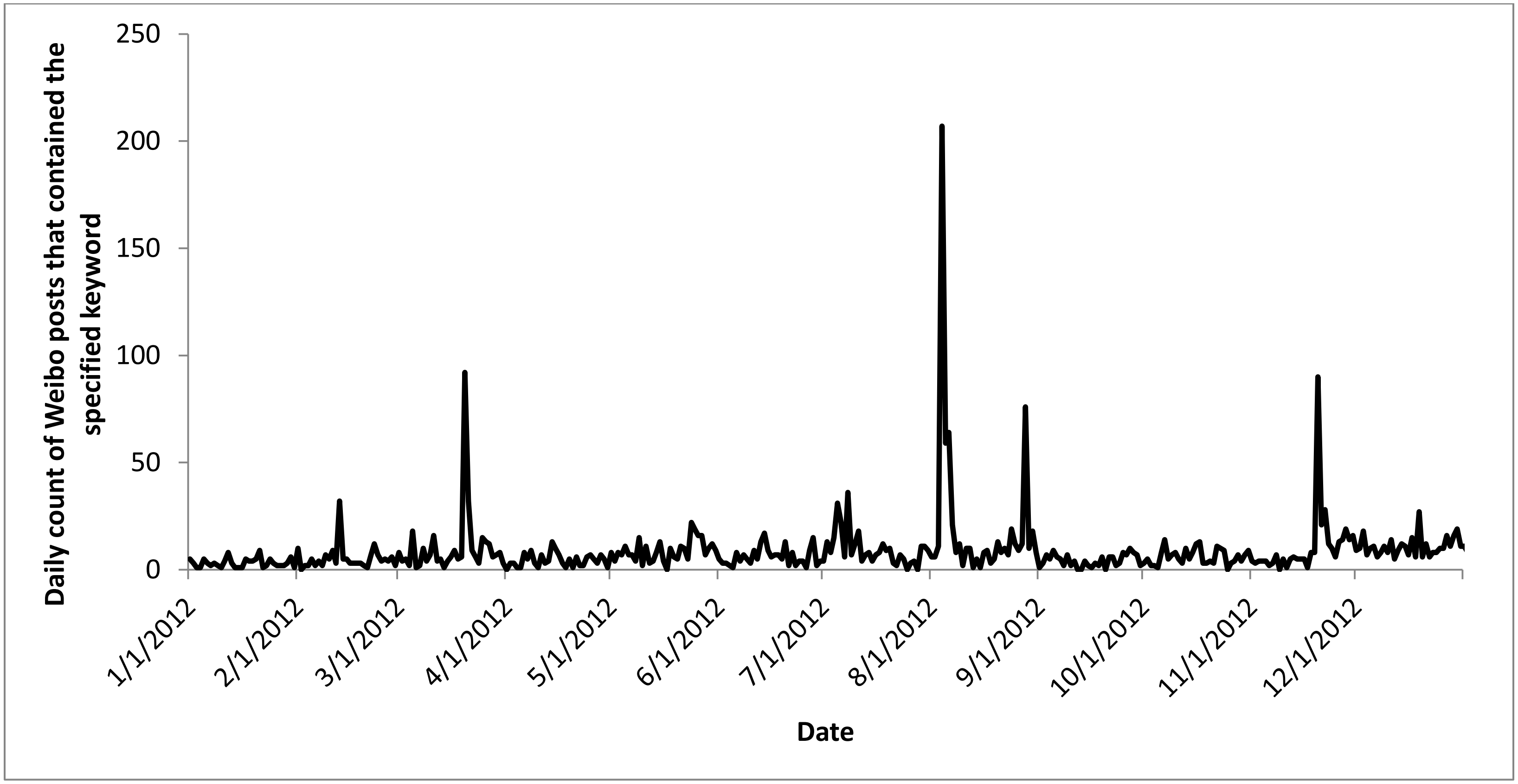

Content analysis was performed for Weibo posts generated on the peak date of August 4, 2012.

Peak date: August 4, 2012
Total count: 207

Summary: There were 207 Weibo posts containing the keyword for "syphilis" (梅毒) on August 4, 2012. Of these, 168 were related to a case of denial civil servant employment due to a positive syphilis test. Tang Haiqing ranked number one for a Chinese government position that she applied. Unfortunately, she was repeatedly tested positive for syphilis in hospitals specified by the authorities. However, when she sought independent tests from other hospitals, she was found negative. She posted her negative results online and was rebutted by her Communist Party superior.

Another 39 posts were about a case of an elderly who had been tested for syphilis and HIV 6 times during his episodes of hospitalizations.

Table S28. Sample Weibo entries that contained the keyword for "Syphilis" (梅毒) on August 4, 2012.

Sample Weibo posts on that day	English translation / description	Count (%)	Sources / References / Notes	Category
**A case of denial of employment due to a positive test result of syphilis**		**168 (81%)**		Social issue
【贵州省委称选调生头名患梅毒曾复查确认】贵州省委组织部有关负责人日前针对选调生被查出梅毒遭拒录换医院检测为阴性事件作出回应，负责人表示，此前根据考生申请按规定进行复查，考生对复查结果仍提出质疑，为对考生负责，又在其他医院进行检查，结果仍然为阳性。<br>唐海情在贵州选调生招考中报考碧江区检察院成绩第一，却被拒绝录用，原因是"得了梅毒"。但她到另三家医院检验均显示指标呈阴性，她把检测结果发上网，当天下午便接到省委组织部电话。领导批评其发帖行为：是党员应该相信党组织.	"The Communist Party Committee of Guizhou claimed that the top candidate of *Xuandiao sheng** was infected with syphilis and was confirmed again in a review." The responsible officer of the Organizational Department of Communist Party Committee of Guizhou Province responded to a recent case that a Xuandiao sheng was found syphilis-positive and was declined employment and then was found negative in another hospital. The responsible officer said that the candidate followed the regulations to apply for a review. The candidate was still skeptical of the review results. In order to be accountable to the candidate, they performed tests in other hospitals, and the results remained positive.<br>Tang Haiqing ranked number one in the Xuandiao sheng examination for the Procuratorate of Bijiang District, but was declined employment. The reason was "infected with syphilis". However, she went to another three hospitals for examination and all the tests were found negative. So, she uploaded the results online. In the same afternoon, she received a phone call from the Organizational Department of the Provincial Committee. The leader criticized her action of posting her test results online: A party member should	89 (43%)	http://news.ifeng.com/mainland/detail_2012_08/05/16566559_0.shtml	Social issue: Loss of public trust of the hospitals and the government

	believe in the Party Organization.			
裆说列宁没梅毒，列宁同志就没梅毒。 //@[user1] 组织上说你有梅毒，你就必须有梅毒！ //@[user2] 党需要你得梅毒，你必须服从组织安排。	The Party said Lenin did not have syphilis, so Comrade Lenin did not have syphilis. //@[user1] The Organization says that you have syphilis, so you must have syphilis! //@[user2] The Party needs you to have syphilis, so you must obey the arrangement of the Organization.	79 (38%)	http://t.cn/zW0ZOCy	Social issue: Sarcasm about the Communist Party
**Repetitive HIV tests for an elderly**		**39 (19%)**		
【8 旬老人患冠心病 3 家医院竟做六次艾滋检查】"我都八十多岁的人了，既不吸毒，也不存在任何不正常的性关系，本本分分做人，这些医院为什么反反复复、给我检查六遍艾滋病和梅毒？而且三次住院，每次都让我做上百项检查，很多还是重复的，这不是折腾人吗！"……via:吉和资讯	"An elderly in the 80s had coronary heart disease 3 hospitals performed 6 HIV tests" "I am over 80 years old. I do not take drugs and I do not have any abnormal sexual relationships. I am a responsible person. Why would these hospitals repeatedly perform HIV and syphilis tests for me? I was hospitalized three times. Each time, they performed over 100 tests on me, of which many were repetitive. Wasn't it tormenting?!"…… via: Jihe Information	39 (19%)	http://t.cn/zWpeOAi	Mistrust between medics and patients: Suspicion of unnecessary medical examinations

* *Xuandiao Sheng* (选调生) are the elite recruits of the Chinese government. They are university graduates with high levels of academic achievements and political loyalty. Compared to regular civil servants, they are on a fast track in their career progression. After a number of years of training experiences on the grass-root level, they will soon be promoted to higher levels of administration in the government.

29. Leptospirosis

Figure S29. Daily count of Weibo posts with the keyword for “Leptospirosis” (钩端螺旋体病) in 2012 among Sina Weibo users with 1000 followers or more.

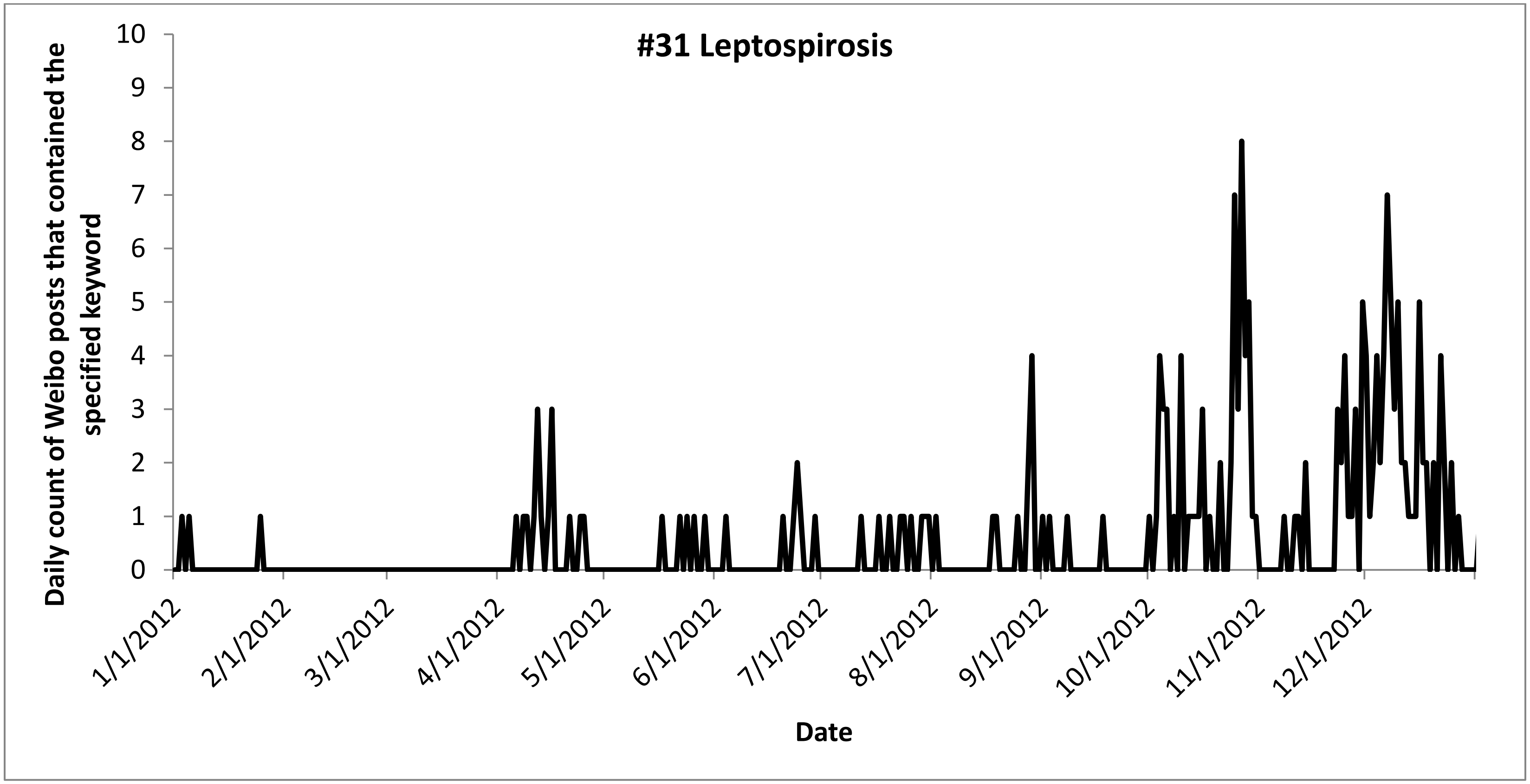

Content analysis was performed for Weibo posts generated on the peak date of October 27, 2012.

Peak Date: October 27, 2012
Total count: 8

Summary: Eight Weibo posts on October 27, 2012 mentioned “leptospirosis”. They were all related to a health education Weibo post that provided basic medical information about this disease.

Table S29. Sample Weibo entries that contained the keyword for “Leptospirosis” (钩端螺旋体病) on October 27, 2012.

Sample Weibo posts on that day	English translation / description	Count (%)	Sources / References / Notes	Category
【常见人犬共患传染病】钩端螺旋体病：几乎所有哺乳动物都可感染钩端螺旋体病。狗、兔是钩端螺旋体的宿主。人接触了患畜尿、血组织或环境中的钩端螺旋体都可发病。潜伏期大约 10 天，随后出现败血症特征，头痛、疲劳、肌肉酸痛、发热、黄疸和有血尿等症状。	“Zoonotic infectious diseases common in both human and dogs” Leptospirosis: Nearly all mammals can be infected with leptospirosis. Dogs and rabbits are host of Leptospira. After human contact with Leptospira in the urine, blood tissue or the environment, they may fall ill. Incubation period lasts for about 10 days. Afterwards, the characteristics of septicemia will appear: headache, fatigue, muscle aches, fever, jaundice, hematuria and other symptoms.	8(100%)	http://pet.pclady.com.cn/dog/health/0911/473384_all.html	Health education

30. Schistosomiasis

Figure S30. Daily count of Weibo posts with the keyword for "schistosomiasis" (血吸虫病) in 2012 among Sina Weibo users with 1000 followers or more.

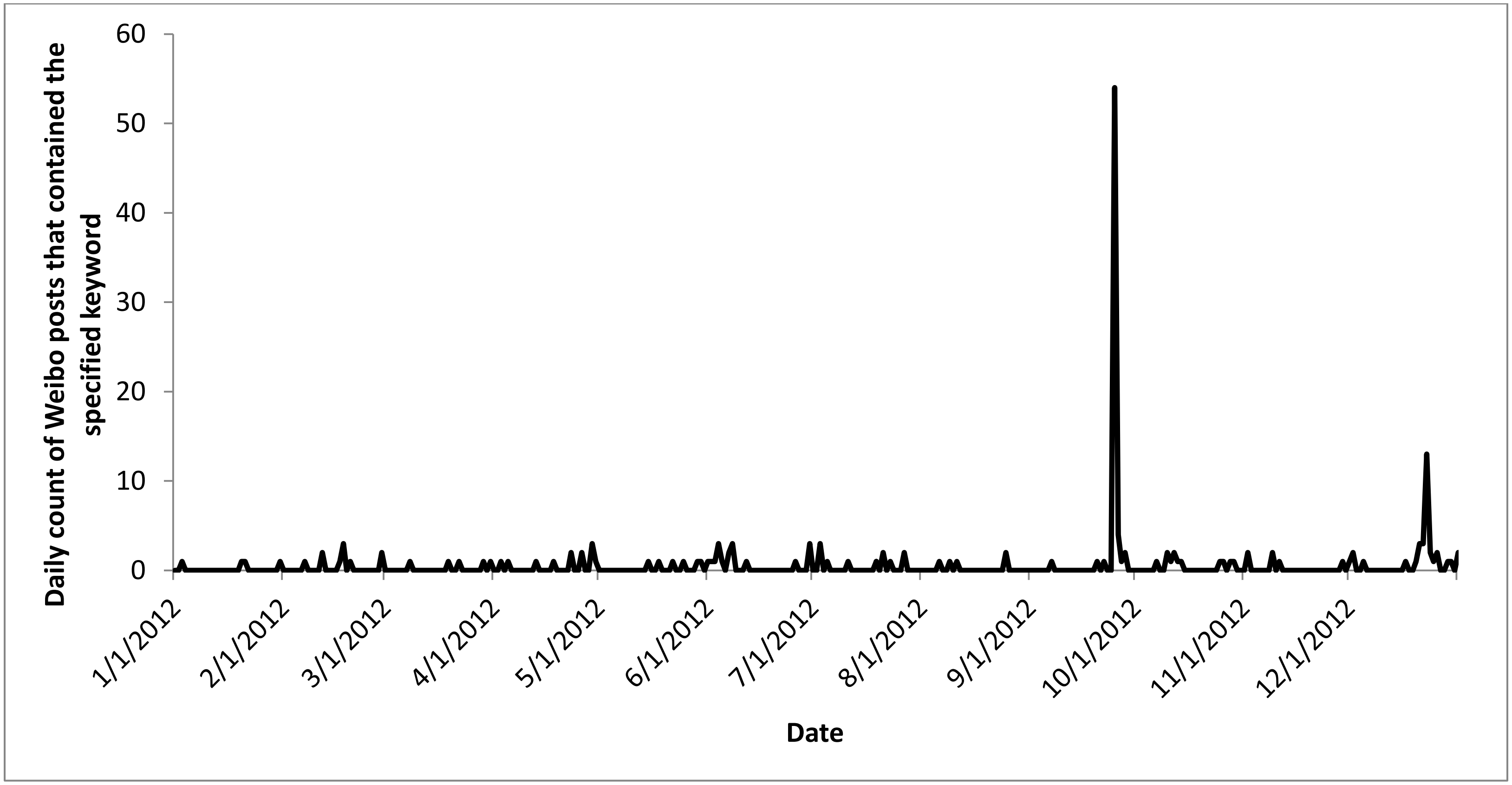

Content analysis was performed for Weibo posts generated on the peak date of September 25, 2012.

Peak Date: September 25, 2012

Total count: 54

Summary: On September 25, 2012, all of the 54 Weibo posts that mentioned “schistosomiasis” (血吸虫病) were about a case of suspected medical malpractice. A man who had his spleen removed due to schistosomiasis found his left kidney “gone missing”. The surgeon responsible for the surgery said that the patient actually had a congenital unitary kidney. It was the tension between the patient and the surgeon that drew the attention of many netizens. We classify this under the category of social issue.

Table S30. Sample Weibo entries that contained the keyword for “schistosomiasis” (血吸虫病) on September 25, 2012.

Sample Weibo posts on that day	English translation / description	Count (%)	Sources / References / Notes	Category
【男子摘除脾脏手术后左肾莫名消失 医生否认切除】一年前，岳阳人刘勇因血吸虫病切除了脾脏。不久前，他因腰痛去检查，意外发现自己左肾“不见了”。刘勇和家人怀疑，手术时自己的肾被人切掉了。刘勇的主刀医生称，医院不可能把患者的肾切掉，患者的左肾实际上是个“哑肾”。http://t.cn/zlLNYZv	“A man’s left kidney disappeared inexplicably after a spleen extirpation surgery. The doctor denied the resection of his kidney.” One year ago, Liu Yong who was from Yueyang had his spleen extirpated due to schistosomiasis. Not long ago, he went to do medical check-up because of backache and surprisingly found that his left kidney had “gone missing”. Liu Yong and his family doubted that his kidney was removed during the surgery. His surgeon said it was impossible that Liu Yong’s left kidney was extirpated in the hospital, and that his left kidney is actually a “Dumb kidney” [unilateral renal agenesis, i.e. the patient has a congenital unitary kidney]. http://t.cn/zlLNYZv	54 (100%)	http://www.chinanews.com/fz/2012/09-25/4208934.shtml	Social issue – tension between a medical doctor and a patient (News of a case of unilateral renal agenesis / suspected medical malpractice)

31. Malaria

Figure S31. Daily count of Weibo posts with the keyword for "Malaria" (疟疾) in 2012 among Sina Weibo users with 1000 followers or more.

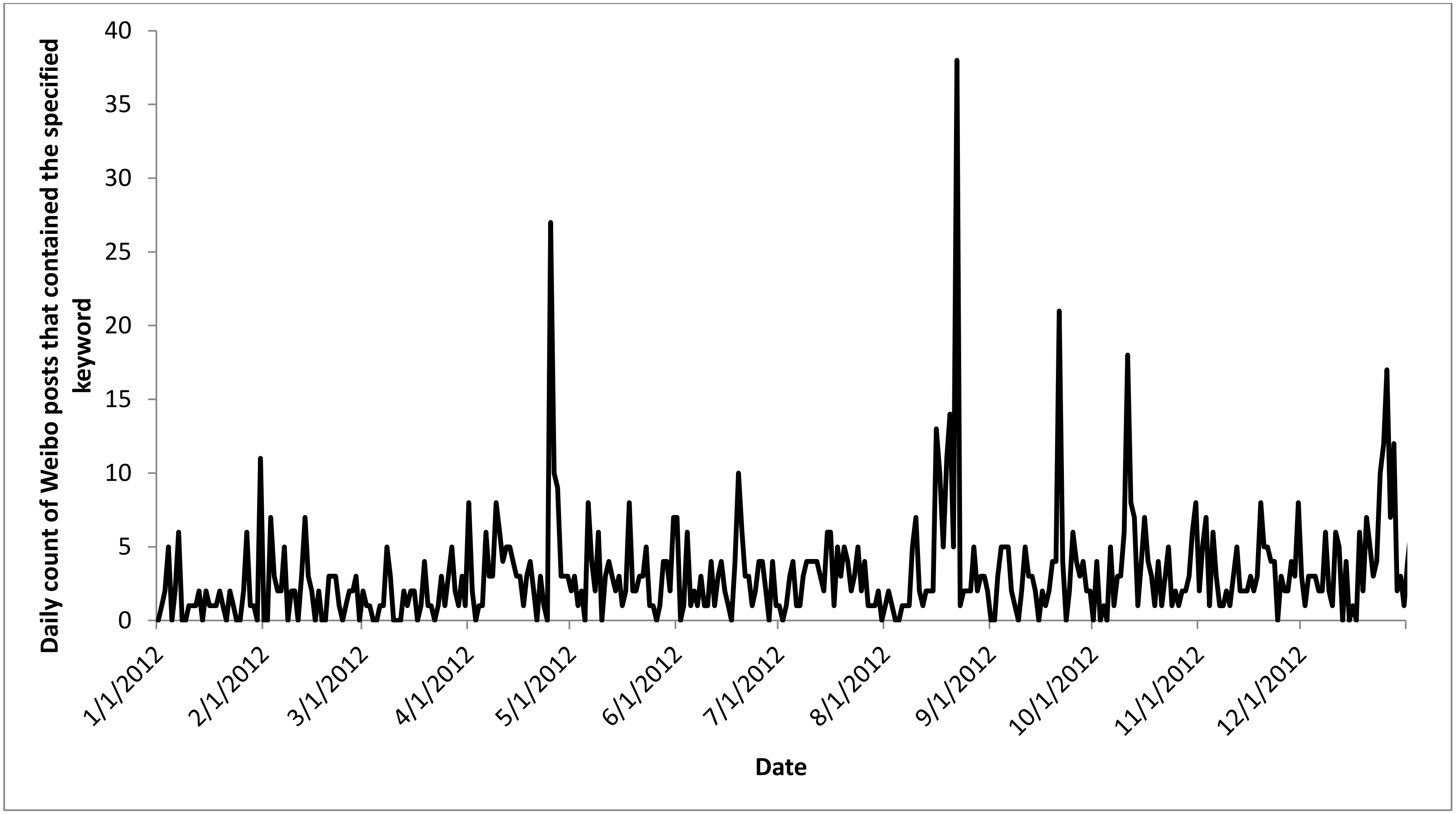

Content analysis was performed for Weibo posts generated on the peak date of August 22, 2012.

Peak Date: August 22, 2012

Total count: 38

Summary: The peak of Weibo posts mentioning "malaria" (疟疾) on August 22, 2012 was mainly related to a case of malaria in Beijing. According to a newspaper report (*The Beijing News*, August 23, 2012: http://epaper.bjnews.com.cn/html/2012-08/23/content_366619.htm?div=-1), Mr. ZHANG Liang (张亮), who travelled to Africa frequently on business trips, was infected with malaria and had severe complications. He received treatment in a hospital in Beijing. Due to his severe conditions, his 5-day treatment plan involved a transfusion of 4000cc of blood plasma each day, which required 8000cc of blood of type AB. (According to a Weibo post, the severe malaria led organ failure and a loss of liver function and blood transfusion was used to maintain life (not cure); see: http://www.weibo.com/2923373595/yz6T3oBYM?mod=weibotime; accessed June 10, 2014). Based on a rate of blood donation of 200cc per head per donation, Mr. Zhang's 5-day treatment plan requires 200 blood donors of blood type AB. In order to recruit enough blood donors, his wife wrote a Weibo post on August 15, 2012, asking for help, in the hope that enough blood donors will visit the Beijing Red Cross blood center to donate blood. The post was circulated on the internet for a week. By August 23, *the Beijing News* reported that blood donors had successfully donated 250 units of 200cc of blood for Mr. Zhang. However, at the end, the patient died on August 25, 2012, leaving behind a widow and two orphans (Weibo post released by his family or friends: http://www.weibo.com/1407255042/yyNx8fflv; accessed June 10, 2014). This was a case of using Weibo to recruit blood donors in a short period of time (one week) to try to save one individual's life.

Table S31. Sample Weibo entries that contained the keyword for "Malaria" (疟疾) on August 22, 2012.

Sample Weibo posts on that day	English translation / description	Count (%)	Sources / References / Notes	Category
**Recruit blood donors for an imported case of severe malaria in Beijing**		**33 (87%)**		**News of a case**
张亮同志在非洲出差期间突然回国是因母亲病危，在老家照顾三四天后身心俱疲，发病时由于未随身携带疟疾药物所以只能回北京治疗。说起来真是太惋惜了。为张亮祈福。	Comrade Zhang Liang suddenly returned to China from his business trip in Africa because his mother was dying from illness. Being back at home taking care of his mother for 3-4 days made him very tired. When he fell ill, because he did not carry any anti-malarial medication with him, he could only return to Beijing for treatment. It is really sad. Pray for Zhang Liang.	11 (29%)	*The Beijing News* (新京報), August 23, 2012. http://epaper.bjnews.com.cn/html/2012-08/23/content_366619.htm?div=-1<br><br>http://tweets.seraph.me/user/爱他的幸福/2907595697	Seeking help (blood donors) via Weibo; and related comments

继续扩散 //@[user1] #微救命#好，多一办法救北京张亮。 继续扩!//@[user2] 堂妹在非洲迦麦隆也得过疟疾，已垂危，家人不抱希望，后经一位在美国学医回国的非洲医生治好。可否也在北京急寻这种背景医生，或请非洲使馆帮助？	Continue to share. //@[user1] #Micro Help# Good, one more method to save Zhang Liang of Beijing. Continue to share! //@[user2] When my cousin was in Cameroon, Africa, she once had malaria and was dying (family had no hope). Later, an African doctor, who returned to Cameroon after receiving medical education in the United States, successfully cured her. Can we find a doctor in Beijing who has such a background? Or can we ask African embassies for help?	22 (58%)	Examples of a similar post (re-posted a month later): http://www.weibo.com/2280166590/yDprI3U5k	Seeking help (blood donors) via Weibo; and related comments
**Other posts**		**5 (13%)**		
这是和我们在马赛马拉的旅伴步行看动物的照片，妈妈由于拒吃抗疟疾药昏倒了。由于他们说以前住在哥伦比亚，我一直认为这是一家毒枭所以对人家很友好。我们站的地方就是昨晚看到小豹子捕猎的地方，能笑的出来是因为对面有个拿着来福枪的警卫。。。	This is the photo that we took with our travel companion in Maasai Mara [National Reserve, Kenya]. Mother was [sick and] unconscious because she refuses to take anti-malarial drugs. Because they said that they previously lived in Columbia, I kept on thinking that they were drug lords, so I treated them very well. Where we stood was where we saw the small leopard hunting. We can smile because opposite us, there stool a guard with his rifle…	5 (13%)	Original Weibo post	Personal comments / experience

32. Influenza

Figure S32. Daily count of Weibo posts that mentioned the keyword word for influenza (流行性感冒 or 流感) in 2012 among Sina Weibo users with 1000 followers or more.

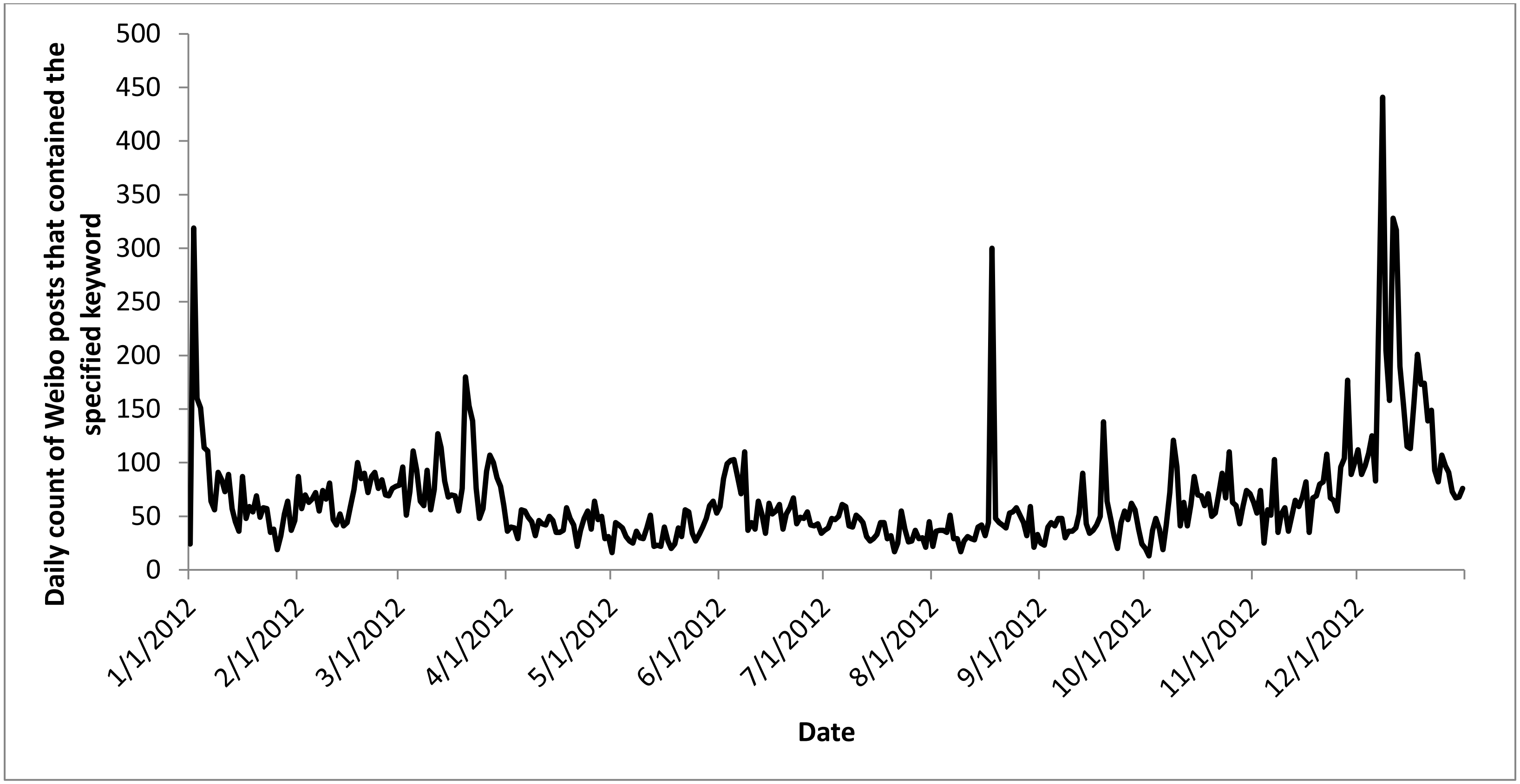

Content analysis was performed for Weibo posts generated on the peak date of December 8, 2012.

Peak Date: December 8, 2012

Total count: 441

Summary: Of the 441 Weibo posts generated on December 8, 2012, that mentioned influenza or flu, the vast majority (n=384, 87%) were about the movie "2012". The day before (December 7) was the Kamaishi earthquake that generated a tsunami that hit Miyagi Prefecture, Japan. It so happened that in the movie "2012", it also suggested that Japan would experience an earthquake on December 7, 2012. People were so intrigued by this and therefore they discussed whether the story line of the movie, that included an outbreak of influenza in Europe, would really come true in the real world. This is another example that disease-specific keyword search in Weibo or Twitter may not necessarily help us obtain posts/tweets that are really about that disease.

For the remainder of the posts, we identified many posts about influenza prevention. Some provided health information on various topics, such as statistics of zoonotic diseases (n=3), maternal and child health (n=1), germs on a mobile phone (n=1), common cold (n=1), common diseases in autumn and winter (n=1).

However, many posts contained alternative health information, according to our categorization. There were posted about dietotherapy; health effects of various types of food and tea. One post suggested that using vinegar as nose drops to prevent influenza infection. Another post suggested that consumption of curry helps the Indian people prevent influenza infection. Our observation can serve as a starting point for future research in social sciences to understand how these pieces of mis-information are circulated in social circles and online.

Three posts described personal experiences of influenza-like illness: either current experience (n=1), past experiences (n=1), or one's kid's experience (n=1). Two other post were personal comments. There were five posts that were advertisements.

Lastly, there were sixteen posts that were not related to influenza. They were picked up by our keyword search, due to the issue of tokenization of Chinese texts. Texts that contained the characters for 流感 but were unrelated to influenza were picked up by our algorithm. Future improvement of algorithm may resolve this issue.

Table S32. Sample Weibo entries that contained the keyword for "influenza" (流行性感冒 or 流感) on December 8, 2012.

Sample Weibo posts on that day	English translation / description	Count (%)	Sources / References / Notes	Category
**Entertainment (movie)**				
根据电影《2012》里的描述，7 号日本会发生地震,11 号会有国家爆发军事冲突,18 号欧洲出现极难治愈的流感病毒，20 号美国黄石国家森林公园火山爆发，21 号 the end。七号日本真的地震了。如果 11 号再应验了...我是不是就能放肆的为所欲为了 如果真的有末日，那么谁会陪我在最后一刻？	According to the description of the movie "2012", on the 7th there would be an earthquake in Japan; on the 11th there would be armed conflicts between certain countries; on the 18th an influenza virus that was extremely difficult to treat appeared in Europe; on the 20th a volcano erupted in Yellow Stone National Park in the USA; and on the 21st, the end. On the 7th, there was really an earthquake in Japan. If on the 11th, it'll come true again… Does it mean that I can be presumptuous and can do whatever I want If there is really a Last Day, who will accompany new till the	384 (87.07%)	Original Weibo post	Entertainment (Personal comment on the movie *2012*)

	very last moment?			
【12/12/07#星踪#】#kristen stewart#的电影#在路上#将于 12 月 21 日于美国小范围上映，该电影最近也开始了前期宣传活动虽然之前因为严重的流感而缺席了#OTR#的 Q&A 活动，但敬业的她还是带病参加了 LA 的私人放映会，当晚她的装束来自#Jason Wu#美丽依旧更多美图 http://t.cn/zjJSnvS	"December 7, 2012 #Star Trek#" #kristen stewart#'s movie #Road# will be released in a small group of theaters in the US on December 21. The early stage promotion activity of this movie has also begun recently. Even though she was absent from #OTR#'s Q&A activity because of severe flu, but she was very dediated and still attended a private screening in LA despite she was sick. That evening, her outfit was from #Jason Wu# Remains beautiful. More beautiful picture. http://t.cn/zjJSnvS	1 (0.23%)	http://ent.kankanews.com/c/2012-11-06/0011769575.shtml	News about the movie *Road*
我们别无选择，只能从这个流感盛行的世界出发。——宫崎骏谈《On Your Mark》-Pandacc_	"We have no choice, but to begin our journey from this world, with its epidemics of flu." – *On Your Mark*, Hayao Miyazaki.	1 (0.23%)	Original Weibo post	Entertainment (a quote of a Japanese animation)
**Health information**				
全球每年增 24 亿例人畜共患病 致 220 万人死亡】综合路透社和美国全国广播公司 7 月 5 日消息，一项全球性调查研究结果表明，人类通过动物所感染的包括肺结核、艾滋病和禽流感在内的仅 13 种疾病每年可造成 24 亿例新病患和 220 万死亡案例。	"Globally each year 2.4 billion cases of zoonotic diseases causing 2.2 million deaths" Integrating the information on July 5 from Reuters and the United States National Broadcasting Company, a global research study indicates that 13 diseases that human acquire via animals, including tuberculosis, HIV and avian influenza, have resulted in 2.4 billion new cases and 2.2 million deaths each year.	3 (0.68%)	http://gb.cri.cn/27824/2012/07/06/6071s3758903.htm	Health information (Statistics of zoonotic diseases, including avian influenza)
#FACEMEMO 母婴健康词典#预防宝宝感冒的 8 个对策：1、在感冒流行期少外出。2、全家都要常洗手。3、让宝宝多喝水。4、保持室内的通风。5、接种流感疫苗。6、全面均衡的饮食。7、充足的睡眠。8、增加宝宝的户外户内活动。	#FACEMEMO Maternal and child health dictionary# 8 strategies to prevent kids from catching common cold: 1. Go out less frequently during outbreaks of common cold. 2. The whole family should wash hands frequently. 3. Let the kids drink more water. 4. Keep the indoor environment ventilated. 5. Get vaccinated against flu. 6. To have a balanced diet. 7. To have succient sleep. 8. Increase the outdoor and indoor activities of the kids.	1 (0.23%)	Original Weibo post	Health education (encouraging parents to have the kids vaccinated against influenza)
【手机遍布大肠菌群！】美国最新研究发现，手机肮脏程度堪比厕所门把	The most recent study from the US discovers that the dirtiness of a mobile phone is comparable to that of the	1 (0.23%)	Original Weibo post	Health information

手；打电话时手机与身体有亲密接触，这样的温度恰好是细菌喜欢的；一部手机的病菌有可能引发流感、传染性结膜炎或者腹泻。可惜目前并没切实可行的清洁方式，依靠清水擦拭除菌效果并不明显，酒精虽除菌效果好，但容易损伤屏幕。	door of the restroom. When we make a phone call, our bodies are making a close contact with the mobile phone. Such temperature is what bacteria like. The germs on a mobile phone may cause influenza, conjunctivitis or diarrhea. Unfortunately, up to now, there is no cleaning method that is reliable and feasible. The germ removal effect of rubbing with clean water is not very apparent. The germ removal effect of alcohol is good, but it will easily damage the screen.			
【感冒爱找哪类人】#健康生活#“明明我比他年轻，为啥每次流感我都被感染，他却没事？”“我每天在家里待着，没机会接触感冒的人，怎么还会感冒？”很多人有过这种疑惑。其实，这可能是你的生活方式出了问题。专家提醒，感冒爱找有下列生活习惯（详见长微博）的人。	“What types of people are prone to common cold” #Healthy Living# “I am apparently younger than him. But why would every time when I catch flu, he does not catch it?” “I stay at home every day and do not have any opportunity to meet people with a cold. How would I still catch a cold?” Many people have this type of question. Actually, this may indicate that your style of living may be problematic. Experts remind that people with the following habits may be prone to common cold (see Long Weibo for details)	1 (0.23%)	Original Weibo post	Health education
【秋冬季常见病防范】1.流感：典型的流感呈现为乏力、头痛、头晕、全身酸痛;常有咽痛，鼻塞、流涕；2.鼻炎：患者会出现鼻塞，流鼻涕，鼻痒，喉部不适，咳嗽症状；3.哮喘：寒冷季节易导致呼吸道感染；4.支气管炎：当气温骤降、呼吸道小血管痉挛缺血、防御功能下降可致病。	“The prevention of diseases that are common in autumn and winter” 1. Influenza: The typical flu symptoms are fatigue, headache, dizziness, body aches; often with sore throat, nasal congestion and a running nose. 2. Rhinitis: The patient will have nasal congestion, a running and itchy nose, throat irritation, and cough. 3. Asthma: The patient is prone to respiratory infections in the cold season. 4. Bronchitis: When the temperature dips, the small blood vessels of the respiratory tract may suffer from spasm and ischaemia. The immune defense funcation may decrease and leads to illness.	1 (0.23%)	Original Weibo post	Health education
**Alternative health information**				
【4 种常见的食疗方法】常见的食疗方法通过饮食调节身体机能，达到治疗病症的效果。但是这些常见的食疗方法必须长期坚持才有效果。1、菠菜	“4 common types of dietotherapy” Common dietotherapy can regulate bodily functions to achieve disease treatment effects. However, these common dietotherapy will only have an effect if the patients persist to take them for a long period of time. 1.	4 (0.91%)	Original Weibo post	Alternative health information

防治视网膜退化；2、红茶防治流感；3、维生素 B6 防治糖尿病（糙米、面粉、蛋、白菜、干酵母等）；4、牛奶防治支气管炎。	Spinach prevents retinal degeneration; 2. Red tea prevents influenza; 3. Vitamin B6 prevents diabetes (brown rice, flour, egg, *Brassica rapa Chinensis* (bok choi), and dry yeast etc.); 4. Milk prevents bronchitis.			
【8 种食物帮宝宝抵御流感】1、酸奶——益生菌保护肠道。2、 鸡汤——美味的感冒药。3、 牛肉——补锌增强免疫力。4、蘑菇——增加白细胞抗感染。5、鱼和贝类——补硒防病毒。6、大蒜——大蒜素抗感染和细菌。7、燕麦和大麦——抗细菌抗氧化。8、红薯——增强皮肤抵抗力。	“8 types of food that help babies defend against flu” 1. Yoghurt: Probiotic bacteria protect the intestines. 2. Chicken soup: delicious medication for common cold. 3. Beef: Zinc supplement that strengthens immunity. 4. Mushrooms: increase the number white blood cells to prevent infection. 5. Fish and shellfish: Selenium supplements that protects against virus. 6. Garlic: Allicin is anti-infection and ant-bacteria. 7. Oats and barley: anti-bacteria and anti-oxidation. 8. Sweet potato: Improve the immunity of the skin.	3 (0.68%)	Original Weibo post	Alternative health information
#冬季小常识#1. 怕冷与饮食中缺少无机盐有关，应多摄取含根茎的蔬菜 2. 冬季常吃萝卜，可达到止咳化痰，清热解毒。3.香菇具有防治流感功效，常吃还能阻止血管硬化。4.常饮凉白开水有预防感冒的功效。5 多吃蔬菜，多补充水分。常吃大蒜能预防感冒。	#Little common knowledge in winter# 1. Feeling cold is related to a lack of inorganic salt in the diet. One should eat more vegetables with roots. 2. Regularly eating carrots in the winter can achieve the effect of suppressing cough and dissolving phlegm, as well as detoxication. 3. Shiitake has anti-influenza effect. Frequent consumptions of shiitake can help prevent atherosclerosis. 4. Drinking cold water regularly can help prevent common cold. 5. Eating more vegetables, drinking more water and eating garlic regularly can help prevent common cold.	2 (0.45%)	Original Weibo post	Alternative health information
流感来袭秋冬季防治感冒小妙招】 1.每晚热水泡脚 15 分钟； 2.生葱拌凉菜吃； 3.每日早晚、餐后用淡盐水漱口； 4.每天洗脸时要用冷水； 5.常按摩鼻沟； 6.白酒擦身； 7.鼻子擦葱治鼻塞； 8.电吹风对着太阳穴吹 3 至 5 分钟热风； 9.每次一汤匙蒜泥蜂蜜；10.早晚各服一次香油拌蛋；11.呼吸杯中热水的蒸气。	“The attack of influenza is coming. Little clever tricks to prevent common cold in autumn and winter” 1. To bath your feet in hot water for 15 minutes every night; 2. To eat raw spring onions with cold dishes; 3. To gargle with slightly salted water every morning, every night and after meals; 4. To use cold water to wash yoru face every day; 5. To massage your nasal groove regularly; 6. To use baijiu to brush the body; 7. To rub your nose with spring onions to cure nasal congestion; 8. To blow against your temple with hot air for 3-5 minutes, using an electric hair dryer; 9. To take one tablespoon of mashed garlic with honey; 10. To take an egg with sesame oil every morning and every night; 11.	2 (0.45%)	Original Weibo post	Alternative health information

	To breathe in the steam off the hot water in a cup.			
【秋天到了，又要到接种流感疫苗的时候了】在秋季，没必要给宝宝接种流感疫苗，那个疫苗不是预防所有病毒的，只是对某些种病毒有效，不是说宝宝接种了就不感冒了，你就注意平时多给宝宝喝温开水，饮食多样化，多带宝宝到户外运动，增强宝宝的免疫力就行，免疫力强，宝宝就不感冒 http://t.cn/zjxpgEu	"Autumn has arrived. Once again this is the time to get vaccinated against flu." In autumn, there is no need to get kids vaccinated against flu. This vaccine does not prevent all viruses. It is only effective against some virus. It does not mean that once vaccinated, you won't get common cold. It will be fine if you can give your kids more warm water to drink; have a diverse diet; bring your kids to do exercise outdoor, to strengthen the immunity of your kids. Once the immunity is strong, the kids won't get common cold. http://t.cn/zjxpgEu	1 (0.23%)	Original Weibo post	Alternative health information (Comment against receiving influenza vaccine)
【7 种菌类功效】1、蘑菇：抗肺癌、皮肤癌 2、香菇：降压抗癌、滋养皮肤、预防流感 3、金针菇：健脑抗癌 4、猴头菌：安神助消化、抗胃癌、食道癌 5、竹荪：减肥降压 6、草菇：增强免疫、解毒、滋阴壮阳、抗坏血病、健胃 7、平菇：软化血管、治疗植物神经紊乱，缓解更年期综合症。	[Effects of 7 fungi] 1. *Agaricus* has protective effects against lung cancer and skin cancer. 2. Shiitake reduces blood pressure; has protective effect against cancer; nourishes the skin and prevents influenza. 3. Enokitake nourishes the brain and has protective effects against cancer. 4. *Hericium erinaceus* improves digestive functions, has protective effects against cancer of the stomach and the esophagus. 5. *Phallus indusiatus* helps reduce weight and blood pressure. 6. *Volvariella volvacea* strengthens the immune system, detoxicates the body, balances the regulations of bodily functions [literally, improves the *yin* and *yang* of the body], has a protective effect against scurvy and improves the functions of the stomach. 7. Pleurotus ostreatus: softens the blood vessels, treats autonomic disorders, and relieves the menopausal syndrome.	1 (0.23%)	Original Weibo post	Alternative health information / Traditional Chinese Medicine
【#蕨菜#解热毒，常吃防流感】蕨菜中的蕨菜素对细菌有一定的抑制作用，具有良好的杀菌清炎、降气化痰、清热解毒的功效，经常食用可有效预防流感。小贴士：新鲜蕨菜在食用前可先用沸水浸烫一下，能清除蕨菜表面的黏性物质和土腥味，口感会更好。	[#Pteridium aquilinum# relieves the "heat" toxin. Frequent consumption helps prevent influenza] An element in *Pteridium aquilinum* has a certain inhibitory effect against bacteria. It is effective against bacteria and inflammation. It helps resolve the phlegm and has the effect of detoxication. Frequent consumption can effectively prevent influenza. Little tips: Boil it in water for a while before consuming fresh *Pteridium aquilinum*. This will remove the sticky substances and the smell of the soil from the surface of the *Pteridium*	1 (0.23%)	Original Weibo post	Alternative health information

	aquilinum. It tastes better.			
【咖喱可以抵御感冒】全球第一篇关于咖喱药用价值的研究文章发表于1970年，到现在为止，在医学文献检索系统上已有1700篇研究文章。可以说，咖喱的药用价值已经引起了全世界的关注。一位印度医生也说，流感很难在印度流行，就是因为人们天天吃咖喱，把流感消灭在了萌芽状态。	[Consuming curry helps prevent influenza] The first research article about the medical value of curry globally was published in 1970. Up to now, one can identify 1700 research articles on this topic from bibliographic databases of medical literature. Therefore, one can say that the medical value of curry has attracted the attention of the whole world. A doctor from India also said that, influenza is difficult to cause epidemics in India, because people there eat curry every day, destroying the influenza outbreak at its beginning.	1 (0.23%)	Original Weibo post	Alternative health information
我在@[user] #定时微博# 分享了一个很不错的文件："流行性感冒.ppt"，快来看看吧~ http://t.cn/zjJXfpu	I have shared a good document, “influenza.ppt” on @[user]’s #Fixed time Weibo#. Let’s take a look! http://t.cn/zjJXfpu	1 (0.23%)	http://vdisk.weibo.com/s/j9Xwk/1354935727	Alternative health information
从新生儿、婴幼儿智能开发的角度看，冬季出生的新生宝宝更需要通过游泳使四肢运动，促进脑部中枢神经系统的发育成熟，提高体质，增强机体的抗寒能力。还可以免受户外空气污染的侵害，有效提高人体的免疫能力，是宝宝抵御流感的“好帮手”http://t.cn/zjfTwpC	From the perspective of the intellectual development of neonates, babies and young children, neonates that were born in winter are more in need of swimming to exercise the limbs, to facilitate the brain and the central nervous system to develop to maturity, to strengthen the body, to raise the human immune capacity, which is a “good helper” to help kids defend against flu. http://t.cn/zjfTwpC	1 (0.23%)	Original Weibo post	Alternative health information
#欧世蒙牛360营养食谱#【宝宝防流感食谱】鸡蛋豆腐葱花汤：1、豆腐切块，烧煎发水煮沸后葱切碎放入；2、淋入鸡蛋，加精盐并勾少许生粉，点入香油即成。PS 鸡蛋含蛋白质、脂肪、卵黄素、卵磷脂、维生素和铁、钙、钾等，豆腐含有丰富的大豆卵磷脂，有益于神经、血管、大脑的发育(图片来自网络)	#Arla Mengniu 360 Nutrition Recipe# “Recipe to help kids prevent flu” Egg, tofu and spring onion soup: 1. Cut the tofu into pieces. Fry it and then add water. When the water boils, cut spring onions into small pieces and add them to the tofu. 2. Add an egg. Add salt and some corn flour. Add some sesame oil. PS: Eggs contain protein, fat, riboflavin, lecithin, vitamin and iron, calcium and potassium. Tobu contains a lot of soy lecithin and is good for the development of the nerves, blood vessels and brain. (Picture from the internet)	1 (0.23%)	Original Weibo post	Alternative health information (Recipe)
【7款排毒花草茶】①薄荷甘草茶：清凉解毒、防治感冒；②杜仲茶：补	[7 types of herbal tea for detoxication] 1. Licorice Mint Tea has the detoxication effect and prevent	1 (0.23%)	Original Weibo post	Alternative health

血壮骨、强健腰身、缓解久坐疲劳；③枸杞茶：补肾益精、养肝明目、缓解视疲劳、增强记忆力；④陈皮姜茶：止咳化痰、健胃消食；⑤人参茶：补中益气、安神生津；⑥玫瑰花茶：凉血养颜、减肥祛口臭；⑦金银花茶：预防流感、呼吸道炎症。	common cold. 2. *Eucommia ulmoides* tea strengthens the circulatory system and the bones, strengthens the waist and relieves fatigue due to a sedentary life style. 3. Lycium tea improves kidney functions, strengthens the liver the eye. It relieves the fatigue of the eyes and improves memory. 4. Sundried tangerine peel ginger tea suppresses coughs, resolves phlegm and improve digestive functions. 5. Ginseng tea relieves the deficiency of bodily functions [literally, *qi*] and have soothing funcations. 6. Rose tea improves one's beauty, reduces weights and relieves bad breath. 7. Japanese honeysuckle tea: prevent influenza and inflammation of the respiratory tracts.			information / Traditional Chinese Medicine
【预防流感应坚持的生活习惯】①经常洗手；②用手关节擦眼睛。③把牙刷放在微波炉用高火加热 10 秒；④注射流感疫苗；⑤减少自责；⑥冬天把窗户留一个缝；⑦调控居室湿度；⑧每天食用大蒜瓣；⑨每天饮用一杯酸奶；⑩每晚清洗指甲沟；⑾擦鼻涕而不要擤鼻涕。	"Habits that should be maintained to prevent flu" 1. Frequent handwashing. 2. Rub the eye with the joint of the hand. 3. To heat a toothbrush in the microwave for 10 seconds at High level. 4. To vaccinate against flu. 5. To reduce blaming oneself. 6. To leave a seam at the windows in winter. 7. To control the humidity at home. 8 Everyday consume garlic petal. 9. Everyday drink a cup of yoghurt. 10. To clean nail grooves every night. 11. To clean your nose, not to blow your nose.	1 (0.23%)	Original Weibo post	Alternative health information
【食醋滴鼻、熏蒸】将食醋以冷开水稀释，配制成 5%-10%溶液滴鼻，每日 4-6 次，每侧鼻孔滴入 2-3 滴，对治疗感冒及流行性感冒有很好的疗效。尤其是感冒初期，疗效更佳，食醋可杀灭潜伏在鼻咽部的感冒病毒。在感冒流行期间，用食醋滴鼻有可靠的预防作用。	"Vinegar as nose drop and in fumigation" Dilute vinegar with cold water as 5% - 10% solution and use it as nose drops, 4-6 times a day, with 2-3 drops per nostril. It has a very good treatment effect against common cold and influenza. Especially in the early phrase of common cold, its treatment effect is even better. Vinegar can kill the common cold virus hidden in the nasopharynx. During an influenza epidemic, using vinegar as nose drops has a reliable preventive effect.	1 (0.23%)	Original Weibo post	Alternative health information
【冬天湿发外出容易感冒。是真是假】也许是。两年前，科学家将 180 人分为两组，其中一组人用冷水泡脚弄湿，结果该组人患感冒的风险增加两倍。专家新解：也不必过于害怕，外出前洗头还是可以的。但在流感季	"In winter, going out with wet hair is prone to catching cold. Is it true or false?" Perhaps it is true. Two years ago, scientists divide 180 people into two groups, of which one group had put their feet into cold water to make them wet. At the end, the risk of catching common cold among this group of people increased two fold. New explanations from experts: there is no	1 (0.23%)	Original Weibo post	Alternative health information

节到来之前要打疫苗，未雨绸缪。	need to be over-anxious. Washing your hair before going out is still OK. But before the flu season, one should get prepared and receive vaccination.			
Personal experience				
個天懲罰我免費流感針都唔打，即病，各種唔舒服，訓唔到，好辛苦😭聽日 outing，唔好咁😭😭😭😭	The heaven is punishing me for not taking the freely available influenza vaccine. And I fall ill immediately.<br>All kinds of discomfort. Cannot sleep. Very tough. 😭<br>Tomorrow [is our] outing. Don't fall ill like that 😭😭😭😭	1 (0.23%)	Original Weibo post Note: this post was written in Cantonese and was in traditional Chinese characters. It is likely to come from a user in Hong Kong or Macau.	Personal experience of influenza
到美国后很少感冒 偶尔一次好难受啊 迁延数日不愈 耽误事儿 其实每年学校都给打 flu shot，我却从来不打 看来以后得考虑一下了 记得出国前在医院上班 每年冬天全科医护必集体流感三次 值班室吊瓶如林 唯一年入冬我坚持步行上班 到开春儿竟一针未挨 运动是王道啊	Since my arrival at the US, I seldom have common cold. Once in a while I have a cold, and it is really uncomfortable. Lasting for several days, I still have not recovered, delaying things. Actually every year, the school is giving us flu shot, but I never take it. It seems that in the future, I should consider it. I remember that before I left my country, I worked in the hospital. Every winter, all the doctors and nurses in our department always catch the flu 3 times. Our office is full of the intravenous infusion bottle, just like a forest. It was only a particular year that I insisted on walking to work. Until the beginning of spring, I have never experienced intravenous infusion. Exercise is the royal path!	1 (0.23%)	Original Weibo post	Personal experience about flu infection in the past
@[user] 話今朝醫生八點半已巡房，中左隻菌類似流感果啲，冇咩事可以出院。係車瞓左返到黎醒左，唔夠瞓，發脾氣，唔肯沖涼、唔肯換衫、唔肯瞓覺。後尾醒晒冇發脾氣，之後佢同佢婆出去滙合姨姨。佢見有新琴玩，催返屋企，玩多陣就去瞓晏覺。全日冇燒，要食消炎藥兩日，係差不多瞓時開始間中有咳	@[user] said that this morning doctors have done their grand round at 8.30am. Those with infections similar to flu who did not have serious complications could be discharged. Falling asleep in the car, and waking up upon arrival, [the kid] did not have enough sleep and had a bad temper, refusing to take a shower, refusing to change clothes and refusing to go to bed. Later, when [the kid] is fully awake, he [or she] no longer lost his [or her] temper. Later, he [or she] went out with his [or her] grandma to meet his [or her] aunt. He [or she] saw that there was a new piano at home, urged to go home. After playing for a while, he [or she] went to bed. No fever the whole day. Has to take antibiotics for two days. When he [or she] was about to fall asleep, he [or	1 (0.23%)	Original Weibo post Note: this post was written in Cantonese and in traditional Chinese characters.	Personal experience possibly about one's kid

	she] had a little cough.			
**Personal comments**				
早晨坐公交车时，忍不住打了个喷嚏， 就听后座一女人娇滴滴的说：“老公，听说最近甲流感很严重呢，好害怕哦～”然后那男人说：“怕啥，人流你都不怕还怕甲流？～～”	In the morning, when I travelled on the bus, I could not help but sneeze once. And then the woman sitting behind me, speaking in a sweet voice, “Hubby, I have heard that the flu A is really serious recently. I am really scared…” Then the guy said, “Scared what? You were not even scared of abortion, how come you are now scared of flu A?...”	1 (0.23%)	Original Weibo post	Personal comment
迎接流感	To greet flu	1 (0.23%)	Original Weibo post	Personal comment
**Advertisement**				
#丫迷每日养生#冬季养生八宜：一宜微出汗，二宜手脚暖，三宜防流感，四宜多喝水，五调精气神，六宜太阳晒，七宜心放宽，八宜多锻炼。养好“八宜态”，健康就常在！ http://t.cn/zlyEMBO 健康食材，智慧养生，就在丫迷小厨哦~	# Ya Mi Everyday Keep Healthy # 8 things to do to keep yourself healthy in winter: 1. To sweat a little bit; 2. To keep your hands and feet warm; 3. To prevent influenza; 4. To drink more water; 5. To regulate your mind; 6. To sunbathe; 7. To keep yourself happy; and 8. To do more exercises. Maintaining these eight good habits will help you stay healthy! http://t.cn/zlyEMBO Healthy food and the wisdom to keep oneself healthy are found in the Ya Mi Little Kitchen.	3 (0.68%)	Original Weibo post	Advertisement of a restaurant
NatraBio Childrens 天然顺势婴儿咳嗽滴剂/水果味宝宝止咳糖浆，现售价$6.39！适合 4 个月-12 岁以下的宝宝，NatraBio 的系列产品均为天然顺势疗法的药物和保健品.儿童感冒和流感天然药物滴剂适合 4 个月以上的婴幼儿使用,安全无副作用,能解除由感冒、流感、鼻塞、刺激引起的咳嗽 http://t.cn/zjJO2LR	NatraBio Childrens Natural homeopathic baby cough drop/Fruit favor children cough suppressant syrup. Retails price is $6.39! It is suitable for kids from 4-month-old to below 12 years of age. Products of NatraBio series are all homeopathic medicine and health products. Natural medication drops for common cold and flu among children is suitable for use in babies and young kids above the age of 4 months. It is safe with no side effects. It can relieve the cough induced by common cold, flu, nose congestion and other stimulations. http://t.cn/zjJO2LR	1 (0.23%)	http://blog.sina.com.cn/s/blog_89be57a30101bvic.html	Advertisement of homeopathic medicine
【美国亚马逊】Mabis 蒸汽吸入器，有效帮助缓解感冒、流感和鼻窦炎等症状，折后低$30.32 http://t.cn/zjJDDsq	“US Amazon” Mabis Steam Inhaler effectively relieves the symptoms of common cold, influenza and sinusitis. It costs $30.32 after discount. http://t.cn/zjJDDsq	1 (0.23%)	http://www.haitaocheng.com/Product/Detail-3998.html	Advertisement of health care apparatus

**Not related to influenza ***		16 (3.63%)		

* Our keyword search picked up 16 posts that were not related to influenza. Fifteen contained the term "潮流感" (Cháoliú gǎn), which means trendy. One contained the term "交流感情" (Jiāoliú gǎnqíng), which means communication or exchange of emotions. In both cases, 流感 (liú gǎn; flu) were part of it and so these Weibo posts that were unrelated to influenza were picked up. To better increase the precision (positive predictive value) of our algorithm in future studies, we will need to perform a tokenization procedure of the Chinese text before the keyword search.

33. Mumps

Figure S33. Daily count of Weibo posts with the keyword for "Mumps" (腮腺炎) in 2012 among Sina Weibo users with 1000 followers or more.

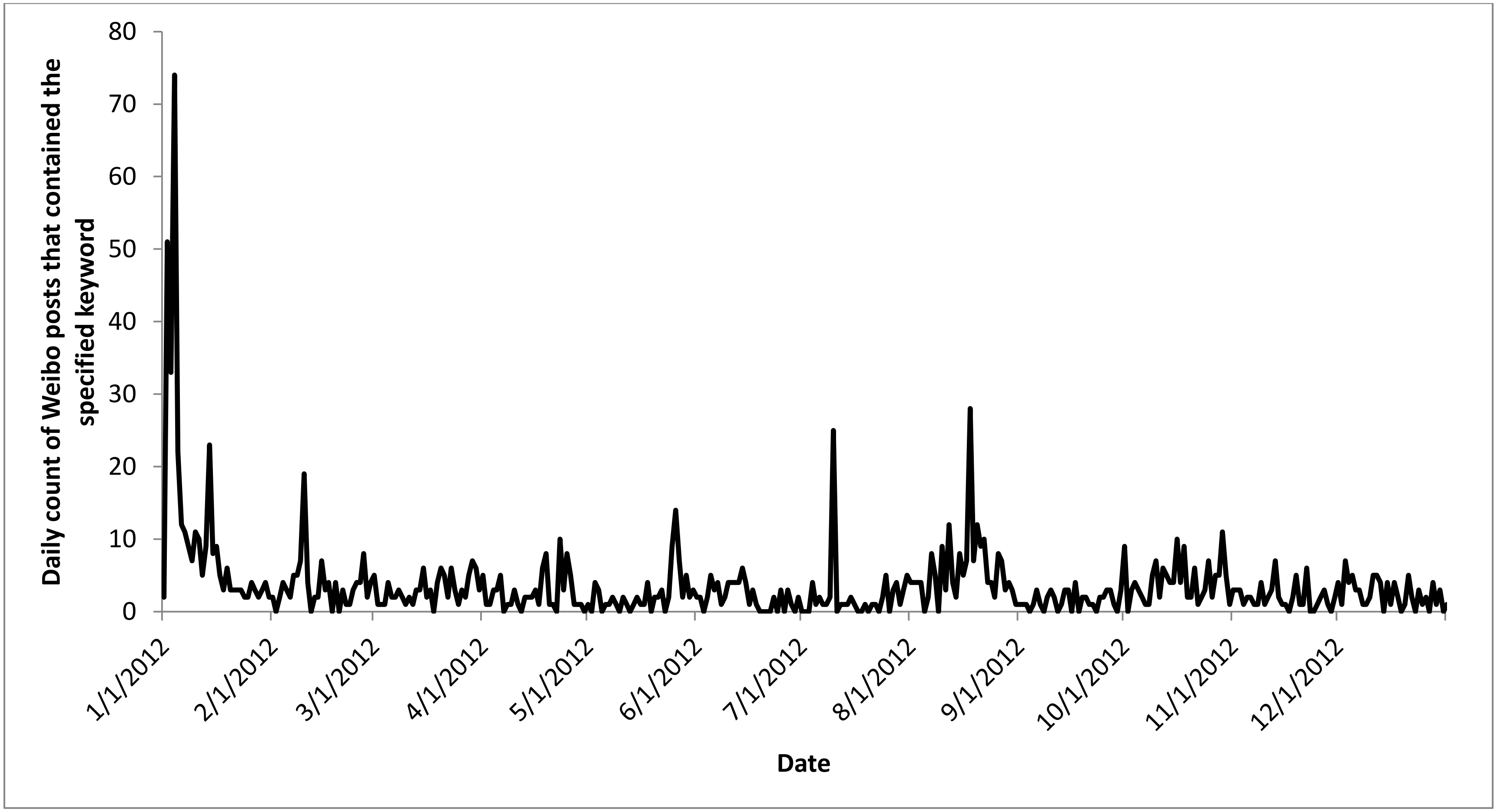

Content analysis was performed for Weibo posts generated on the peak date of January 4, 2012.

Peak Date: January 4, 2012

Total count: 74

Summary: There were 74 Weibo posts that mentioned "mumps" (腮腺炎) on January 4, 2012. Of them, 67 were posts and re-posts of health information provided by a company that mentioned four diseases that have symptoms similar to common cold. Another seven posts were about traditional Chinese medicine (n=2), health information (n=2), outbreak information released by a municipal CDC (n=1), alternative health information (n=1) and personal experience (misdiagnosis of a friend's kid) (n=1).

Table S33. Sample Weibo entries that contained the keyword for "Mumps" (腮腺炎) on January 4, 2012.

Sample Weibo posts on that day	English translation / description	Count (%)	Sources / References / Notes	Category
【四种"假感冒"要当心"】治病最怕误诊，有四种病与感冒的症状类似，千万别大意。1、水痘：身上会出水泡；2、麻疹：面颊上会出现红点，红点的中心是白色或蓝色，之后向全身蔓延；3、脑膜炎：手足剧烈疼痛、高烧、呕吐、难以唤醒；4、腮腺炎：低烧、头痛、两腮肿起。——汉妮威健康睡眠系统	"Beware of four types of "false common cold"." Misdiagnosis is the worst when it comes to treating illness. There are four illnesses that have symptoms to common cold. Do not be careless. 1. Chicken pox: blisters on the body; 2. Measles: red dots will appear on cheeks. The center of the red dots is white or blue. Later, they spread across the whole body; 3. Meningitis: severe pain at the extremities, high fever, vomiting, difficult to wake up; 4. Mumps: low fever, headache, swollen cheeks. – Honeywest Healthy Sleep System	67 (90.54%)	http://www.eyoogo.com/info-2582.html	Health information (provided by a commercial company)
板蓝根有清热解毒等功效，对感冒、流感、腮腺炎、红眼病、麻疹等常见病，有较好的预防和治疗作用。但是，少数人以为板蓝根冲剂是良性药，不管有病没病都要来两包，其实这是不对的。	Indigowoad root has the effects of detoxification. It has a better preventive and treatment effects on common diseases such as cold, flu, mumps, conjunctivitis, and measles. However, a minority of people think that indigowoad root powder is "benign drug". Whether one is ill or not one would take two packets. This is not correct.	2 (2.60%)	http://health.pclady.com.cn/healthzq/blg/<br><br>(2 different posts in the same category)	Alternative health information/ Tradition Chinese Medicine
#2012 健康月历，祝您月月吉祥，岁岁平安！#4 月份 清明时节心病多☆气候特点：清明到，气温升高，湿气仍重，气压低。☆高发疾病：抑郁症、躁狂症、精神分裂症、急性腹泻、风疹、流行性腮腺炎、阴道炎。☆健康提点：1、春天气候多	#2012 Health Calendar. We wish that every month is auspicious to you and peace to you every year! #April Qingming Festival More mental health problem. ☆Climatic characteristics: As Qingming Festival approaches, the temperature increases, humidity remains high, the air pressure is low. ☆Diseases of high incidence: depression, mania, schizophrenia, acute diarrhea, rubella, mumps, vaginitis. ☆Health reminders: 1. Weather varies a lot in	2 (2.60%)	Original Weibo post	Health information

变，气压低，阴雨绵绵，加上清明，易引发精神异常，致精神心理疾病高发。	Spring. Air pressure is low and it is rainy. Furthermore, it is Qingming. People are susceptible to mental disorders, leading to high incidence of mental illness.			
请大家转发扩散：【临沂城区小学现流行性腮腺炎】近期，流行性腮腺炎发病较往年同期明显上升，城区部分小学、托幼机构出现病例。市疾控中心和兰山区疾控中心共同发布疾病预报，提醒市民注意预防。定期开窗通风，保持空气流通和环境清洁；疾病流行期间，尽量少带孩子去公共场所。	Please re-post: "Mumps appears in primary schools in Linyi City." Recently, mumps incidence has increased sharply compared to the same period of the last year. Incident cases appear in certain primary schools and daycare centers in the city. Municipal CDC and Lanshan District CDC jointly release disease warning, to remind citizens to pay attention to disease prevention. Regularly open the window to facilitate the air flow, to maintain air circulation and to keep the environment clean. During the epidemic, try to keep children away from public space.	1 (1.35%)	Original Weibo post	Health information (public health agencies' message)
亲朋好友聚会，胖嘟嘟的宝宝真是可爱，尤其是脸上那两块"肥肥"的肉，很难让人忍住不去扭几记，猛亲几下。但医学专家却告诫大家：这样很可能会导致他们的腮腺和腮腺管一次又一次地受到撕、压、挤而导致受伤。导致流涎、口腔粘膜炎和腮腺炎等。喜爱宝宝方法一定要得当哦！	In family and friends' gathering, cute and fat babies are so lovely, especailly the two "fat" cheeks. People found it hard to resist not to twist the cheek a few times and to kiss it several times. But medical experts caution everyone: this may harm their parotid gland and parotid duct through repeated tearing, pressing and squeezing, leading to drooling, oral mucositis and mumps etc. So, to love babies we need take care of them with the right methods!	1 (1.35%)	Original Weibo post	Alternative health information
人都走了，哪找的回来啊，住的时候只有她找得着我，我从来找不着她，号称工作忙没法接电话！还一件事：我一个朋友的孩子得了普通的感冒发烧，说来也巧正好去医院看的这个大夫，结果被她给误诊成了腮腺炎，本来要带孩子去美国，结果怕恶化或者传染就没去，换了家医院开了点药没几天就好了。	That person has gone. How can one find her? When I was an in-patient, only she could find me. I could never locate her. She claimed that she was too busy to pick up the phone! Also, there was one thing: My friend's kid had a fever due to common cold. It so happened that the kid was there in the hospital seen by that doctor. So, the kid was misdiagnosed by her to have mumps. Originally my friend wanted to take the kid to the USA. However, he feared that it may get worse or may infect others, and so the kid did not go. Later, they visited another hospital. The kid was prescribed some medication. After a few days, the kid recovered.	1 (1.35%)	Original Weibo post	Personal experience (a misdiagnosis made by a doctor)

34. Rubella

Figure S34. Daily count of Weibo posts with the keyword for "Rubella" (风疹) in 2012 among Sina Weibo users with 1000 followers or more.

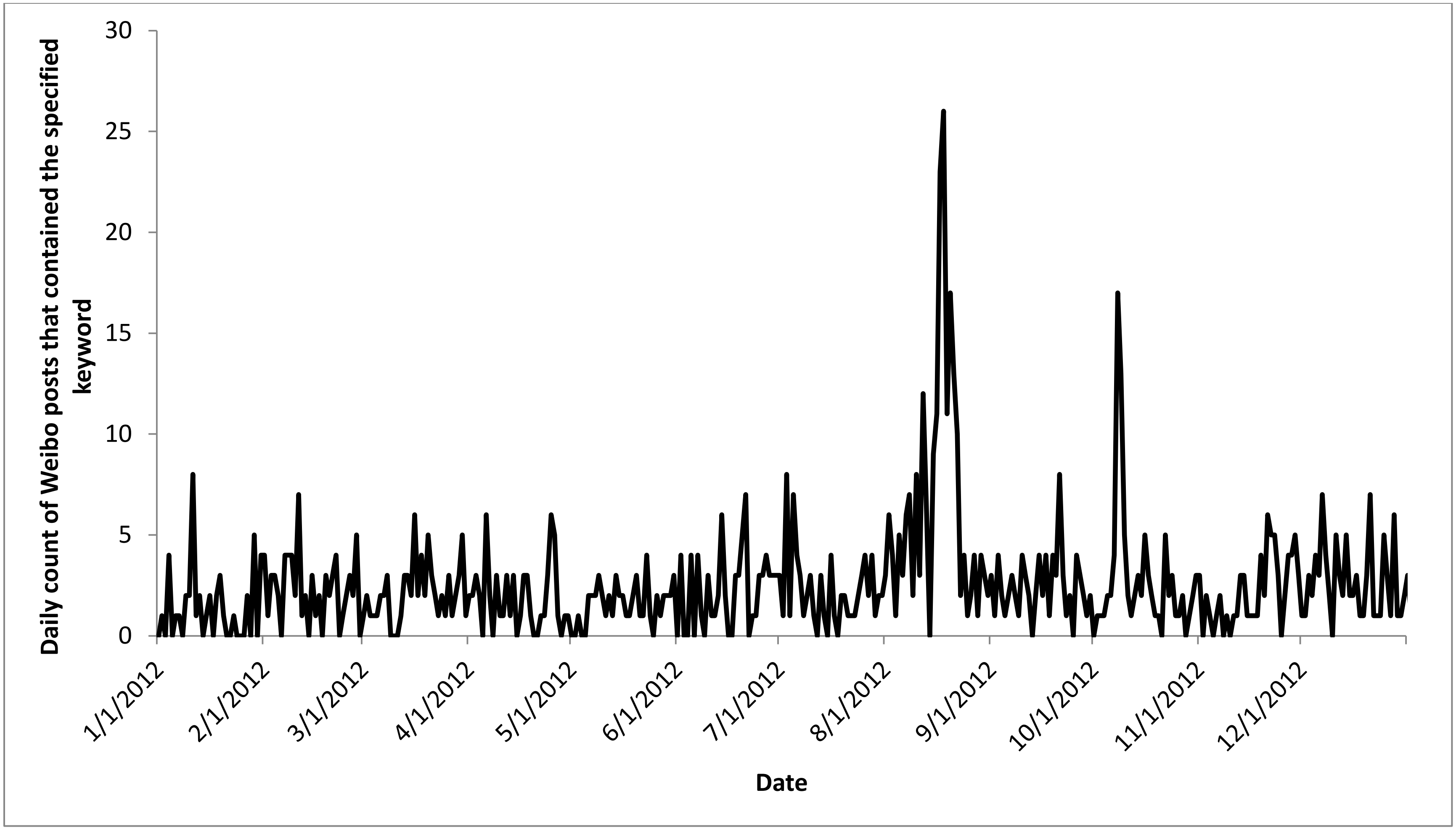

Content analysis was performed for Weibo posts generated on the peak date of August 18, 2012.

Peak Date: August 18, 2012
Total count: 26

Summary: Of the 26 Weibo posts mentioning "rubella" on August 18, 2012, 10 were related to health information about prevention of childhood disease and 2 on vaccination. There were 12 posts related to alternative health information (9 on digit ratio; 3 on alternative dietary information about crabs). There were 2 posts related to personal experience with hospitals.

Table S34. Sample Weibo entries that contained the keyword for "Rubella" (风疹) on August 18, 2012.

Sample Weibo posts on that day	English translation / description	Count (%)	Sources / References / Notes	Category
春季小儿疾病早预防——幼儿保健： 春季使小儿一年中最容易得病的季节，家长应针对孩子的体质状况，有放矢地做 好预防工作。春季疾病大致可分以下三类： 一：呼吸道传染病：常见的如麻疹、风疹、水痘、上感、腮腺炎、流行性脑膜炎	In spring, prevent childhood diseases early – infant health protection: Spring is the season when infants are most susceptible to diseases. Parents should perform targeted preventive work, tailored to the body conditions of the children. Spring diseases can be roughly categorized into 3 categories: (1) Respiratory infections: Common diseases include: measles, rubella, chickenpox, upper respiratory tract infection, mumps, epidemic meningitis	10 (38%)	This post was the first paragraph of this article: http://www.jy135.com/html/yuerbaike/changjianwenti/2009/0215/18859.html	Health information
【看手指辨健康】无名指长于食指者易患：①关节炎：患关节炎危险会增加一倍。②厌食症。③感冒：除了感冒几率更大，也更容易发生水痘和风疹。食指长于无名指者易患：①心脏病：食指更长的男性在 50 岁前发生心脏病的危险更大，无名指越长心脏病危险越小。②易饿症。③哮喘和过敏。	"Look at fingers Differentiate Health" People with ring fingers longer than index fingers are susceptible to: (1) Arthritis: the risk of arthritis increase by one fold; (2) anorexia; (3) common cold: apart from an increased probability of catching a cold, they are more susceptible to chickenpox and rubella. People with index fingers longer than ring fingers are susceptible to: (1) heart disease: men with longer index fingers have a higher risk of an onset of heart disease before the age of 50 years. The longer is the ring finger, the risk of heart disease is smaller; (2) Bulimia; (3) Asthma and Allergy.	9 (35%)	This article cites the *Daily Mail* citing John Manning: http://www.91kangkang.com/c/2BbVWuT6aHOj.html	Alternative health information (News article about digit ratio)
【7 类人不适合吃螃蟹】1、伤风、发热、胃痛以及腹泻的病人，吃螃蟹加重病情 2、慢性胃炎、十二指肠溃疡、胆囊炎、胆结石症、肝炎活动期的病人 3、冠心病、高血压、动脉硬化、高血脂的人少吃蟹黄 4、过敏的人，起风疹块 5、脾胃虚寒的人，易腹痛	"7 types of people should not eat crab" 1. Patients with common cold, stomach pain and diarrhea. Eating crab exacerbates the disease. 2. Patients with chronic gastritis, duodenal ulcer, cholecystitis, gallstone, and active hepatitis. 3. People with Coronary heart disease, high blood pressure, arteriosclerosis, high cholesterol should eat crab roe less. 4. People allergic to crab will develop urticaria (*Fengzhen kuai*)*. 5. People with *Piwei Xuhan* (Spleen and stomach deficiency) are	3 (12%)	Apparently this Weibo post seems to be derived from a certain article with different titles on different websites, e.g. 秋季 7 种人群不宜吃螃蟹 http://www.360doc.com/content/10/0826/07/1720781_48832419.shtml 蟹肉蛋白质比猪肉高 七类人不	Alternative health information

腹泻 6、孕妇 7、老年人。	susceptible to stomach ache and diarrhea. 6. Pregnant women. 7. Elderly.		适宜吃螃蟹 http://www.ahlx.gov.cn/web/Content.aspx?chn=118&id=31880	
【接种疫苗选孩子身体好时】接种前先测体温，若有发烧要推迟接种、未完全恢复健康前暂缓注射，但应在病好后及时补接种。接种当天不要洗澡，也不能让孩子太疲劳。如孩子正在发烧，患有急性传染病、哮喘、风疹、湿疹等疾病或有心脏病、肾炎及肝炎等疾病时，暂时不要接种。	"To vaccinate, choose the time when children's health is good" Before vaccination, first measure body temperature. If there is a fever, postpone vaccination until full recovery. Should vaccinate as soon as he is recovered. Do not take a shower on the day of vaccination and cannot let the child become too tired. If the child has a fever, being infected with diseases such as acute infections, asthma, rubella and eczema, or have diseases such as heart disease, nephritis or hepatitis, then temporarily he should not be vaccinated.	2 (8%)	This Weibo post was derived from the first section of this article: 接种疫苗要孩子身体好的时候进行 http://www.360doc.com/content/11/0411/10/6146507_108772067.shtml	Health information
李时珍华佗快显灵吧 //@[user1]小时候我从床上摔下来，额头超大一个包，医生说要开颅，后来过了半个月自己就消了~高中我得风疹，医院验血说我是食物过敏，吊针屁股针各种药一起上，没好，也是在家躺着后来好了。还有后来补牙。各种不满，然后就再没去医院~//@[user2]操！	Li Shizhen, Hua Tuo, please appear! //@[user1]When I was small, I fell from my bed. My forehead was swollen. The doctors said that I need head surgery. Later, after half a month, it was resolved. ~ When I was in high school, I was infected with rubella. Blood tests at the hospital concluded that it was food allergy. With intravenous therapy, intradermal injection and other medication all going together, I did not recover. At the end, I rested at home and recovered eventually. Also, dental filling. Various kinds of discontent, And later I don't go to hospitals anymore. ~//@ [user2]Fxxk!	2 (8%)	Original Weibo post	Personal comments / personal experience

* Urticaria is also known as 风疹块 *Fengzhen kuai* in Chinese.

35. Conjunctivitis

Figure S35. Daily count of Weibo posts with the keyword for "Conjunctivitis" (结膜炎) in 2012 among Sina Weibo users with 1000 followers or more.

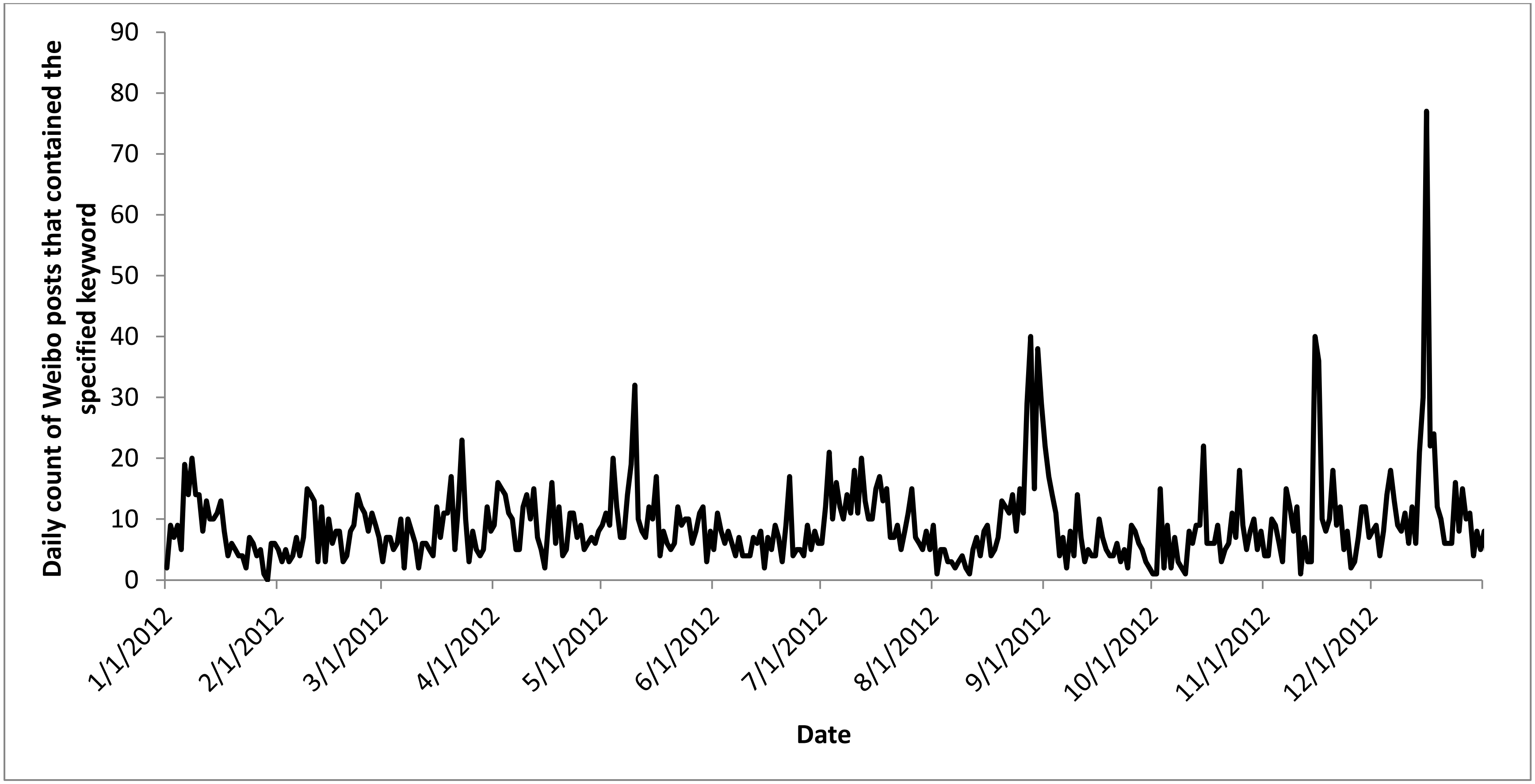

Content analysis was performed for Weibo posts generated on the peak date of December 16, 2012.

Peak Date: December 16, 2012

Total count: 77

Summary: Of the 77 Weibo posts that mentioned "conjunctivitis" (结膜炎) on December 16, 2012, 70 posts were about preservatives in eye drops. The posts mentioned that if used inappropriately, eye drops with preservatives may lead to chronic conjunctivitis and blepharitis and may exacerbate keratoconjunctivitis sicca.

Another 3 posts listed 8 situations when contact lens should be worn. One of which was "In Spring, wearing contact lens may easily lead to allergic conjunctivitis".

There were 4 other posts that mentioned 3 acupuncture points on the thumb can help relieve eye symptoms, including acute conjunctivitis.

Table S35. Sample Weibo entries that contained the keyword for "Conjunctivitis" (结膜炎) on December 16, 2012.

Sample Weibo posts on that day	English translation / description	Count (%)	Sources / References / Notes	Category
#微提醒#【市面上 90%眼药水含防腐剂 医生建议慎用】觉得眼睛疲劳、不舒服，你是不是会使用眼药水来缓解？眼科专家表示，目前市面上 90%以上的眼药水均含有防腐剂，而防腐剂这类添加剂对眼表有毒性，不利于眼睛的健康。若使用不当，有可能会引发慢性结膜炎、睑缘炎，加剧"干眼症"。http://t.cn/zjo3dbq	#Micro Reminder# "90% of eye drops available in the market contain preservatives. Doctors advice use with caution." When you feel that your eyes are tired and uncomfortable, would you relieve your discomfort with eye drops? Ophthalmologists say that, 90% of eye drops available in the market contain preservatives. And additives such as preservatives are toxic to the ocular surface and is harmful to the health of the eyes. If used inappropriately, eye drops may lead to chronic conjunctivitis and blepharitis, and may exacerbate "dry eye syndrome" [keratoconjunctivitis sicca]. http://t.cn/zjo3dbq	70 (91%)	http://guangbaobei.net/u/d-i-34845.htm<br><br>http://hunan.sina.com.cn/news/s/2012-12-16/085930272.html	Health information
【8 种情况勿戴隐形眼睛】①长距离骑车镜片会变硬，损伤角膜;②月经期间眼压高、不宜戴隐形;③感冒时常伴有视网膜炎症，戴隐形加重炎症;④乘坐飞机戴隐形眼镜角膜易水肿溃疡;⑤孕期戴隐形可致急性角膜损伤;⑥游泳时戴隐形眼镜镜片会吸附病菌;⑦用电脑戴隐形易眼睑痉	"Eight conditions in which contact lens should not be used." (1) Long distance cycling: lens will harden and damage cornea; (2) Contact lens are not suitable during menstrual period, as intraocular pressure is high; (3) When one has a cold, it often comes with retinal inflammation. Using contact lens will exacerbate the inflammation; (4) Wearing contact lens during flights will easily lead to retinal edema and ulceration; (5) Wearing contact lens during pregnancy may lead to acute cornea damage; (6) Wearing contact lens when one is swimming, bacteria will attach to the lens; (7) Wearing contact lens when one is using computers may	3 (4%)	http://www.b086.com/info_902.htm	Alternative health information

挛;⑧春季戴隐形易得过敏性结膜炎。	easily lead to blepharospasm; (8) In Spring, wearing contact lens may easily lead to allergic conjunctivitis.			
【视力恢复奇穴】在我们的拇指上有三个相邻接的穴道，分别是明眼、凤眼、大空骨（如下图）。明眼、凤眼能够改善眼睛疲劳和急性结膜炎，大空骨则可改善一切有关眼睛的症状。平时眼睛容易疲劳的人，每天要刺激这三个穴道两次。此方法还可以抑制老人性的白内障。童鞋们，帮父母学一招吧！	"Wonderful acupuncture points that can recover sight" On our thumb, there are three acupuncture points that are adjacent to each other, they are Mingyan, Fengyan and Dakonggu (see figure below). Mingyan and Fengyan can improve eye tiredness and acute conjunctivitis, while Dakonggu can improve all eye-related symptoms. People whose eyes are easily tired, can stimulate these three acupuncture points two times daily. This method can also help inhibit the development of senile cataract. Children, help [your] parents to learn this method!	4 (5%)	http://wenku.baidu.com/view/06bbdd0a52ea551810a687af.html	Alternative health information / Traditional Chinese Medicine (Acupuncture points)

36. Leprosy

Figure S36. Daily count of Weibo posts with the keyword for "Leprosy" (麻风) in 2012 among Sina Weibo users with 1000 followers or more.

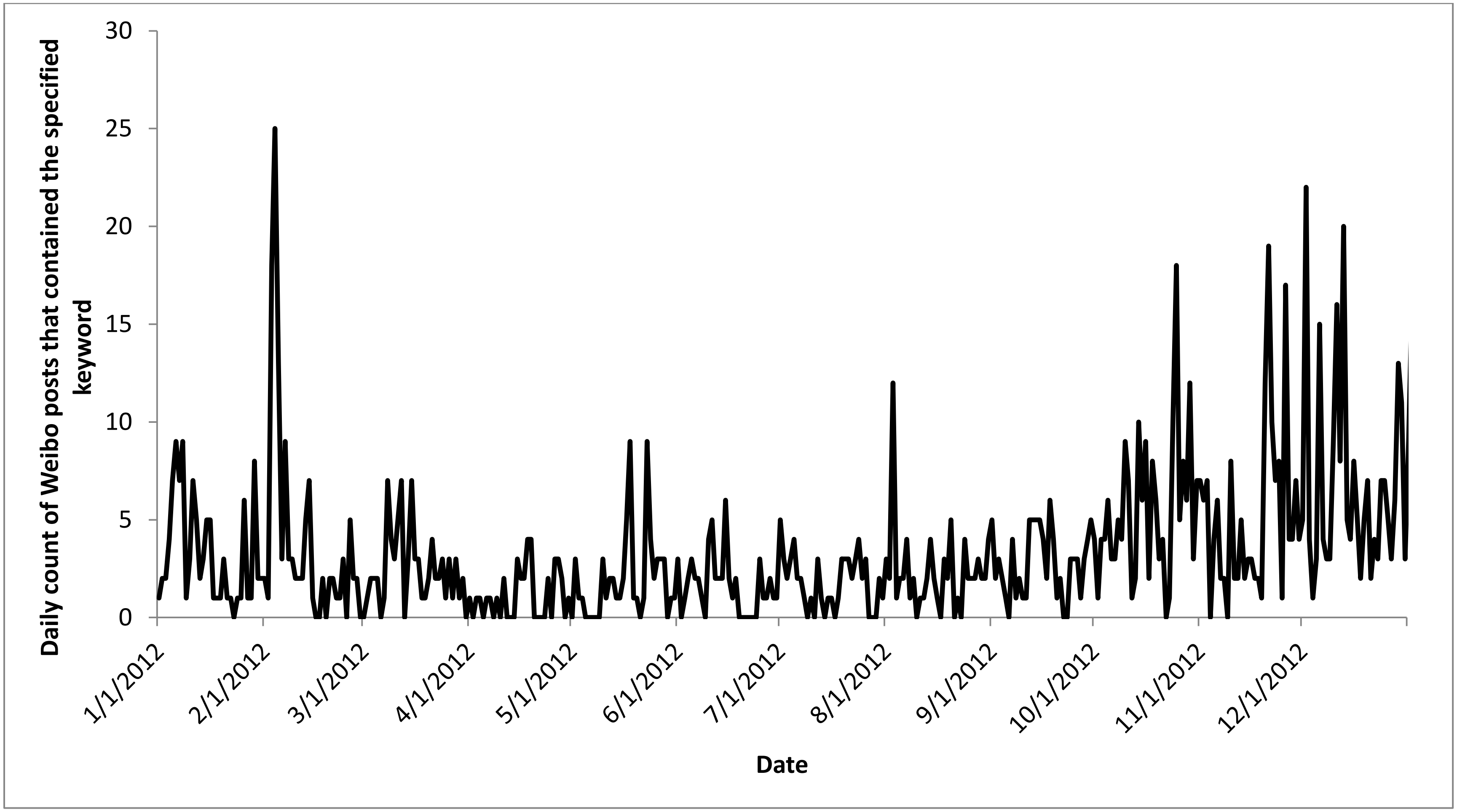

Content analysis was performed for Weibo posts generated on February 4, 2012.

Peak Date: February 4, 2012

Total count: 25

Summary: In the evening of February 3, 2012, Inspire China (Gandong Zhongguo) 2011 annual award ceremony was broadcasted on China Central Television. One of the awardees was Zhang Pingyi. Zhang Pingyi was a Taiwanese reporter-philanthropist who raised money to build a school for kids whose parents were leprosy patients in a rural village in Yuexi County, Liangshan Prefecture, Sichuan Province. (News reference: http://news.xinhuanet.com/tw/2012-02/03/c_122654869.htm). This accounted for 24 out of 25 Weibo posts created on February 4, 2012. Twelve of the 24 posts were re-posts of news articles. Another twelve were comments on this event, of nine were complementary. Of these, eight mentioned “Helping kids with leprosy, eleven years as a day. If we can persist for 10 years, China for sure will become a more open, transparent and egalitarian.” (See Table S36) However, one comment was sarcastic: “On the section on the Taiwanese journalist visiting the leprosy village at Liangshan, supporting the construction of a school there for several years, my comment is that the authors want to express a close relationship between the Mainland and Taiwan. What an irony! Is our country short of money? Is our country short of civil servants?” (台湾女记者到凉山麻风村几年如一日支教建校那段，我看作者是想表达浓厚的两岸关系，真是莫大的讽刺！我国差钱吗？差公务员吗？) This stand-alone comment at least shows that there was an individual who could read and be openly sarcastic about the political overture behind the script of this event.

Table S36. Sample Weibo entries that contained the keyword “Leprosy” (麻风) on February 4, 2012.

**Sample Weibo posts on that day**	**English translation / description**	**Count (%)**	**Sources / References / Notes**	**Category**
**Inspire China 2011 award ceremony**		**24 (96%)**		
●感动中国 2011 年度人物揭晓 >>“感动中国”2011 年度颁奖盛典 3 日晚在中央电视台综合频道播出。“最美妈妈”吴菊萍，改变山区麻风病村的台湾爱心人士张平宜等获奖。	Inspire China 2011 annual awardees revealed >> “Inspire China”: 2011 award ceremony was broadcasted in China Central Television Comprehensive Channel on the evening of the 3rd. “The most beautiful mum” Wu Juping and the Taiwanese philanthropist Zhang PingYi who has changed the leprosy village in the mountains etc. were awarded.	12(48%)	http://news.cntv.cn/program/xwlb/20120203/120740.shtml http://news.cntv.cn/china/20120203/123881.shtml http://news.cntv.cn/china/20120203/122929.shtml News about an award ceremony, in which a philanthropist who worked in a village of leprosy patients was awarded	Entertain-ment (TV show)
十一年如一日，帮助麻风孩童。我们如能坚持十年，中国必是一个更公开透明平等的中国。	Helping kids with leprosy, eleven years as a day. If we can persist for 10 years, China for sure will become a more open, transparent and egalitarian.	12(48%)	Original Weibo post	Personal comment on the awardee Zhang Pingyi
**Other Weibo posts**		**1 (4%)**		
在凉山彝族自治州走访麻风病村，这里的老人因为患病手脚无知觉，冬天靠烤火取暖，极易烫伤，经常需要护	I visited the leprosy village in the Yi Ethnic autonomous preferecture at Liangshan. The elderly there, because of lack of senses at the extremities, they are easily scalded when they	1(4%)	Original post from someone’s Renren public page (equivalent to Facebook) (not related to the award ceremony)	Personal experience

理人员帮忙处理伤口。我在为老人处理伤口时得知，老人这辈子有个遗憾，就是身份证上把他的名字写错了。或许他们这辈子都走不出这座大山了，而他们要的，只不过是一份对于其公民身份的认可和尊重。	used open fire for heating. They often require nurses to help them treat their scalds. When I help treating the scald of that old man, I learn from him that he has a regret all his life, which is the mis-printing of his name on his identity card. Perhaps they will never leave this mountain. But they want is simply a recognition and respect for their citizenship status.		http://page.renren.com/600904829/channel-photoshow-5549797411#5549797411	

37. Typhus

Figure S37. Daily count of Weibo posts that contained the keyword for “typhus” (斑疹伤寒) in 2012 among Sina Weibo users with 1000 followers or more.

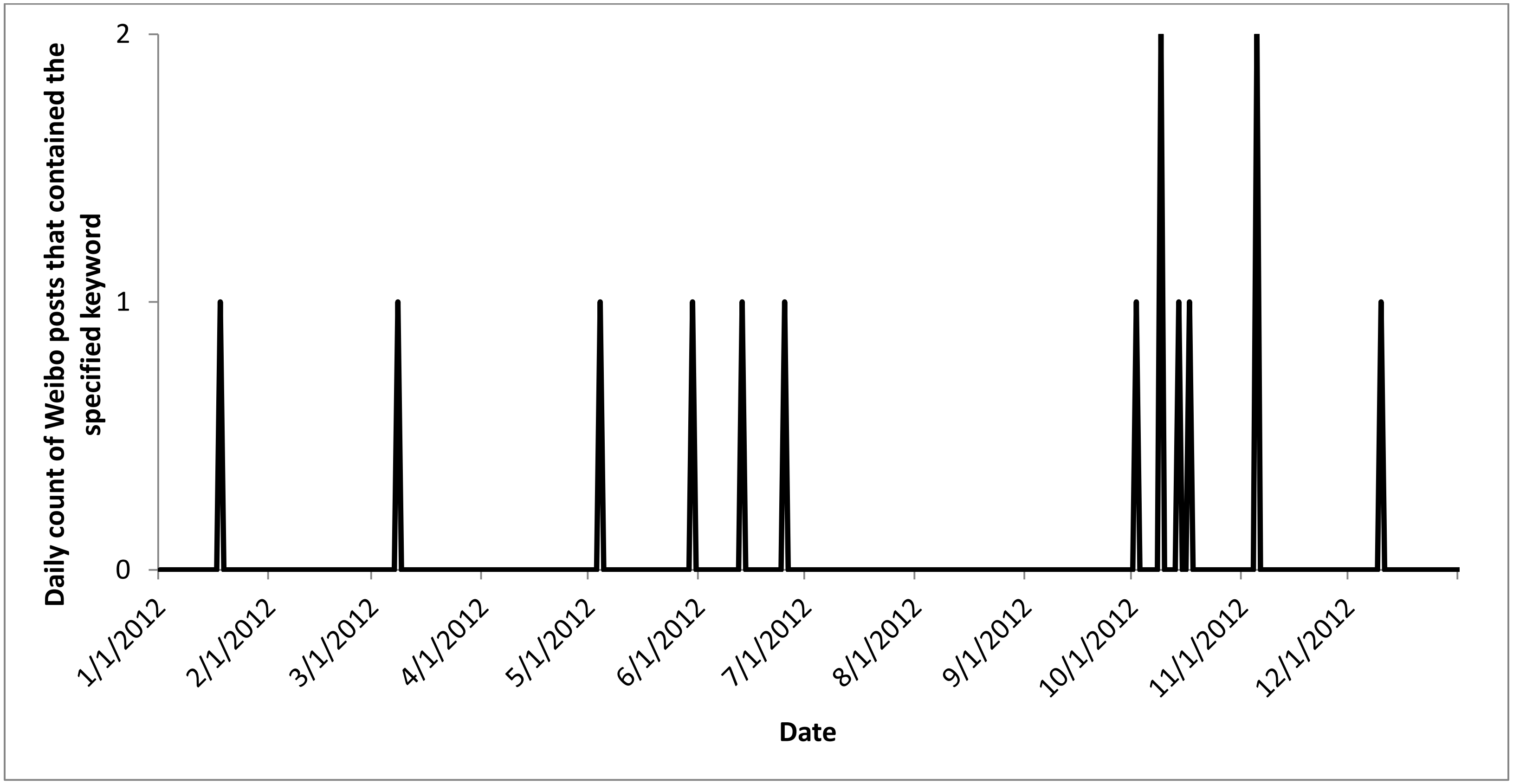

Content analysis was performed for all Weibo posts in 2012 in our database that mentioned the keyword for “typhus”. There were a total of 14 posts in the whole of 2012.

Whole year of 2012.

Total count: 14

Summary: Of the 14 posts that mentioned the keyword for “typhus” (斑疹伤寒) in the whole year of 2012, four were a quote from the movie *Schindler's List* and two were about the history of the Nobel Prize in Physiology or Medicine. Another five different Weibo posts were health information about various diseases (including typhus). One post was about the death of a footballer. According to that post, he died of typhus. There are two other posts that mentioned typhus but did not fall into our five categories.

Table S37. Sample Weibo entries that contained the keyword for “typhus” (斑疹伤寒) in the whole year of 2012.

Sample Weibo posts on that day	English translation / description	Count (%)	Sources / References / Notes	Category
他们在你身上投射了一道咒符，你知道吗，犹太人。当你和他们很亲近地工作时，就像我，你这么想。他们有这样的能量。就像是病毒。有些我的人就被这种病毒感染了。他们应该被同情，而不是惩罚。他们应该接受治疗因为这样才能像斑疹伤寒症一样真实。——《辛德勒的名单》	They cast a spell on you, you know, the Jews. When you work closely with them, like I do, you see this. They have this power. It's like a virus. Some of my men are infected with this virus. They should be pitied, not punished. They should receive treatment because this is as real as typhus. -- *Schindler's List*	4 (28.57%)	This is a quote from *Schindler's List* by Amon Goeth.	Entertain-ment (movie)
【医学诺贝尔之路】自 1901 年以来，诺贝尔生理学或医学奖已颁发了 103 次。消灭斑疹伤寒的法国医生尼克尔（1928 年），研究维生素的荷兰医生医生艾克曼与英国科学家霍普金斯（1929 年），因研究线粒体呼吸链及肿瘤细胞而获得三次诺奖提名的德国士兵瓦尔伯（1931 年） 诺奖往事连载中 →http://t.cn/zl0BhZD	“The road of the medical Nobel prize” Since 1901, the Nobel Prize in Physiology or Medicine has been awarded 103 times. The French doctor who eliminate typhus (1028), the Dutch doctor Eijkman and the British scientist Hopkins who studied vitamins (1929), the German soldier Warburg who had been nominated three times for his research on mitochondrial respiratory chain and tumor cells (1931). Nobel history stories series: http://t.cn/zl0BhZD	2 (14.29%)	http://songshuhui.net/?s=%E5%8C%BB%E5%AD%A6%E8%AF%BA%E8%B4%9D%E5%B0%94%E4%B9%8B%E8%B7%AF	Health education (History of the Nobel Prize in Physiology or Medicine)
昆虫叮咬可能带来的危害：● 蚊子可能携带疟疾、登革热等疾病。 ● 苍蝇可以通过接触传播病毒，它们可能是嗜睡症、伤寒症、霍乱的病源，有时还会携带痢疾病毒。 ● 跳蚤可以传播瘟疫。 ● 虱子会传染斑疹伤寒症、回归热。 ● 扁虱也可能携带并传播疾病，例如落基山斑疹热，这在美国许多地区很常见。	Harms that insect bites may bring: ● Insects may carry malaria, dengue and other diseases. ● Flies can transmit diseases via contacts. They can be the sources of sleeping sickness, typhoid or cholera. Sometimes they may carry virus that causes dysentery. ● Fleas may transmit plague. ● Lice can spread typhus and relapsing fever. ● Ticks can also carry and transmit diseases, such as Rocky Mountain spotted fever. This disease is very	5 (35.71%)	Note: 5 entirely different health information Weibo posts. The one on the left is just a representative.	Health information

	common in very parts of the United States.			
【俱乐部拖欠四月月薪 巴拉圭球员无钱看病客死印尼】效力于印尼联赛的巴拉圭球员迭戈·门迭塔近日因感染斑疹伤寒去世，他所在的俱乐部拖欠了他 4 个月薪水，以至于他无钱看病，其死亡时年纪为 32 岁。	“Club owed four month salary Paraguayan player had no money to seek treatment Died in Indonesia” Paraguayan footballer Diego Mendieta, who played in the Indonesian League, recently died of typhus. His club owed him his salary for 4 months, and so he did not have money to receive treatment. When he died, he was only 32 years old.	1 (7.14%)	Original Weibo post	News of a case of typhus (Sports news)
Others		2 (14.29%)		

38. Leishmaniasis

Figure S38. Daily count of Weibo posts that mentioned the keyword for "leishmaniasis"(黑热病) in 2012 among Sina Weibo users with 1000 followers or more.

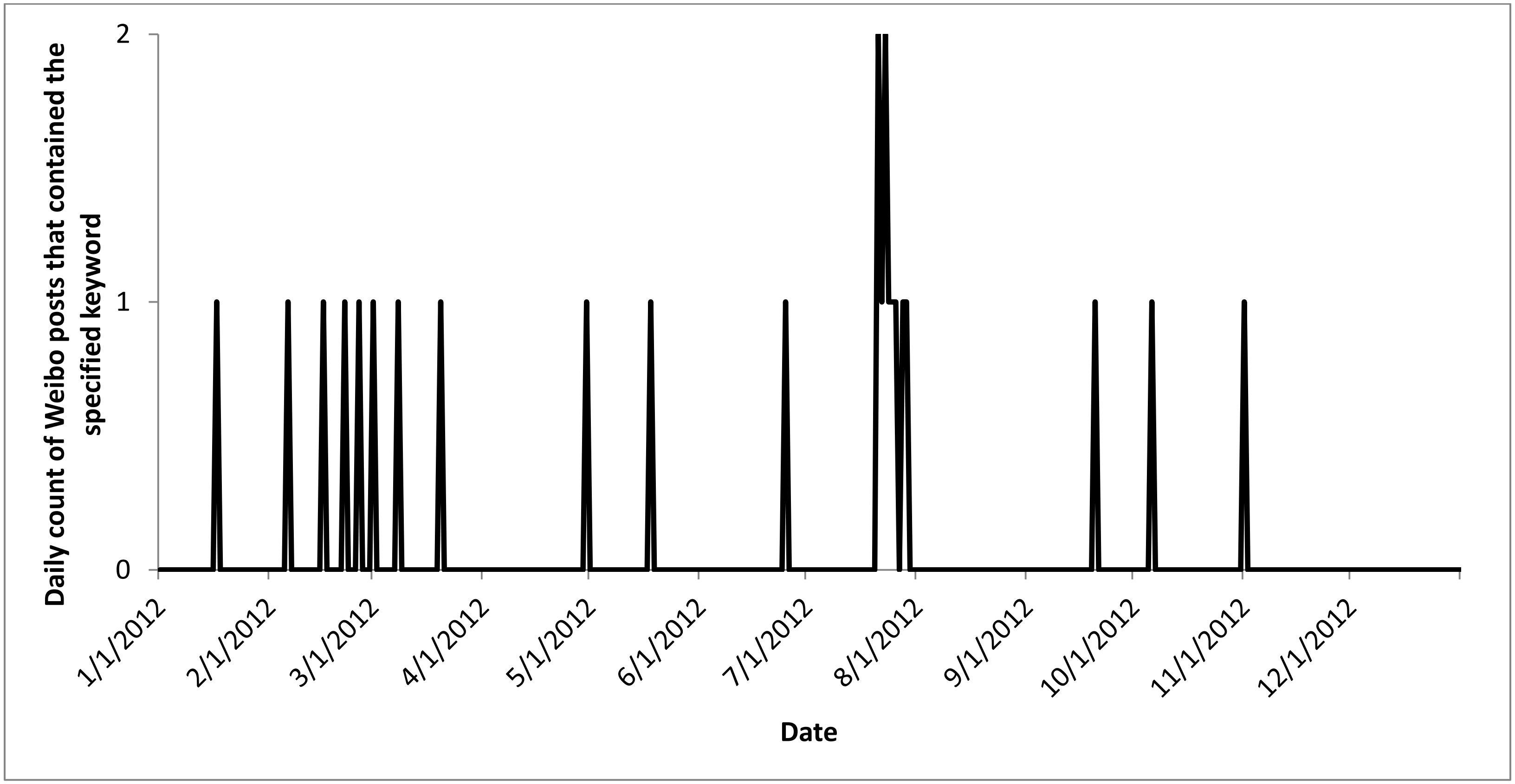

As the total number of Weibo posts was very small, content analysis was performed for Weibo posts of the whole year of 2012.

Whole year of 2012

Total count = 24

Summary: Of the 24 Weibo posts that mentioned “leishmaniasis” (黑热病) in the whole year of 2012, 13 posts were promotional materials about Médecins Sans Frontières medical work on leishmaniasis, three posts about medical history, two about health insurance in Gansu Province, five about health information, and one personal comment.

Table S38. Sample Weibo posts mentioning the keyword for “leishmaniasis” (黑热病) in 2012

Sample Weibo posts on that day	English translation / description	Count (%)	Sources / References / Notes	Category
3 月 15 日是首个“黑热病日”，黑热病这种被忽略的热带疾病，是 76 个国家的风土病，每年导致全球五万人死亡，带来的挑战因环境而异，但一般影响的都是全球最贫穷和最脆弱的人民。若未能获得治疗，几乎所有病人都会死亡。请立即点击，与无国界医生一同关注黑热病，为病人争取更佳诊治 http://t.cn/zOJlgIn	March 15 is the first “Leishmaniasis Day”. Leishmaniasis, this neglected tropical disease, is endemic in 76 countries, causing 50,000 deaths globally. The challenges it brings are different depending on the environment. However, generally, whom it affects globally are always the poorest and the weakest people. If untreated, nearly every patient will die. Please click immediately and care about leishmaniasis. http://t.cn/zOJlgIn	13 (54.17%)	This sample Weibo post contained a link to the Médecins Sans Frontières Hong Kong website http://www.msf.org.hk/index.php?option=com_content&view=article&id=1508&Itemid=9&lang=sc<br><br>Note: This is a group of 13 different posts promoting MSF work on leishmaniasis.	Health information (MSF work on leish-maniasis)
#历史的今天# 我国著名热带医学专家钟惠澜逝世 钟惠澜，1901 年生，广东嘉应（今梅县）人。1939 年在国内首次阐明犬、人、白蛉在黑热病传染环节上的关系，倡用骨髓穿刺方法，检查黑热病利什曼原虫，对黑热病的预防、早期诊断和治疗作出了重要贡献。1987 年 2 月 6 日逝世。	# Today in history # Our country’s famous tropical medicine experts Zhong Huilan died. Zhong Huilan, born in 1901, was from Jiaying (now Meixian), Guangdong. In 1939, in China, he was first to demonstrate the relationship between dogs, humans and sandflies in the transmission of leishmaniasis, advocating the use of marrow puncture to inspect the presence of *Leishmania*. He had made significant contribution to the prevention, early diagnosis, and treatment of leishmaniasis. He died on February 6, 1987.	3 (12.50%)	Note: a group of different Weibo posts about medical history that mentioned leishmaniasis	Health information (medical history)
从甘肃省卫生厅获悉，我省已将克山病、大骨节病、布鲁氏菌病、黑热病、克汀病、包虫病、氟骨症、砷中毒、疟疾等 9 种地方病治疗，纳入 2012 年新型农村合作医疗门诊特殊	Informed by the Gansu Provincial Health Bureau, our province has included 9 diseases that are endemic there (Keshan disease, Kashin–Beck disease, brucellosis, leishmaniasis, cretinism, echinococciosis, skeletal fluorosis, arsenic poisoning and malaria) into 2012 new rural cooperative healthcare outpatient special diseases	2 (8.33%)	http://xb.gansudaily.com.cn/system/2012/02/02/012355369.shtml	Social issue (health insurance)

病种统筹补偿范围，补偿不设起付线，可补偿费用的报销比例为 70%，每人年度累计补偿封顶线为 1000 元。http://t.cn/zOqIdd5	general compensation scheme. There was no co-payment. The proportion of reimbursement is 70%. The annual cumulative maximum amount of reimbursement is 1000 yuan.			
【首个黑热病疫苗进入临床试验】利什曼病有多种形式，其中内脏利什曼病是最糟糕的。若不进行治疗，几乎都会在 2 年内死亡。位于美国西雅图的一个非营利性传染病研究所（IDRI）已准备开展一种疫苗的人体试验，这种疫苗可能使利什曼病成为历史。http://t.cn/zObyNx3	"The first leishmaniasis vaccine enters clinical trial" There are several forms of leishmaniasis, of which visceral leishmaniasis is the worst. If untreated, nearly everyone will die within 2 years. A non-profit Infectious Disease Research Institute has prepared to begin a human vaccine trial. This vaccine may put leishmaniasis into history. http://t.cn/zObyNx3	5 (20.83%)	Note: a group of different Weibo posts that mentioned leishmaniasis	Health information
Others		1 (4.17%)		Personal comment

39. Echinococciosis

Figure S39. Daily count of Weibo posts with the keyword for "echinococciosis" (包虫病) in 2012 among Sina Weibo users with 1000 followers or more.

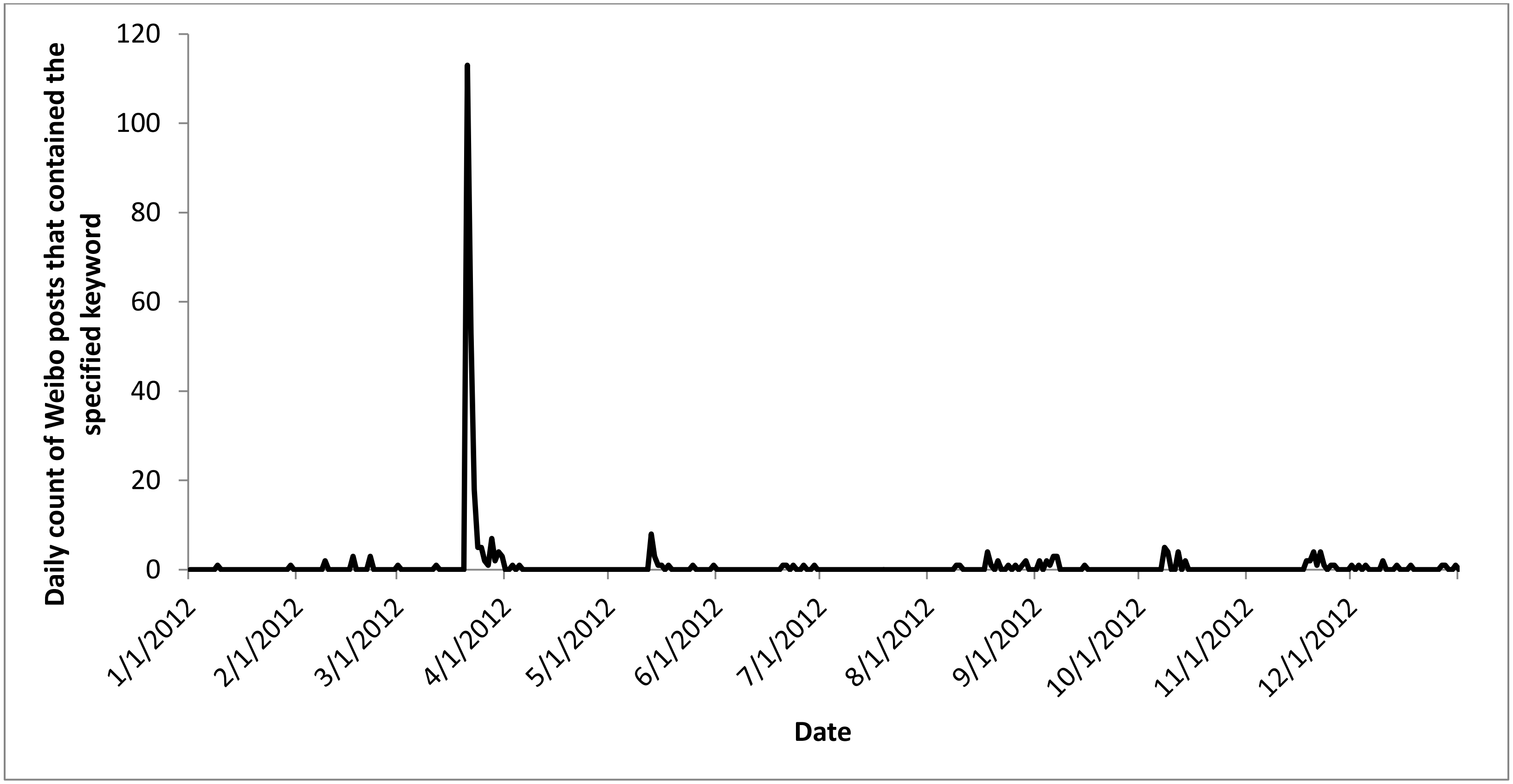

Content analysis was performed for Weibo posts generated on the peak date of March 21, 2012.

Peak Date: March 21, 2012

Total count: 113

Summary: All of the 113 Weibo posts mentioning "echinococciosis" (包虫病), on March 21, 2012, are related to the news of a case of echinococciosis. Of these 113 posts, 105 were news reports or reposts thereof, and the other 8 were personal comments. Since echinococciosis is a rare disease, the news about a young lady paralyzed by a parasite generated a lot of attention on a single day.

Table S39. Sample Weibo entries that contained the keyword for "echinococciosis" (包虫病) on March 21, 2012.

Sample Weibo posts on that day	English translation / description	Count (%)	Sources / References / Notes	Category
**A case of echinococciosis**		**113 (100%)**		**News about a case**
【少女遭虫子噬骨瘫痪 疑因养宠物患病】少女小周患怪病导致下肢瘫痪，医生推测她幼年通过家里的宠物染上包虫病，导致寄生虫长期蚀骨。据悉，小周从 14 岁就开始每半年发一次高烧，最后病情恶化右腿无法站立困瘫在床上。手术后，小周恢复良好已能借工具行走。（大洋网-广州日报） http://t.cn/zOJ39Hw	"Young girl's bone was eaten by worms leading to paralysis May be infected via pets". Young lady Little Zhou got a strange disease leading to paraplegia. Doctors speculated that when she was young, she was infected with echinococciosis via pets kept at home, leading to bones eaten up by parasites. It is reported that Little Zhou had a high fever every half a year since the age of 14. At last, the disease exacerbated and she could not stand with her right leg and was left paralyzed in bed. After surgery, Little Zhou recovered and can now walk with the help of tools. (Da Yang Net – Guangzhou Daily) http://t.cn/zOJ39Hw	105 (98%)	http://hebei.sina.com.cn/health/ynb/2012-03-21/12269.html	News about a case
肝包虫病就是它惹出来的。//@[user] 好可怕的棘球蚴！	Echinococciosis is caused by it! //@[user] Dreadful echinococcus!	8 (7%)	Original Weibo post	Personal comments on the case

40. Filariasis

Figure S40. Daily count of Weibo posts with the keyword for "filariasis" (丝虫病) in 2012 among Sina Weibo users with 1000 followers or more.

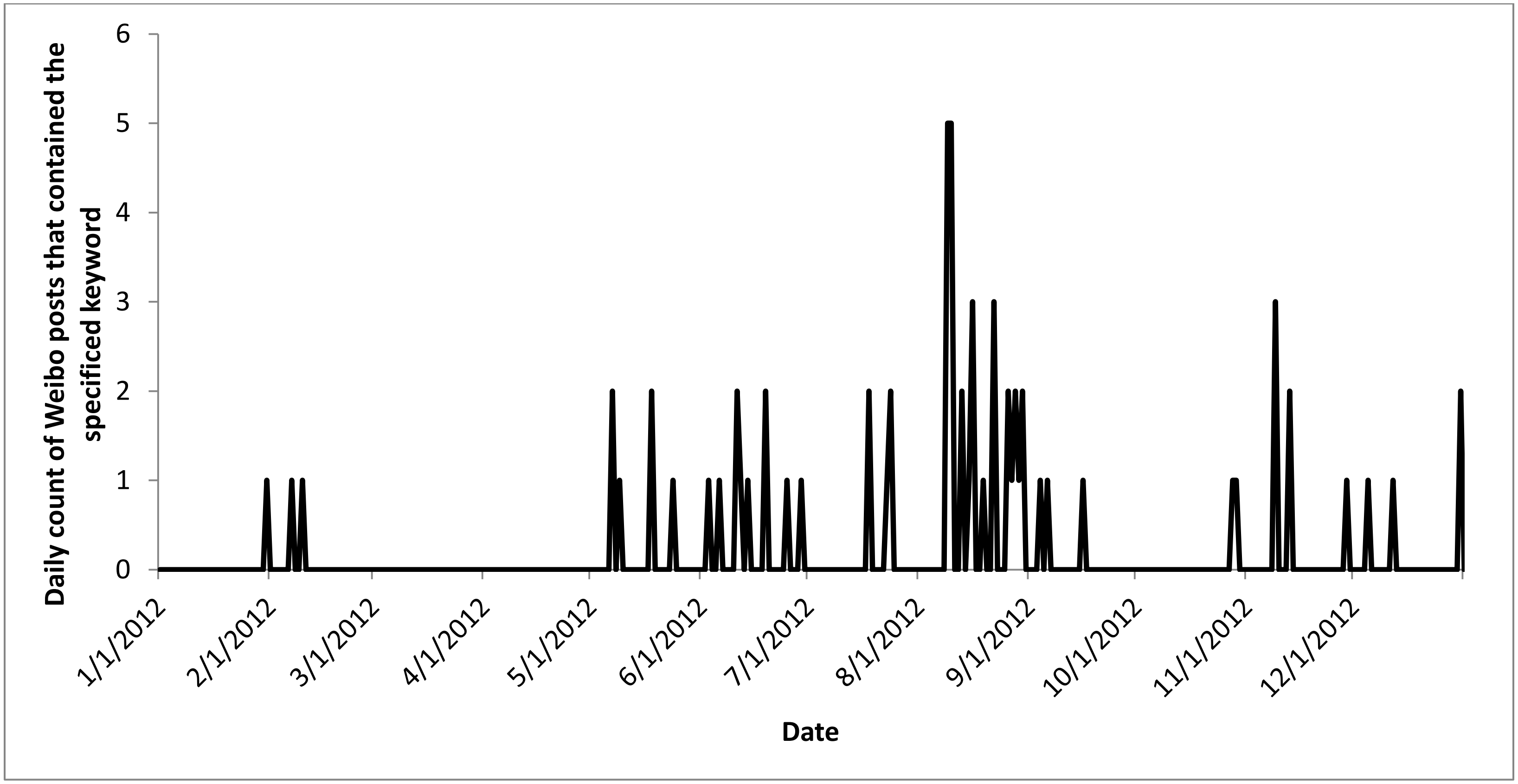

Content analysis was performed for the Weibo posts on the dual peak dates of August 9-10, 2012.

Summary: The peak of Weibo posts that contained the keyword "filariasis" (丝虫病) on August 9 and 10, 2012, consisted of 10 posts over two days (5 posts each day). Nine of the 10 posts were health education posts about four mosquito-borne diseases: epidemic encephalitis B, malaria, dengue and filariasis.

Peak Date: August 9, 2012
Total count: 5

Table S40A. Sample Weibo entries that contained the keyword for "filariasis"(丝虫病) on August 9, 2012.

Sample Weibo posts on that day	English translation / description	Count (%)	Sources / References / Notes	Category
【被蚊子咬了易患哪些病】1、流行性乙型脑炎 多发于儿童，重症患者常留下后遗症，死亡率高；2、疟疾 临床症状和发作规律表现不一，恶性疟疾可侵犯内脏；3、登革热和登革出血热 前者病死率低，后者病死率高；4、丝虫病 早期症状表现为发热、过敏等，后期易形成象皮肿、乳糜尿或腹水。	"After bitten by mosquitoes what diseases are we likely to get" 1. Epidemic encephalitis B. The cases are usually children. Severe cases usually have sequelae. Case fatality rate is high. 2. Malaria. Clinical symptoms and onset patterns are irregular. Severe malaria can infect internal organs. 3. Dengue fever and dengue hemorrhagic fever. The case fatality ratio of the former is low while that of the latter is high. 4. Filariasis. Early symptoms include fever and allergy. Later, it may easily lead to elephantiasis, chyluria or ascites.	4(80%)	http://gd.sina.com.cn/news/jk/2012-07-20/09563539.html	Health education
回复@[user1] 丝虫病，这个不是开玩笑的，希望有医生能提供消毒的办法。//@[user1] 以前我们小时候也这样玩，大人说会生大脚疯！ //@[user2] 刚咨询了医生，洗干净后消毒，先用碘酒再用酒精脱碘。	Reply @[user1] Filariasis. This is not a joke. Hope that doctors can provide a method for disinfection. //@[user1]When we were small, we also played like that. Adults said that we would get elephantiasis! //@[user2] Just consulted a doctor. Disinfection after washing, first use iodine and then use alcohol to de-iodinate.	1(20%)	Original Weibo Post	Personal comment

Peak Date: August 10, 2012
Total count: 5

Table S40B. Sample Weibo entries that contained the keyword “Filariasis” (丝虫病) on August 10, 2012.

Sample Weibo posts on that day	English translation / description	Count (%)	Sources / References / Notes	Category
【被蚊子咬了易患哪些病】1、流行性乙型脑炎 多发于儿童，重症患者常留下后遗症，死亡率高；2、疟疾 临床症状和发作规律表现不一，恶性疟疾可侵犯内脏；3、登革热和登革出血热 前者病死率低，后者病死率高；4、丝虫病 早期症状表现为发热、过敏等，后期易形成象皮肿、乳糜尿或腹水。	“After bitten by mosquitoes what diseases are we likely to get” 1. Epidemic encephalitis B. The cases are usually children. Severe cases usually have sequelae. Case fatality rate is high. 2. Malaria. Clinical symptoms and onset patterns are irregular. Severe malaria can infect internal organs. 3. Dengue fever and dengue hemorrhagic fever. The case fatality ratio of the former is low while that of the latter is high. 4. Filariasis. Early symptoms include fever and allergy. Later, it may easily lead to elephantiasis, chyluria or ascites.	5 (100%)	http://gd.sina.com.cn/news/jk/2012-07-20/09563539.html	Health education

41. Diarrhea

Figure S41. Daily count of Weibo posts that mentioned the keyword for "diarrhea" (腹泻) in 2012 among Sina Weibo users with 1000 followers or more.

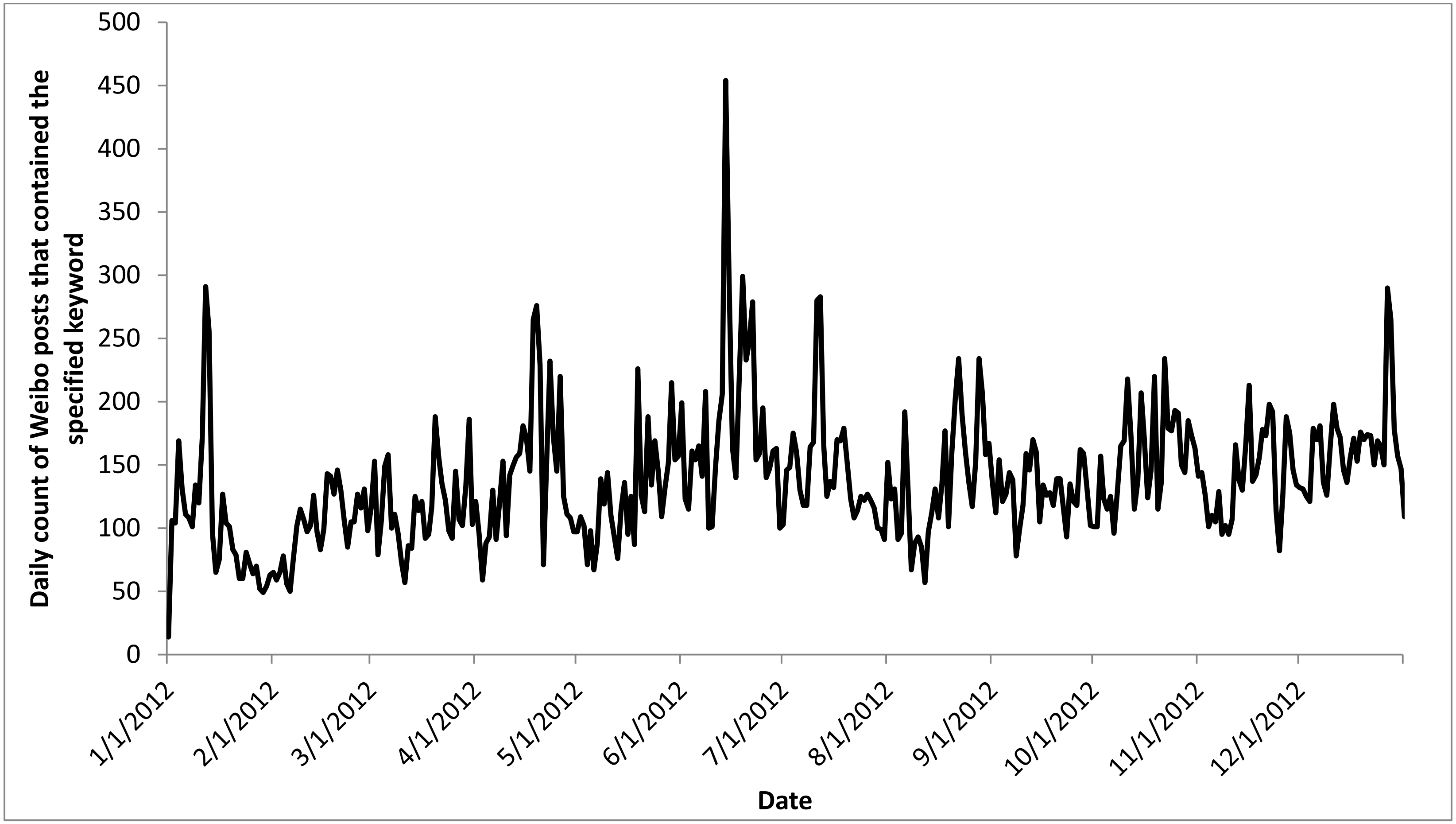

Content analysis was performed for Weibo posts generated on the peak date of June 14, 2012.

Peak Date: June 14, 2012

Total count: 454

Summary: Of the 454 Weibo posts that mentioned "diarrhea" (腹泻) on June 14, 2012, 242 (53%) were about news of cases of diarrhea, 28 cases (6%) of health education/information, 162 (36%) alternative health information/Traditional Chinese Medicine, 4 (0.9%) advertisements, and 18 (4%) personal comments.

Of the 242 posts about news of diarrheal cases, 196 about two diarrheal cases after consuming ham sausages produced by a famous Chinese meat processing company. Another 24 were about a diarrheal case after drinking milk. Another 14 posts were about a funny video about a diarrheal case in a swimming pool. Another 7 posts were about a diarrheal case after consuming mixed ingredient porridge produced by a famous Chinese company. Finally there was one post about a case of a baby having diarrhea due to consuming milk that was not infant formula.

There had been many Weibo posts providing health information about diarrhea, from how to avoid diarrhea but abstaining certain food to how to manage infant's diarrhea. However, we categorized many of these posts as alternative health information/Traditional Chinese Medicine (n=163), as the information contained therein (or at least part of that) might not fit well with a modern medical understanding of diarrhea and its prevention. Many of those posts were related to food, e.g. what to eat and what not to eat in the summer. The very existence of these posts – created or re-posted by Weibo users with 1000 or more followers – indicated that people were interested in how to stay healthy by choosing the right type of food to consume.

Table S41. Sample Weibo entries that contained the keyword "diarrhea" (腹泻) on June 14, 2012.

**Sample Weibo posts on that day**	**English translation / description**	**Count (%)**	**Sources / References / Notes**	**Category**
**News of cases of diarrhea**		**242 (53.30%)**		
【双汇火腿内现蛆虫 孕妇食用后先兆性流产】北京张先生在物美购买两袋双汇火腿肠，姐姐和女儿吃后均出现腹泻呕吐症状，姐姐引起先兆性流产。工商局里，拆开无问题同批次火腿肠，存放一天后均都爬满了白色蛆虫。双汇回应，有可能在流通或储存环节出现了破袋的情况。http://t.cn/zWPsoS1	"Maggots appeared in Shuanghui ham. After eating, pregnany woman experienced threatened abortion" Mr. Zhang of Beijing bought two bags of Shuanghui ham sausage from Wumei. His elder sister and his daughter, after eating the ham sausage, both experienced diarrhea and vomiting symptoms. It even triggered a threatened abortion for his elder sister. In the industry and commerce bureau, after the ham sausages of the same batch have been unpacked and left for a day, there were white maggots crawling all over the sausages. Shuanghui responded that, it could happen that in the process of circulation or storage, the packages were broken. http://t.cn/zWPsoS1	196 (43.17%)	http://news.cntv.cn/society/20120614/100206.shtml Shuanghui is a major meat processing company in China.	News of two cases of diarrhea
【蒙牛特仑苏牛奶又致消费者拉肚子】中国乳制品行业正处在最好的时代？6 月 9 日，山东济南市民李女士购买 1 箱生产于 3 月 10 日、保	"Mengniu Deluxe Milk causes diarrhea for a consumer" Is the dairy industry in China experiencing its best epoch? On June 9, Ms. Li, a citizen of Jinan, Shandong, bought a box of Mengniu Deluxe Milk, that was produced on March 10 with a preservation period of 6 months. After drinking the milk, her family experienced	24 (5.29%)	http://health.sohu.com/20120613/n345526560.shtml	News of cases of diarrhea

质期 6 个月的蒙牛特仑苏牛奶，家人喝后腹泻不止。济南蒙牛先表示赔偿 1 箱奶，后又增至 2 箱，最终商定赔 10 箱。据人民网 http://t.cn/zWPn3iB	non-stop diarrhea. Mengniu at Jinan expressed that they will compensate Ms. Li with one box of milk. Later they increased the compensation to two boxes. At the end, they agreed to a compensation of 10 boxes. According to People's Net http://t.cn/zWPn3iB			
【视频】在泳池突然腹泻，哥不淡定了，这是猛人，最后是亮点。。。】 http://t.cn/zWPsApS	"Video" Sudden diarrhea in a swimming pool. The guy is not calm. This is a brilliant guy. The very end is the focus… http://t.cn/zWPsApS	14 (3.08%)	http://www.tudou.com/programs/view/i5lmZq6XJNE/?resourceId=92873908_08_12_99&rpid=92873908	A funny video about a case of diarrhea
【娃哈哈八宝粥质量难保证解决问题多用"以粥换粥"】被频繁投诉娃哈哈桂圆莲子八宝粥又出问题。网友称"打开后现果冻状。老人吃两罐出现腹泻恶心情况。"地方销售负责人称产品没质量问题,提出赔偿 8-10 件娃哈哈八宝粥解决此事。此前该产品屡现异物：烟蒂、塑料、虫子丨 http://t.cn/zWPJvCX	"Wahaha cannot guarantee the quality of its mixed ingredient porridge Problem solution is primarily "exchanging porridge with porridge"" The frequently complained Wahaha Longan seed porridge had problems again. A netizen said, "Once opened, it looks like jelly. After eating two cans of the porridge, an elderly person experienced diarrhea and nausea." The local sales person-in-charge said that there was no quality problem with the product and proposed a compensation of 8-10 cans of Wahaha mixed ingredient porridge to settle the dispute. Previously, this product was found frequently with strange thing: cigarette butts, plastics and worms. http://t.cn/zWPJvCX	7 (1.54%)	http://finance.china.com.cn/consume/tbch/20120613/798446.shtml 八宝粥(Bābǎozhōu) is a porridge or congee with a mixture of ingredients. Its name can be translated literally, as "porridge with eight treasures".	News related to a case of diarrhea
【营业员错把孕妇奶粉当婴儿奶粉卖给消费导致 4 个月大宝宝腹泻不止】近日，一奶粉专卖店营业员将孕妇奶粉当做婴儿奶粉销售给消费者，导致 4 个月大婴儿因错食孕妇奶粉腹泻不止，送往医院治疗仍未能改善，对此专家表示婴幼儿长期措施奶粉不但会导致腹泻，还有可能引发生理紊乱 http://t.cn/zWhxxzO	"A shop assistant mistook pregnant women formula for infant formula and sold it to consumers. This led to continuous diarrhea of a 4-month-old baby." Recently, a shop assistant of a milk powder shop mistook pregnant women formula for infant formula and sold it to consumers. This led to continuous diarrhea of a 4-month-old baby. Even after delivery to the hospital, its condition has not improved. Regarding this case, experts said that if babies and young children drink the wrong formula for a long time, this will lead to diarrhea, and may cause other physiological disorders. http://t.cn/zWhxxzO	1 (0.22%)	http://www.qianyecao.com/news/shipinanquan/10029.html	News about a case of diarrhea
**Health education / Health information**		**27 (5.95%)**		

【护理腹泻宝宝的 3 大要点】①补水：为防止宝宝出现脱水现象，可以给腹泻宝宝饮用葡萄糖盐水(白糖水也可以)②保暖，爸爸妈妈可适当地用热水袋对宝宝腹部进行热敷，也可以帮宝宝轻揉肚子，以缓解其疼痛。③护臀，患病宝宝用过的东西要及时洗涤并进行消毒处理，以免发生交叉感染。	"3 main points to take care of babies experiencing diarrhea" 1. Rehydration: To prevent babies from dehydration, one can give babies experiencing diarrhea to drink glucose salt water (plain sugar water is also OK). 2. To keep them warm. Parents can duly use hot water bag as a heating pad for the baby's stomach, and can massage the baby's stomach, to relieve its pain. 3. Everything that the sick baby has used, including nappies, should be duly washed and disinfected, to avoid cross-infection.	6 (1.32%)	http://baby.163.com/10/0401/11/636BTEM700262I25.html	Health education
【"拉肚子"高峰期到来】随着夏季到来，气温升高，本市也进入肠道传染病发病高峰期，主要表现为腹泻，也就是"拉肚子"。昨天，市疾控中心提醒市民不食用腐败变质的食品；一旦患病，不要自行服用抗菌素治疗，要尽快到就近的肠道门诊就医；病人污染的用具要用漂白粉、84 等消毒。	"The peak of diarrhea has arrived" As summer is approaching, the temperature increases, our city has also reached the epidemic peak of enteric infections. The primary symptom is diarrhea, also known as "pulling the stomach" [in lay Chinese terms]. Yesterday, the municipal Center for Disease Control and Prevention advises citizens not to eat rotten food. If one is sick, do not treat yourself with antibiotics. Visit an enteric disease outpatient clinic as soon as possible. Utensils used by the patient should be disinfected with breaching powder or "84 disinfectant".	5 (1.10%)	http://www.morningpost.com.cn/szb/html/2012-06/12/content_166028.htm<br>Note: "84 disinfectant" is a generic name given to a type of disinfectant first developed by Beijing Ditan Hospital in 1984.	Health education
【经常熬夜小心炎症性肠病】经常熬夜、长期疲劳、吸烟、精细饮食等，都是可能诱发炎症性肠病的主要原因，表现一般都是腹泻、腹痛，甚至会出现血便。在日常生活中，患者需要注意饮食调理，把握食物质软、易消化、高营养的原则，少食多餐、定时定量。	"Staying up late frequently. Beware of inflammatory bowel disease" Staying up late frequently, long-term tiredness, smoking and fine dining etc., are major reasons that can trigger inflammatory bowel disease. The common symptoms are diarrhea and stomach ache, and even blood stool. In their daily lives, patients should pay attention to regulate their diets. They should uphold the rules to eat food that is soft, easy to digest and of high nutrition values. They should eat more frequently but with less quantity, as well as in constant volume in a regular manner.	3 (0.66%)	Original Weibo post	Health education
夏季 BB 腹泻该怎用药？夏季腹泻高发期。宝贝腹泻时要观察是否有脱水现象：如哭	Babies having diarrhea in the summer. How should we use medication? Summer is the epidemic peak of diarrhea. When babies experience diarrhea, one should	3 (0.66%)	Original Weibo post	Health education

时泪少或无泪，尿少，说明脱水很严重，要立即补液。母乳喂养的宝宝腹泻，请继续喂。牛奶喂养的，可将奶冲稀点或换成无乳糖奶粉(腹泻奶粉)。大点 BB，则建议停止牛奶和肉蛋等食物，吃点稀饭或面条就可，腹泻止吃药。	observe if there is any sign of dehydration, such as little or no tear when crying, little urine, which indicates a severe dehydration, which will require immediate fluid infusion. If breast-fed babies experience diarrhea, please continue breast feeding. For babies that were fed with cow milk, one can dilute it a little bit, or change it to lactose-free infant formulae (formulae for babies with diarrhea). For babies that are older, we recommend to stop feeding with milk and eggs. Just eat some congee or noodles will be fine, and take some anti-diarrhea medication.			
5 月份，湖南省共报告丙类传染病 8 种 53698 例，死亡 33 例（手足口病）。报告发病数居前 5 位的病种为：手足口病 47663 例、流行性腮腺炎 2864 例、其它感染性腹泻病 1914 例、风疹 695 例、流行性感冒 387 例，共报告 53523 例，占本月丙类传染病报告发病总数的 99.69%。	In May, Hunan Province reports 8 types of Class C infectious diseases, 53,698 cases with 33 deaths (hand-foot-and-mouth disease). The top 5 diseases in terms of incident cases in the report are: hand-foot0-and-mouth disease (47,663 cases), mumps (2864 cases), other diarrheal diseases (1914 cases), rubella (695 cases), influenza (387 cases), a total of 53,523 cases, accounting for 99.69% of incident cases of Class C infectious diseases this month.	3 (0.66%)	http://hn.qq.com/a/20120614/000090.htm	Health information (Official statistics of diarrhea in Hunan Province)
宝宝腹泻拉肚子怎么办：http://t.cn/zWhwpzS（文章来自：父母路）	How one can handle a baby with diarrhea: http://t.cn/zWhwpzS (Source: Parents' road)	1 (0.22%)	Link to this post: http://fumulu.com/post/112.html	Health education
奶酪能增进人体抵抗疾病的能力，促进代谢，增强活力，保护眼睛健康并保护肌肤健美。奶酪中的乳酸菌及其代谢产物对人体有一定的保健作用，有利于维持人体肠道内正常菌群的稳定和平衡，防治便泌和腹泻。英国睡科医生认为，吃饭时吃一些奶酪，有助于防止龋齿，增加牙齿表层的含钙量。	Yoghurt can help improve the human body's ability to defend itself against illness. It helps improve metabolism, energizes the body, keep the eyes healthy and maintain the beauty of the skin. The lactobacillus in yoghurt and their metabolites have certain health effects on the human body. They are beneficial to the body as they maintain the stability and balance of the normal bacterial flora in the human intestines and can help prevent constipation and diarrhea. British doctors think that consumption of some cheese at meals helps prevent caries and increase the calcium content of the surface of the teeth.	1 (0.22%)	Original Weibo post	Health education
【宝宝出现这 10 种症状必须	"If kids experience these 10 symptoms, they should be	1 (0.22%)	Original Weibo post	Health

送急症】1、出现将近 40 度高热;2、咳嗽声如破竹并呼吸急促;3、婴幼儿剧烈呕吐;4、一天腹泻超过 4 次;5、呼吸困难或者急促;6、吐血、咯血、便血、衄血及各种外伤出血;7、惊厥超过 3 分钟;8、发热、呕吐或腹泻时脱水;9、头部受伤;10、意外伤害。健康关注	delivered to emergency care” 1. Fever, approaching 40 degrees [Celcius]. 2. Cough sound like bamboo and shortness of breath. 3. Infants and toddles experience severe vomiting. 4. Diarrhea over 4 times in a single day. 5. Difficulty or shortness of breath. 6. Hematamesis, hemoptysis, blood in the stool, bleeding not caused by injury and bleeding caused by injury. 7. Seizures for over 3 minutes. 8. Dehydration when experiencing fever, vomiting or diarrhea. 9. Head injury. 10. Accidental injury. Pay attention to health.			education
【腌肉好吃别过量 警惕亚硝酸钠中毒】亚硝酸钠是工业盐，人食用 0．2 克到 0．5 克可能出现中毒症状，表现为头晕乏力、气短、恶心呕吐、腹泻、抽搐、昏迷，甚至危及生命。 另外亚硝酸钠在体内会生成致癌物。新腌制泡菜中含亚硝酸钠，所以泡菜最好在淹制 15 天后食用，当中的亚硝酸盐含量会逐渐降低。	“Preserved meat is tasty but do not eat too much. Beware of sodium nitrite poisoning” Sodium nitrite is industrial salt. Intake of 0.2 – 0.5g may lead to poisoning. Manifestations include dizziness, fatigue, shortness of breath, nausea, vomiting, diarrhea, convulsions and coma. This may even be fatal. Furthermore, sodium nitrite is carcinogeneous. Newly preserved kimchi contains sodium nitrite. Therefore, it is best to consume kimchi after it had been preserved for 15 days. By then the sodium nitrite contents would have decreased gradually.	1 (0.22%)	Original Weibo post	Health education
【腹泻怎样办】吃隔夜剩菜饭，或吃生冷、不洁的食物，容易造成急性肠胃炎。医生建议，腹泻后要多喝水，防止造成脱水和钠、钾电解质紊乱，此时以补充糖盐水（100 毫升水＋11 克白糖＋0.9 克盐）最为适宜。另外，可适当补充维生素 B，有助于调节神经功能、帮助消化、消除疲劳。	“How one can manage diarrhea” The consumption of left over food, or uncooked, unclean food, may easily lead to acute gastroenteritis. Doctors advise that after diarrhea, one should drink more water, to avoid dehydration and sodium and potassium electrolyte imbalance. At that moment, the most appropriate is to supplement with sugar-salt water (100 ml of water + 11 gram of white sugar + 0.9 gram of salt). Furthermore, one can supplement oneself with vitamin B. It will help regulate the functions of the nervous system, facilitate digestion and relieve fatigue.	1 (0.22%)	Original Weibo post	Health education (oral rehydration salt)
因为夏季穿衣物较少、儿童在家时间较长等原因，儿童伤害中约 40%发生在夏季。	Because in the summer children wear fewer clothes, and spend more time at home, 40% of injury among children happen in the summer. Drowning, diarrhea caused by food poisoning, scalding and bruises are	1 (0.22%)	Original Weibo post	Health education

溺水、食物中毒导致的腹泻、烧烫伤、碰伤是夏季儿童常见的意外伤害。	common accidental injuries experienced by children in the summer.			
【保质期仅三五天 喝扎啤需留意生产日期】炎炎夏季，扎啤受到偏爱。但你是否知道：扎啤是一种普通的生啤酒，因未经过高温杀菌，其保质期较短，一般在 3—5 天。新鲜扎啤口感清爽，不新鲜的则会出现刷锅水味、酸味等。一旦误饮，轻者致腹泻、肠炎等症状，重则威胁生命。 http://t.cn/S4mDKP	"Shelf life is around 3 to 5 day long. Drinking draft beer need to take note of the date of production." In a hot summer, many people prefer draft beer. But do you know that draft beer is not pasteurized, and their shelf life is relatively short, usually 3-5 days. Fresh draft beer tastes fresh, while draft beer that is no longer fresh will taste like water used for dishwashing and will taste acidic. If it is consumed by mistake, it may lead to symptoms such as diarrhea or enteritis. In severe cases, it may be life-threatening. http://t.cn/S4mDKP	1 (0.22%)	Original Weibo post	Health education
**Alternative health information / Traditional Chinese Medicine**		**163 (35.90%)**		
【夏日养生：夏天吃西瓜的禁忌】1、糖尿病患者要少吃。吃西瓜后会致血糖增高。2、肾功能不全者。易诱发急性心力衰竭。3、感冒初期。会使感冒病情加重或病程延长。4、口腔溃疡病人。5、产妇。产妇吃多西瓜会因过寒而损脾胃。6、不宜在饭前及饭后吃。7、西瓜不宜吃得太多，易引起腹胀、腹泻。	"Keeping healthy in the summer: taboo of eating watermelon in the summer" 1. Diabetics should eat less watermelon, since after eating watermelon, the blood sugar level will increase. 2. People who have renal insufficiency. Eating watermelon would easily trigger acute heart failure. 3. Early phase of common cold. Eating watermelon would exacerbate the symptoms of common cold or extend its duration. 4. Patients with mouth ulcers. 5. Pregnant women. If pregnant women eat many watermelons, their spleen and stomach would be damaged because they are too cold. 6. Do not eat watermelon before meals or after meals. 7. Do not eat too many watermelons. This will easily lead to bloating and diarrhea.	36(7.93%)	Original Weibo post	Alternative health information / Traditional Chinese Medicine
【不同的水果，调理出来不同的健康】1・过度用脑：香蕉；2・过度用眼：番木瓜；3・牙龈出血：猕猴桃；4・心脏病史：葡萄柚；	"Different fruits attend to the needs of different health conditions" 1. Overworked mind: banana; 2. Overtired eye: papaya; 3. Bleeding gums: kiwi fruit; 4. A history of heart disease: grapefruit; 5. Long-term smoking: grape; 6. Muscle strain: pineapple; 7. To prevent winkles: mango; 8. Inadequate supply of oxygen in the	17(3.74%)	Original Weibo post	Alternative health information

5・长期吸烟：葡萄；6・肌肉拉伤：菠萝；7・预防皱纹：芒果；8・血液供氧不足：樱桃；9・腹泻：苹果；10・胃痛：木瓜；11・消化不良：梨子、草莓、枇杷。	blood: cherry; 9. Diarrhea: apple; 10. Stomachache: papaya; 11. Indigestion: pear, strawberry, loquat.			
【哪几类人不宜吃冰激凌】1 欲控制体重人群：冰激凌糖和脂肪含量都很高，这种特点能促使人变胖；2 孕妇：因为孕妇消化液和消化酶分泌减少，胃肠功能减弱，冰激凌会使胃肠血管突然收缩，出现腹痛腹泻；3 孩子：冰激凌易引起青少年肥胖；4 肠胃功能不好者：低温会加重病情。http://t.cn/zWPgAuG	"Which types of people should not eat ice-cream" 1. People who would like to control body weight: The sugar and fat ingredients are high in ice-cream. This characteristic will make people to grow fat. 2. Pregnant women: Because the secretion of digestive juices and digestive enzymes secretion decrease among pregnant women, the functional capacity of the stomach and the intestines decreases. Ice-cream will make the blood vessels of the stomach and the intestines suddenly contract, leading to stomachache and diarrhea. 3. Children: Ice-cream would easily make adolescents fat. 4. People with poor stomach and intestine functions: Low temperature will exacerbate the symptoms. http://t.cn/zWPgAuG	10(2.20%)	http://health.sina.com.cn/hc/2012-06-14/071936477.shtml	Alternative health information
【不能随便吃的蔬果，你懂吗？】1．芒果止血，来月经的人不要吃；2．韭菜壮阳，小孩子不要多吃；3．南瓜升压、止喘，高血压的人不要吃；4．山药补气，失眠的人不要吃；5．蘑菇、黑木耳泻下，腹泻的人不要吃；6．香菜发汗，汗多的人不要吃；7．芋头、苦瓜、雪里红清热，阳痿的人不要吃	"Vegetables and fruits that cannot be eaten casually. Do you know?" 1. Mango stops bleeding. People experience menstruation should not eat mangoes. 2. *Allium tuberosum* is aphrodisiac. Children should not eat much. 3. Pumpkin increases blood pressure and is antiasthmatic. People with high blood pressure should not eat pumpkins. 4. Chinese yam replenish one's *qi*, people experiencing insomnia should not eat. 5. Mushrooms and *Auricularia auricula-judae* are laxatives. People who experienced diarrhea should not eat them. 6. Coriander makes people sweating. People with lots of sweats should not eat coriander. 7. Taro, bitter melon, and *Brassica juncea var. crispifolia* "clear heat", impotent people should not eat.	10(2.20%)	Original Weibo post	Alternative health information
【女性必知的 7 个健康常识】1、皮肤不好是食盐过多;2、经期腹泻不是正常现象：内分泌水平失衡所致;3、紧急避孕药只能一年吃一次：卵巢	"7 pieces of general health knowledge that women must know" 1. Unhealthy skin is due to over-intake of salt. 2. Diarrhea during menstruation is not a regular phenomenon: this is due to imbalance in internal secretion. 3. Emergency birth control pill can only be	10(2.20%)	Original Weibo post	Alternative health information

肝肾代谢紊乱;4、每日化妆会增加患癌概率;5、黑眼圈可能跟妇科疾病有关；6、每天一杯豆浆能去掉小腹：阻断糖变脂肪过程；7、辣椒是好补品：VE 并加速代谢。	taken once a year: otherwise, there will be disorders in the metabolism of the ovary, liver and kidney. 4. Putting on make-up every day will increase the probability of getting cancer. 5. Having dark circles around the eyes may be related to gynecological diseases. 6. A daily cup of soya milk can remove abdominal fat: interrupting the process of the transformation of sugar into fat. 7. Chili peppers is a good supplement: VE plus acceleration of metabolism.			
【注意！相克的食物不能一起吃！】1.咖啡+香烟：易致胰腺癌。2.梨+开水：吃梨喝开水，必致腹泻。3.洋葱+蜂蜜：同食会伤眼睛，引起眼睛不适，严重会失明。4.西红柿+地瓜：同食易得结石病、呕吐、腹痛、腹泻。5.豆腐+蜂蜜：同食严重时可致耳聋。为了您和家人的健康，收藏起来吧	“Attention! Food that is incompatible with each other should not be eaten together!” 1. Coffee + Cigarette: These make one prone to pancreatic cancer. 2. Pears + Boiled water: Eating pears and drinking boiled water, would lead to diarrhea. 3. Onions + Honey: Consumption of both will hurt the eyes, causing eye irritation. In severe cases, this will lead to blindness. 4. Tomato + sweat potato: Consumption of both together will easily lead to gall stone, vomiting, stomach ache and diarrhea. 5. Tofu + honey. Consumption of both will lead to dumbness. For the health of yourself and your family, preserve this post.	5(1.10%)	Original Weibo post	Alternative health information
【宝宝巧喝水，远离 7 种病】入夏了，怎样喝水最科学？1、腹泻、呕吐：喝白开水、小米汤、苹果水、胡萝卜水；2、解暑：白开水、西瓜水、酸梅汤；3、感冒、发烧：白开水、鲜果汁、淡盐水；4、咳嗽痰多：白开水、梨水、荸荠水；5、便秘：白开水、白萝卜水、青菜水。欢迎关注	“Kids drink water smartly Staying away from 7 diseases” As it is summer now, what is the most scientific way to drink water? 1. Diarrhea, vomiting: drink boiled water, millet water, apple water, carrot water; 2. To relieve the summer heat: boiled water, water mellon water, sour plum soap; 3. Common cold, fever: Boiled water, fruit juice, slightly salted water; 4. Coughs with lots of sputum: boiled water, pear water, chestnut water; 5. Constipation: boiled water, radish water, cabbage water. Welcome you to follow [this Weibo account].	5(1.10%)	Orginal Weibo	Alternative health information
【虾和什么不能一起吃】1、和维生素 C 同食会可致砷中毒 2、南瓜同食会引起痢疾，可以用黑豆、甘草解毒 3、果汁同食会腹泻 4、黄豆同食会	“What cannot be consumed with shrimps together” 1. Consumption of shrimps of vitamin C may lead to arsenic poisoning; 2. Consumption of shrimps with pumpkins will lead to dysentery. One can detoxicate with black beans and Chinese liquorice. 3. Consumption of shrimps with fruit juice will lead to	4(0.88%)	http://www.69jk.cn/yinshi/yswq/112092.html	Alternative health information

消化不良 5、红枣同食会中毒。虾忌金瓜,食则拉痢。解救:吃黑豆或甘草水 6、同猪肉、鸡肉同食，同食会产生肝肾衰竭 7、河虾与番茄同食易食物中毒	diarrhea. 4. Consumption of shrimps with soy beans will lead to indigestion. 5. Consumption of shrimps with *Gymnopetalum chinense*, will lead to dysentery. Shrimps should not be consumed with pumpkins. Otherwise, it will lead to dysentery. Solution: To take black beans and Chinese liquorice water. 6. Consumption of shrimps with pork and chicken. This will lead to liver and kidney failure. 7. Consumption of river shrimps with tomatoes, one will be prone to food poisoning.			
【夏天吃水果的禁忌】1.苹果：冠心病、心肌梗塞、肾病、糖尿病的人不宜多吃。2.甜瓜：不宜大量常吃，易伤胃，引起消化不良或腹痛腹泻。3.桃：胃肠功能不良者及老人小孩不宜多吃，易伤胃及胸闷。4.荔枝：性热，阴虚火旺、舌苔发红、有内热的人不宜多吃，易会引发恶心腹泻、头晕、四肢无力的不良反应。	"Fruit consumption taboos in the summer" 1. Apples. People with coronary heart disease, myocardial infarction, kidney disease and diabetes should not eat many apples. 2. Melons: Do not frequently eat a large quantity. This will cause damage to the stomach, causing to indigestion, stomach ache and diarrhea. 3. Peaches: People with poor digestive functions, the elderly and children should not eat many peaches, as peaches may damage the stomach and cause chest tightness. 4. Lychee: People with a "hot quality" (*xing re*), a weak *yin* and a "flaming fire" (*yin xu huo wang*), a red tongue coating, and with "internal heat" (*nei re*) should not eat many lychees, as one will be prone to bad reactions, such as, nausea and diarrhea, drowsiness, and limb weakness.	3(0.66%)	Original Weibo post	Alternative health information / Traditional Chinese Medicine
【适合宝宝冬季吃的 7 种食物】1、黄豆：唯一能够替代动物蛋白的植物性食品，称为"绿色乳牛"。2、胡萝卜：是最适合咳嗽的宝宝用以食疗。3、卷心菜：对帮助宝宝预防贫血有很好的作用。但消化功能差、腹泻的宝宝不宜食用。4、红薯：营养丰富、易于消化，可提供给宝宝大量的热量。有黑斑的勿	"7 types of food that are suitable for babies to eat in winter" 1. Soy: the only vegetarian food product that can replace animal protein. It is called "green milk cow". 2. Carrots: The best dietotherapy for kids suffering from coughs. 3. Cabbage: It is very beneficial as it prevents kids from anemia. But kids with poor digestive functions or diarrhea should not eat cabbage. 4. Sweet potatoes: Nutritious, easy to digest, sweet potatoes can provide a large quantity of energy. Sweet potatoes with black spots should not be consumed.	3(0.66%)	Original Weibo post	Alternative health information

用。				
【家中七大急救药】1、粗盐—用布或毛巾裹着敷于扭伤处缓解疼痛；2、苹果—帮助缓解腹泻；3、糖—缓解打嗝；4、小黄瓜—敷切片能止痒；5、香菜混合米酒—缓解瘙痒；6、姜—姜汁和姜末含在嘴里止吐；7、大蒜水—取六瓣大蒜，将其压碎，并加入温水，每天喝两次，连服三天即可缓解咳嗽。	“7 first-aid medication at home” 1. Coarse salt: Apply to twist injury baddaged with a cloth, to help alleviate pain. 2. Apples: help relieve diarrhea. 3. Sugar: helps alleviate hiccups. 4. Cucumber: apply slices of cucumber helps alleviate itchiness. 5. Bok choy mixed with rice wine: to soothe itchiness. 6. Ginger: Ginger juice and ginger powder can be used as antiemetic by holding them in the mouth. 7. Garlic water. Take 6 peels of garlic. Press them into pieces. Add warm water. Drink twice a day for three days. This will alleviate coughs.	3(0.66%)	Original Weibo post	Alternative health information
【黄瓜混搭怎么吃？】两种错误吃法：1、和花生搭配，虽然口感好，但容易腹泻，建议肠胃不好的人少食；2、与辣椒、芹菜配，降低对黄瓜中 Vc 的吸收。两种健康吃法：1、与木耳搭配，排毒、减肥功效好，黄瓜中丙醇二酸抑制脂肪转化，木耳则吸附体内杂质；2、与豆腐搭配，解毒消炎、润燥平胃。	“What should one eat together with cucumber?” Wrong combinations: 1. With peanuts: Even though it tastes good, but it will easily lead to diarrhea. People with unhealthy intestines and stomach are advised to avoid this combination. 2. With peppers and celery: this combination will lower the absorption of vitamin C in cucumber. Two healthy combinations: 1. With cloud ear fungus. It is effective for detoxication and slimming. Tartronic acide in cucumber suppresses lipid transformation. Cloud ear fungus can absorb impurities in the body. 2. With tofu. It is effective for detoxication, anti-inflammatory effect, moistening effect and soothing the stomach.	3(0.66%)	Original Weibo post	Alternative health information
【香蕉的十大健康功效】1.防治胃肠溃疡 2.治疗皮肤瘙痒症 3.降低胆固醇血清 香蕉的果柄具可降低胆固醇 4.治疗高血压 5.治疗忧郁症 6.治疗咳嗽 7.治疗痔疮及便血 8.减肥 9.美容 使皮肤清爽润滑，去除脸上痤疮，淡化雀斑 10.解酒 提醒：香蕉性寒，脾胃虚寒、胃痛腹泻的患者应少用。	“Top ten health effect of banana” 1. To prevent gastrointestinal ulcers. 2. To treat skin itchiness. 3. Lower serum cholersterol. Banana stalk can reduce cholesterol. 4. To treat high blood pressure. 5. To treat depressure. 6. To treat cough. 7. To treat hemorrhoids and blood in the stool. 8. Slimming. 9. Comestic use. To make the skin fresh and smooth. To remove the face acne and freckles. 10. To ease hangover. Reminder: the nature (*xing*) of banana is “cold”, people with a weak digestive system, with stomach ache and diarrhea, should eat less banana.	2(0.44%)	Original Weibo post	Alternative health information / Traditional Chinese Medicine

【5 种食物，宝宝不宜多吃】1、桔子：容易产生＂叶红素皮肤病＂、腹痛腹泻，甚至引起骨病。；2、菠菜：影响骨骼、牙齿发育；3、鸡蛋：容易造成营养过剩，导致肥胖，还能增加胃肠、肝肾的负担，引起功能失调。4、果冻：影响儿童的生长发育和智力健康。5、罐头：易造成慢性中毒。	"5 types of food that babies should not eat much." 1. Tangerine: may easily cause "Erythroderma", stomach ache and diarrhea, and even causing bone diseases. 2. Spinach: affects the development bones and teeth. 3. Eggs: easily lead to over-nutrition, leading ot overweight, and can also increase the burden on the stomach, intestines, liver and kidney, causing disfunction of these organs. 4. Jelly: affects the bodily development and intellectual health of children. 5. Canned food: may easily cause chronic poisoning.	2(0.44%)	Original Weibo post	Alternative health information
【宝宝喝酸奶 6 大注意事项】1、鉴别品种：乳酸奶没有保健效果。2、要饭后 2 小时左右饮用。3、饮后及时漱口。4、不要加热：乳性乳酸菌 如经加热或开水稀释会大量死亡。5、不宜与氯霉素、红霉素等抗生素、磺胺类药物和治疗腹泻的收敛剂次碳酸、鞣酸蛋等药物同服。6、不要给婴儿喂食。	"Kids drink yoghurt 6 big issues to pay attention" 1. Discern the types of yoghurt: Probiotic milk does not have the positive health effects. 2. Drink two hours after meals. 3. Gargle one's mouth after meals. 4. Lactobacillus will die in bulk if heated or diluted in water. 5. Do not consume yoghurt with medications such as chloramphenicol, erythromycin and other antibiotics, sulfonamides, and subcarbonates, albumin and other astringents (that are used to treat diarrhea). 6. Do not feed babies with yoghurt.	2(0.44%)	Original Weibo post	Alternative health information
【吃甜食不发胖最佳时间】1. 体育运动前；2.疲劳饥饿时；3. 游泳前；4. 呕吐或腹泻；5. 头晕恶心；6.吃得太油腻时，喝杯芹菜汁可燃烧脂肪;8. 吃火锅后喝点酸奶有效保护胃肠道黏膜；	"The best time to eat sweet food and won't get fat." 1. Before exercises. 2. When one is tired and hungry. 3. Before swimming. 4. Vomiting or diarrhea. 5. Dizziness and nausea. 6. After consuming too much oily food, drinking a cup of celery juice can help burn fat. 8. After having a hotpot a meal, drinking a bit of yoghurt can effectively protect the mucosa of gastrointestinal tract.	2(0.44%)	Original Weibo post	Alternative health information
喝豆浆的禁忌 1.豆浆性质偏寒，消化不良、嗝气和肾不好，腹胀、腹泻的人最好少喝豆浆。有痛风症状、虚寒体质者都不适宜饮用豆浆。	Taboo for drinking soya milk 1. The nature of soya milk is relatively "cold". It is best for people with indigestion, burping, poor kidney, bloating or diarrhea should drink less soya drink. Soya milk is not suitable for people who have symptoms of gout, or of a "cold" constitution. 2. Do not add brown sugar to soya milk. 3.	2(0.44%)	Original Weibo post	Alternative health information

2.豆浆里不能加红糖。3.不能用暖瓶装豆浆。4.煮豆浆要敞开锅盖，小火煮熟。5.豆浆一定不要与红霉素等抗生素一起服用 6.豆浆里不能加鸡蛋 7.不能喝过量	Do not put soya milk into thermos flasks. 4. When one cooks soya milk, one should leave the lid open and use low power to cook. 5. One must not take erythromycin or other antibiotics with soya milk. 6. Do not add chicken eggs to soya milk. 7. Do not drink too much soya milk.			
大家常带狗狗去户外玩耍，您对植物与狗狗的关系了解有多少？万年青:恶心、呕吐、腹痛、腹泻，严重逐渐昏迷致死。	We often take dogs to play outdoor. How much do you know about the relationship between plants and dogs? Evergreen: nausea, vomiting, abdominal pain, diarrhea. In severe cases, [the dog] will gradually goes into coma and then death.	2(0.44%)	Original Weibo post	Alternative health information about pet dog
【防暑降温常备药】1、仁丹：主治中暑受热引起的头昏脑涨、胸闷、腹痛腹泻；2、十滴水：适于中暑所致头昏、恶心呕吐、胸闷腹泻；3、藿香正气水：适于暑天因受寒所致的头昏、腹痛、呕吐、腹泻突出者；4、清凉油：可治疗暑热引起的头昏头痛，或因贪凉引起的腹泻；5、无极丹：清热祛暑、镇静止吐。	“Medication that should be kept standy-by to prevent heatstroke and lower the temperature” 1. *Ren Dan*: primarily for the treatment of dizziness, chest tightness, abdominal pain and diarrhea, caused by heatstroke; 2. *Shi Di Shui*: Suitable for the treatment for dizziness, nausea, vomiting, chest tightness and diarrhea caused by heatstroke; 3. *Huo xiang zhengqi shui*: Suitable for the treatment of dizziness, abdominal pain, vomiting and diarrhea caused by catching cold in the summer; 4. Soothing oil: can treat dizziness and headache caused by the summer heat, or diarrhea caused by catching cold; 5. *Wu Ji Dan*: To soothe the summer heat and stop vomiting.	2(0.44%)	Original Weibo post	Alternative health information / Traditional Chinese Medicine
【低血压者喝菊花茶别超三朵】菊花有降血压功效，血压偏低老人和血压正常老人，不宜每天喝菊花茶，可以隔三差五地喝，每天不要超过三朵。体质偏寒的人喝菊花茶的时候要放点枸杞，而脾胃虚寒的人只在苦夏难耐时适量饮用。体虚、脾虚、胃寒病者、容易腹泻者，最好不要常饮菊花茶。	“People with low blood pressure should not drink chrysanthemum tea with more than three chrysanthemum flowers.” Chrysanthemum is effective to lower blood pressure. Elderly people whose blood pressure is relatively low or normal, are not suitable to drink chrysanthemum tea every day. They can drink chrysanthemum every few days. Each day, one should not consume more than three chrysanthemum flowers. People whose bodily constitution is relatively “cold” should add some Lycium to chrysanthemum tea. People whose spleen and stomach are weak and “cold” should only drink an appropriate volume of chrysanthemum tea in summer when the heat is intense. If one’s body is weak, or whose spleen is weak, or who has stomach	2(0.44%)	Original Weibo post	Alternative health information

	illness, or who is prone to diarrhea, one should avoid chrysanthemum tea.			
Other posts on alternative health information		38 (8.37%)	38 invidiual posts of entirely different content	Alternative health information
**Advertisements**	[There were 4 Weibo posts of advertisements of products or services.]	**4 (0.88%)**		
**Personal comments**		**18 (3.96%)**		
双标教元老呗。右边+1，以及我开始觉得这科学神棍脑子有问题是从他说痛恨滥用抗生素哪怕自己儿子都腹泻到便血了也没让医生开抗生素。//@[user1] 我只想说当初怎么没一锤子敲死这祸害 //@[user2] 这是我见过的最绵密的打脸帖，啪啪啪。 //@[user3] 这是我目前所看到最完整剖析方舟子的文章。	[This Weibo post contained abuses targeted against a famous Chinese intellectual. We left it untranslated.]	4 (0.88%)	http://blog.sina.com.cn/s/blog_53e38e5801013d8u.html	Personal comment
让自己成为普通人 做自己擅长的事 幸福感就会降临。若你出现以下征象意味着需要放慢脚步：乏力、头晕失眠、记忆力下降、莫名腹泻便秘、体重（肥肉）增加、性欲下降、情绪不易控制、反复无节制消费、不能静下来读完甚至一篇文章等等，请慢下脚步。这个地球离了谁都转	Let yourself be a common person. Do what you are good at. The feeling of bliss will arrive. If you experience the following symptoms, they imply that you need to slow down your pace: fatigue, dizziness, insomnia, memory loss, diarrhea or constipation due to unknown reasons, weight gain, decreased libido, difficulty to control one's mood, repeated uncontrolled spending, or inability to calm down to finish reading even one article etc. Please slow down your pace. The earth will still rotate in the absence of anyone.	3(0.66%)	Original Weibo post	Personal comment
【腹泻】如有发生，不妨一试。	"Diarrhea" If it happens, let's try this.	2(0.44%)	Original Weibo post	Personal comment
额，看着都得腹泻！	Huh, acquiring diarrhea while looking!	1(0.22%)	Original Weibo post	Personal comment
天呐，我家灰灰腹泻、吐了	O heaven, Huihui in my family experiences diarrhea	1(0.22%)	Original Weibo post	Personal

不是因为这个吧？	and vomiting. Wouldn’t it be a result of this?			comment
继续加大腹泻流量！	Continue to increase the flow of diarrhea!	1(0.22%)	Original Weibo post	Personal comment
扁桃体发炎、腹泻、头痛、鼻塞、痛经，好久没生病了，病就病么，你们还组团来了。群殴我一个很光荣吗？最可恶的是，我现在百稿缠身，病不起啊病不起…	Tonsil inflammation, diarrhea, headache, nasal congestion, dysmenorrhea. For a long time I have not been sick. It is ok to get sick, how come you come in a group! Is it a glory to gang fight me? The worst is that I have so many manuscripts to write right now, I cannot fall ill…	1(0.22%)	Original Weibo post	Personal comment
#银魂# 小三如痛经 反复来扰乱 缘分如腹泻 来去匆匆一场虚幻。	# Silver soul# Affairs is like dysmenorrhea. It disturbs one’s life repeatedly. *Yuanfen* is like diarrhea. It comes and goes in a hurry. It is all emptiness.	1(0.22%)	Original Weibo post<br>*Yuanfen* may be translated as the chances of affinity between two persons. Sometimes it is translated as fate.	Personal comment
我在等待肚子痛 腹泻 等等症状。。。好忐忑	I am waiting for symptoms such as abdominal pain, diarrhea etc.…very unease.	1(0.22%)	Original Weibo post	Personal comment
不会是让你再腹泻十次吧？还有人敢喝吗？	Wouldn’t it cause you to have diarrhea ten more times? Will anyone dare to drink it?	1(0.22%)	Original Weibo post	Personal comment
那些童年父母交代的事情：不能用手指月亮，耳朵会缺会掉；吃稀饭不要用筷子指小孩，小孩会拉肚子；吃水果一定要把籽吐掉，不然身体里会长出各种水果；踩影子会长不高；你是妈妈捡来的；玩火会尿床；草莓味的思密达不是饮料是治腹泻的。	Those things that parents instructed us when we were young: Do not use fingers to point to the moon, otherwise the ears will be damaged and will fall off. Do not use chopsticks to point at other kids when one is eating congees, otherwise the kids will have diarrhea. One must spit away the seeds when one is consuming fruits, otherwise different types of fruits will grow out of your body. Do not step on one’s shadows, otherwise one would not grow tall. You are picked up somewhere by your mum. If one plays with fire, one will wet one’s bed. Strawberry-favored Smecta is not a drink but medicine to treat diarrhea.	1(0.22%)	Original Weibo post	Personal comment
家里应该常备哪些宝宝感冒、发烧、腹泻的药呢？	What time of stand-by medication should be kept at home to treat babies’ common cold, fever and diarrhea?	1(0.22%)	Original Weibo post	Personal comment

42. Hand-foot-and-mouth disease

Figure S42. Daily count of Weibo posts with the keyword for "Hand-foot-and-mouth disease" (手足口病) in 2012 among Sina Weibo users with 1000 followers or more.

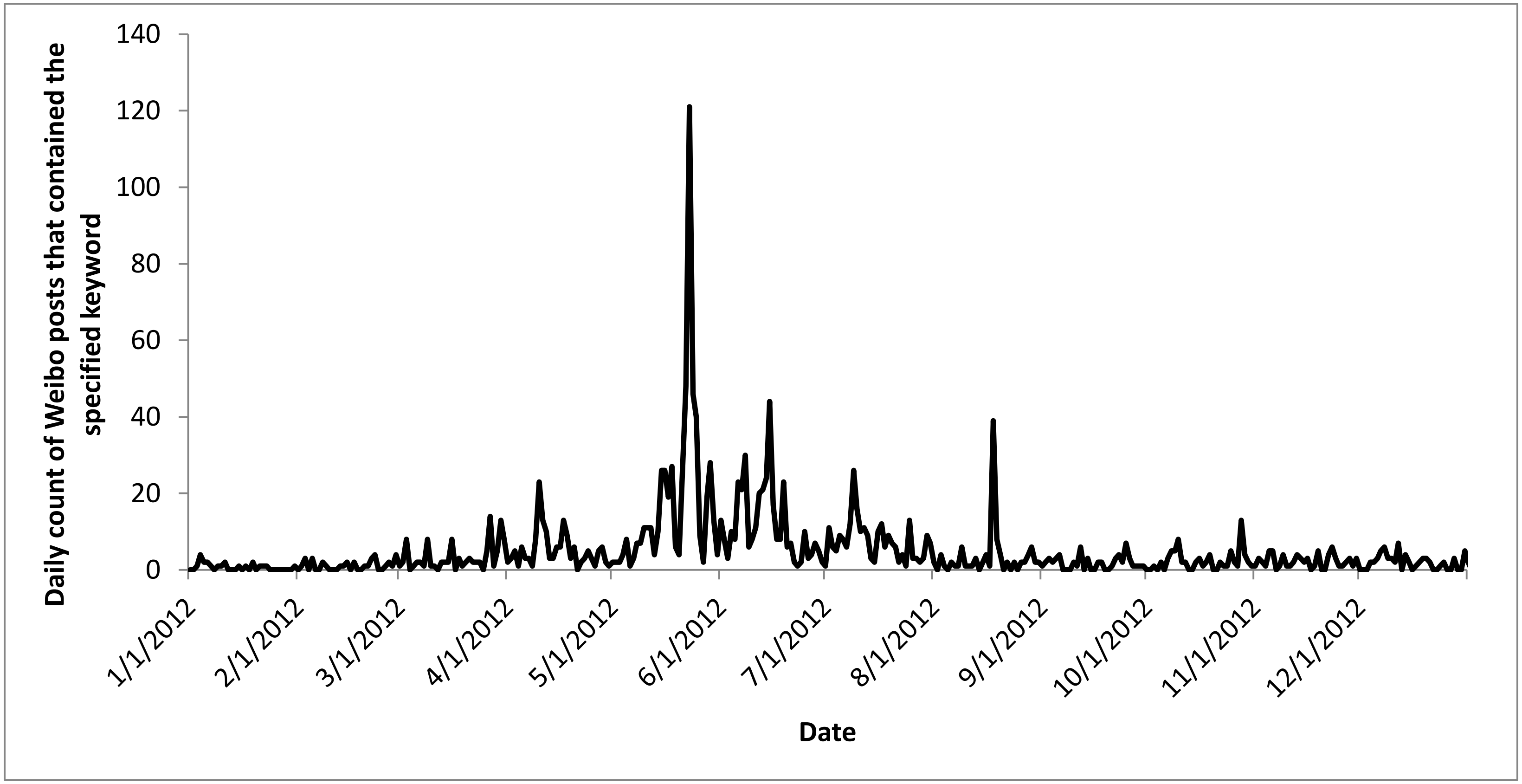

Content analysis was performed for the Weibo posts generated on the peak date of May 23, 2012.

Peak Date: May 23, 2012.

Total count: 121

Summary: Of the 121 Weibo posts mentioning the keyword for "Hand-foot-and-mouth disease", created on May 23, 2012, 63 posts were about the news of a cashier at a hospital in Shenzhen, Guangdong Province, who apparently wrote the following note and posted it at the cashier's counter: "Regardless whether you have a fever, diarrhea, cough or hand-foot-and-mouth disease, you will have to wait for 6-9 hours. If you can wait, then register. If you cannot wait, please go to another hospital!" That cashier was later reprimanded by the hospital authorities for such an action. This piece of news attracted a lot of attention from the mass media and Weibo users. This was another incident reflecting the tension between patients and staff members of a hospital.

There were 22 posts about the news of the epidemic of hand-foot-and-mouth disease across various provinces in China, 27 posts on the prevention of hand-foot-and-mouth (health education) and another 9 posts that shared personal experience of or comments on the disease.

Table S42. Sample Weibo entries that contained the keyword for "Hand-foot-and-mouth disease" (手足口病) on May 23, 2012.

Sample Weibo posts on that day	English translation / description	Count (%)	Sources / References / Notes	Category
【深圳儿童医院窗口告示：不能等，就去其他医院】"不管是你是发烧、拉肚子、咳嗽或是手足口病，都要等 6-9 个小时，如果你能等待，就挂号，如果你不能等待，请去其他医院！"日前，深圳儿童医院的挂号窗口的告示引发非议。昨日院方回应，此事为收费窗口工作人员所为，已严肃处理。 http://t.cn/zO1icqf	"A notice at a counter at Shenzhen Children's Hospital: If you cannot wait, go to another hospital" "Regardless whether you have a fever, diarrhea, cough or hand-foot-and-mouth disease, you will have to wait for 6-9 hours. If you can wait, then register. If you cannot wait, please go to another hospital!" Recently, a notice at a counter at Shenzhen Children's Hospital has attracted a lot of criticism. Yesterday, the hospital responded that this was done by a staff member who worked at the cashier's counter. The hospital had dealt with this incident seriously.	63 (52.07%)	The sample Weibo post contains a link to China National Radio: http://www.cnr.cn/gundong/201205/t20120523_509675084.shtml	Social issue / News (Relationship between a hospital staff member and patients)
【广西、广东等地区手足口病高发】来自卫生部的消息说，全国目前手足口病已经进入疫情的高发期。截止 5 月 19 日，全国累计报告手足口病 707244 例。其中，重症 7190 例，死亡 203 人。4 月 12 日，病情、病例增加幅度较大，广东、广西、湖南等地区疫情上升明显。 http://t.cn/zO3sxaC	"Hand-foot-and-mouth disease epidemic. High incidence in Guangxi, Guangdong and other regions"] According to information from the Ministry of Health, hand-foot-and-mouth disease has entered its peak season nationally. Up to May 19, the national cumulative incidence reaches 707244 cases. Of which, there were 7190 severe cases and 203 deaths. On April 12, The severity and the number of cases increase with a bigger margin. There was an obvious increase in number of cases in Guangdong, Guangxi, Hunan and other regions.	14 (11.57%)	The sample Weibo post contains a link to China Central Television: http://news.cntv.cn/china/20120522/112924.shtml	News about outbreaks in various provinces *

	http://t.cn/zO3sxaC			
【永州千名儿童突发手足口病 唯一定点医院爆棚】湖南郭女士反映自己的孩子得了手足口病，可是当地仅有一家收治手足口病患儿的定点医院，已经爆满，挂号、诊治都需要排长队，更不要说住院。当地卫生局回应称，现在病人确实比较多。http://t.cn/zO16nWz	"At Yongzhou, a thousand children were suddenly infected with hand-foot-and-mouth disease. The only designated hospital is full." Ms. Guo from Hunan told us that her child is infected with hand-foot-and-mouth disease. However, there is only one hospital that is designated to treat hand-foot-and-mouth disease and it has been full already. There is a long queue to write for registration and treatment, let alone to be taken as in-patients. The health bureau there responded that, currently there are relative more patients. http://t.cn/zO16nWz	8 (6.61%)	The sample Weibo post contains a link to China Central Television: http://news.cntv.cn/china/20120523/120938.shtml	News about an outbreak in Yongzhou, Hunan
#手足口病预防# 每年 4-6 月是手足口病高发季节，部分地区还会出现秋冬季小高峰。发病人群以 5 岁及以下儿童为主，同一儿童可因感染不同型肠道病毒而出现多次发病。	# Hand-foot-and-mouth disease prevention # Every year from April to June, it is the peak season for hand-foot-and-mouth disease. A minor peak may appear in the autumn and the winter in some regions. The symptomatic age group is primarily children of 5 years of age or below. The same child can be infected with different enteric virus and fall ill multiple times.	27 (22.31%)	Original Weibo post	Health education **
#预防手足口病# 刚去幼儿园接儿子，老师告诉我一个吓人的消息，我班仅剩 11 名孩子中一位今中午突然手心长了很多红点，下午去医院确诊感染手足口病。回家我放下所有事情，第一件事彻底清洁手，接着补充一杯白开水，家中熏好白醋，然后给孩子洗澡，接着一杯新奇士柠檬橙汁补充大量 VC,明天所有衣物被套消毒	# Prevent hand-foot-and-mouth disease # Just went to kindergarten to pick up my son. The teacher told me an astonishing news. Of the 11 kids that remain in our class, one suddenly had a lot of red spots appearing in his/her palm at noon. In the afternoon, the kid was confirmed to have hand-foot-and-mouth disease when s/he was taken to the hospital. When we return home, I leave everything aside. The first thing is to thoroughly clean my hands. Next, I drink a glass of water. I fume my home with white vinegar. And then I gave my son a bath, followed by a cup of Sunkist Lemon Orange Juice to supplement a large quantity of Vitamin C. Tomorrow, I will have all clothes and duvet covers disinfected.	9 (7.44%)	Original Weibo post	Personal experience / comment **

* This is a group of different Weibo posts describing outbreaks in various provinces of China. ** This indicates groups of different Weibo posts that share similar themes, but of literal differences.

References

1. National People's Congress (1989, revised 2004) Law of the People's Republic of China on Prevention and Treatment of Infectious Diseases.
2. National People's Congress (1989, revised 2004) Law of the People's Republic of China on Prevention and Treatment of Infectious Diseases [official English translation]. Available: http://www.npc.gov.cn/englishnpc/Law/2007-12/12/content_1383919.htm
3. Wheeler C, Vogt TM, Armstrong GL, Vaughan G, Weltman A, et al. An outbreak of hepatitis A associated with green onions. N Engl J Med 2005; 353: 890-897.
